# Protein arginine methyltransferase 3 promotes glycolysis and hepatocellular carcinoma growth by enhancing arginine methylation of lactate dehydrogenase A

**DOI:** 10.1002/ctm2.686

**Published:** 2022-01-28

**Authors:** Yu Lei, Ping Han, Yu Chen, Han Wang, Shuhui Wang, Muru Wang, Jingmei Liu, Wei Yan, Dean Tian, Mei Liu

**Affiliations:** ^1^ Department of Gastroenterology, Tongji Hospital of Tongji Medical College Huazhong University of Science and Technology Wuhan Hubei Province China

**Keywords:** hepatocellular carcinoma (HCC), lactate dehydrogenase A (LDHA), glycolysis, protein arginine methyltransferase 3 (PRMT3)

## Abstract

**Background:**

Protein arginine methylation has emerged a pivotal role in cancer progression. However, the role of protein arginine methyltransferase 3 (PRMT3) in hepatocellular carcinoma (HCC) remains unknown.

**Methods:**

The expression pattern of PRMT3 in HCC was analysed using quantitative real‐time‐polymerase chain reaction (qRT‐PCR), Western blotting and immunohistochemistry assays. Loss‐ and gain‐of‐function experiments were carried out to determine the oncogenic role of PRMT3 in HCC. Glucose consumption and lactate production assays, seahorse bioscience, mass spectrometry, co‐immunoprecipitation, metabonomic analysis and site‐specific mutation experiments were used to explore the underlying molecular mechanisms. Furthermore, a xenograft mouse model was established to investigate the effects of PRMT3 and its inhibitor, SGC707, treatment on tumour growth in vivo.

**Results:**

The expression of PRMT3 was significantly upregulated in HCC, with high expression of which correlated with poor prognosis. PRMT3 knockdown led to the decrease in proliferation, glycolysis of HCC cells and tumour growth, whilst its overexpression showed opposite results. The catalytic activity of PRMT3 was important in mediating these biological processes. Mechanistically, our data showed that PRMT3 interacted with and mediated asymmetric dimethylarginine (ADMA) modification of lactate dehydrogenase A (LDHA) at arginine 112 (R112). Compared with LDHA‐wild‐type (LDHA‐WT) cells, LDHA‐R112K‐mutant‐expressing HCC cells exhibited a decrease in lactate dehydrogenase (LDH) activity, HCC cell glycolysis and proliferation. Furthermore, the administration of SGC707, a selective inhibitor of PRMT3, disrupted the PRMT3‐mediated LDHA methylation and abolished PRMT3‐induced HCC glycolysis and tumour growth.

**Conclusions:**

Our results suggested a novel oncogenic role of PRMT3 in HCC, and it could be a promising therapeutic target for HCC by linking post‐translational modification and cancer metabolism.

## Graphical abstract

Our results suggest that PRMT3 promotes glycolysis and HCC growth by enhancing arginine methylation of LDHA, and is a potential biomarker for prognosis of HCC patients. The administration of PRMT3 inhibitor, SGC707, effectively attenuated PRMT3‐induced HCC glycolysis and tumour growth. Our study provides evidence for developing a novel potential target linking post‐translational modification and cancer metabolism for HCC therapy.

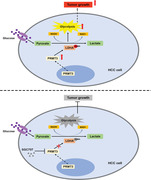

## 1 INTRODUCTION

Protein arginine methylation is an important post‐translational modification that plays a critical role in various cell processes, including gene expression, DNA repair and immune surveillance.^1^ The family of protein arginine methyltransferases (PRMTs) is the major ‘writer’ of arginine methylation in mammals.^1^, ^2^ A growing number of evidence has defined the importance of PRMTs in multiple diseases, especially in cancers.^3^

Amongst the nine identified mammalian PRMTs, PRMT3 differs from the others in some ways, though it belongs to the type I PRMT class of enzymes, which are primarily responsible for catalysing the asymmetric dimethylarginine (ADMA) modification of arginine residues on proteins.^2^ PRMT3 has a unique C2H2 zinc finger domain crucial for substrate recognition, which is not presented in other PRMTs. Another distinction is that PRMT3 is mainly localised in the cytoplasm under physiological conditions.^4^, ^5^ The pathophysiological role of PRMT3 still remains unclear because of the limited number of substrates identified. Ribosomal protein S2 (RpS2) was the first reported substrate for PRMT3 in the cytoplasm,^6^ followed by several other PRMT3 substrates reported, such as nuclear poly(A) binding protein (PABPN1), heterogeneous nuclear ribonucleoprotein A1 (hnRNPA1) and glyceraldehyde‐3‐phosphate dehydrogenase (GAPDH).^7^, ^8^, ^9^ Furthermore, PRMT3 was reported to function as a co‐activator of the liver X receptor (LXR) to facilitate hepatic lipogenesis in the nucleus, indicating the role of PRMT3 independent of methylation.^10^ Recently, Min et al.^11^ have reported that PRMT3 can activate miR‐3648 expression by promoting histone H4 arginine 3 asymmetric dimethylation (H4R3me2a), which revealed the potential impact of PRMT3 on histone methylation. Importantly, though the overexpression of PRMT3 has been detected in pancreatic cancer and is associated with chemoresistance,^8^ the role of PRMT3 in other human cancers still remains elusive to date.

Metabolism reprogramming has been recognised as an emerging hallmark of cancer, and aerobic glycolysis, also known as Warburg effect, is one of the most representative types of metabolic reprogramming.^12^ In this process, rapidly dividing cells convert the most of the glucose into lactate, regardless of availability of oxygen. Meanwhile, intermediate metabolites generate large amounts of reducing equivalents and macromolecules that support cancer cell proliferation and tumour growth.^13^ The liver is an important metabolic organ that coordinates a variety of metabolic activities. For example, the liver is critical in maintaining glucose homeostasis by controlling multiple glucose metabolism pathways, including gluconeogenesis, glycogenolysis and glycolysis.^14^, ^15^ It is also involved in various processes of lipid metabolism, including lipid uptake, synthesis and export, fatty acid oxidation, lipid droplets formation and catabolism.^16^, ^17^ Metabolic abnormalities participate in driving both hepatocellular carcinoma (HCC) initiation and malignant progression.^18^, ^19^ For instance, loss of branched‐chain amino acid catabolism has been observed in hepatocarcinogenesis, thereby promoting HCC development and progression.^20^ Enhanced glycolysis is frequently observed in HCC, which is linked to cancer aggressiveness and poor prognoses of patients with HCC.^21^ Although several recent studies have identified the relationship between protein arginine methylation and glycolysis in HCC, investigations on this field have been limited to only part of the PRMT family members, and further explorations need to be conducted.^22^, ^23^

In this study, we found that PRMT3 expression was upregulated in HCC, with high expression of which correlated with poor prognosis in HCC patients. A novel oncogenic function of PRMT3 was also revealed through in vitro and in vivo experiments. Mechanistically, we demonstrated that PRMT3 promoted HCC growth by enhancing glycolysis in HCC via mediating arginine methylation of lactate dehydrogenase A (LDHA). Furthermore, the administration of PRMT3 inhibitor, SGC707, disrupted the increased ADMA modification mediated by PRMT3, and abolished PRMT3‐induced HCC glycolysis and tumour growth. These findings suggested that PRMT3 played a vital role in HCC, and might be a promising therapeutic target for HCC, linking post‐translational modification and cancer metabolism.

## 2 METHODS AND MATERIALS

### 2.1 Cell culture and reagents

The HCC cell lines were kept in the Institute of Liver and Gastrointestinal Diseases (Tongji Hospital, Huazhong University of Science and Technology). Chang liver, Huh7, MHCC97H, HCCLM3 and SK‐hep‐1 were cultured in DMEM medium, HL7702 and SNU398 were cultured in RPMI 1640 medium, and Hep3B and PLC/PRF/5 were cultured in MEM medium. These cell lines were incubated in at 37°C, 5% CO_2_. All of the medium were added 10% fetal bovine serum (Invitrogen Gibco, USA). SGC707 (MCE, cat# HY‐19715), 2‐deoxy‐D‐glucose (2‐DG) (MCE, cat# HY‐13966), oligomycin (MCE, cat# HY‐N6782) and XY‐1 (MCE, cat# HY‐19714) were purchased from MedChemExpress. Cycloheximide (Selleck, cat# HY‐12320) was purchased from Selleck.

### 2.2 Human samples

The human samples were obtained from patients who underwent excisional surgery of HCC in Tongji Hospital. No patients received any pre‐operative adjuvant therapy. The diagnosis of HCC was confirmed pathologically. The present study was approved by the Ethics Committee of Tongji Hospital and complied with the World Medical Association Declaration of Helsinki.

### 2.3 Immunohistochemistry assays

All tissues were paraformaldehyde‐fixed and paraffin‐embedded. Human tissues were stained for PRMT3 (Abcam, cat# ab191562) expression, and animal xenograft tissues were stained for Ki‐67 (Abcam, cat# ab15580). The SP Detection System Kits were used for immunohistochemistry (IHC) staining (ZSGB‐BIO, cat# SP‐9000) following the instructions. The intensity of staining was divided into 0 (negative), 1 (weak), 2 (medium) and 3 (strong). The percentage of positive cells was scored from 0 to 4 (0%, 1%–25%, 26%–50%, 51%–75%, 76%–100%). Total score ranged from 0 to 12. The low (0–3) staining or high (4–12) staining classification of each sample was calculated by multiplying these two scores.

### 2.4 Immunoprecipitation analysis

Cells were lysed on the ice with NP‐40 solution for 30 min. After centrifugation, supernatants were mixed with Protein A/G Magnetic Beads (MCE, cat# HY‐K0202) and appropriate antibodies PRMT3 (Abcam, cat# ab191562), LDHA (Proteintech, cat# 19987‐1‐AP), HA‐tag (Proteintech, cat# 51064‐2‐AP), Flag‐tag (Proteintech, cat# 20543‐1‐AP) and Normal Rabbit IgG (Cell Signaling Technology, cat# 2729) overnight at 4°C. The magnetic beads were separated from the mixture, and incubated with the supernatants. Precipitated proteins were washed with lysis buffer three times. Samples were collected for subsequent Western blotting analyses and mass spectrometry (MS) analysis.

### 2.5 MS analysis and modification residue identification

For PRMT3 interacting proteins identification, the protein samples were enriched by immunoprecipitation (IP) assays mentioned above. Then, the immunoprecipitates were separated by SDS‐PAGE. The gel bands were proteolysed and dried. Then, the dried peptide samples were reconstituted and centrifuged. The supernatant was taken for MS detection. Peptides were analysed by MS (Beijing Genomics Institution, Shenzhen, China). The results of protein identification were obtained through aligning experimental MS/MS data with theoretical values from the database, such as UniProt protein database.

For modification residue identification, MS data were then searched against the target protein database with Proteome Discoverer (Thermo Fisher Scientific). Carbamidomethyl (C) was marked as a static modification, and the oxidation (M), deamidation (N, Q), phospho (S, T, Y), methyl (K/R) and acetyl (K) were set as dynamic modification.

### 2.6 Animal experiments

All animal experimental procedures were conducted following National Institutes of Health Guide for the Care and Use of Laboratory Animals. The protocol was approved by the Committee of Ethics of Animal Experiments of Tongji Hospital, Huazhong University of Science and Technology. Five‐week‐old BALB/C male nude mice were raised in specific pathogen‐free conditions. In subcutaneous xenograft tumour model, 2 × 10^6^ indicated stable HCC cells were injected subcutaneously. The width and length of the subcutaneous tumours were measured and recorded every 3 days. After about 30 days, mice were sacrificed, and tumour tissues were resected, weighted and photographed. *XY* ^2^/2 was used to calculate tumour volumes (*X* is the largest diameter and *Y* is the smallest diameter of two perpendicular diameters).

### 2.7 Cellular energy metabolism analysis

Glycolysis Stress Test Kit (Agilent Technologies, cat# 103020‐100) was used for extracellular acidification rate (ECAR) measurement, whilst Cell Mito Stress Test Kit (Agilent Technologies, cat# 103015‐100) was used for oxygen consumption rate (OCR) analysis, by extracellular flux (XF24) analyser (Seahorse Bioscience). In short, HCC cells were seeded in 200 μl culture medium in XF24 plates, and then incubated at 5% CO_2_ and 37°C prior to assay. On the day of the ECAR measurement, cells were washed, resuspended in XF assay medium supplemented with 2 mM glutamine (Agilent Technologies, cat# 103579‐100), and incubated for 30 min without CO_2_ supplement before the probe cartridge calibration was completed. Glycolysis was measured by injection of glucose (10 mM), oligomycin (1.5 μM) and 2‐DG (50 mM). For OCR measurement, cells were washed, then resuspended in XF assay medium supplemented with 2 mM glutamine, 1 mM pyruvate (Agilent Technologies, cat# 103578‐100) and 10 mM glucose (Agilent Technologies, cat# 103577‐100) incubated for 45 min without CO_2_. OCR was measured after injection of oligomycin (1.5 μM), carbonyl cyanide‐4‐trifluoromethoxy phenylhydrazone (1.0 μM) and rotenone/antimycin A (0.5 μM).

### 2.8 Metabonomic analysis

The extraction solution containing isotope‐labelled internal standard mixture was added to the sample in proportion. After vortexing for 30 s, the samples were frozen and thawed three times with liquid nitrogen. The samples were then sonicated in an ice water bath for 10 min. Then, the samples were incubated at −40℃ for 1 h and centrifuged at 12 000 rpm at 4℃ for 15 min. The supernatants were transferred to a new glass bottle for liquid chromatography‐tandem MS (LC‐MS/MS) analysis. The quality control samples were prepared by mixing aliquots of the sample supernatants. LC‐MS/MS analyses were conducted by ultra high performance liquid chromatography (UHPLC) system (Thermo Fisher Scientific), with UPLC BEH amide column combined with Q Exactive HFX mass spectrometer. Electrospray ionisation was utilised to test positive and negative ions. ProteoWizard was utilised to convert the original data into mzXML format, with an internal program for further processing. The program was based on XCMS, and was used for peak detection, extraction, alignment and integration. The internal MS2 database (BiotreeDB) was then applied to metabolite annotations. Metabolite levels were normalised to that of the cell counts. Five replicates per group were analysed. Variable importance values (VIP) in the projection in the first principal component were obtained by orthogonal projections to latent structures‐discriminate analysis (OPLS‐DA) analysis. VIP > 1 and *p* < .05 were marked as the threshold for significant change.

### 2.9 Statistical analysis

Student's *t*‐test was used for quantitative data analysis, and Fisher's exact test was utilised to analyse categorical data. The Kaplan–Meier method (log‐rank test) were used to determine cumulative survival rates. Independent factors influencing survival were determined with the Cox proportional hazards model based on variables selected from univariate analyses. Two‐sided *p* < .05 was considered statistically significant. Statistical analyses were calculated using SPSS software (version 21.0) and Prism 6.0 (GraphPad Software).

More methods and materials used in this study were described in Supporting Information.

## 3 RESULTS

### 3.1 PRMT family genes are dysregulated in HCC

Previous studies have reported the aberrant expression of PRMT family genes in different cancers. First, we analysed the expression of PRMT family members with HCC samples in The Cancer Genome Atlas dataset. The results showed that the mRNA expression of PRMT1 (fold change = 1.54, *p* = .002), PRMT2 (fold change = 1.22, *p* = .0193), PRMT3 (fold change = 1.90, *p* < .001), PRMT4 (fold change = 1.40, *p* < .001), PRMT5 (fold change = 1.34, *p* < .001) and PRMT7 (fold change = 1.21, *p* = .0043) was higher in HCC tissues than in normal liver tissues. In contrast, the mRNA expression of PRMT6 (fold change = 0.78, *p* = .0089) and PRMT9 (fold change = 0.74, *p* < .001) was notably reduced. As PRMT8 is specifically expressed in the brain, it was undetectable both in HCC and normal liver tissues (Figure S1).^24^ Kaplan–Meier analysis revealed that the increased mRNA expression levels of PRMT1/3/5/6 were remarkably associated with shorter survival in HCC patients, whilst HCC patients with higher mRNA expression level of PRMT8 or PRMT9 had a better prognosis (*p* < .05) (Figure S2). These data suggested the widespread dysregulation of arginine methylation in HCC, which is relevant to patients’ survival. Amongst these nine PRMT family genes, we observed that PRMT3 was the most upregulated in HCC tissues and higher expression of PRMT3 was correlated with poor prognosis, which indicating a potential role of PRMT3 in HCC. However, as we reviewed previously, the role of PRMT3 in HCC has not been explored yet, whilst the functions of many other PRMTs have been reported.^25^ Considering this point, we became interested in PRMT3, and focused our attention on it to investigate its potential role in HCC.

### 3.2 PRMT3 is significantly upregulated in HCC and associated with poor prognosis

**FIGURE 1:**
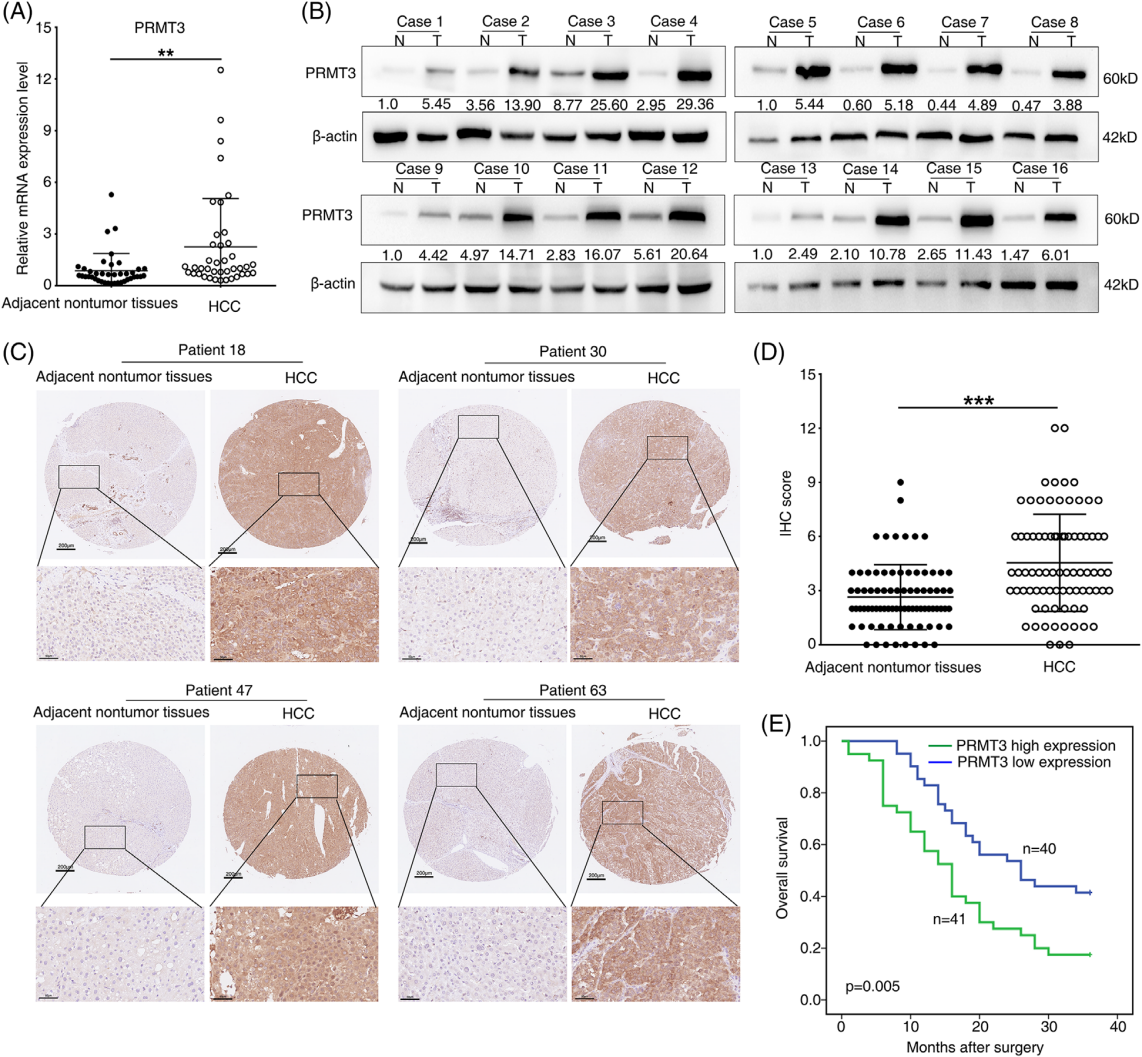
Protein arginine methyltransferase 3 (PRMT3) is significantly upregulated in hepatocellular carcinoma (HCC) and associated with a poor prognosis. (A) Quantitative real‐time‐polymerase chain reaction (qRT‐PCR) analysis of PRMT3 mRNA expression level in 40 pairs of HCC and corresponding adjacent non‐tumour tissues. (B) Western blotting analysis of PRMT3 protein expression level in 16 pairs of HCC and corresponding adjacent non‐tumour tissues. (C) Representative immunohistochemistry (IHC) staining images showing PRMT3 expression in HCC and adjacent non‐tumour tissues (scale bars: 200 μm (upper), 50 μm (lower)). (D) IHC scores of PRMT3 in human HCC cohort (*n* = 81). (E) Kaplan–Meier analysis of the association between PRMT3 expression and overall survival of HCC patients with high or low PRMT3 expression. The data are represented as mean ± S.D. *p < .05, **p < .01, ***p < .001

We, then, analysed PRMT3 mRNA expression in 40 pairs of HCC and matched adjacent non‐tumour tissues using quantitative real‐time‐polymerase chain reaction (qRT‐PCR). The mRNA expression level of PRMT3 was significantly higher in HCC tissues than in corresponding adjacent non‐tumour tissues (Figure 1A). Similarly, Western blotting analyses and IHC assays also showed that PRMT3 protein expression was remarkably higher in HCC tissues compared with matched adjacent non‐tumour tissues (Figure 1B–D). Furthermore, to explore the prognostic value of PRMT3 in HCC, we analysed the relationship between PRMT3 protein expression level and patients’ clinical features in a cohort of human HCC samples by IHC. PRMT3 upregulation was significantly correlated with hepatitis B virus infection, increased tumour number, larger tumour size and higher tumour‐nodule‐metastasis stage (Table 1). Kaplan–Meier analysis showed that patients with high PRMT3 expression had a shorter survival time than those with low expression of PRMT3 (Figure 1E). Univariate and multivariate analyses indicated that high PRMT3 expression was an independent predictor of overall survival in HCC (Table S1). Collectively, these findings indicated that PRMT3 could serve as a potential prognostic biomarker of HCC progression.

**TABLE 1:** Correlation between protein arginine methyltransferase 3 (PRMT3) expression and clinicopathological characteristics of hepatocellular carcinoma (HCC) patients

	PRMT3		
Clinicopathological variables	Low expression (*n* = 40)	High expression (*n* = 41)	*p*‐Value
Age			.585
≤45 years	16	14	
>45 years	24	27	
Sex			.967
Female	5	5	
Male	35	36	
Serum AFP			.913
≤400 ng/ml	20	21	
>400 ng/ml	20	20	
HBsAg			.002*
Negative	3	15	
Positive	37	26	
Tumour number			.022*
Single	35	27	
Multiple	5	14	
Maximal tumour size			.034*
≤5 cm	14	6	
>5 cm	26	35	
TNM stage			.020*
I–II	23	13	
III–IV	17	28	

Abbreviations: AFP, alpha fetoprotein; HBsAg, hepatitis B surface antigen; TNM, tumour‐nodule‐metastasis.

* *p* < .05.

### 3.3 PRMT3 promotes cell proliferation and tumour growth in HCC in vitro and in vivo

**FIGURE 2:**
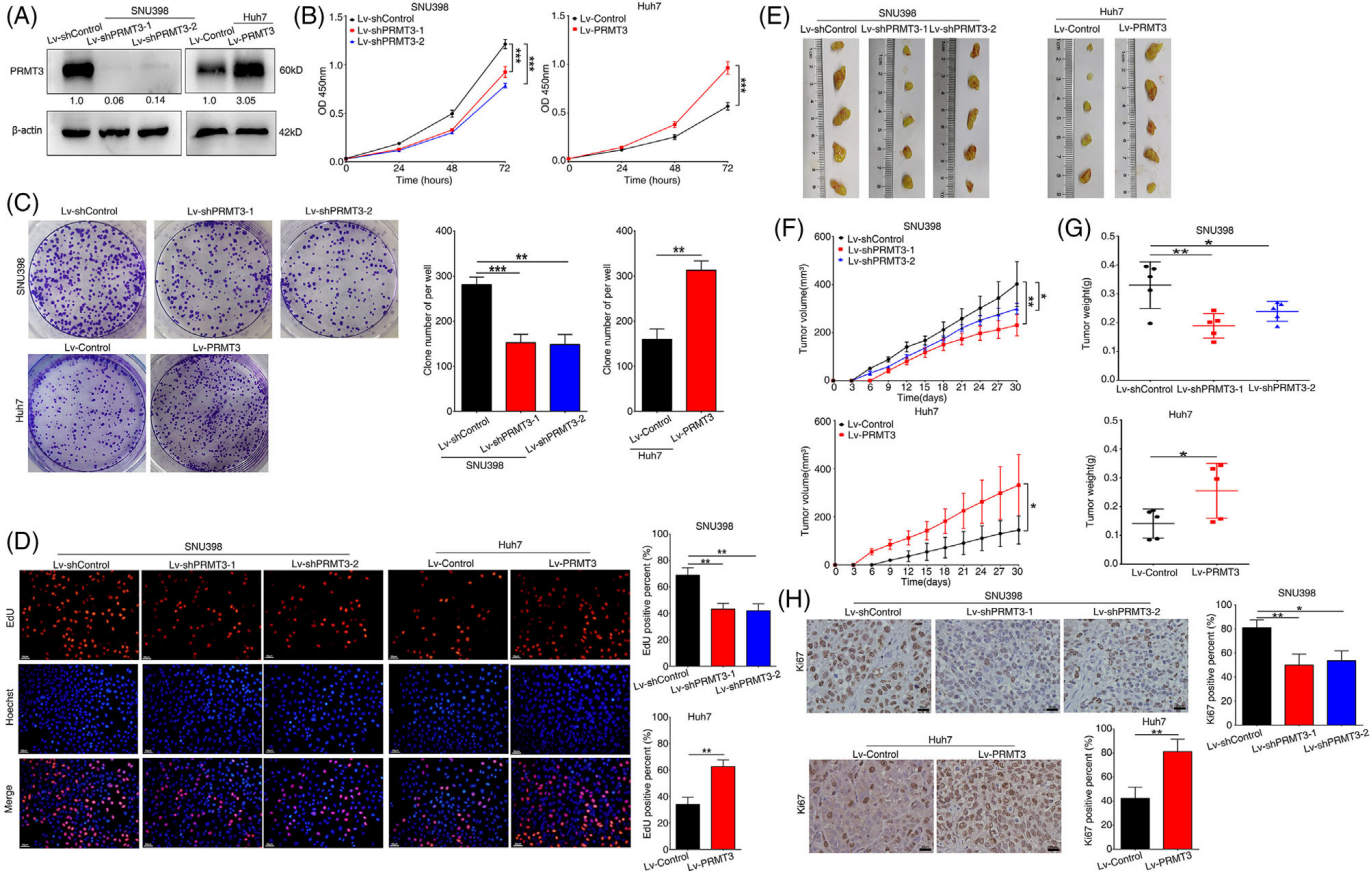
Protein arginine methyltransferase 3 (PRMT3) promotes cell proliferation and tumour growth in hepatocellular carcinoma (HCC) in vitro and in vivo. (A) Western blotting analysis confirmed the expression of PRMT3 in the indicated SNU398 cells (left) and Huh7 cells (right) after lentivirus transfection (*n* = 3). (B) Cell counting kit‐8 (CCK‐8) assays showed the proliferation capacities of SNU398 cells with PRMT3 knockdown and Huh7 cells with PRMT3 overexpression (*n* = 3). (C) Colony formation assays were used to analyse the proliferation abilities of the indicated SNU398 cells and Huh7 cells (*n* = 3). (D) EdU assays were performed to detect the cell proliferation of the indicated SNU398 cells and Huh7 cells (*n* = 3, scale bars: 50 μm). (E–G) The subcutaneous HCC xenograft model was established in nude mice to investigate tumourigenic ability of PRMT3 in vivo. (E) Images of subcutaneous tumours (*n* = 5 for each group). (F) Tumour growth curves of each group. (G) Tumour weight of the tumours. (H) Representative Ki‐67 staining images showed the proliferation of xenografts in the indicated groups (scale bars: 20 μm). The experiments were replicated *n* times, and the data are represented as mean ± S.D. *p < .05, **p < .01, ***p < .001

Cancer cell growth is crucial in the multistep process of tumour progression. To elucidate the contribution of PRMT3 in HCC growth, we conducted loss‐ and gain‐of‐function experiments. First, we analysed the protein expression level of PRMT3 in HCC cell lines, and observed that PRMT3 was upregulated in most HCC cell lines, especially in those with relatively high malignancy (Figure S4A). As the expression of PRMT3 was relatively higher in SNU398 cells and its expression in Huh7 cells relatively low, we selected these two HCC cell lines to establish stable cell lines SNU398‐Lv‐shPRMT3‐1, SNU398‐Lv‐shPRMT3‐2 and Huh7‐Lv‐PRMT3 using lentivirus infection (Figure 2A). The results of cell counting kit‐8 (CCK‐8) and colony formation assays showed that PRMT3 downregulation decreased proliferation ability of SNU398 cells, whereas upregulation of PRMT3 enhanced Huh7 cells proliferation (Figure 2B,C). Similarly, the results of 5‐ethynyl‐2'‐deoxyuridine (EdU) staining assays showed that PRMT3 knockdown markedly reduced the percentage of EdU‐positive cells, whilst there were more cells positive for EdU amongst those with PRMT3 overexpression (Figure 2D). As shown in Figure S3A, changes in global ADMA levels were consistent with cell proliferation capacities in cells with PRMT3 knockdown or overexpression, which indicated that cell proliferation might be related to PRMT3‐linked arginine methylation. Cell apoptosis analyses showed that PRMT3 silence caused the increase in cell apoptosis percentage, whereas PRMT3 overexpression resulted an opposite effect (Figure S5A,C). Moreover, to rule out line‐specific effects and avoid off‐target effects, we also used MHCC97H and Hep3B to establish stable cell lines for functional experiments, and observed similar results (Figures S4B–E and S5B,D).

The subcutaneous HCC xenograft model was established in nude mice to investigate tumourigenic ability of PRMT3 in vivo. The tumours of mice in PRMT3‐knockdown groups grew more slowly and were smaller than those in the control group, whilst PRMT3 overexpression exhibited the opposite effects (Figure 2E–G). Ki‐67 staining of the xenografts showed that downregulation of PRMT3 expression significantly decreased proliferative activity and upregulation of PRMT3 expression increased proliferative activity, as indicated by the percentage of cells positive for Ki‐67 (Figure 2H). These findings suggested that PRMT3 promoted cell proliferation and tumour growth in HCC in vitro and in vivo.

### 3.4 PRMT3 promotes glycolysis in HCC

**FIGURE 3:**
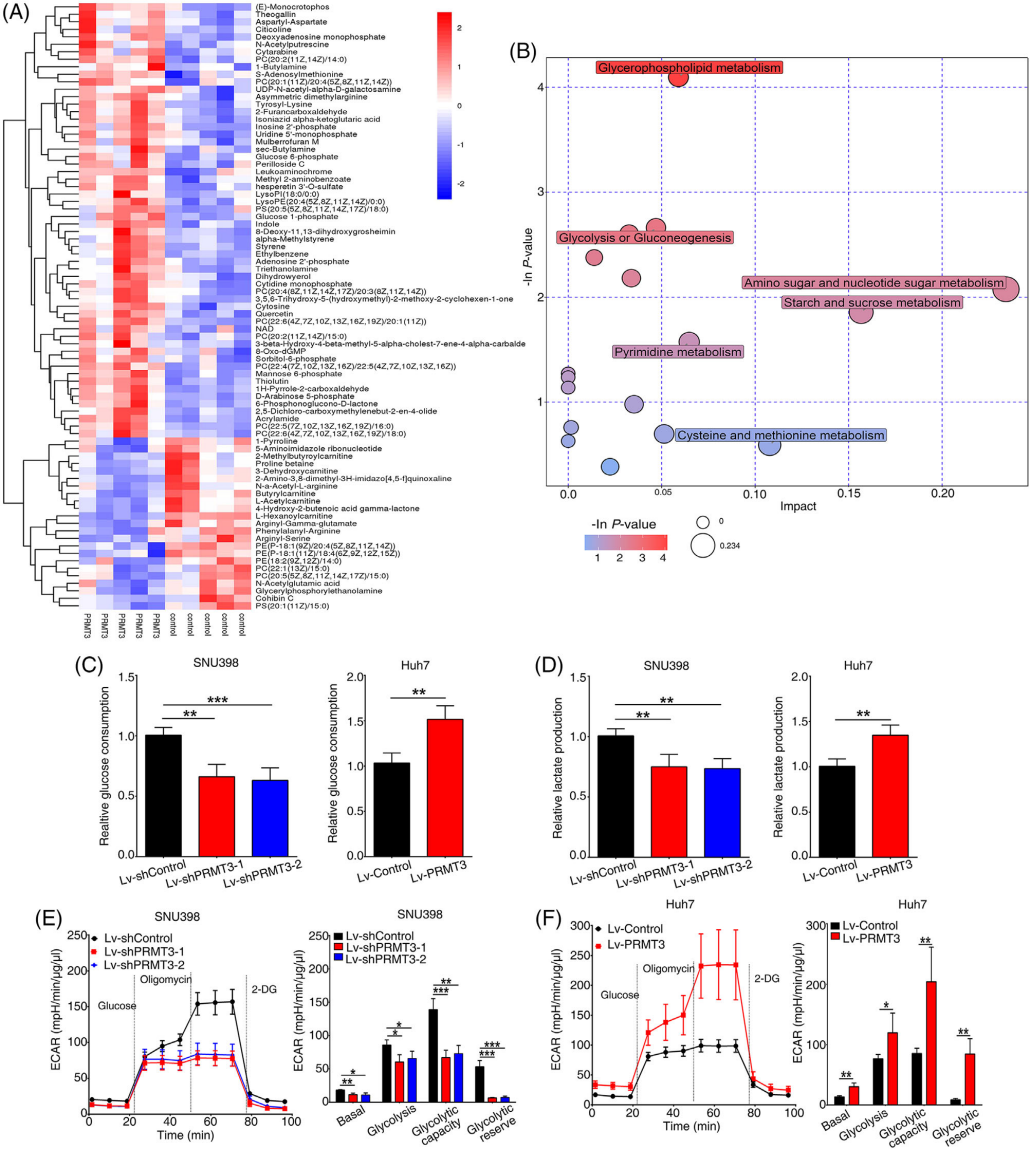
Protein arginine methyltransferase 3 (PRMT3) promotes glycolysis in hepatocellular carcinoma (HCC). (A) The hierarchical clustering heatmap of differentially metabolites between control and PRMT3‐overexpressing Huh7 cells in positive ion mode. (B) Bubble plot of pathway analysis for group control versus PRMT3‐overexpressing Huh7 cells in positive ion mode. The colour depth and bubble size indicated ‐ln *p*‐values and impact of the pathway. (C) Relative glucose consumption of SNU398 cells with PRMT3 knockdown and Huh7 cells with PRMT3 overexpression (*n* = 4). (D) Relative lactate production of the indicated SNU398 cells and Huh7 cells (*n* = 4). (E) Extracellular acidification rate (ECAR) measured in PRMT3 knockdown and control SNU398 cells. Basal ECAR measurement was measured in extracellular flux (XF) assay medium without glucose, following by the addition of glucose (10 mM), oligomycin (1.5 μM) and 2‐deoxy‐D‐glucose (2‐DG) (50 mM). Column statistics of ECAR was shown in the right panel (*n* = 4). (F) ECAR measured in PRMT3‐overexpressing and control Huh7 cells. Column statistics was shown in the right panel (*n* = 4). The experiments were replicated *n* times, and the data are represented as mean ± S.D. *p < .05, **p < .01, ***p < .001

Previous studies have gradually recognised the roles of PRMTs in metabolism, including PRMT3.^1^ During construction of stable HCC cells, we observed that culture medium colour showed decreased acidity after PRMT3 knockdown in SNU398 cells, whilst opposite results were observed in PRMT3‐overexpressing cells (Figure S6A,B). It is reported that the phenol red in cell culture medium exhibits a gradual colour shift from red to yellow at lower pH values due to lactate accumulation.^22^ Given these findings, we wondered whether PRMT3 played a part in the regulation of HCC glucose metabolism. LC‐MS/MS analysis technique was used to detect metabolites changes in HCC cells (Tables S4 and S5). The heatmap of hierarchical clustering analysis demonstrated the primary differential metabolites between control and PRMT3‐overexpressing cells (Figures 3A and S7A), and the differential metabolites were enriched in ‘glycolysis or gluconeogenesis’ pathway both in positive and negative ion modes by metabolic pathway analysis (Figures 3B and S7B). Combining above findings, we focused on exploring the influence of PRMT3 on glycolysis in HCC.

As shown in Figures 3C,D and S9A,B, downregulation of PRMT3 resulted in a significant decrease in glucose consumption and lactate production, whilst PRMT3 upregulation led to an opposite effect. Then, we measured the ECAR and OCR of indicated cells, indicator of glycolysis and oxidative phosphorylation (OXPHOX), respectively. The results showed that PRMT3 knockdown suppressed the glycolysis rate and capacity, whereas overexpression of PRMT3 led to an increase in ECAR (Figures 3E,F and S9C,D). However, the results of OCR analysis showed no noticeable difference between groups, except that few OXPHOX pathways were changed slightly (Figures S8A,B and S9E,F). Therefore, we concentrated on studying the effects of PRMT3 on glycolysis in HCC. Rapid glycolysis provides energy and essential macromolecular substances for cancer cells, thus supporting their growth.^26^ To investigate the role of PRMT3‐promoted glycolysis in HCC cell growth, we added 2‐DG into cell culture medium, a common inhibitor of glycolysis, to suppress the glycolysis in HCC cells. The results showed that 2‐DG treatment inhibited the proliferation of both control and PRMT3‐overexpressing cells, especially in the latter. The inhibitory effect of 2‐DG on PRMT3‐overexpressing cells proliferation is much higher than that of the control group, which suggested that PRMT3 might promote HCC growth by enhancing glycolysis (Figure S10A–C).

### 3.5 PRMT3 interacts with and promotes arginine methylation of LDHA

**FIGURE 4:**
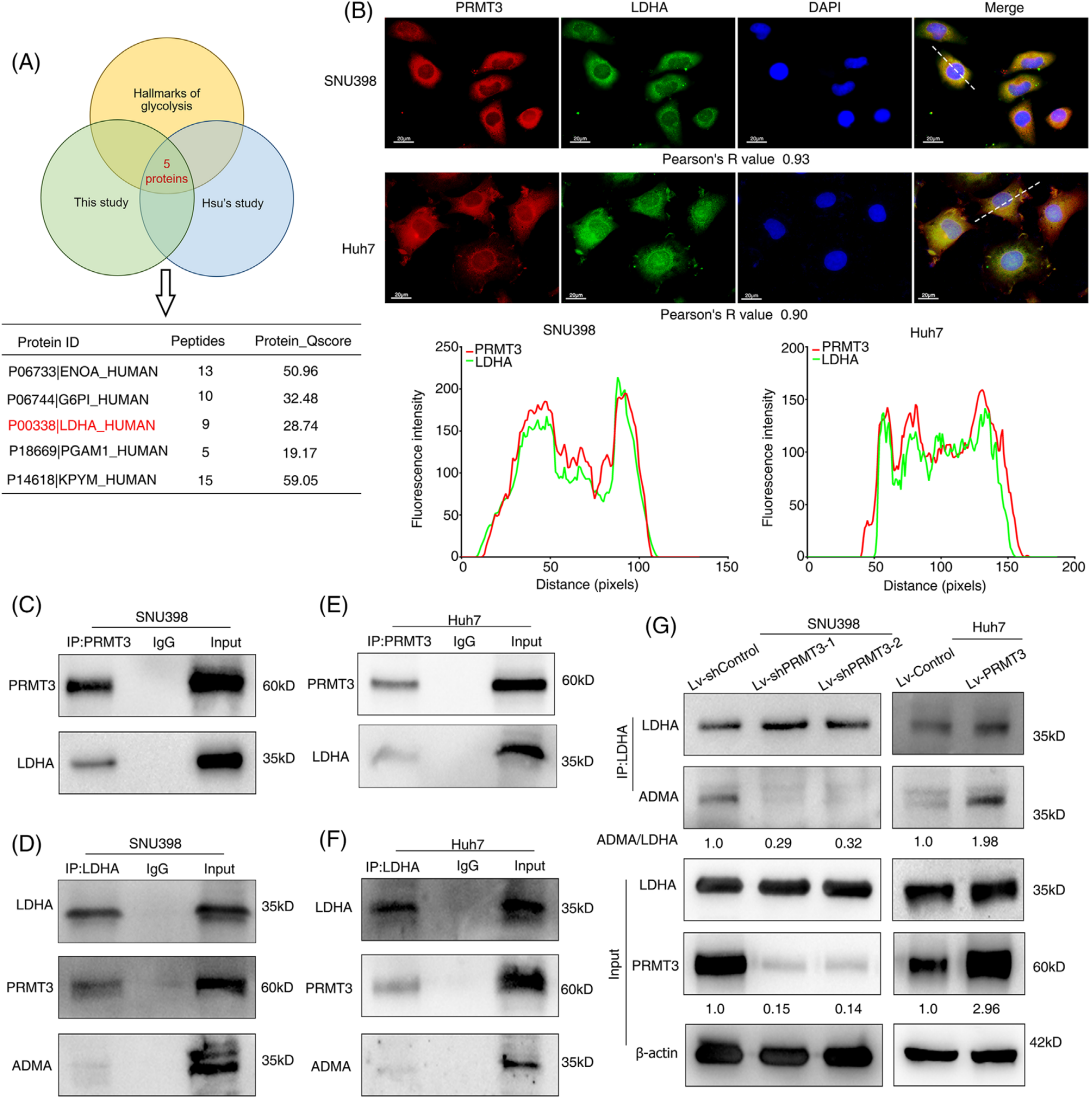
Protein arginine methyltransferase 3 (PRMT3) interacts with and promotes arginine methylation of lactate dehydrogenase A (LDHA). (A) Venn diagram showing the overlap amongst potential PRMT3‐interacting proteins identified in a previous study, in our study and proteins in database for hallmarks of glycolysis. (B) The co‐localisation status of PRMT3 and LDHA in SNU398 and Huh7 cells was reflected by immunofluorescence (IF) assay (upper panel). Pearson's correlation coefficient was used to reflect the co‐localisation of two proteins, and the intensity analysed by ImageJ software (lower panel) (scale bars: 20 μm). (C) PRMT3 proteins were immunoprecipitated from SNU398 cells and subjected to Western blotting assays to detect its interaction with LDHA. IgG was used as a negative control (*n* = 3). (D) LDHA proteins were immunoprecipitated from SNU398 cells and subjected to Western blotting assays to detect its interaction with PRMT3, as well as the signal of asymmetric dimethylarginine (ADMA) (*n* = 3). (E) PRMT3 proteins were immunoprecipitated from Huh7 cells and subjected to Western blotting assays to detect its interaction with LDHA (*n* = 3). (F) LDHA proteins were immunoprecipitated from Huh7 cells and subjected to Western blotting assays to detect its interaction with PRMT3, as well as the signal of ADMA (*n* = 3). (G) LDHA proteins were immunoprecipitated from the indicated stable hepatocellular carcinoma (HCC) cells, and subjected to Western blotting assays to detect the level of ADMA after PRMT3 knockdown or overexpression (*n* = 3). The experiments were replicated *n* times

To elucidate the mechanisms of PRMT3‐regulated glycolysis in HCC, we sought to identify its potential interacting proteins in HCC cells by IP/MS analysis (Table S3). Proteins identified by MS that may interact with PRMT3 are shown in Table S3. Consistent with above results, functional enrichment analysis also showed these proteins were enriched in ‘glycolysis or gluconeogenesis’ pathway (S11A). Five potential glycolysis proteins were identified both in previous study and our study (Figure 4A).^9^, ^27^ We conducted co‐immunoprecipitation (Co‐IP) assay to further confirm the interaction of PRMT3 with these proteins in another HCC cell line, SNU398 cells, and then focused on LDHA, a key enzyme for glycolysis (Figure S11B). Immunofluorescence (IF) analysis results showed that PRMT3 and LDHA were mainly co‐localised in the cytoplasm (Figure 4B), and the interaction between PRMT3 and LDHA was further confirmed in both SNU398 and Huh7 cells by Co‐IP assays (Figure 4C–F). Furthermore, we also detected the presence of ADMA signals of LDHA in HCC cells (Figure 4D,F). Nevertheless, because of the high efficiency of PRMT3 knockdown, we chose cells with PRMT3 knockdown as the negative control, and the results showed that in these cells, there was no bands when LDHA protein immunoprecipitates were subjected to Western blotting assays to detect the signal of PRMT3 and ADMA (Figure S12A,B). In addition, we analysed the interactions between LDHA and other type I PRMTs upregulated in HCC (responsible for catalysing ADMA), and the results only showed obvious interaction with PRMT3 (Figure S11C). Given that previous studies have shown that catalysing ADMA modification on proteins is one of the most important ways for PRMT3 to function,^8^, ^9^ we wondered whether PRMT3 was capable of enhancing the ADMA modification of LDHA. We analysed ADMA signals in cells with PRMT3 knockdown and overexpression, respectively. The results showed that downregulation of PRMT3 reduced ADMA signals of LDHA, whilst in cells with PRMT3 upregulation, the ADMA signals of LDHA was increased (Figure 4G). These results suggested that PRMT3 interacted with and promoted arginine methylation of LDHA.

### 3.6 PRMT3 catalytic activity is important for PRMT3‐mediated HCC glycolysis and growth

**FIGURE 5:**
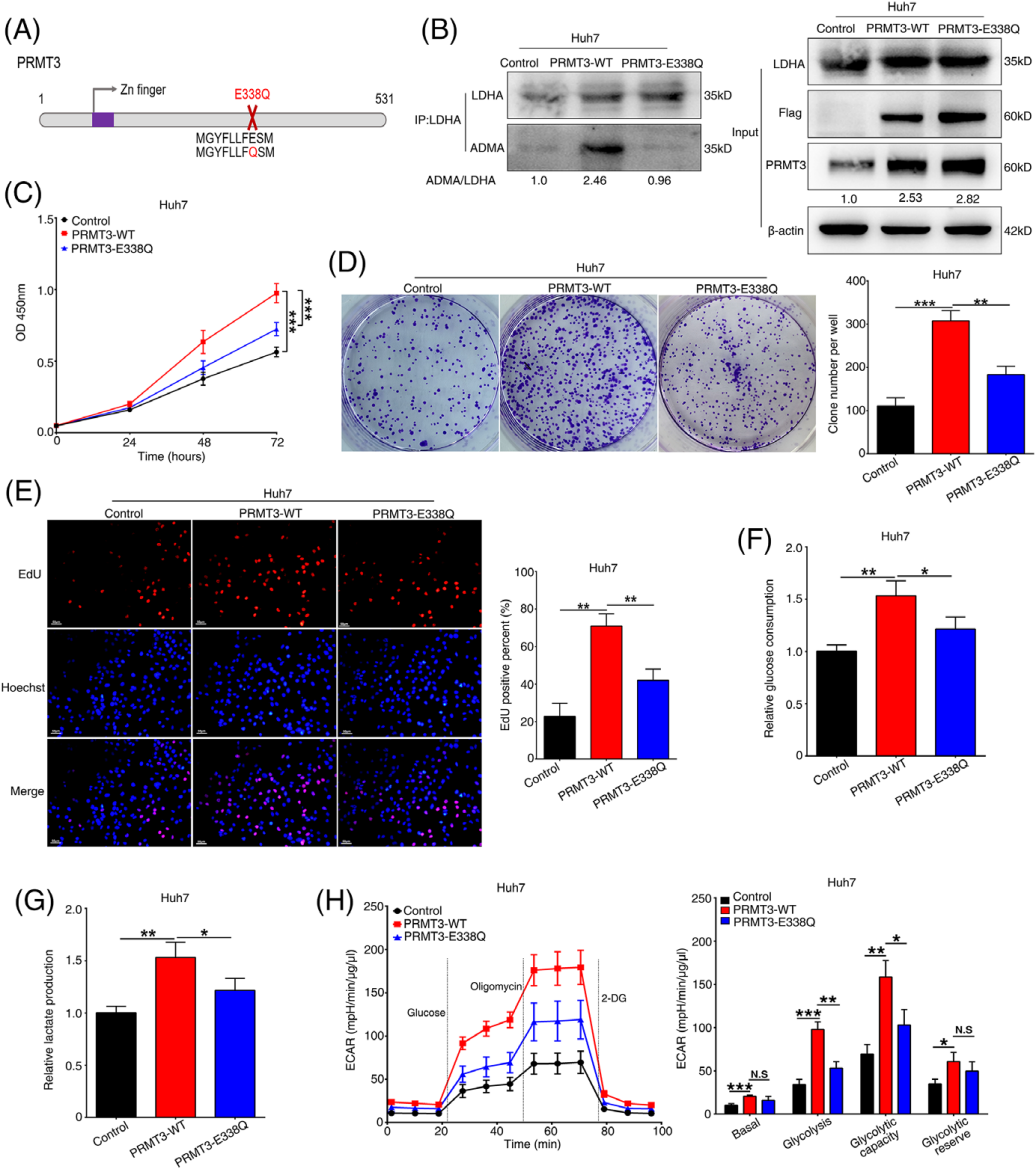
Protein arginine methyltransferase 3 (PRMT3) catalytic activity is important for PRMT3‐mediated hepatocellular carcinoma (HCC) glycolysis and growth. (A) Schematic diagram of wild‐type PRMT3 (WT) and catalytic inactive mutant PRMT3 E338Q constructs used in this study. (B) Lactate dehydrogenase A (LDHA) proteins were immunoprecipitated from the indicated HCC cells, including control HCC cells, PRMT3‐WT‐overexpressing cells and PRMT3‐E338Q‐overexpressing cells, then subjected to Western blotting assays to detect the level of asymmetric dimethylarginine (ADMA) (*n* = 3). (C) Cell counting kit‐8 (CCK‐8) assays showed the proliferation capacities of the indicated HCC cells (*n* = 3). (D) Colony formation assays were used to analyse the proliferation abilities of the indicated HCC cells (*n* = 3). (E) EdU assays were performed to detect the proliferation of the indicated HCC cells (*n* = 3, scale bars: 50 μm). (F) Relative glucose consumption of the indicated HCC cells (*n* = 3). (G) Relative lactate production of the indicated HCC cells (*n* = 3). (H) Extracellular acidification rate (ECAR) measured in the indicated HCC cells. Column statistics was shown in the right panel (*n* = 3). The experiments were replicated *n* times, and the data are represented as mean ± S.D. *p < .05, **p < .01, ***p < .001. N.S, no significance

PRMT3 downregulation and upregulation resulted in a concomitant decrease and increase of ADMA signals on LDHA, respectively, indicating the possible involvement of PRMT3 catalytic activity. To examine whether PRMT3 catalytic activity confers altered HCC glycolysis and growth, we conducted further studies using PRMT3‐wild‐type (WT) and catalytic inactive mutant PRMT3‐E338Q (Figure 5A).^28^, ^29^ We found that the catalytic activity of PRMT3 was critical for PRMT3‐mediated ADMA modification of LDHA, because overexpression of PRMT3‐WT led to a significant increase in ADMA signals of LDHA, whilst the catalytic inactive mutant of PRMT3 was incapable to confer such change in ADMA modification of LDHA (Figure 5B). Functional experiments results demonstrated that cells overexpressing catalytic inactive mutant of PRMT3 showed a partial decrease in cell proliferation and glycolysis activity, compared with those overexpressing WT PRMT3, as evidenced by weaker cell proliferation activities, less glucose consumption and lactate production, as well as less ECAR (Figure 5C–H). These findings suggested that PRMT3 catalytic activity was important for PRMT3‐mediated HCC glycolysis and growth.

### 3.7 LDHA methylation is important for PRMT3‐mediated HCC growth and glycolysis

Increasing studies have shown the importance of arginine methylation in regulation of activities of enzymes.^30^ With LDHA belonging to the lactate dehydrogenase (LDH) isoenzymes family,^31^ we observed that PRMT3 knockdown reduced LDH activity, whilst overexpression of PRMT3 led to an opposite result (Figure S13A). We next studied whether PRMT3 influenced LDHA protein stability. Our results did not support the hypothesis because (i) PRMT3 had no influence on LDHA protein expression (Figure 4G), and (ii) the stabilities of LDHA protein in cells with PRMT3 downregulation and upregulation were also similar (Figure S13B). As LDHA can form an active homotetramer protein complex, we then, investigated whether PRMT3 facilitated assembly of active tetramer. However, the results of native gel electrophoresis did not show the difference in indicated cells (Figure S13C). As the capability of LDH to bind the nucleotide cofactor reduced form of nicotinamide‐adenine dinucleotide (NADH) is essential to LDH activity,^31^ then, we sought to test the possibility that PRMT3 might promote the oxidation of NADH to nicotinamide‐adenine dinucleotide (NAD)+, and found that PRMT3 enhanced NAD+/NADH ratio (Figure S13D). These data suggested that PRMT3 might enhance LDH activity by facilitating the oxidation of NADH to NAD+.

**FIGURE 6:**
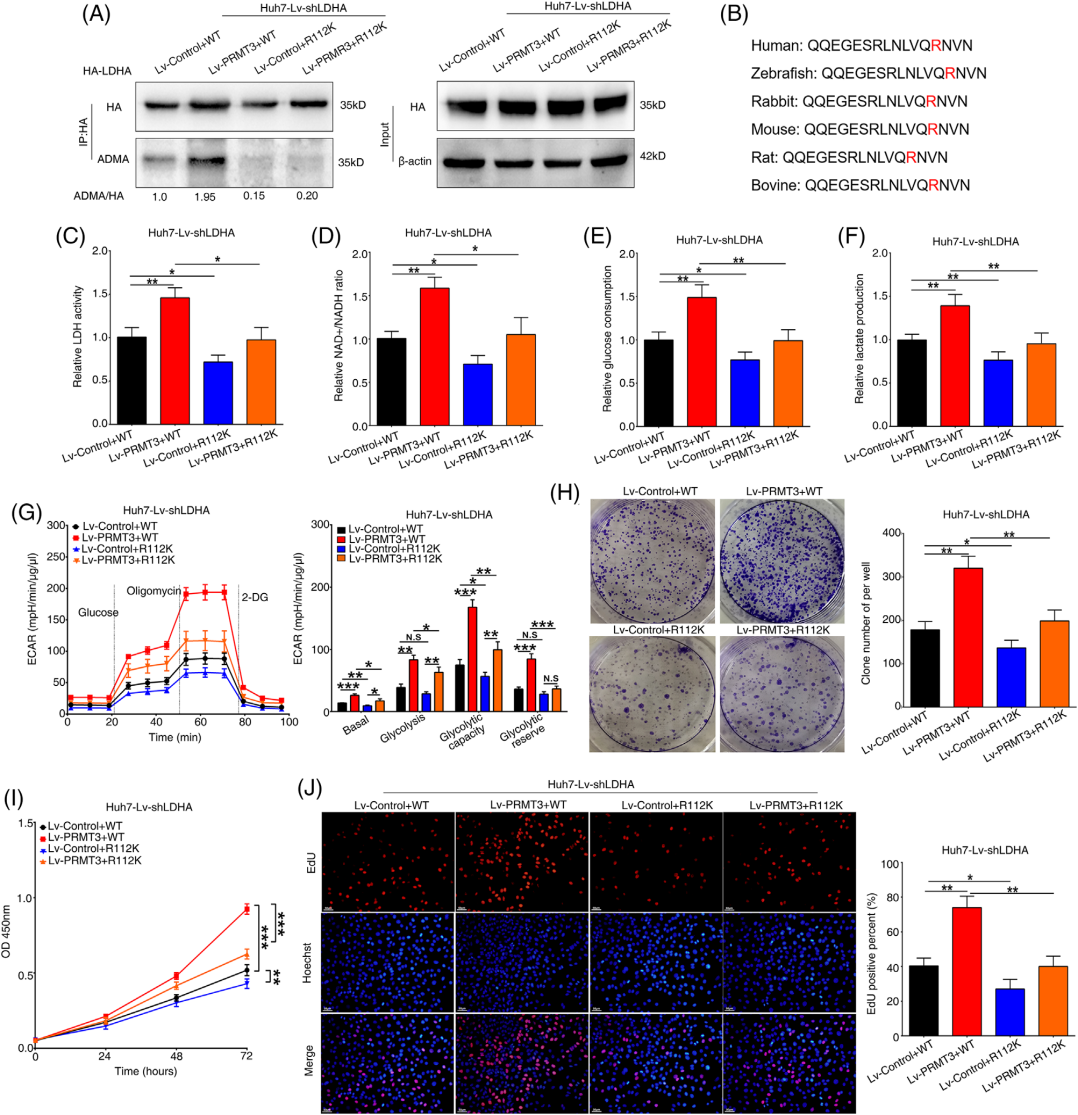
Lactate dehydrogenase A (LDHA) methylation is important for protein arginine methyltransferase 3 (PRMT3)‐mediated hepatocellular carcinoma (HCC) growth and glycolysis. (A) WT and R112K mutant LDHA protein were immunoprecipitated with HA beads in PRMT3 overexpression and control cells co‐transfected in cells with LDHA knockdown, and then subjected to Western blotting assays to detect the level of asymmetric dimethylarginine (ADMA) (*n* = 3). (B) The amino acid sequence alignment around R112 of LDHA protein amongst different species. (C) Lactate dehydrogenase (LDH) activity assays of the indicated HCC cells (*n* = 3). (D) Nicotinamide‐adenine dinucleotide (NAD)+/reduced form of NAD (NADH) assays of the indicated HCC cells (*n* = 3). (E) Relative glucose consumption of the indicated HCC cells (*n* = 3). (F) Relative lactate production of the indicated HCC cells (*n* = 3). (G) Extracellular acidification rate (ECAR) measured in the indicated HCC cells. Column statistics was shown in the right panel (*n* = 3). (H) Cell counting kit‐8 (CCK‐8) assays showed the proliferation capacities of the indicated HCC cells (*n* = 3). (I) Colony formation assays were used to analyse the proliferation abilities of the indicated HCC cells (*n* = 3). (J) EdU assays were performed to detect the proliferation of the indicated HCC cells (*n* = 3, scale bars: 50 μm). The experiments were replicated *n* times, and the data are represented as Mean ± S.D. *p < .05, **p < .01, ***p < .001. N.S, no significance

To identify potential methylation residues, we used an IP assay to enrich the LDHA protein for MS analysis. As arginine methylation modifications of great biological significance always occur on highly conserved residues in different species, we predicted six potential arginine residues (R99, R106, R112, R157, R169, R171) combining amino acid sequence analysis of LDHA across multiple species and GPS‐MSP analysis (http://msp.biocuckoo.org/) (Figure S14A).^32^, ^33^ Arginine (R)‐to‐lysine (K) mutation was always considered as a methylation‐deficient mutant. A series of R‐to‐K LDHA mutants were generated and transfected into Huh7 cells, followed by ADMA signal analysis (Figure S14B). The result showed that ADMA methylation of LDHA was significantly abolished in cells transfected with R112K mutants but was still clearly observed in cells with other mutants (Figures S14C and S15A,B). Meanwhile, we also identified two potential arginine residues in our MS result, R106 and R112 (Figure S16A). To further confirm MS results, we expressed R106K and R112K mutants in Huh7 cells, and observed the significant decrease in ADMA signals of LDHA in cells with R112K and R106K+R112K LDHA mutant by ADMA status analysis, whilst R106K mutant did not affect ADMA signals (Figure S16B). Furthermore, we also established stable cell line Huh7‐Lv‐shLDHA for further investigation (Figure S17A). In cells with LDHA knockdown, ADMA signals were hardly detected when R112K mutant was transfected into both PRMT3‐overexpressing and control cells (Figure 6A). Sequence alignment demonstrated the highly conservation of this arginine residue across species (Figure 6B), implying methylation of this residue may be biologically significant.

More importantly, the results of LDH activity assay showed that mutation of R112 markedly reduced the PRMT3‐faciliated increase in LDH activity and NAD+/NADH ratio in the indicated cells (Figure 6C,D), whilst the results of LDHA protein stability and native gel electrophoresis analysis showed no differences in indicated cells (Figure S17B,C). These findings demonstrated that PRMT3 methylated LDHA at R112 to increase LDH activity.

Next, we sought to investigate the biological function of PRMT3‐mediated LDHA methylation. The results of glucose and lactate analyses showed that the mutation of R112 significantly reduced both the PRMT3‐faciliated increase in glucose consumption and lactate production in PRMT3‐overexpressing cells. Intriguingly, compared with control cells transfected with WT vectors, those with R112K mutant also showed a slight decrease in glucose consumption and lactate production, which may be caused by endogenous PRMT3 (Figure 6E,F). To further support the results, we measured the ECAR of indicated HCC cells and observed a similar effect (Figure 6G). The CCK‐8, colony formation and EdU staining assays results indicated that R112 mutation attenuated HCC proliferation in the indicated cells (Figure 6H–J). These data suggested that LDHA methylation was involved in PRMT3‐mediated HCC growth and glycolysis.

### 3.8 PRMT3 inhibitor SGC707 abolishes HCC growth and glycolysis

We have identified the potential role of PRMT3 in promoting HCC growth, and therefore we explored further to determine whether pharmacological inhibition of PRMT3 could reverse the effect. SGC707, a potent, selective inhibitor of PRMT3, was utilised to observe the therapeutic effect of PRMT3 pharmacological inhibition in PRMT3‐overexpressing carcinomas. As shown in Figure S18A, ADMA signals of LDHA were remarkedly reduced with treatment with SGC707 at the dose of 1 μM. Higher concentration of SGC707 resulted in more significant reduction of ADMA signals of LDHA. Since a multitude of targets could be affected at high concentration of SGC707,^29^ to demonstrate specific effects, we therefore selected the dose of 1 μM for functional analysis.

**FIGURE 7:**
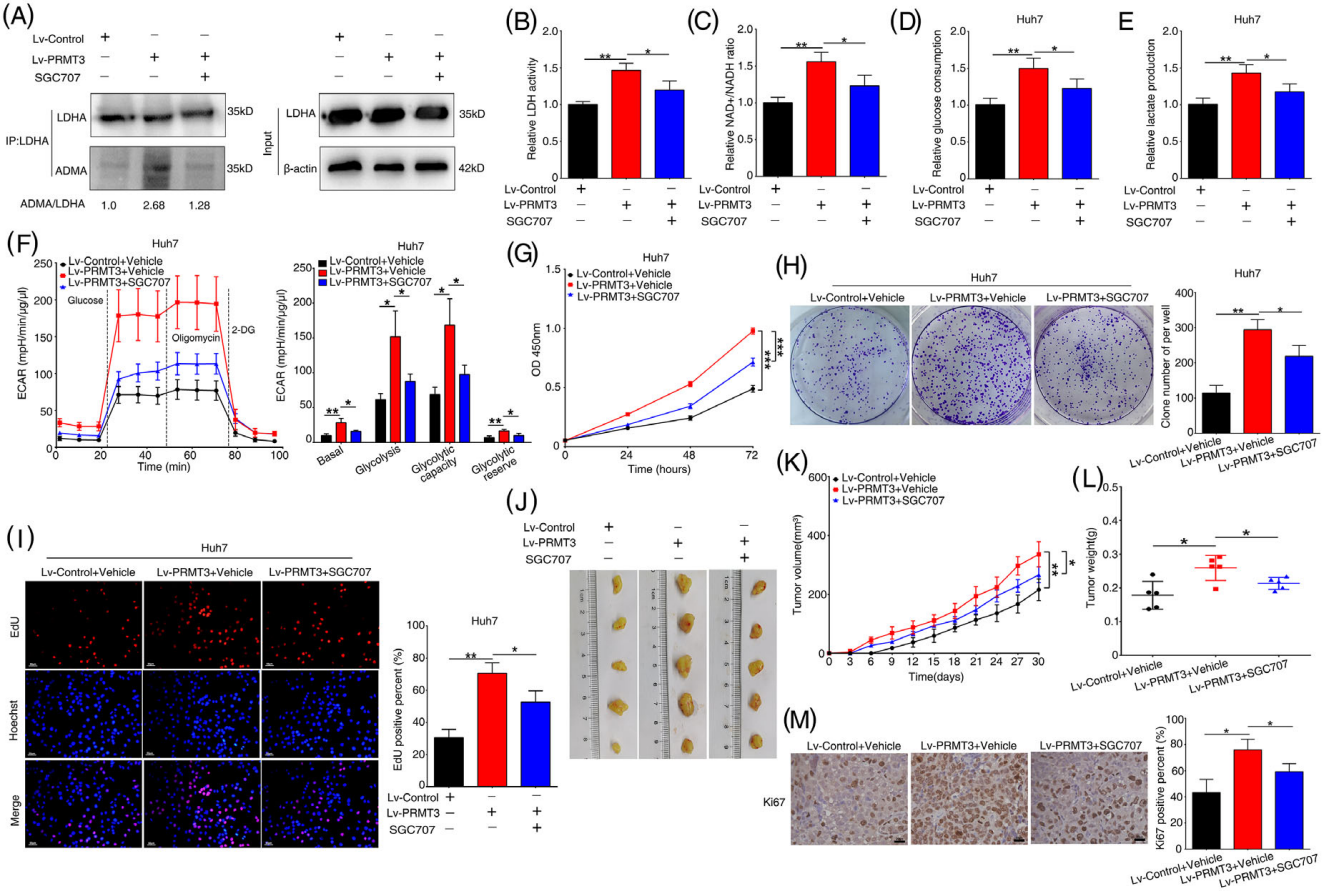
Protein arginine methyltransferase 3 (PRMT3) inhibitor SGC707 abolishes hepatocellular carcinoma (HCC) growth and glycolysis. (A) Lactate dehydrogenase A (LDHA) proteins were immunoprecipitated from the indicated HCC cells, including control HCC cells with DMSO treatment for 48 h, PRMT3‐overexpressing cells with dimethyl sulfoxide (DMSO) treatment for 48 h and PRMT3‐overexpressing cells with SGC707 (1 μM) treatment for 48 h, then subjected to Western blotting assays to detect the level of asymmetric dimethylarginine (ADMA) (*n* = 3). (B) Lactate dehydrogenase (LDH) activity assays of the indicated HCC cells (*n* = 3). (C) Nicotinamide‐adenine dinucleotide (NAD)+/reduced form of NAD (NADH) assays of the indicated HCC cells (*n* = 3). (D) Relative glucose consumption of the indicated HCC cells (*n* = 3). (E) Relative lactate production of the indicated HCC cells (*n* = 3). (F) Extracellular acidification rate (ECAR) measured in the indicated HCC cells. Column statistics was shown in the right panel (*n* = 3). (G) Cell counting kit‐8 (CCK‐8) assays showed the proliferation capacities of the indicated HCC cells (*n* = 3). (H) Colony formation assays were used to analyse the proliferation abilities of the indicated HCC cells (*n* = 3). (I) EdU assays were performed to detect the proliferation of the indicated HCC cells (*n* = 3, scale bars: 50 μm). (J–L) The subcutaneous HCC xenograft model was established and xenograft nude mice were intraperitoneally administrated with SGC707 or vehicle every 2 days at a dose of 30 mg/kg from the day after subcutaneous injection. (J) Images of subcutaneous tumours (*n* = 5 for each group). (K) Tumour growth curves of each group. (L) Tumour weight of the tumours. (M) Representative Ki‐67 staining images showed the proliferation of xenografts in the indicated groups (scale bars: 20 μm). The experiments were replicated *n* times, and the data are represented as mean ± S.D. *p < .05, **p < .01, ***p < .001

PRMT3 was found to upregulate the ADMA methylation of LDHA, and, the effect was reversed by SGC707 treatment (Figure 7A). Meanwhile, the LDH activity and NAD+/NADH ratio increased with PRMT3 overexpression were also partially attenuated by SGC707 treatment (Figure 7B,C). Next, we investigated whether SGC707 treatment had an effect on the enhancement of glycolysis in PRMT3‐overexpressing cells. The results demonstrated that the addition of SGC707 at a dose of 1 μM blocked the elevation of glycolysis induced by PRMT3 overexpression (Figure 7D–F). Moreover, compared with the addition of vehicle, those with SGC707 treatment possessed a weakened proliferation capacity, especially in cells with PRMT3 upregulation, which indicated the role of SGC707 in PRMT3‐mediated proliferation (Figures 7G–I and S19B–D). Notably, control cells with SGC707 treatment also showed a slight but not significant decrease in cell proliferation capacity, which might be caused by endogenous PRMT3 (Figure S19B–D). In contrast, the enhanced ADMA signals and proliferation abilities of the indicated cells were not remarkably altered when we treated the indicated cells with XY‐1, a close analogue of SGC707, completely inactive against PRMT3 at concentration up to 100 μM (Figure S19A–D).

Oligomycin is an inhibitor of F0/F1 adenosine triphosphate (ATP) synthase and mitochondrial respiration, which has been reported to suppress tumour growth.^34^ Since SGC707 elicited an effect on suppressing PRMT3‐enhanced HCC glycolysis and growth, we wondered whether combination SGC707 with oligomycin could exhibit a more significant growth‐suppressive effect. Through our preliminary study, we observed that combination SGC707 with oligomycin treatment in PRMT3‐overexpressing cells resulted in a more significant impact on cell proliferation inhibition (Figure S20A–C). Nevertheless, additional studies are required to validate these findings as well as to explore the underlying mechanisms.

In order to explore whether SGC707 could suppress PRMT3‐mediated HCC growth in vivo, xenograft nude mice were intraperitoneally administrated with SGC707 or vehicle every 2 days with the dose of 30 mg/kg from the day after subcutaneous injection onward. In in vivo tumour growth assays, the increase in both tumour volume and weight were detected in the group of mice injected with PRMT3‐overexpressing cells, whilst SGC707 treatment reversed this effect (Figure 7J–L). Additionally, Ki‐67 staining of the xenografts showed the same results, further supporting above findings (Figure 7M). These results indicated that PRMT3 inhibitor SGC707 might be a promising approach to suppress HCC growth.

## 4 DISCUSSION

HCC is one of the most prevalent cancers worldwide with a high mortality.^35^ Though adjuvant chemotherapy and molecular targeted therapy are now available for patients with advanced HCC, their efficacy is limited to a small proportion of patients. Thus, novel targets for HCC therapy need to be developed in order to improve the prognosis of patients with HCC. Here, we reported a novel role of PRMT3, a member of PRMTs, in HCC. Over the past decades, the role of PRMT family members in HCC is being gradually discovered. For example, PRMT2 has been reported to accelerate HCC tumourigenesis by activating BCL2, whilst PRMT6 has been considered as a tumour suppressor of HCC.^36^, ^37^ In the present study, we first demonstrated that PRMT3 was upregulated in HCC tissues, overexpression of which was related with poor prognosis of HCC patients, as well as malignant clinicopathological characteristics. These observations strongly suggested that PRMT3 might be a functional driver of HCC. Intriguingly, our data showed that PRMT3 promoted HCC growth in vitro and in vivo, which supports this hypothesis.

Proliferating tumour cells often exhibit aberrant glucose metabolism featured with elevated glucose uptake and lactate production, as well as a high rate of glycolysis. Increased metabolic flux through glycolysis in turn provides biosynthetic precursors for the rapid synthesis of macromolecules and cellular redox homeostasis maintenance to facilitate cell growth.^38^ Glycolysis and tumour cell growth are mutually dependent in some instances. In the present study, we found that PRMT3 could enhance glycolysis to promote HCC growth, which provided evidence for the significance of glycolysis in tumour growth.

Post‐translational modification is of great significance in regulating the structures, functions and activities of many metabolic enzymes, thereby serving as a vital mechanism for cellular metabolism regulation.^39^, ^40^ Though arginine methylation is a type of post‐translational modifications that regulates multiple cellular biological processes, studies on the association between arginine methylation and metabolism still remain in the beginning stage to date. For instance, PRMT6 was reported to methylate sirtuin 7 at R388, thus coordinating glucose availability with mitochondria biogenesis to maintain energy homeostasis.^41^ In HCC, CRAF methylation by PRMT6 had an effect on modulating aerobic glycolysis‐driven tumourigenesis in HCC via PKM2 nuclear localisation and activation.^22^ In fact, three studies have reported that GAPDH can be methylated by PRMT1, PRMT4 and PRMT3. Cho et al.^42^ showed that PRMT1 was able to mediate arginine methylation of GAPDH in primary bone marrow‐derived macrophages, but they did not mention the specific metabolic processes or methylation sites. Another study reported that GAPDH was methylated by PRMT4 at R234 with its catalytic activity inhibited, and the glycolysis was suppressed in HCC cells.^23^ During the preparation of this study, the impact of PRMT3 on metabolic reprogramming in pancreatic cancer was revealed. Hsu Et al. demonstrated that PRMT3 promoted both glycolysis and mitochondrial respiration by methylating GAPDH. These findings have indicated that PRMT3 might play a role in mediating modifications of metabolic enzymes. Consistent with previous studies, in this study, we found that PRMT3 promoted HCC growth by enhancing glycolysis via arginine methylation of LDHA, thus increasing its activity. It is worth mentioning that, in the negative control model, there was no obvious bands or ADMA signals when LDHA protein immunoprecipitates subjected to Western blotting assays. However, in SNU398 and Huh7 cells, we observed the interaction between PRMT3 and LDHA proteins via IF and Co‐IP assays. This further confirms the link between these two proteins, as well as arginine methylation and metabolism regulation. However, it is expected that further investigation will reveal a broader role of arginine methylation in regulating cell metabolism.

LDHA is one of the key enzymes of glycolysis and responsible for catalysing the conversion of pyruvate to lactate. In cancers, LDHA has been identified as an oncogene that can help tumour cells produce large amounts of energy rapidly through glycolysis to support cell growth.^43^ Actually, LDHA has been reported to possess different post‐translational modification in previous studies, including acetylation and phosphorylation. LDHA can be activated by upstream kinases via phosphorylation at tyrosine 10 (Y10), thus promoting tumour invasion and metastasis.^44^ Recently, high succinylation of LDHA at K222, which inhibits LDHA degradation, has been observed and reported to be associated with poor prognosis in gastric cancer patients.^45^ However, in another study, LDH activity was shown to be inhibited by LDHA K5 acetylation, which decreased proliferation and migration in cancer cells.^46^ In our study, we first reported that LDHA could be methylated at R112, which was crucial for PRMT3‐induced glycolysis and HCC growth. R112 is located at the nucleotide‐binding Rossmann fold and near the catalytic loop, which plays a key role in committing LDH to catalysis.^31^ It is possible that methylation at this residue might influence LDH activity. This hypothesis is supported by our findings that R112 mutation reduced LDH activity and NAD+/NADH ratio, with the latter important for LDH activity. It is likely that, even on the same protein, different types of post‐translational modifications exhibit different effects, which ensures that cells can effectively coordinate metabolic regulation to maximise the ability to survive.

Instead of the irreversibility of genetic mutations, reversibility of epigenetic modifications provides more opportunities for cancer therapy.^47^ Current drugs for HCC therapy produced unsatisfactory results. Importantly, increasing number of PRMT inhibitors are undergoing clinical trials, further demonstrating the potential of PRMT inhibitors in clinical applications. For example, GSK3368715, a PRMT1 inhibitor has been utilised in patients with B‐cell lymphoma, whilst several antagonists targeting PRMT5 have been investigated in many clinical trials for solid tumours and non‐Hodgkin lymphoma.^30^ Motivated by these results, we also presented data regarding the effectiveness of the PRMT3 inhibitor, SGC707, in suppressing PRMT3‐induced HCC glycolysis and tumour growth. Previous studies have shown that SGC707 can alleviate hepatic steatosis in mice and inhibit the osteogenesis of human mesenchymal stem cells.^10^, ^11^, ^29^ Since no specific PRMT3 inhibitor is available for clinical treatment, our work revealed a novel point for HCC intervention targeting PRMT3. Furthermore, as a proof‐of‐concept, our work has also shown the effectiveness of combining PRMT3 inhibitor and oligomycin in suppressing HCC growth in a subset of HCC patients with high PRMT3 expression. However, a recent study has reported the potential relevance of arginine methylation and platelet, suggesting that the PRMT inhibitors may show adverse effects correlated with impaired platelet function, which should be considered when they are in pre‐clinical and clinical trials as emerging anticancer drugs.^48^

Several new issues arise from these findings, which require further investigations. For example, more work is required to clarify other functions and potential downstream pathways regulated by PRMT3, and, importantly, how PRMT3 mRNA and protein level is enhanced in HCC. Further studies are needed to explore the effects of combining SGC707 with other cancer treatment drugs, including PRMTs inhibitors.

## 5 CONCLUSIONS

**FIGURE 8:**
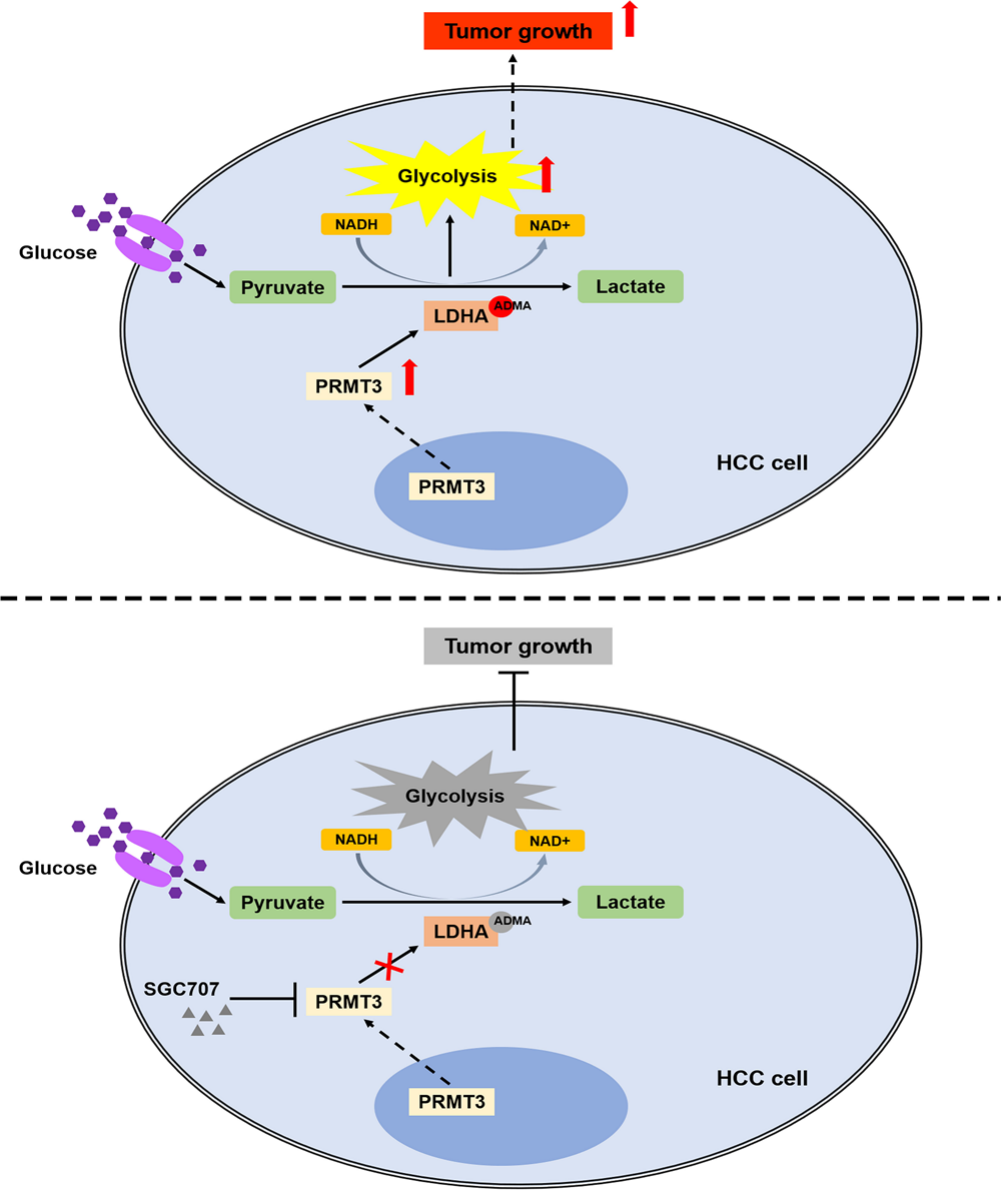
A schematic diagram of the role of protein arginine methyltransferase 3 (PRMT3) in enhancing lactate dehydrogenase A (LDHA) arginine methylation modification to promote glycolysis‐driven hepatocellular carcinoma (HCC) growth. Overexpression of PRMT3 enhanced the glycolysis in HCC cells via mediating arginine methylation of LDHA to increase its activity, thus promoting cell proliferation and tumour growth. The administration of PRMT3 inhibitor SGC707 effectively disrupted the increased asymmetric dimethylarginine (ADMA) modification mediated by PRMT3 attenuated PRMT3‐induced HCC glycolysis and tumour growth

In conclusion, the present study has demonstrated a novel oncogenic function of PRMT3 in HCC. Overexpression of PRMT3 enhanced the glycolysis in HCC cells, thus promoting cell proliferation and tumour growth. Mechanistically, we have identified that PRMT3 facilitated LDHA methylation at R112 to increase its activity, and we reported the significance of LDHA methylation in HCC glycolysis and growth. Moreover, the administration of PRMT3 inhibitor SGC707 effectively disrupted the increased ADMA modification mediated by PRMT3 and attenuated PRMT3‐induced HCC glycolysis and tumour growth (Figure 8). Our study provides a new underlying molecular insight associated with metabolism dysregulation induced by arginine methylation modification in the context of HCC, and provides evidence for developing a novel potential target for HCC therapy.

## CONFLICT OF INTEREST

The authors declare no conflicts of interest.

## Supporting information

Figure S1

Figure S2

Figure S3

Figure S4

Figure S5

Figure S6

Figure S7

Figure S8

Figure S9

Figure S10

Figure S11

Figure S12

Figure S13

Figure S14

Figure S15

Figure S16

Figure S17

Figure S18

Figure S19

Figure S20

Supporting Information

Table S1

Table S2

Table S3

Table S4

Table S5
