# Supplementary figures and images for: Protein arginine methyltransferase 3 promotes glycolysis and hepatocellular carcinoma growth by enhancing arginine methylation of lactate dehydrogenase A
Source: Clin Transl Med. 2022 Jan 28;12(1):e686. doi: 10.1002/ctm2.686 (PMC8797063; doi:10.1002/ctm2.686)

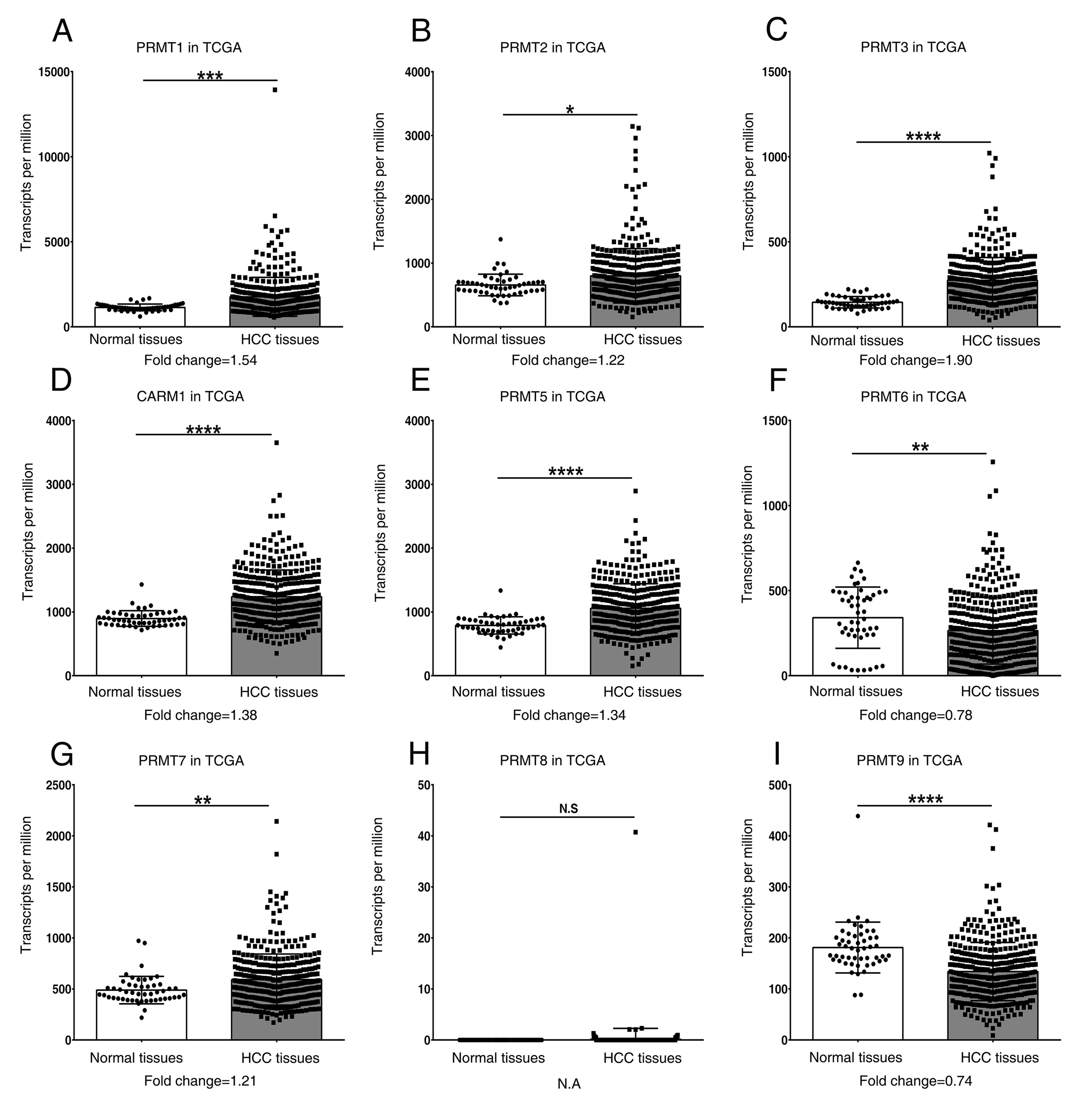

Supplement: Supplementary file 1 — Figure S1 [file CTM2-12-e686-s023.jpg]

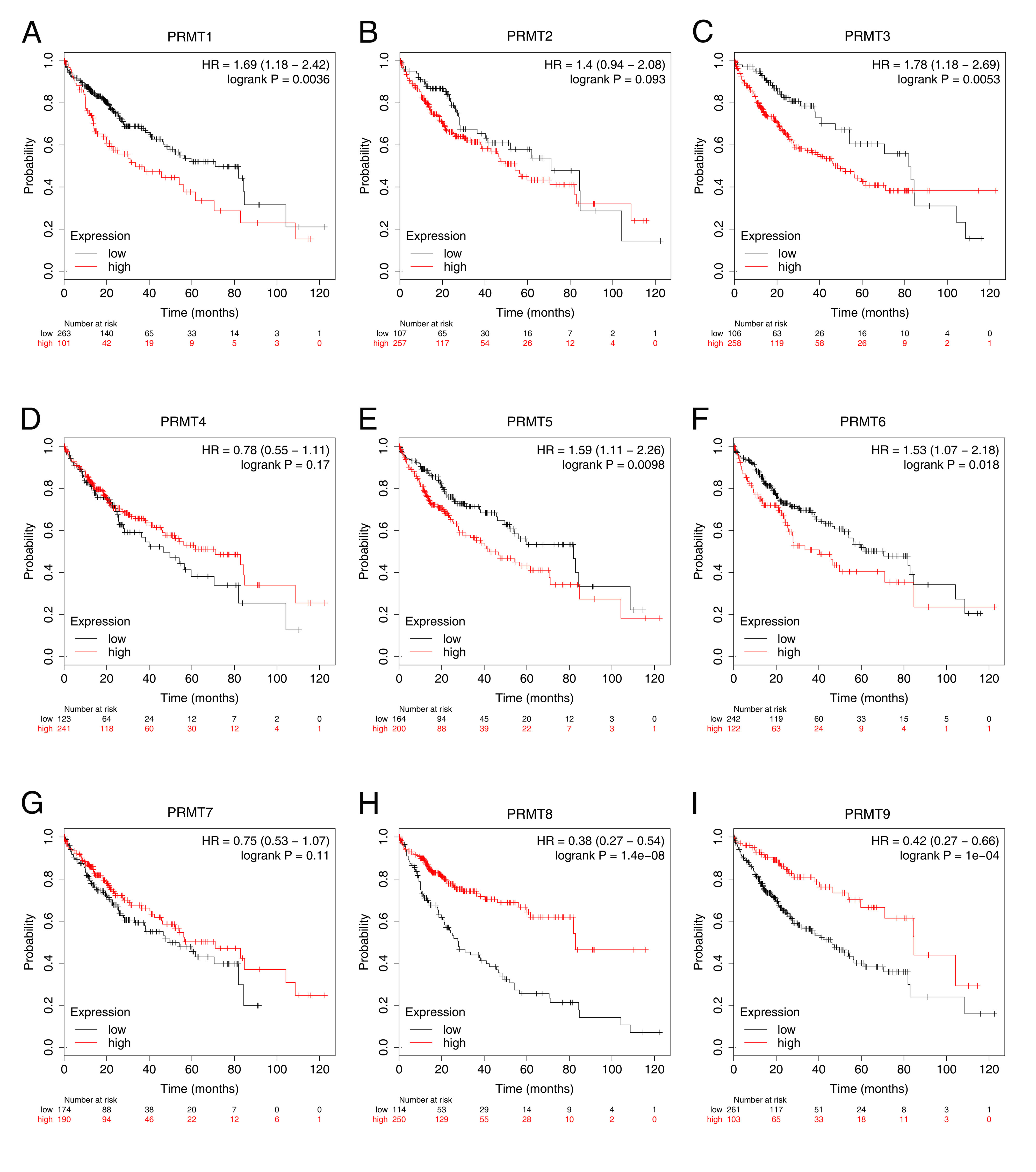

Supplement: Supplementary file 2 — Figure S2 [file CTM2-12-e686-s024.jpg]

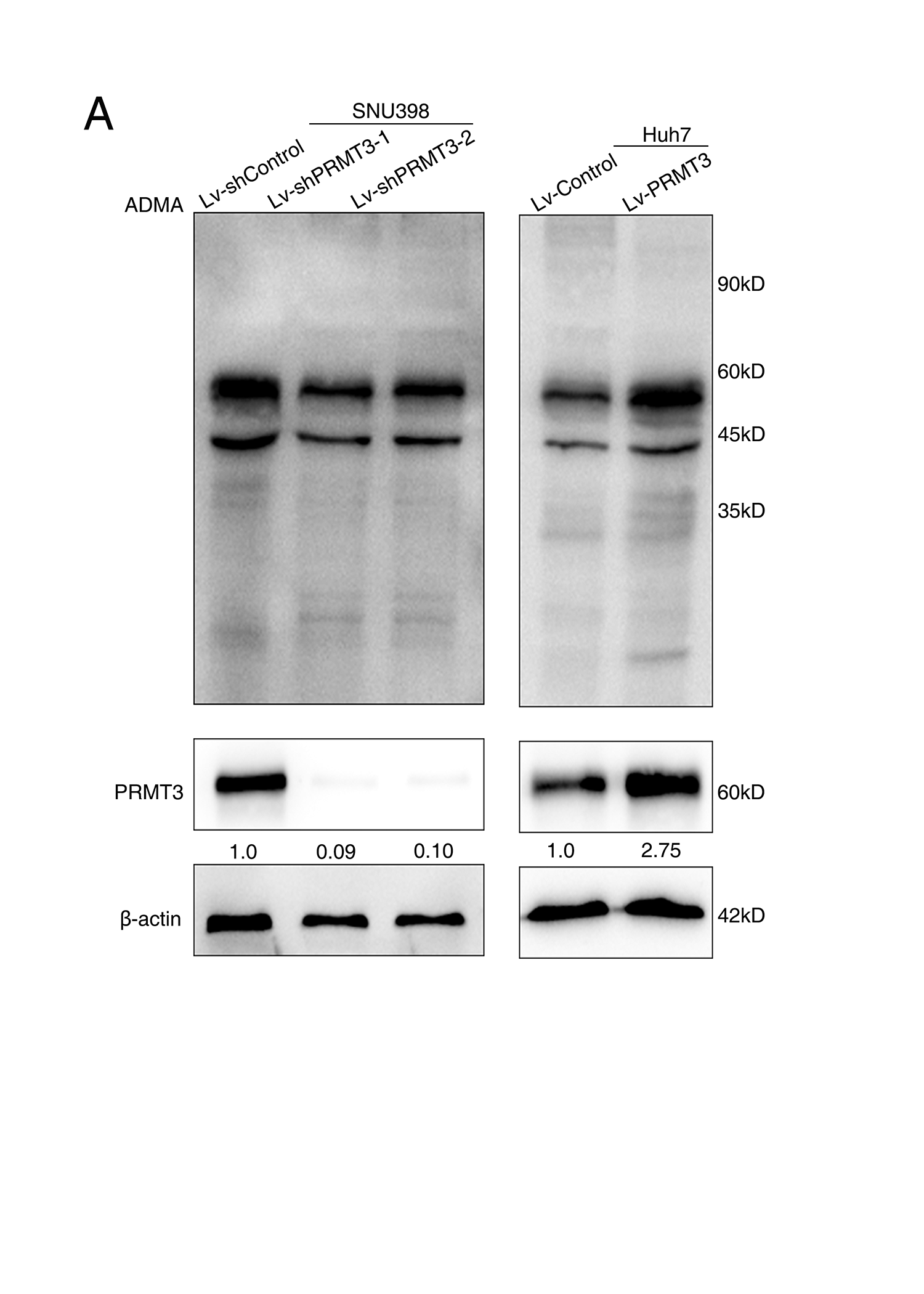

Supplement: Supplementary file 3 — Figure S3 [file CTM2-12-e686-s009.tif]

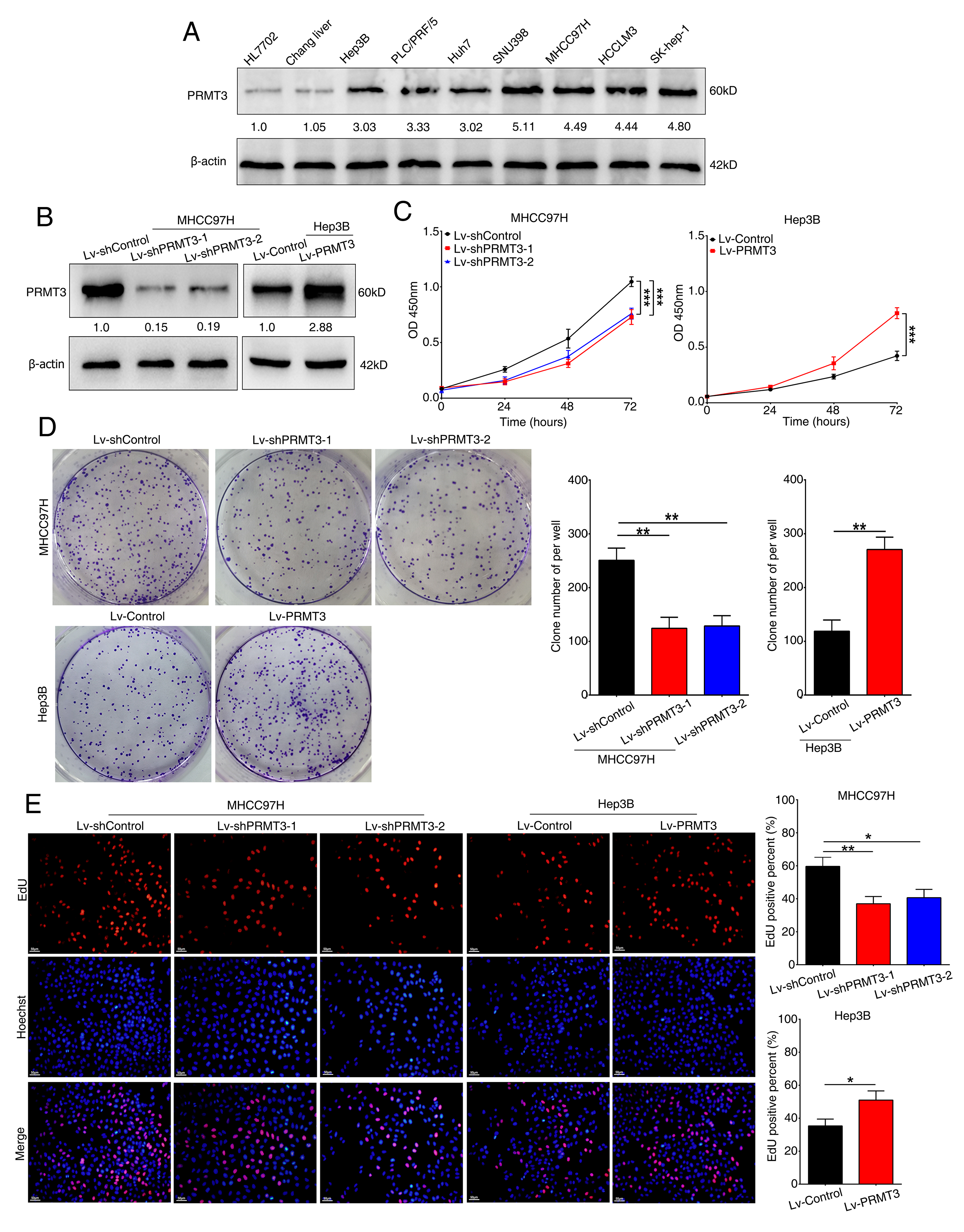

Supplement: Supplementary file 4 — Figure S4 [file CTM2-12-e686-s002.tif]

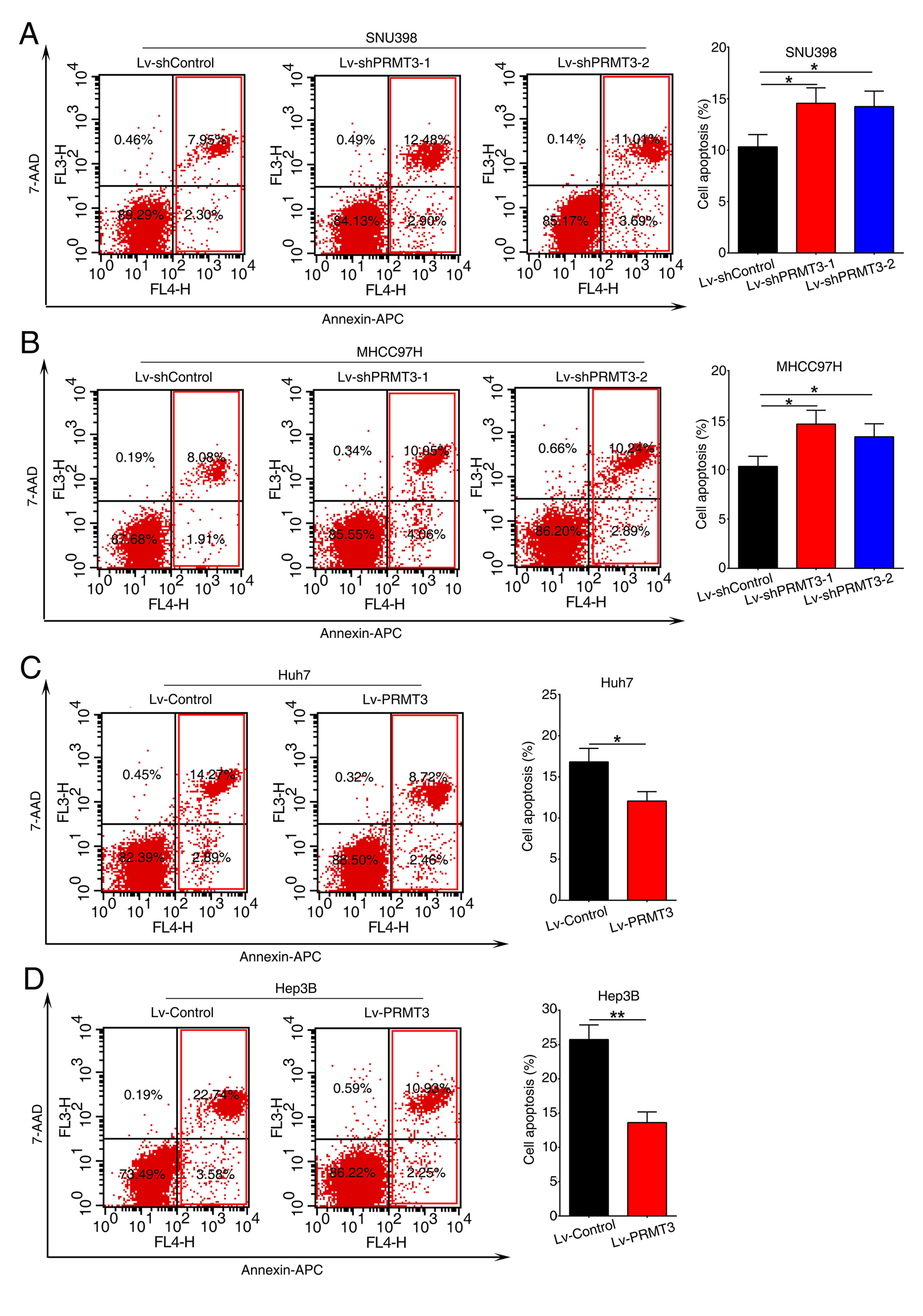

Supplement: Supplementary file 5 — Figure S5 [file CTM2-12-e686-s021.jpg]

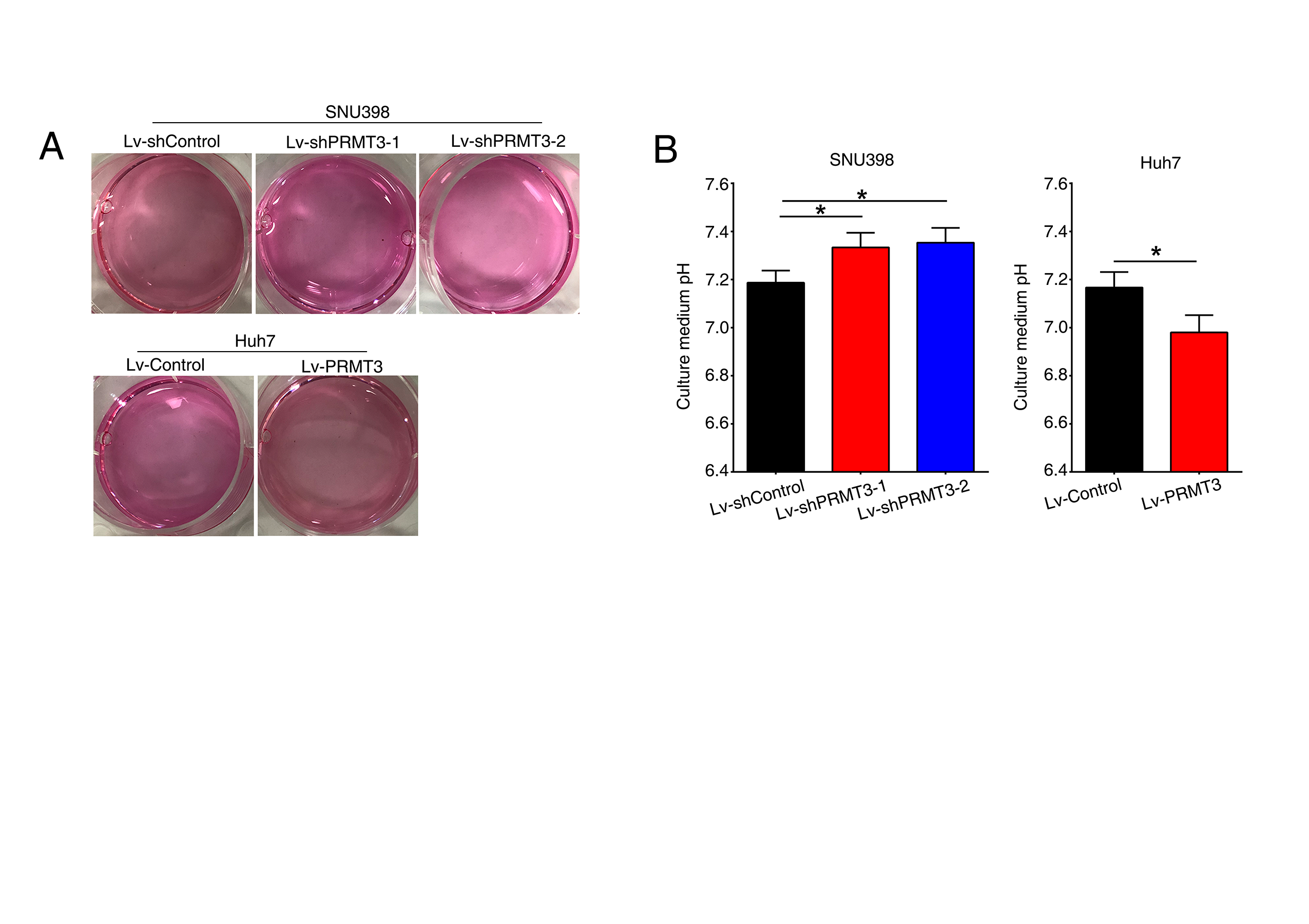

Supplement: Supplementary file 6 — Figure S6 [file CTM2-12-e686-s015.tif]

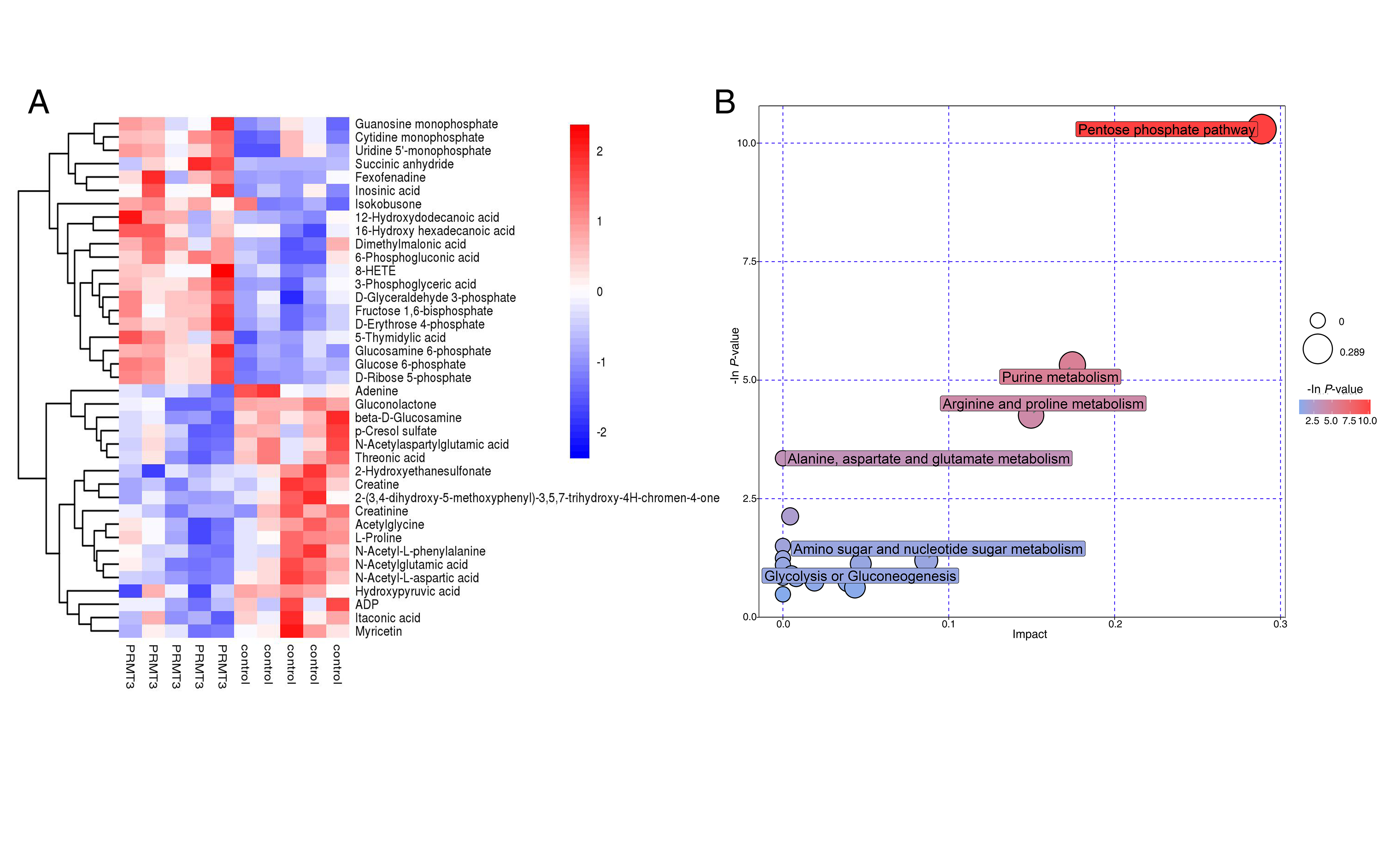

Supplement: Supplementary file 7 — Figure S7 [file CTM2-12-e686-s004.tif]

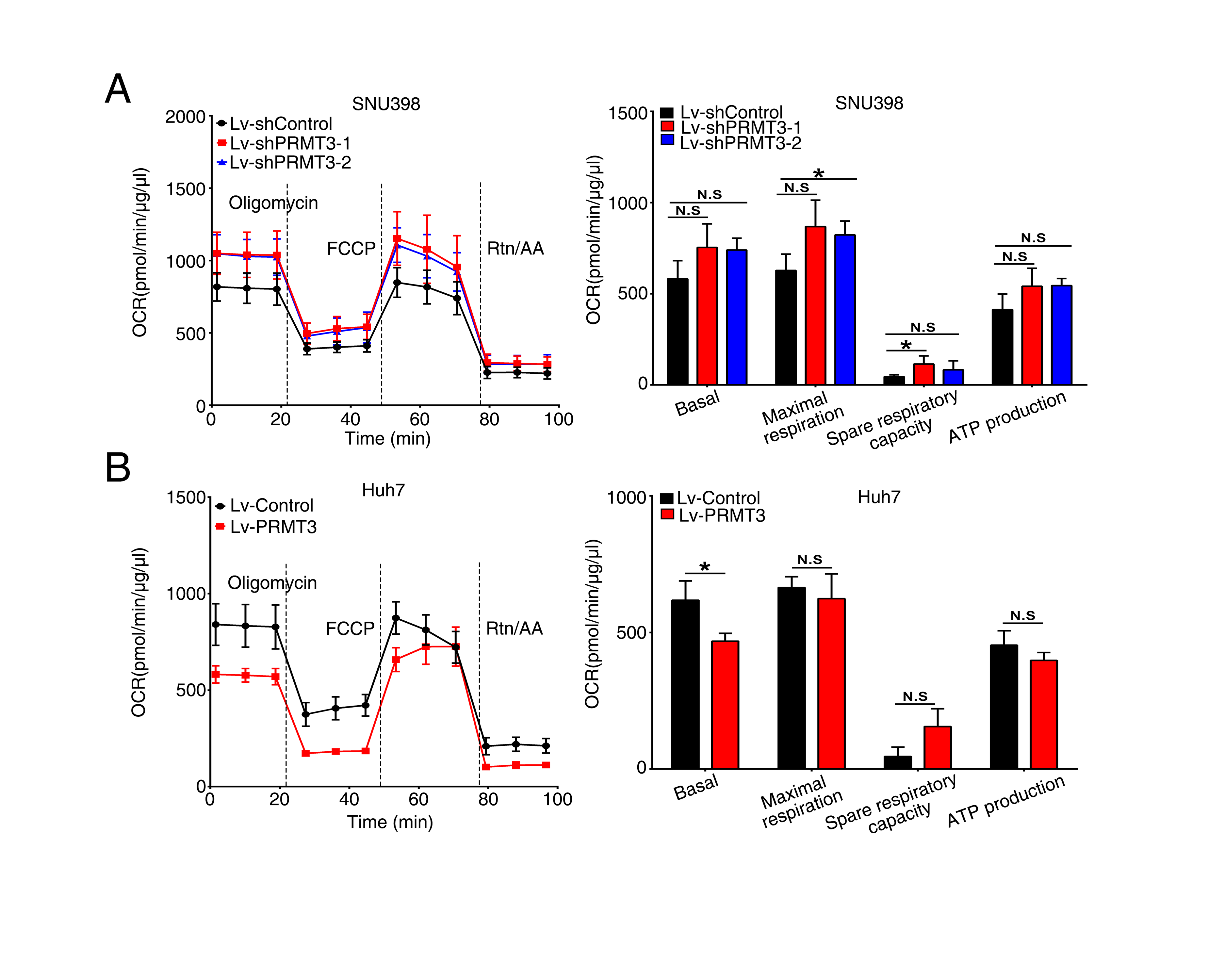

Supplement: Supplementary file 8 — Figure S8 [file CTM2-12-e686-s013.tif]

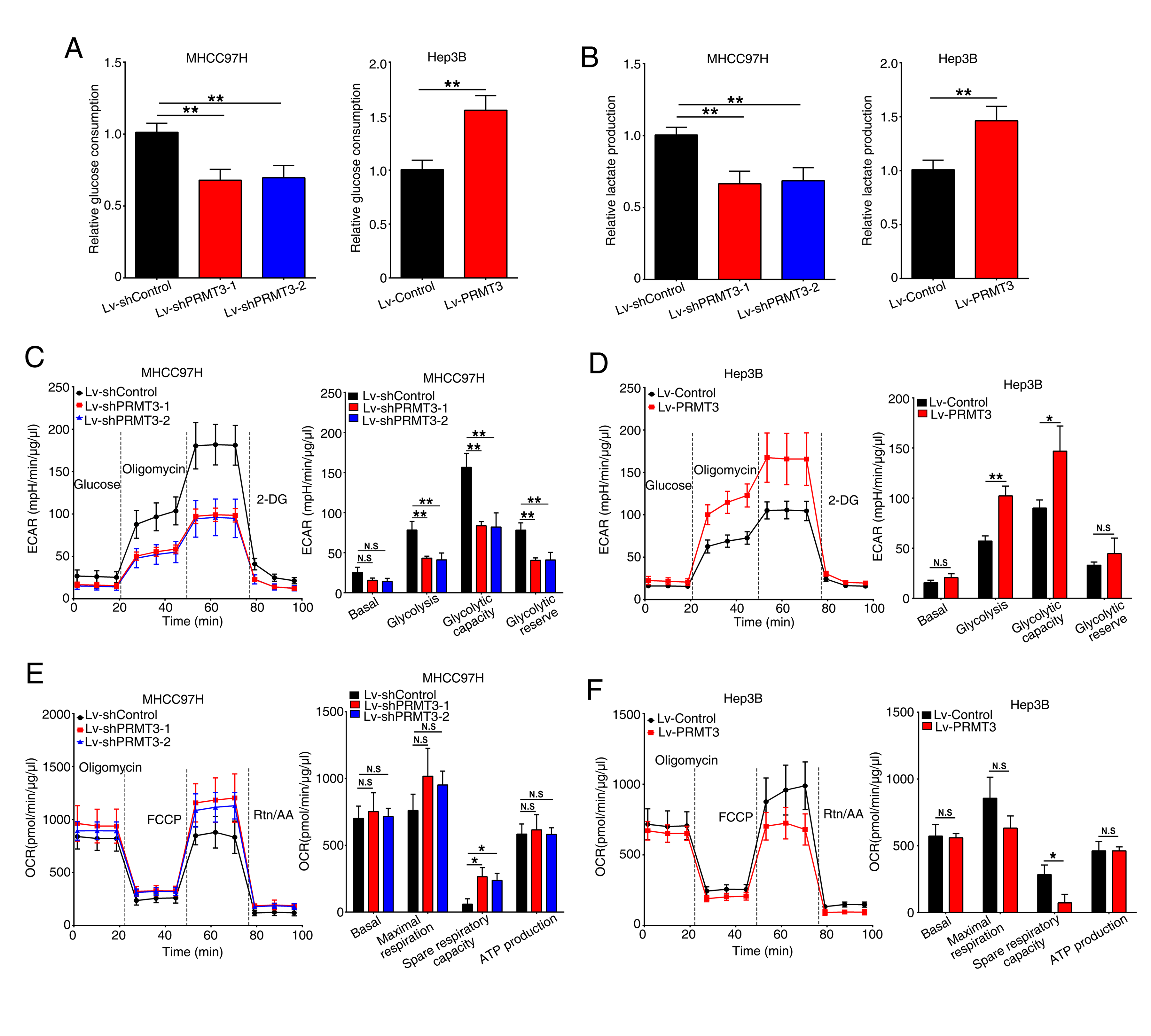

Supplement: Supplementary file 9 — Figure S9 [file CTM2-12-e686-s018.tif]

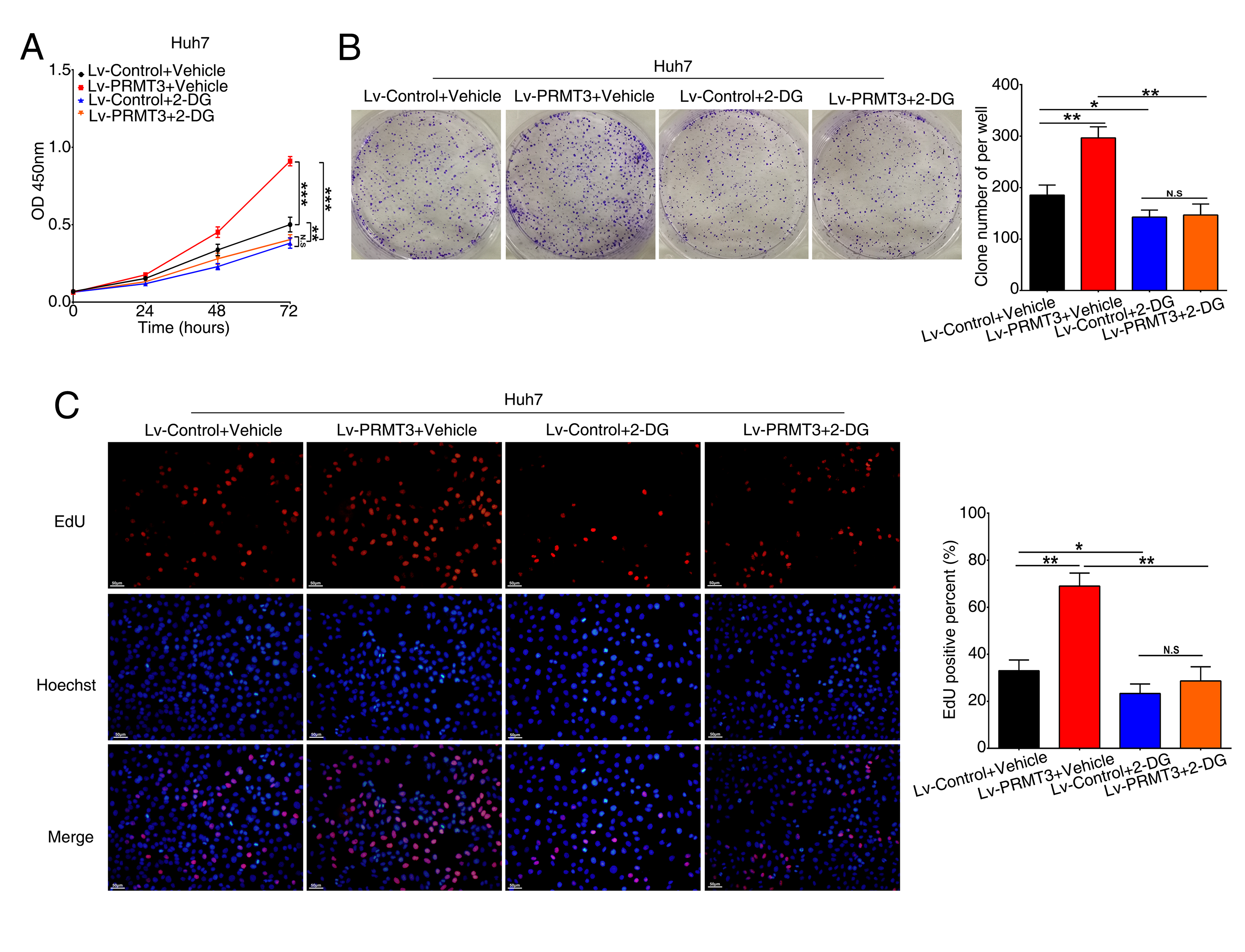

Supplement: Supplementary file 10 — Figure S10 [file CTM2-12-e686-s017.tif]

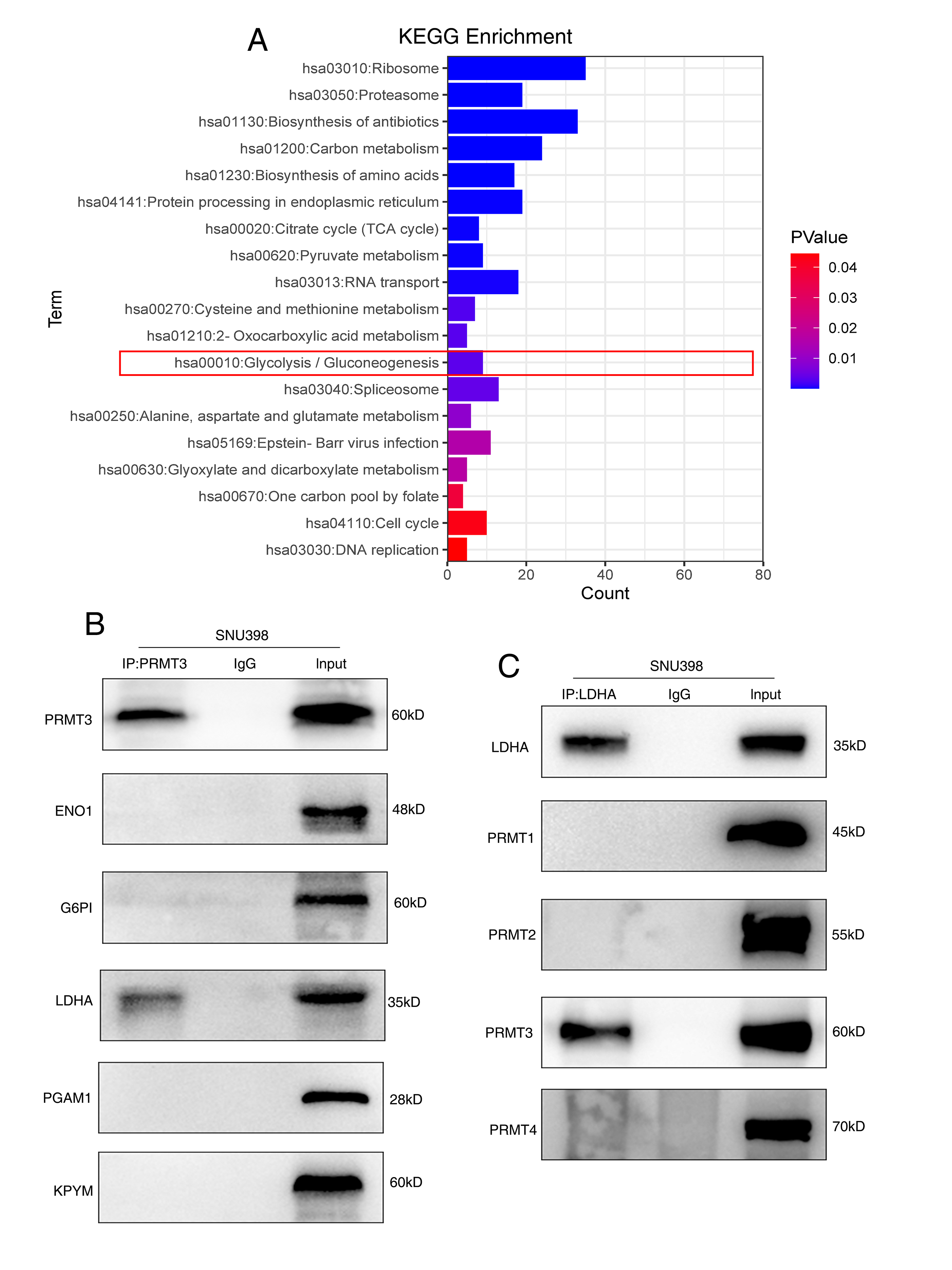

Supplement: Supplementary file 11 — Figure S11 [file CTM2-12-e686-s011.tif]

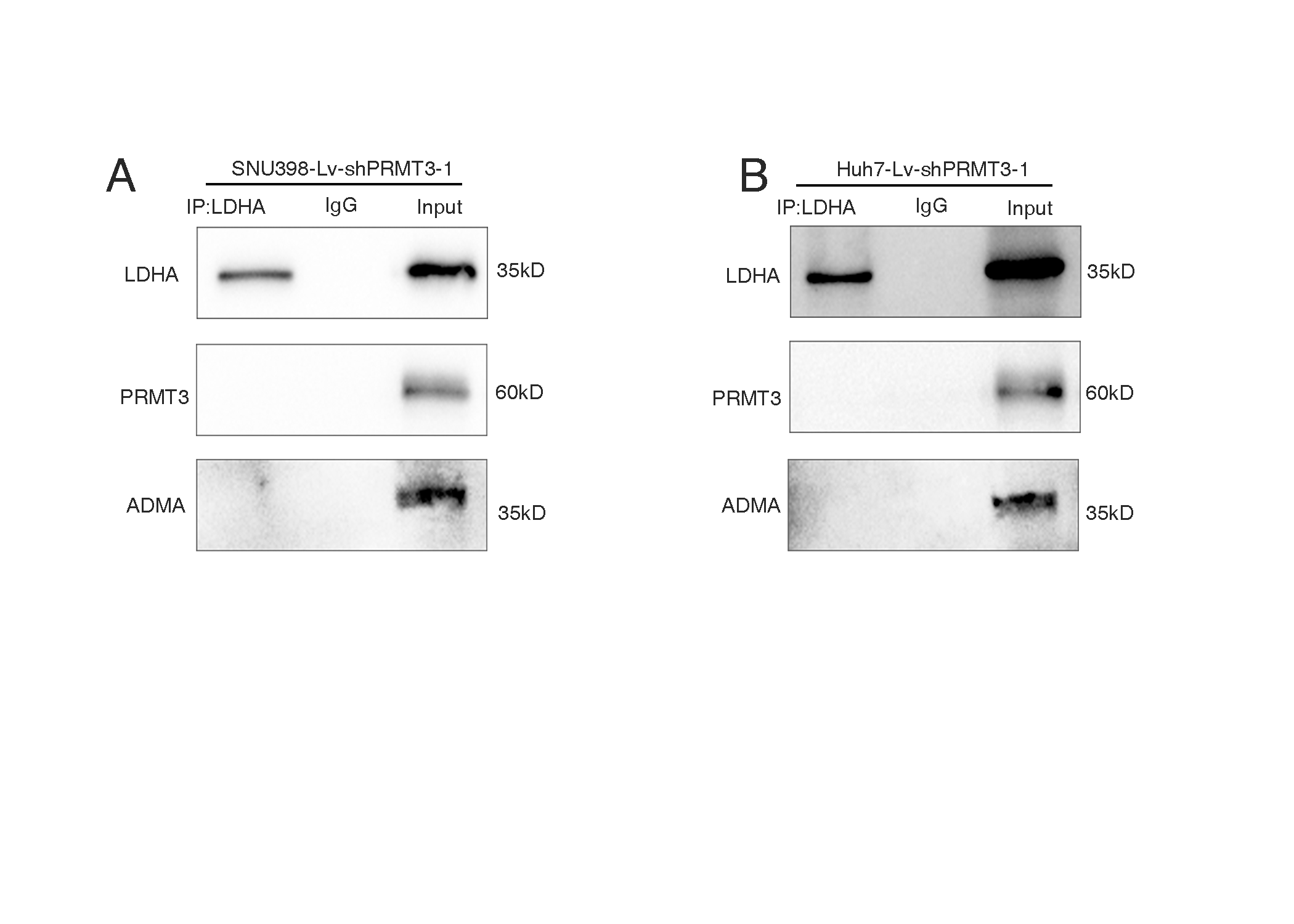

Supplement: Supplementary file 12 — Figure S12 [file CTM2-12-e686-s022.tif]

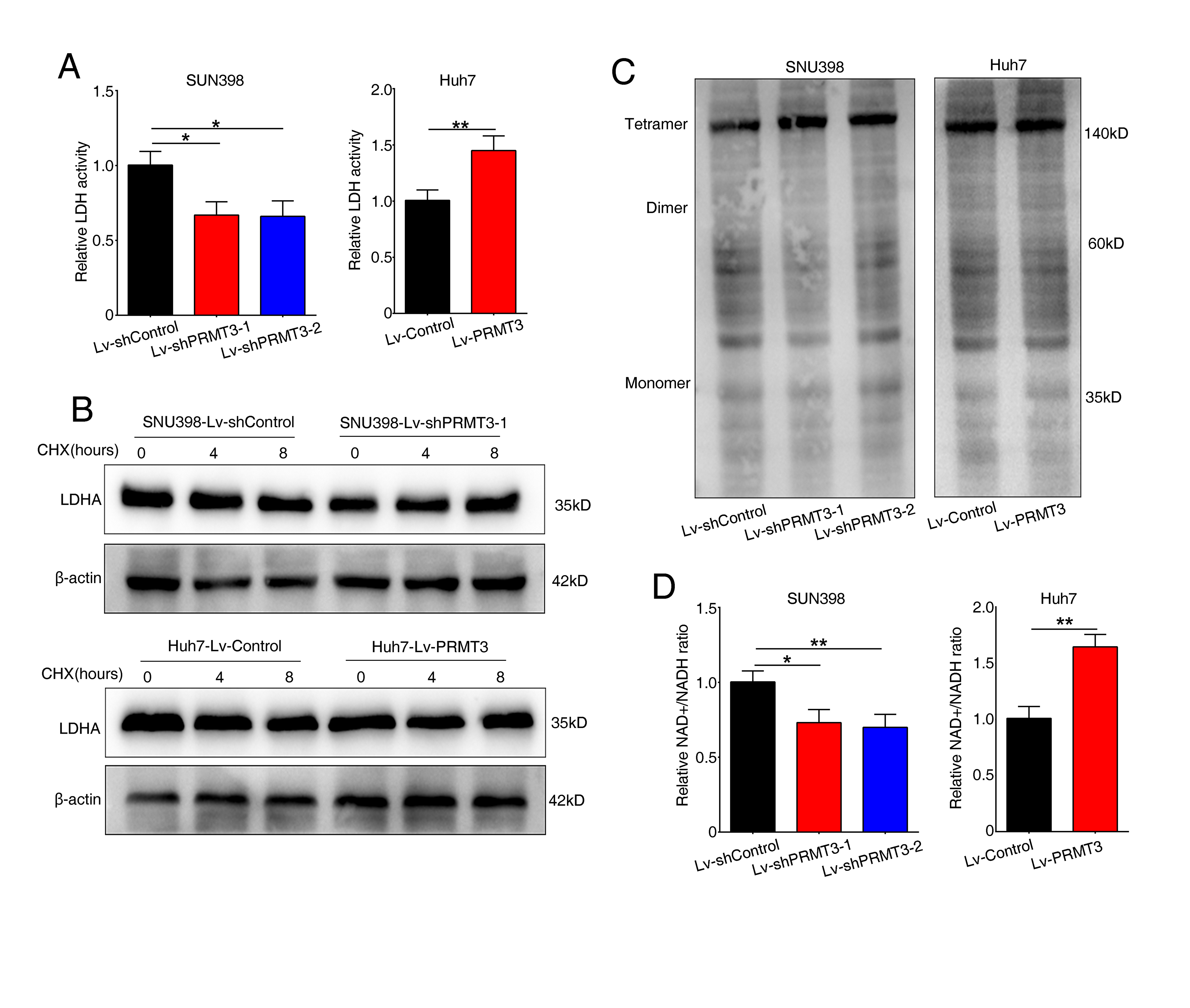

Supplement: Supplementary file 13 — Figure S13 [file CTM2-12-e686-s005.tif]

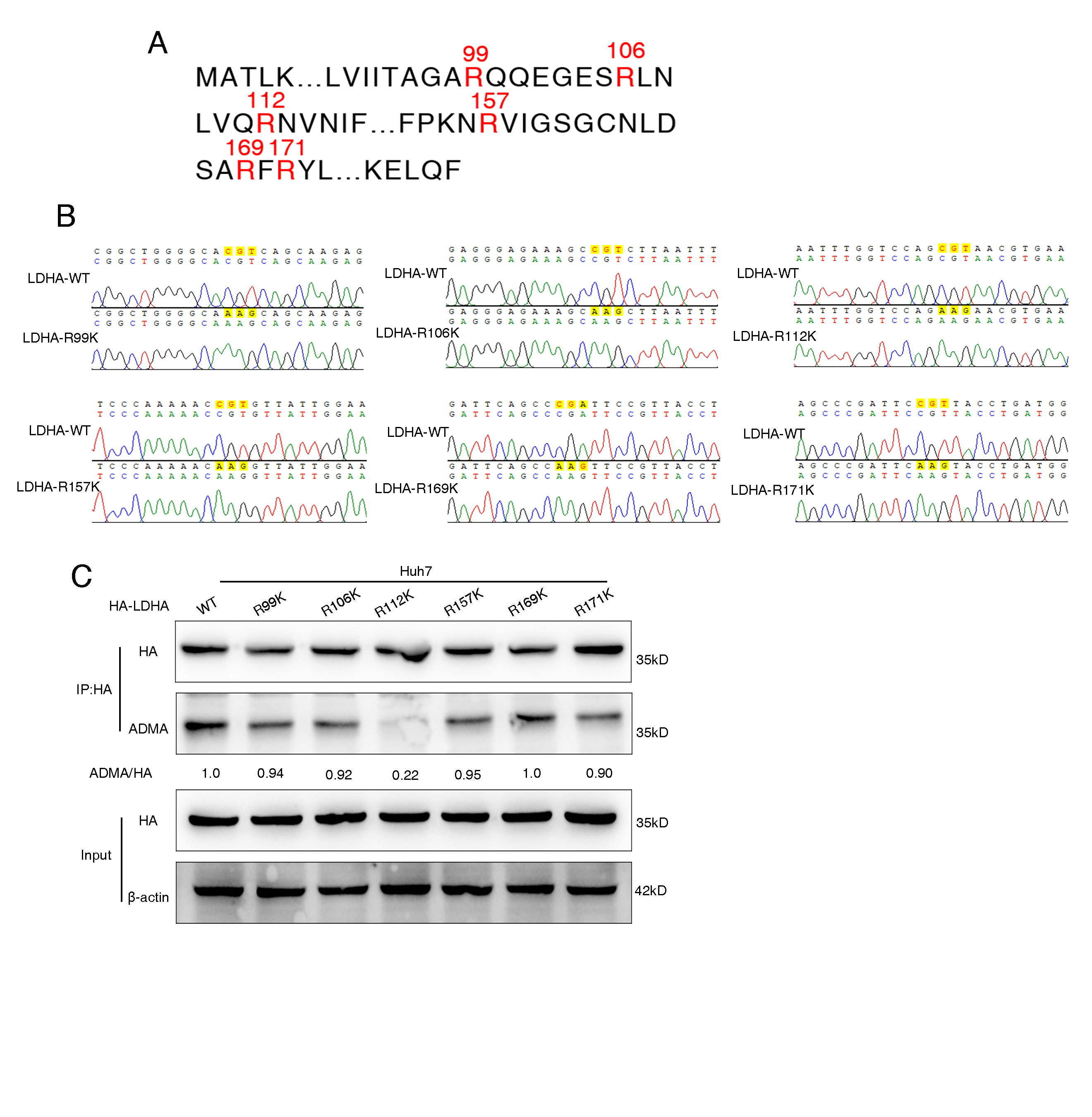

Supplement: Supplementary file 14 — Figure S14 [file CTM2-12-e686-s001.tif]

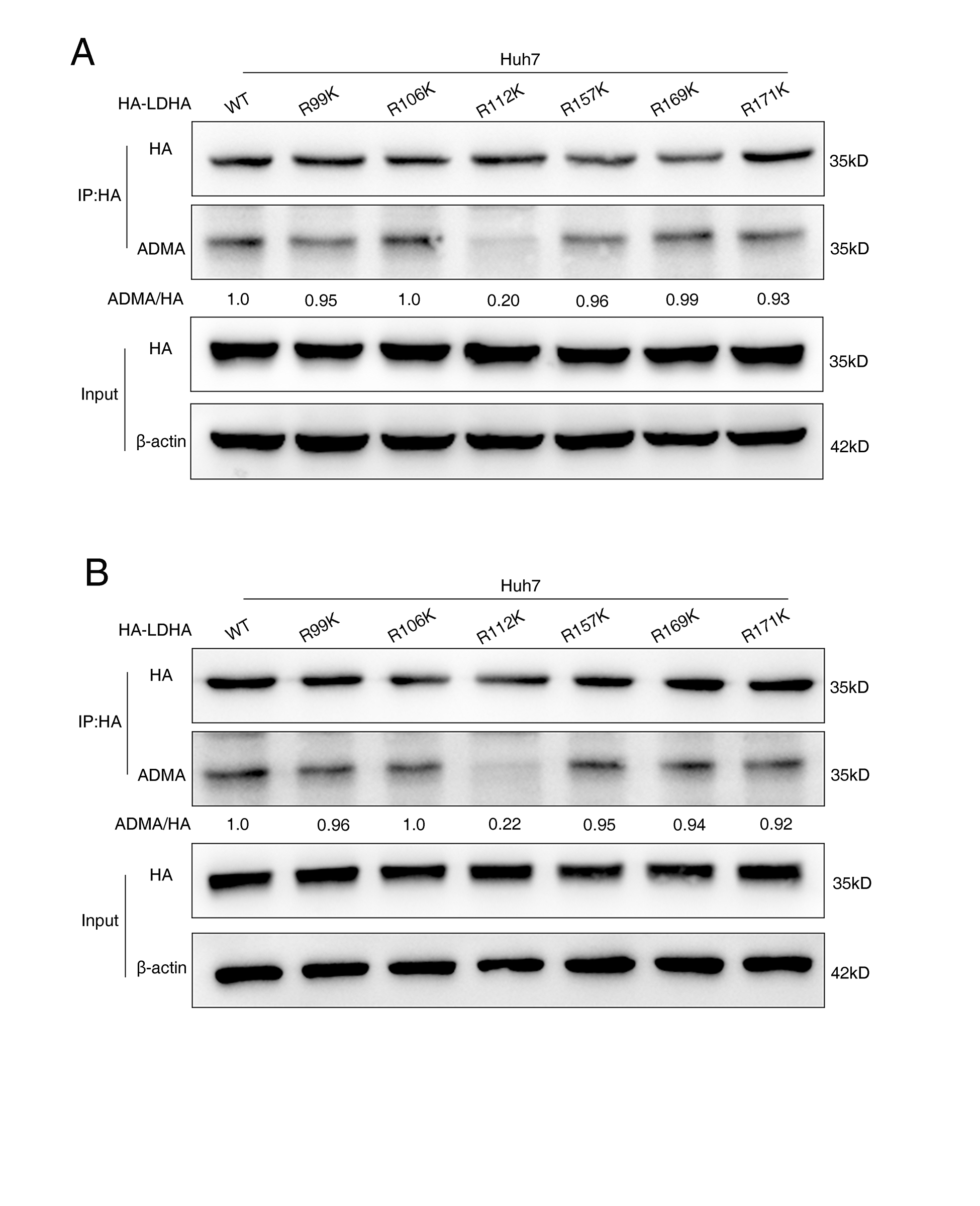

Supplement: Supplementary file 15 — Figure S15 [file CTM2-12-e686-s026.tif]

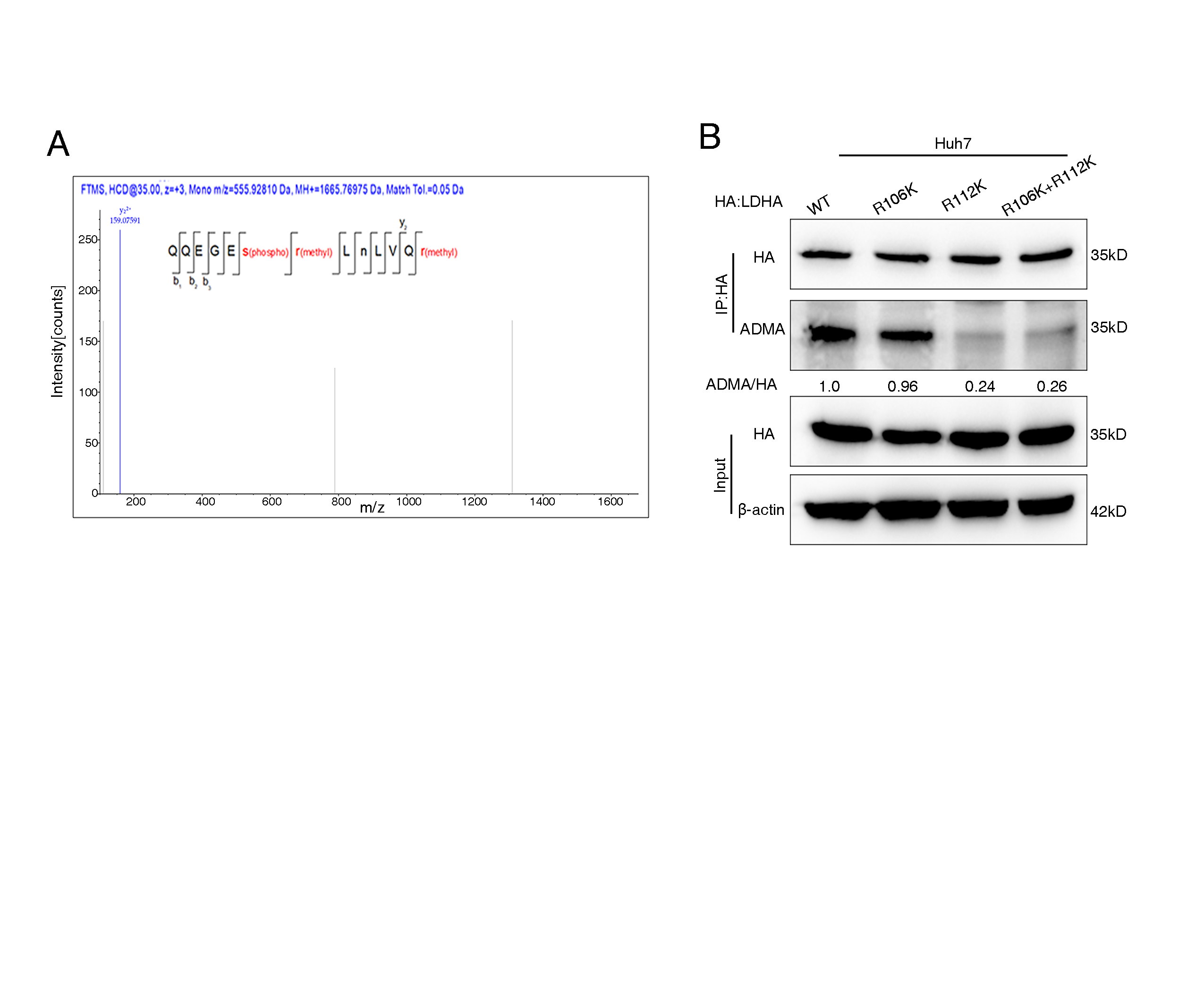

Supplement: Supplementary file 16 — Figure S16 [file CTM2-12-e686-s008.tif]

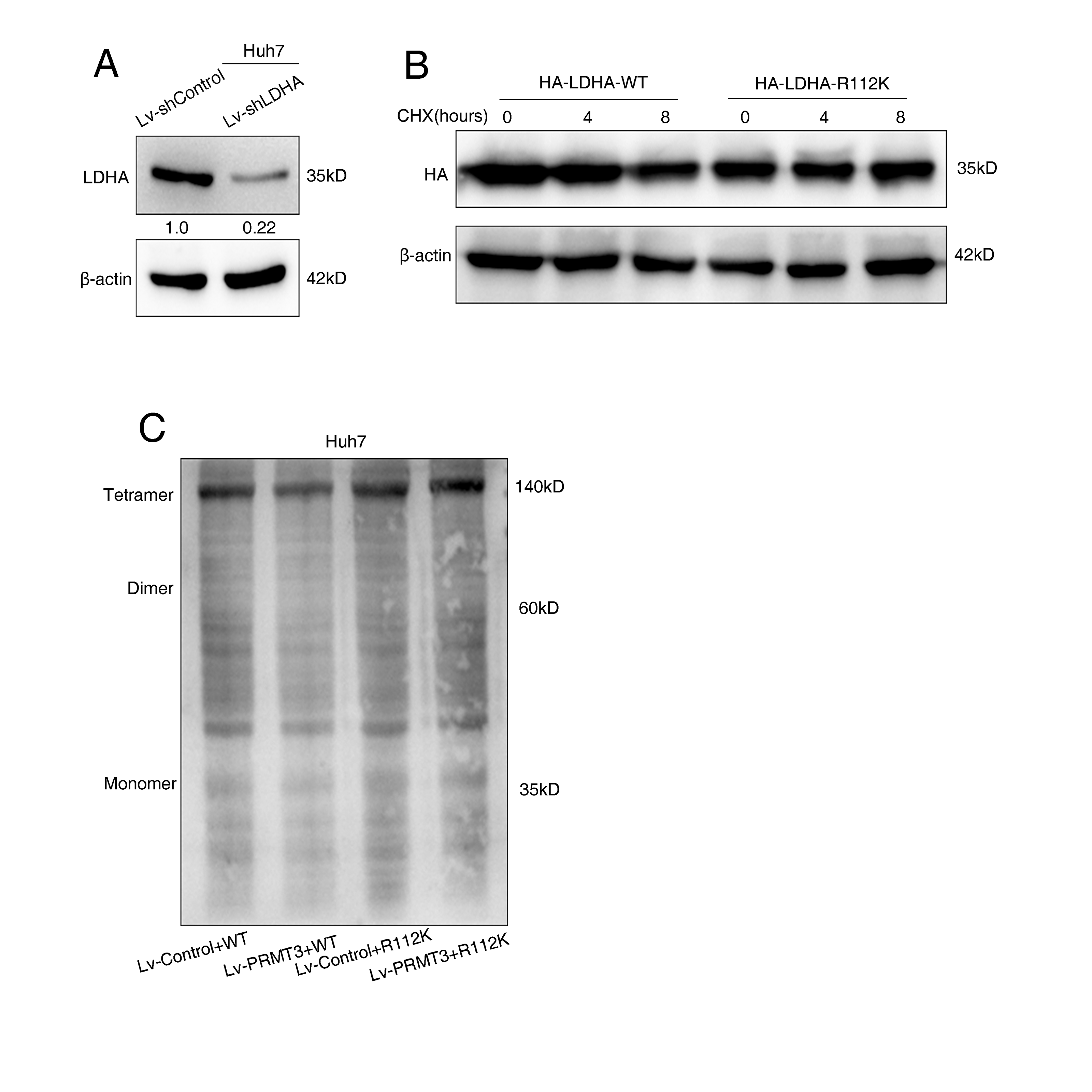

Supplement: Supplementary file 17 — Figure S17 [file CTM2-12-e686-s019.tif]

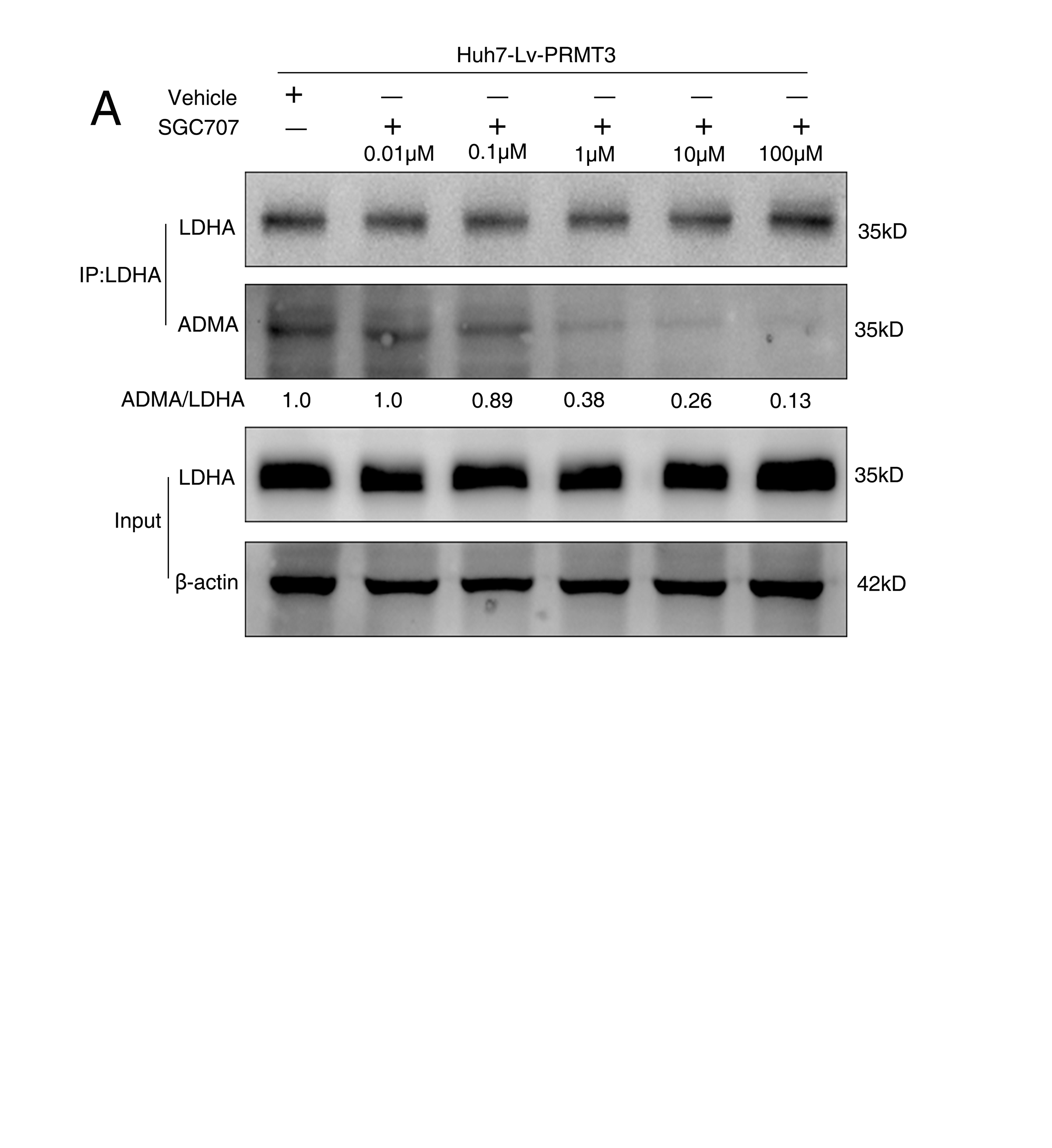

Supplement: Supplementary file 18 — Figure S18 [file CTM2-12-e686-s025.tif]

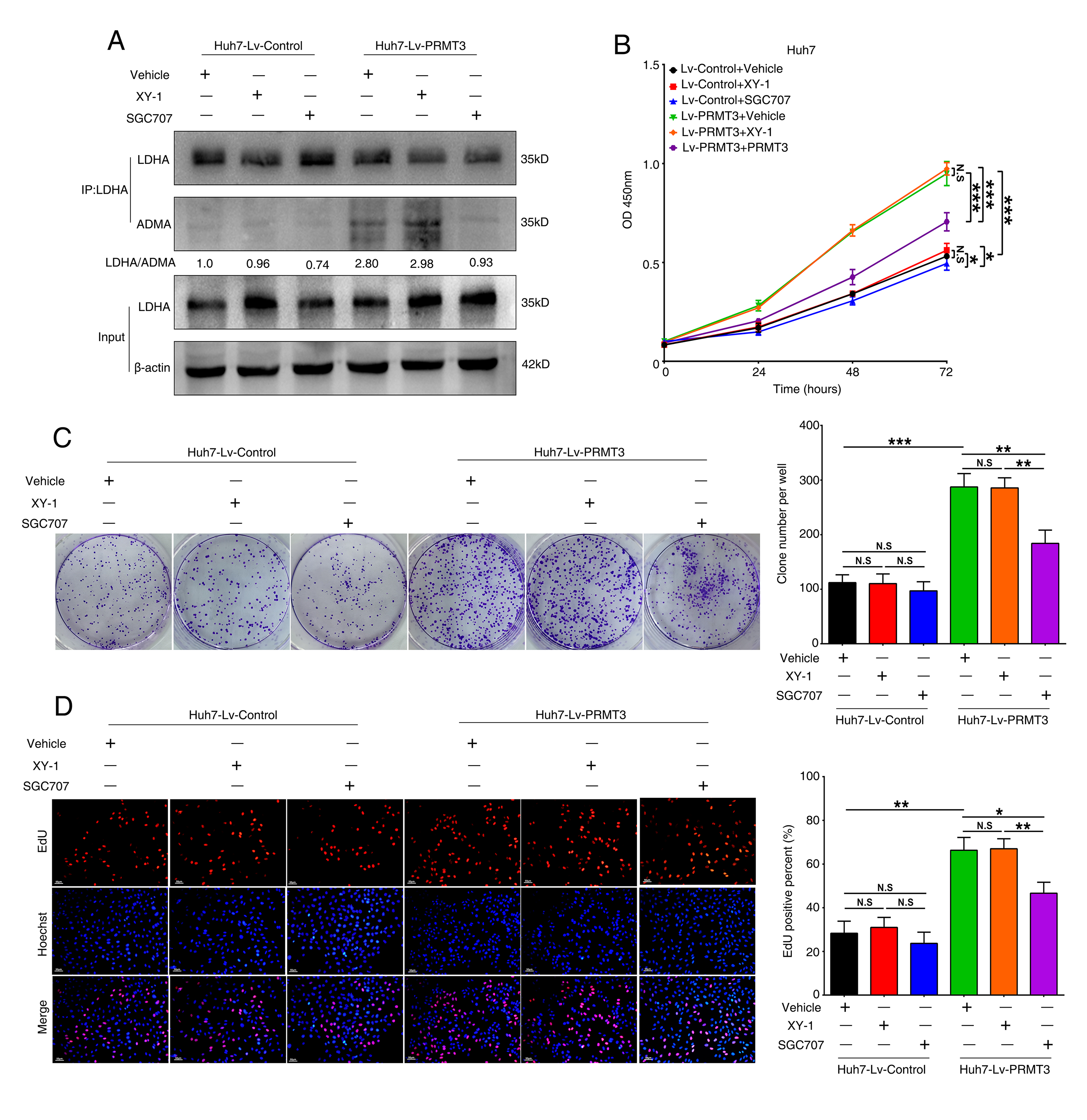

Supplement: Supplementary file 19 — Figure S19 [file CTM2-12-e686-s003.tif]

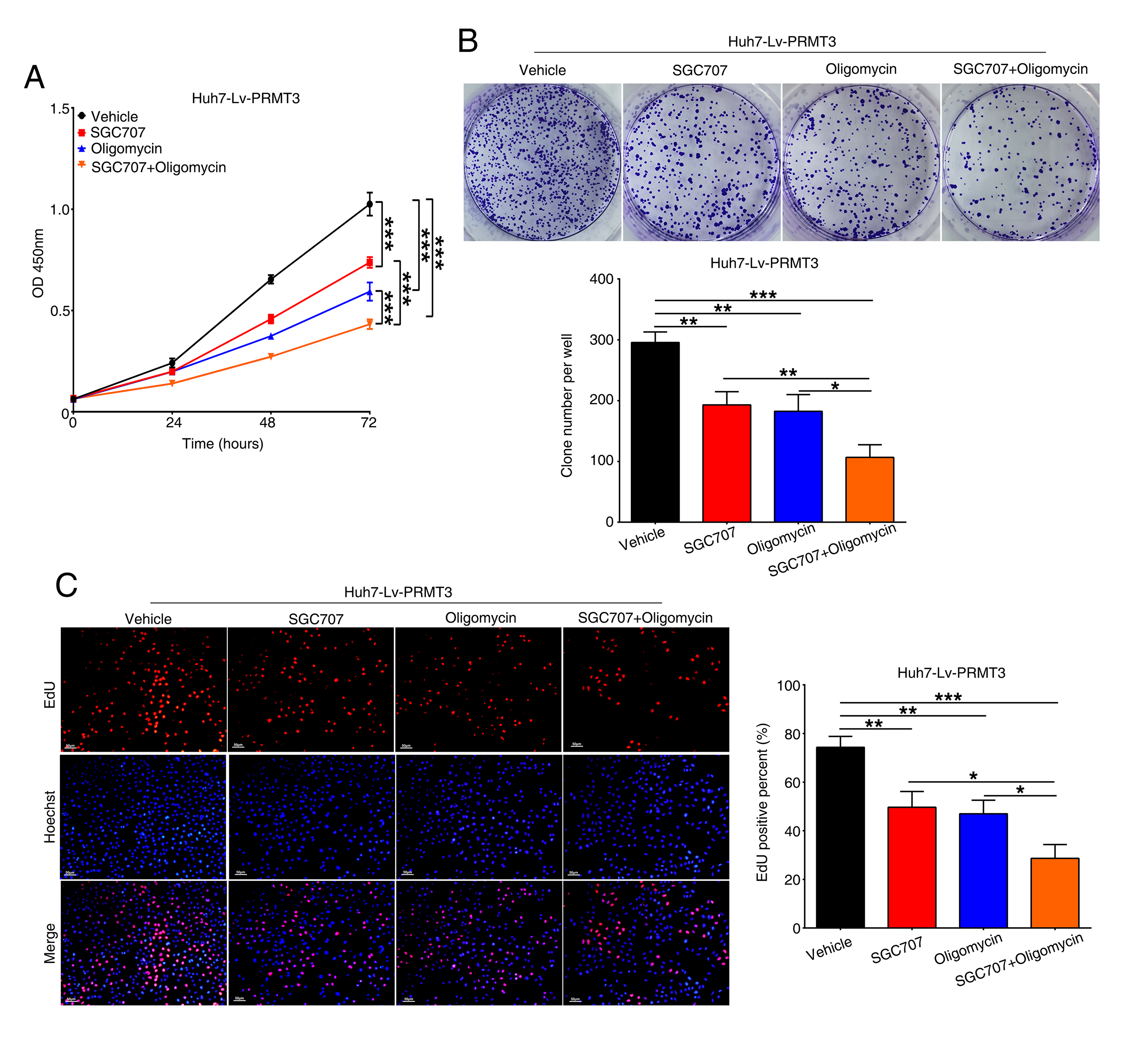

Supplement: Supplementary file 20 — Figure S20 [file CTM2-12-e686-s007.tif]
