# Supplementary material for: Protein arginine methyltransferase 3 promotes glycolysis and hepatocellular carcinoma growth by enhancing arginine methylation of lactate dehydrogenase A
Source: Clin Transl Med. 2022 Jan 28;12(1):e686. doi: 10.1002/ctm2.686 (PMC8797063; doi:10.1002/ctm2.686)
Supplement: Supplementary file 21 — Supporting Information [file CTM2-12-e686-s014.docx]

**Supplementary materials**

**RNA extraction and quantitative real-time-PCR (qRT-PCR)**

The total RNA extraction was conducted using Trizol Reagent (Invitrogen), and reverse transcription was performed using HiScript II Q RT SuperMix for qPCR Kit (Vazyme, cat#R223-01) according to the manufacturer’s instructions. Quantitative PCR was performed on StepOne Real-Time System (Bio-rad) using ChamQ SYBR qPCR Master Mix (Vazyme, cat#Q321-02) according to the manufacturer’s protocol. The qRT-PCR conditions for the reactions were as follows: 95 °C for 30 s, 95°C for 10s and 60°C for 30s for 40 cycles. Gene expression of human tissues was normalized to β-actin mRNA content, and the relative expression of target gene was determined from replicate samples using the 2-ΔΔCt (Ct, cycle threshold). Primer sequences were as follows: β-actin: 5’-CATGTACGTTGCTATCCAGGC-3’ (sense) and 5’-CTCCTTAATGTCACGCACGAT-3’ (antisense); PRMT3: 5’-GTACCCTTCTCATACCCCAATGG-3’ (sense) and 5’-GACGAGCAGGTTCTGACATCT-3’ (antisense).

**Western blotting analyses**

Proteins from lysed cells were fractionated by SDS-PAGE and transferred to nitrocellulose membranes. Nonspecific binding sites were blocked with 5% milk in TBST (120 mM Tris–HCl (pH 7.4), 150 mM NaCl, and 0.05% Tween 20) for one hour at room temperature. Blots were incubated with the specific antibody overnight at 4 °C. Primary antibodies include PRMT3 (Abcam, cat# ab191562), ADMA (Cell Signaling Technology, cat# 13522), LDHA (Cell Signaling Technology, cat# 3582), HA-tag (Proteintech, cat# 66006-2-Ig) (Proteintech, cat# 51064-2-AP), Flag-tag (Proteintech, cat# 20543-1-AP), ENO1 (Cusabio, cat# CSB-PA02395A0Rb), GPI (Abclonal, cat# A6916), PGAM1 (Abclonal, cat# A4015), PKM (Abclonal, cat# A13905), PRMT1 (Abclonal, cat# 1055), PRMT2 (Abclonal, cat# A5835), CARM1 (Abclonal, cat# 2246). Anti-β-actin (Proteintech, cat# 66009-1-Ig) was used as an internal control. The membranes were then washed with PBS three times and incubated with an HRP-conjugated secondary antibody. Immune complexes were visualized using Super ECL Detection Reagent (Yeasen, cat# 36208ES60). Relative grayscale values of the bands were analyzed by Image J software.

**Plasmid and lentiviral shRNA**

Lentiviral vectors encoding the human PRMT3 short hairpin RNAs (shRNA) and PRMT3 were obtained from Genechem (Shanghai, China) and designated as Lv-shPRMT3-1, Lv-shPRMT3-2, Lv-shControl, Lv-PRMT3, Lv-Control. ‘Lv-shControl’ is a non-target shRNA control, and ‘Lv-Control’represents an empty vector as the negative control. The sequences for PRMT3 shRNAs were listed as follow: shPRMT3-1, 5’-GCTGGCTACTTTGATATATAT-3’, shPRMT3-2, 5’-CCTTGTGGTATTAAGCATATA-3’ and control sequence was: 5’-TTCTCCGAACGTGTCACGT-3’. The sequence for LDHA shRNA was: 5’-GCGTAACGTGAACATCTTTAA-3’. LDHA mutants obtained from DesignGene Biotechnology Company (Shanghai, China). The primers used for mutagenesis were shown in Supplementary Table S2.

**Cell counting kit-8 (CCK-8) assay**

For the CCK-8 assay, cells were seeded into 96-well plates at a density of 1000 cells in 100μL of complete medium per well. At each time point, the original medium was replaced with CCK-8 solution (Cell Counting Kit, Promoter, Wuhan, China) and complete medium mixed at a 1:10 ratio, and then the cells were incubated at 37°C for 2 hours. The absorbance of each sample was recorded at 450 nm using a microplate reader (Biotek, USA).

**Colony formation assay**

Cells were seeded at a density of 1000 cells per well in the 6-well plates. After 10 days’ incubation at 37 °C, the clones were fixed by 4% methanol and stained with a 4 g/L crystal violet solution. Clone numbers were counted under the microscope.

**5-Ethynyl-2’-deoxyuridine (EdU) staining assays**

Cells were incubated with 10μM EdU for 2 h with the iClick EdU Andy fluor 555 Imaging Kit (ABP Biosciences, cat# A004) according to the manufacturer’s protocol. The nuclei were counterstained with Hoechst 33342. EdU-positive cells were detected using the Olympus fluorescence microscope (Ex/Em=555/565 nm) and normalized to the total number of Hoechst-positive cells.

**Cell apoptosis assays**

Annexin V-APC/7-AAD apoptosis detecting kits (Nanjing KeyGen Biotech, cat# KGA1023) were used for cell apoptosis assays according to the product’s instruction, and the cells were stained. Flow cytometer (FACSCalibur, BD Biosciences) was performed to determine the cell apoptosis percentages and the results were analyzed with Flow Jo software.

**Immunofluorescence (IF) assays**

For IF assays, cells were fixed with 4% paraformaldehyde at room temperature for 15 min and, then, permeabilized with phosphate-buffered saline containing 0.3% Triton X-100 for 10 min, blocked with 10% goat serum, and incubated with primary antibodies at 4°C overnight. Primary antibodies include PRMT3 (Abcam, cat# ab191562) and LDHA (Proteintech, cat# 66287-1-Ig). The cells were washed with PBS and, then, incubated with the appropriate secondary antibodies (Promoter, Wuhan, China). Fluorescence was detected using an Olympus fluorescence microscope. Pearson’s correlation coefficient was used to reflect the colocalization of two proteins by ImageJ Coloc2 plugin([1](#_ENREF_1)), and IF signal intensity distribution was measured using the ImageJ Radial Profile plugin.

**Glucose consumption and lactate production assays**

Cells were seeded in 6-well plates and a well without cells was as the control. After the cells were attached, they were switched to a complete medium. After 24h of incubation, the medium was collected determine glucose consumption and lactate production. The glucose consumption was measured with a glucose assay kit (Nanjing Jiancheng Biotech, cat# F006-1-1) and the lactate production was determined with a lactate assay kit (Nanjing Jiancheng Biotech, cat# A019-2-1) according to the manufacturer's instructions. Protein concentration was determined for normalization.

**LDH activity assays**

Cells were seeded in 6-well plates of incubation for 24h, then collected to determine LDH activity. LDH activity assay was performed with a LDH assay kit (Nanjing Jiancheng Biotech, cat# A020-2-2) according to the manufacturer's instructions. Briefly, activity of LDH was determined by measuring the NADH oxidation in the reaction containing buffer solution, coenzyme I, 2,4-dinitrophenylhydrazine, 0.4 mol/L NADH using a microplate reader (OD=450nm) and 0.2μmol/ml pyruvate was used as the standard control. Protein concentration was determined for normalization.

**NAD+/NADH assays**

Cells were seeded in 6-well plates of incubation for 24h, then collected to determine NAD+/NADH ratio. The NAD+/NADH ratio assay was conducted with a NAD+/NADH assay kit with WST-8 (Beyotime, cat# S0175) following the product’s protocol([2](#_ENREF_2)). In brief, cells were lysed with 200μL of cold lysis buffer. To measure total NAD+/NADH, 20μL of cell lysates was added to a 96-well plate. For NADH measurement, the lysed cell suspension was incubated at 60°C for 30 min and 20 μL was added to a 96-well plate. Then, 90μL of alcohol dehydrogenase was added and incubated at 37°C for 10 min. Finally, 10μL of chromogenic solution was added to the plate and the mixture was incubated at 37°C for 30 min. Standard curve was generated and measured at the same time as the samples. The absorbance values were measured at 450nm. The amount of NAD+ was derived by subtracting NADH from total NAD+/NADH. Protein concentration was determined for normalization.

**Database analysis**

The gene expression profiles of HCC patients were obtained from the The Cancer Genome Atlas (TCGA) dataset (<https://www.cancer.gov/tcga/>). HCC samples were divided into low PRMT3 expression group or high PRMT3 expression group, and the median value was set as cutoff. As for Gene Set Enrichment Analysis (GSEA), GSEA v4.0.1 was applied to perform KEGG analysis to explore the potential pathways involved in HCC pathogenesis through PRMT3. The correlation between mRNA expression levels of PRMTs and prognosis of HCC patients was evaluated using Kaplan-Meier Plotter ([https://www.kmplot.com/](http://www.kmplot.com/)), an online database containing information about the effects of 54675 genes on survival in more than 20 types of cancers([3](#_ENREF_3)). Significant difference was considered when log rank P-value < 0.05.

**References**

1. Dunn KW, Kamocka MM, McDonald JH. A practical guide to evaluating colocalization in biological microscopy. American journal of physiology Cell physiology. 2011;300(4):C723-42.

2. Li CG, Zeng QZ, Chen MY, Xu LH, Zhang CC, Mai FY, et al. Evodiamine Augments NLRP3 Inflammasome Activation and Anti-bacterial Responses Through Inducing alpha-Tubulin Acetylation. Frontiers in pharmacology. 2019;10:290.

3. Gyorffy B, Lanczky A, Eklund AC, Denkert C, Budczies J, Li Q, et al. An online survival analysis tool to rapidly assess the effect of 22,277 genes on breast cancer prognosis using microarray data of 1,809 patients. Breast cancer research and treatment. 2010;123(3):725-31.

**Supplementary Figure legends**

**Supplementary Figure S1**

(A-I) The mRNA expression level of PRMTs in normal liver tissues (n=50) versus HCC tissues (n=373) in TCGA database.

The data are represented as Mean ± S.D. *P < 0.05, **P < 0.01, ***P < 0.001, ****P < 0.0001. N.S, no significance.

**Supplementary Figure S2**

(A-I) The correlation between mRNA expression levels of PRMTs and overall survival of HCC patients using Kaplan-Meier Plotter analysis.

**Supplementary Figure S3**

(A) Western blotting analysis showing global ADMA level of HCC cells with PRMT3 knockdown or overexpression (n=3).

**Supplementary Figure S4**

(A) Western blotting analysis of relative protein level of PRMT3 in HCC cell lines.

(B) Western blotting analysis confirmed the expression of PRMT3 in the indicated MHCC97H cells (left) and Hep3B cells (right) after lentivirus transfection (n=3).

(C) CCK-8 assays showed the proliferation capacities of MHCC97H cells with PRMT3 knockdown and Hep3B cells with PRMT3 overexpression (n=3).

(D) Colony formation assays were used to analyze the proliferation abilities of the indicated MHCC97H cells and Hep3B cells (n=3).

(E) EdU assays were performed to detect the cell proliferation of the indicated MHCC97H cells and Hep3B cells (n=3, scale bars: 50μm).

The experiments were replicated n times, and the data are represented as Mean ± S.D. *P < 0.05, **P < 0.01, ***P < 0.001.

**Supplementary Figure S5**

(A-D) Annexin V-APC/7AAD staining analysis of the indicated cells apoptosis by flow cytometry (n=3).

The experiments were replicated n times, and the data are represented as Mean ± S.D. *P < 0.05, **P < 0.01.

**Supplementary Figure S6**

(A) Representative images of colors of the indicated SNU398 cells and Huh7 cells culture medium.

(B) The pH value of the indicated SNU398 cells and Huh7 cells culture medium (n=3).

The experiments were replicated n times, and the data are represented as Mean ± S.D. *P < 0.05.

**Supplementary Figure S7**

(A) The hierarchical clustering heatmap of differentially metabolites between control and PRMT3-overexpressing Huh7 cells in negative ion mode.

(B) Bubble plot of pathway analysis for group Control versus PRMT3-overexpressing Huh7 cells in negative ion mode. The color depth and bubble size indicated -ln *P*-values and impact of the pathway.

**Supplementary Figure S8**

(A) OCR measured in PRMT3-knockdown and control SNU398 cells. Basal OCR measurement was measured in XF assay medium, following by oligomycin (1.5μM), FCCP (1.0μM), and rotenone/antimycin A (Rtn/AA) (0.5μM). Column statistics of ECAR was shown in the right panel (n=3).

(B) OCR measured in PRMT3-overexpressing and control Huh7 cells. Column statistics was shown in the right panel (n=3).

The experiments were replicated n times, and the data are represented as Mean ± S.D. *P < 0.05. N.S, no significance.

**Supplementary Figure S9**

(A) Relative glucose consumption of MHCC97H cells with PRMT3 knockdown and Hep3B cells with PRMT3 overexpression (n=3).

(B) Relative lactate production of the indicated MHCC97H cells and Hep3B cells (n=3).

(C) ECAR measured in PRMT3-knockdown and control MHCC97H cells. Column statistics of ECAR was shown in the right panel (n=3).

(D) ECAR measured in PRMT3-overexpressing and control Hep3B cells. Column statistics was shown in the right panel (n=3).

(E) OCR measured in PRMT3-knockdown and control MHCC97H cells. Column statistics of ECAR was shown in the right panel (n=3).

(F) OCR measured in PRMT3-overexpressing and control Hep3B cells. Column statistics was shown in the right panel (n=3).

The experiments were replicated n times, and the data are represented as Mean ± S.D. *P < 0.05, **P < 0.01. N.S, no significance.

**Supplementary Figure S10**

(A) PRMT3-overexpressing and control cells were treated with DMSO and 2-DG (10 μM) for 24h, CCK-8 assays showed the proliferation capacities of the indicated HCC cells (n=3).

(B) Colony formation assays were used to analyze the proliferation abilities of the indicated HCC cells (n=3).

(C) EdU assays were performed to detect the proliferation of the indicated HCC cells (n=3, scale bars: 50μm).

The experiments were replicated n times, and the data are represented as Mean ± S.D. *P < 0.05, **P < 0.01, ***P < 0.001. N.S, no significance.

**Supplementary Figure S11**

(A) KEGG enrichment analysis of potential PRMT3 interacting proteins identified by MS.

(B) PRMT3 proteins were immunoprecipitated from SNU398 cells and subjected to western blotting assays to detect its interaction with five proteins we mentioned (n=3).

(C) LDHA proteins were immunoprecipitated from SNU398 cells and subjected to western blotting assays to detect its interaction with PRMT1/2/3/4 (n=3).

The experiments were replicated n times.

**Supplementary Figure S12**

(A) LDHA proteins were immunoprecipitated from SNU398 cells with PRMT3 knockdown, and subjected to western blotting assays to detect its interaction with PRMT3, as well as the signal of ADMA (n=3).

(B) LDHA proteins were immunoprecipitated from Huh7 cells with PRMT3 knockdown, and subjected to western blotting assays to detect its interaction with PRMT3, as well as the signal of ADMA (n=3).

The experiments were replicated n times.

**Supplementary Figure S13**

(A) LDH activity assays of HCC cells with PRMT3 knockdown or overexpression (n=3).

(B) The indicated SNU398 and Huh7 cells were treated with CHX (10 μg/ml) for the indicated time points. Then, the cell lysates were collected and analyzed by western blot (n=3).

(C) Cell lysates of the indicated cells were collected and subjected to 10% native gel electrophoresis for detecting LDHA tetramers (n=3).

(D) NAD+/NADH assays of HCC cells with PRMT3 downregulation or upregulation (n=3).

The experiments were replicated n times. and the data are represented as Mean ± S.D. *P < 0.05, **P < 0.01.

**Supplementary Figure S14**

(A) Six potential arginine residues (R99, R106, R112, R157, R169, R171) were predicted combining amino acid sequence analysis of LDHA across multiple species and GPS-MSP.

(B) Sequence of the series of R-to-K LDHA mutants generated.

(C) WT and R-to-K mutant LDHA proteins were immunoprecipitated with HA beads, and then subjected to western blotting assays to detect the level of ADMA.

**Supplementary Figure S15**

(A-B) WT and R-to-K mutant LDHA proteins were immunoprecipitated with HA beads, and then subjected to western blots to detect the level of ADMA (Another two replicates).

**Supplementary Figure S16**

(A) Secondary mass spectrometry result of the potential methylation residues.

(B) HA beads were immunoprecipitated from the indicated HCC cells, including LDHA-WT-overexpressing cells, LDHA-R106K-overexpressing cells, LDHA-R112K-overexpressing cells, and LDHA-R106K+R112K-overexpressing cells, then subjected to western blotting assays to detect the level of ADMA (n=3).

The experiments were replicated n times.

**Supplementary Figure S17**

(A) Western blotting analysis confirmed the expression of LDHA in the Huh7 cells with LDHA knockdown after lentivirus transfection (n=3).

(B) The indicated Huh7 cells were treated with CHX (10 μg/ml) for the indicated time points. Then, the cell lysates were collected and analyzed by western blot (n=3).

(C) Cell lysates of the indicated cells were collected and subjected to 10% native gel electrophoresis for detecting LDHA tetramers (n=3).

The experiments were replicated n times.

**Supplementary Figure S18**

(A) LDHA proteins were immunoprecipitated from the indicated HCC cells, including PRMT3-overexpressing cells with DMSO treatment, and PRMT3-overexpressing cells with SGC707 (0.01, 0.1, 1, 10, 100μM) treatment for 48h respectively, then subjected to western blotting assays to detect the level of ADMA (n=3).

The experiments were replicated n times.

**Supplementary Figure S19**

(A) LDHA proteins were immunoprecipitated from the indicated HCC cells, including control and PRMT3-overexpressing cells with DMSO, XY-1 (100μM), and SGC707 (1μM) treatment for 48h respectively, then subjected to western blotting assays to detect the level of ADMA (n=3).

(B) CCK-8 assays showed the proliferation capacities of the indicated HCC cells (n=3).

(C) Colony formation assays were used to analyze the proliferation abilities of the indicated HCC cells (n=3).

(D) EdU assays were performed to detect the proliferation of the indicated HCC cells (n=3, scale bars: 50μm).

The experiments were replicated n times, and the data are represented as Mean ± S.D. *P < 0.05, **P < 0.01, ***P < 0.001.N.S, no significance.

**Supplementary Figure S20**

(A) CCK-8 assays showed the proliferation capacities of the indicated HCC cells, including PRMT3-overexpressing cells with DMSO, SGC707 (1μM), oligomycin (2μM) and SGC707+oligomycin treatment for 48h, respectively (n=3).

(B) Colony formation assays were used to analyze the proliferation abilities of the indicated HCC cells (n=3).

(C) EdU assays were performed to detect the proliferation of the indicated HCC cells (n=3, scale bars: 50μm).

The experiments were replicated n times, and the data are represented as Mean ± S.D. *P < 0.05, **P < 0.01, ***P < 0.001.

**Supplementary Table S1**

Univariate and multivariate analysis of factors associated with overall survival in HCC patients.

**Supplementary Table S2**

Primer sequences used for LDHA mutagenesis.

**Supplementary Table S3**

Proteins identified by mass spectrometry that may interact with PRMT3.

**Supplementary Table S4**

Differentially metabolites between control and PRMT3-overexpressing Huh7 cells in positive ion mode.

**Supplementary Table S5**

Differentially metabolites between control and PRMT3-overexpressing Huh7 cells in negative ion mode.
