# Supplementary material for: Protein arginine methyltransferase 3 promotes glycolysis and hepatocellular carcinoma growth by enhancing arginine methylation of lactate dehydrogenase A
Source: Clin Transl Med. 2022 Jan 28;12(1):e686. doi: 10.1002/ctm2.686 (PMC8797063; doi:10.1002/ctm2.686)
Supplement: Supplementary file 22 — Table S1 [file CTM2-12-e686-s006.docx]

|  | Overall survival | |
| --- | --- | --- |
| Clinical Variables | HR(95%CI) | P value |
| **Univariate Analysis** |  |  |
| Age (≤45 versus >45 years old) | 1.016(0.593-1.742) | 0.954 |
| Sex (female versus male) | 0.577(0.230-1.446) | 0.241 |
| Serum AFP (≤20 versus >20 ng/ml) | 0.721(0.428-1.215) | 0.219 |
| HBsAg infection (negative versus positive) | 0.962(0.509-1.818) | 0.904 |
| Tumor number (single versus multiple) | 0.830(0.453-1.518) | 0.545 |
| Maximal tumor size (≤5 versus >5 cm) | 0.657(0.340-1.269) | 0.211 |
| TNM stage (I-II versus III-IV) | 0.451(0.259-0.787) | **0.005*** |
| PRMT3 (low versus high expression) | 0.484(0.285-0.821) | **0.007*** |
| **Multivariate analysis** |  |  |
| TNM stage (I-II versus III-IV) | 0.520(0.294-0.922) | **0.025*** |
| PRMT3 (low versus high expression) | 0.567(0.329-0.977) | **0.041*** |

**Supplementary Table S1. Univariate and multivariate analysis of factors associated with overall survival in HCC patients (n=81)**
