# Supplementary material for: Protein arginine methyltransferase 3 promotes glycolysis and hepatocellular carcinoma growth by enhancing arginine methylation of lactate dehydrogenase A
Source: Clin Transl Med. 2022 Jan 28;12(1):e686. doi: 10.1002/ctm2.686 (PMC8797063; doi:10.1002/ctm2.686)
Supplement: Supplementary file 23 — Table S2 [file CTM2-12-e686-s010.docx]

**Supplementary Table S2. Primer sequences used for mutagenesis**

| Primer name | Primer sequences |
| --- | --- |
| R99K-LDHA (Forward): | 5’-CACGGCTGGGGCAAAGCAGCAAGAGGGAGAAAG-3’ |
| R99K-LDHA (Reverse): | 5’-CTTTCTCCCTCTTGCTGCTTTGCCCCAGCCGTG-3’ |
| R106K-LDHA (Forward): | 5’-CAAGAGGGAGAAAGCAAGCTTAATTTGGTCCAGCG-3’ |
| R106K-LDHA (Reverse): | 5’-CGCTGGACCAAATTAAGCTTGCTTTCTCCCTCTTG-3’ |
| R112K-LDHA (Forward): | 5’-GTCTTAATTTGGTCCAGAAGAACGTGAACATCTTTAAAT-3’ |
| R112K-LDHA (Reverse): | 5’-ATTTAAAGATGTTCACGTTCTTCTGGACCAAATTAAGAC-3’ |
| R157K-LDHA (Forward): | 5’-GTGGTTTTCCCAAAAACAAGGTTATTGGAAGTGGTTG-3’ |
| R157K-LDHA (Reverse): | 5’-CAACCACTTCCAATAACCTTGTTTTTGGGAAAACCAC-3’ |
| R169K-LDHA (Forward): | 5’-CAATCTGGATTCAGCCAAGTTCCGTTACCTGATGGG-3’ |
| R169K-LDHA (Reverse): | 5’-CCCATCAGGTAACGGAACTTGGCTGAATCCAGATTG-3’ |
| R171K-LDHA (Forward): | 5’-GGATTCAGCCCGATTCAAGTACCTGATGGGGGAAAG-3’ |
| R171K-LDHA (Reverse): | 5’-CTTTCCCCCATCAGGTACTTGAATCGGGCTGAATCC-3’ |
| E338Q-PRMT3 (Forward): | 5’-GTGGATGGGCTATTTTCTTCTGTTTCAGTCTATGTTAGATTCTGTCCTTTATG-3’ |
| E338Q-PRMT3 (Reverse): | 5’-CATAAAGGACAGAATCTAACATAGACTGAAACAGAAGAAAATAGCCCATCCAC-3’ |
