# Supplementary material for: Protein arginine methyltransferase 3 promotes glycolysis and hepatocellular carcinoma growth by enhancing arginine methylation of lactate dehydrogenase A
Source: Clin Transl Med. 2022 Jan 28;12(1):e686. doi: 10.1002/ctm2.686 (PMC8797063; doi:10.1002/ctm2.686)
Supplement: Supplementary file 24 — Table S3 [file CTM2-12-e686-s016.docx]

| **Supplementary Table S3. Proteins identified by mass spectrometry that may interact with PRMT3** | | | | | | |
| --- | --- | --- | --- | --- | --- | --- |
| **Protein_ID** | **Protein_Qscore** | **Protein_Mass** | **Peptides** | **Unique_Peptide_Num** | **Coverage** | **Description** |
| sp\|O75369\|FLNB_HUMAN | 105.3339388 | 277990.0528 | AGPGTLSVTIEGPSK;AHGPGLEGGLVGKPAEFTIDTK;AWGPGLHGGIVGR;DAGEGLLAVQITDQEGKPK;EAGAGGLSIAVEGPSK;EATTDFTVDSRPLTQVGGDHIK;EVGEHLVSIK;FNGSHVVGSPFK;GEAGVPAEFSIWTR;GQHVTGSPFQFTVGPLGEGGAHK;HTIAVVWGGVNIPHSPYR;IAGPGLGSGVR;IGNLQTDLSDGLR;LDVTILSPSR;LIALLEVLSQKR;LKPGAPLKPK;LPNNHIGISFIPR;LVSPGSANETSSILVESVTR;NTVELLVEDK;SPFTVGVAAPLDLSK;TATPEIVDNKDGTVTVR;VEVGKDQEFTVDTR;VFGPGIEGK;VLFASQEIPASPFR;VNIGQGSHPQK;VPVKDVVDPSK;VTVLFAGQHISK;VVASGPGLEHGK;YADEEIPR;YGGPNHIVGSPFK;YHQRPTFR | 31 | 0.1703 | Filamin-B OS=Homo sapiens OX=9606 GN=FLNB PE=1 SV=2 |
| sp\|P78527\|PRKDC_HUMAN | 71.99091734 | 468787.9037 | ATQQQHDFTLTQTADGR;AYVPALQMAFK;FVPLLPGNR;FYGELALK;HSSLITPLQAVAQR;IAPYSVEIK;KFIQTFGK;LGASLAFNNIYR;LGNPIVPLNIR;LKPVELR;LLALNSLYSPK;LLEEALLR;LLNFLMK;LLPAELPAKR;LPLISGFYK;LPPDVLR;LYSLALHPNAFK;MAVLALLAK;SLGPPQGEEDSVPR;SPNLWLK;TQEGSLSAR;TVGALQVLGTEAQSSLLK;VFLALAAK;VIAGLYQR;VPTYKDYVDLFR | 25 | 0.0618 | DNA-dependent protein kinase catalytic subunit OS=Homo sapiens OX=9606 GN=PRKDC PE=1 SV=3 |
| sp\|Q14204\|DYHC1_HUMAN | 70.52025108 | 532071.8312 | AATSPALFNR;ALGEYLER;DLFQVAFNR;GWDDLFNK;IFVFEPPPGVK;INEWLTLVEK;LGEDLNKWQALLVQIR;LLLIQAFRPDR;LLNTFLER;NTISLLVAGLK;QALVAIFTHLR;QLQNISLAAASGGAK;QNLDALLNQLK;QNLFTTWSHHLQQANIQFR;SLLQALNEVK;TEYLSNADER;TKPVTGNLRPEEALQALTIYEGK;TSFLDDAFR;TVENIKDPLFR;VDNEFDQR;VNFLPEIITLSK;VPLAIVNK;VQVALEELQDLK;WFTSQVIR | 24 | 0.0579 | Cytoplasmic dynein 1 heavy chain 1 OS=Homo sapiens OX=9606 GN=DYNC1H1 PE=1 SV=5 |
| sp\|P35580\|MYH10_HUMAN | 74.27928406 | 228857.9499 | ALAYDKLEK;ALEEALEAKEEFER;ALEQQVEEMR;ASRDEIFAQSK;DEIFAQSK;DLSEELEALK;ELDDATEANEGLSR;ELEAELEDERK;FVAELWK;HATALEELSEQLEQAK;HGFEAASIKEER;KEEELQGALAR;KVDDDLGTIESLEEAK;LQNELDNVSTLLEEAEKK;LQQELDDLTVDLDHQR;QLEEAEEEATR;SDLLLEGFNNYR;TFHIFYQLLSGAGEHLK;TGLEDPER;TTLQVDTLNAELAAER;VIQYLAHVASSHK;VVSSVLQFGNISFK | 22 | 0.206 | Myosin-10 OS=Homo sapiens OX=9606 GN=MYH10 PE=1 SV=3 |
| sp\|Q9Y490\|TLN1_HUMAN | 64.2689117 | 269599.0446 | ADAEGESDLENSR;AGFLDLKDFLPK;ALEATTEHIR;AVAEQIPLLVQGVR;AVASAAAALVLK;DPPSWSVLAGHSR;EGTETFADHR;EVIQEWNLTNIK;IGITNHDEYSLVR;ILAQATSDLVNAIK;LAQAAQSSVATITR;LNEAAAGLNQAATELVQASR;NLGTALAELR;QAAASATQTIAAAQHAASTPK;TLAESALQLLYTAK;TSTPEDFIR;VSHVLAALQAGNR | 17 | 0.0882 | Talin-1 OS=Homo sapiens OX=9606 GN=TLN1 PE=1 SV=3 |
| sp\|O60678\|ANM3_HUMAN | 61.6806663 | 59864.78487 | ALSAEAALAR;AVIPEAVVEVLDPK;DFIYQNPHIFK;DFIYQNPHIFKDK;GKIEEVHLPVEK;IAFWDDVYGFK;IEEVHLPVEK;KAVIPEAVVEVLDPK;LEDTITLIK;LFTSAEETFSHCK;LKNPTVEYMNSIYNPVPWEK;LNKLEDTITLIK;QFAQDFVMHTDVR;QTVFLLEKPFSVK;SEHQFNIDSMVHK;TESYRDFIYQNPHIFK;VVLDVGCGTGILSMFAAK | 17 | 0.3164 | Protein arginine N-methyltransferase 3 OS=Homo sapiens OX=9606 GN=PRMT3 PE=1 SV=4 |
| sp\|Q9P2E9\|RRBP1_HUMAN | 60.51864141 | 152364.6782 | DALNQATSQVESK;DAQDVQASQAEADQQQTR;EHTSHLEAELEK;ETSYEEALANQR;HPPAPAEPSSDLASK;IPDHDPAPNVTVLLR;LIEILSEK;LKELESQVSGLEK;LKGELESSDQVR;LLATEQEDAAVAK;QLLLESQSQLDAAK;QSDELALVR;QVLQLQASHR;SVEEEEQVWR;TLVSTVGSMVFNEGEAQR;TTQEQLAR;VEPAVSSVVNSIQVLTSK | 17 | 0.1546 | Ribosome-binding protein 1 OS=Homo sapiens OX=9606 GN=RRBP1 PE=1 SV=5 |
| sp\|Q14697\|GANAB_HUMAN | 57.89372566 | 106806.6457 | AEKDEPGAWEETFK;DAQHYGGWEHR;DVHNIYGLYVHMATADGLR;FGAVWTGDNTAEWDHLK;GLLEFEHQR;IDELEPR;KPGINVASDWSIHLR;LSFQHDPETSVLVLR;LVAIVDPHIK;QYASLTGTQALPPLFSLGYHQSR;REPWLLPSQHNDIIR;VLLVLELQGLQK;VSQGSKDPAEGDGAQPEETPR;VVIIGAGKPAAVVLQTK;WYQMGAYQPFFR;YRVPDVLVADPPIAR | 16 | 0.2458 | Neutral alpha-glucosidase AB OS=Homo sapiens OX=9606 GN=GANAB PE=1 SV=3 |
| sp\|Q8WUM4\|PDC6I_HUMAN | 52.19904411 | 95963.119 | ELPELLQR;ETVLSALSR;FGEEIAR;FTDLFEK;HYQFASGAFLHIK;KDNDFIYHDR;LALASLGYEK;LANQAADYFGDAFK;LLDEEEATDNDLR;NIQVSHQEFSK;QSNNEANLREEVLK;SLLSNLDEVKK;SVIEQGGIQTVDQLIK;TPSNELYKPLR;TSEVDLAKPLVK;YDEYVNVK | 16 | 0.2005 | Programmed cell death 6-interacting protein OS=Homo sapiens OX=9606 GN=PDCD6IP PE=1 SV=1 |
| sp\|P22314\|UBA1_HUMAN | 56.3288259 | 117774.3273 | AAVATFLQSVQVPEFTPK;AENYDIPSADR;ALPAVQQNNLDEDLIR;ATLPSPDKLPGFK;IHVSDQELQSANASVDDSRLEELK;KPLLESGTLGTK;LAGTQPLEVLEAVQR;LKSDTAAAAVR;LQTSSVLVSGLR;NEEDAAELVALAQAVNAR;NGSEADIDEGLYSR;QFLDYFK;QFLFRPWDVTK;QPAENVNQYLTDPK;YDGQVAVFGSDLQEK | 15 | 0.1994 | Ubiquitin-like modifier-activating enzyme 1 OS=Homo sapiens OX=9606 GN=UBA1 PE=1 SV=3 |
| sp\|P10809\|CH60_HUMAN | 55.45884426 | 61016.38048 | AAVEEGIVLGGGCALLR;CEFQDAYVLLSEK;GANPVEIR;GYISPYFINTSK;IGIEIIKR;ISSIQSIVPALEIANAHR;KISSIQSIVPALEIANAHR;LKVGLQVVAVK;LSDGVAVLK;LVQDVANNTNEEAGDGTTTATVLAR;NAGVEGSLIVEK;TLNDELEIIEGMK;TLNDELEIIEGMKFDR;VGGTSDVEVNEKK;VTDALNATR | 15 | 0.3002 | 60 kDa heat shock protein, mitochondrial OS=Homo sapiens OX=9606 GN=HSPD1 PE=1 SV=2 |
| sp\|P14618\|KPYM_HUMAN | 59.05397142 | 57900.02229 | CDENILWLDYK;DPVQEAWAEDVDLR;EAEAAIYHLQLFEELRR;GADFLVTEVENGGSLGSK;GDLGIEIPAEK;GDYPLEAVR;GSGTAEVELK;GVNLPGAAVDLPAVSEK;GVNLPGAAVDLPAVSEKDIQDLK;IYVDDGLISLQVK;LAPITSDPTEATAVGAVEASFK;LDIDSPPITAR;LNFSHGTHEYHAETIK;TATESFASDPILYRPVAVALDTK;TATESFASDPILYRPVAVALDTKGPEIR | 15 | 0.3823 | Pyruvate kinase PKM OS=Homo sapiens OX=9606 GN=PKM PE=1 SV=4 |
| sp\|P30101\|PDIA3_HUMAN | 45.86776566 | 56746.74644 | DGEEAGAYDGPR;EATNPPVIQEEKPK;ELSDFISYLQR;FLQDYFDGNLKR;GFPTIYFSPANK;GFPTIYFSPANKK;IFRDGEEAGAYDGPR;LAPEYEAAATR;LNFAVASR;QAGPASVPLR;SEPIPESNDGPVK;TADGIVSHLKK;VVVAENFDEIVNNENK;YGVSGYPTLK | 14 | 0.2851 | Protein disulfide-isomerase A3 OS=Homo sapiens OX=9606 GN=PDIA3 PE=1 SV=4 |
| sp\|P78371\|TCPB_HUMAN | 44.93855407 | 57452.12867 | AAHSEGNTTAGLDMR;AGADEERAETAR;EALLSSAVDHGSDEVK;GATQQILDEAER;GSGNLEAIHIIK;HGINCFINR;IHPQTIIAGWR;ILIANTGMDTDKIK;KIHPQTIIAGWR;LALVTGGEIASTFDHPELVK;LGGSLADSYLDEGFLLDKK;SLHDALCVLAQTVK;VAEIEHAEKEK | 13 | 0.3103 | T-complex protein 1 subunit beta OS=Homo sapiens OX=9606 GN=CCT2 PE=1 SV=4 |
| sp\|P13667\|PDIA4_HUMAN | 38.7316716 | 72886.97201 | AATQFWR;EFVTAFK;EVSQPDWTPPPEVTLVLTK;FDVSGYPTIK;FHHTFSTEIAK;GQAVDYEGSR;GRPYDYNGPR;IDATSASVLASR;KGQAVDYEGSR;MDATANDVPSDR;SPPIPLAK;YALPLVGHR;YGIVDYMIEQSGPPSK | 13 | 0.2047 | Protein disulfide-isomerase A4 OS=Homo sapiens OX=9606 GN=PDIA4 PE=1 SV=2 |
| sp\|P06733\|ENOA_HUMAN | 50.95922876 | 47139.3184 | AAVPSGASTGIYEALELR;AAVPSGASTGIYEALELRDNDK;DATNVGDEGGFAPNILENK;DATNVGDEGGFAPNILENKEGLELLK;GNPTVEVDLFTSK;HIADLAGNSEVILPVPAFNVINGGSHAGNK;IGAEVYHNLK;KLNVTEQEK;LAMQEFMILPVGAANFR;LAQANGWGVMVSHR;SGKYDLDFK;TIAPALVSK;YDLDFK;YDLDFKSPDDPSR;YISPDQLADLYK | 13 | 0.4101 | Alpha-enolase OS=Homo sapiens OX=9606 GN=ENO1 PE=1 SV=2 |
| sp\|P23526\|SAHH_HUMAN | 42.1842014 | 47685.20297 | ATDVMIAGK;GISEETTTGVHNLYK;IILLAEGR;KLDEAVAEAHLGK;LDEAVAEAHLGK;RIILLAEGR;VADIGLAAWGR;VAVVAGYGDVGK;VNIKPQVDR;VPAINVNDSVTK;YPQLLPGIR;YPVGVHFLPK;YSASKPLK | 13 | 0.2708 | Adenosylhomocysteinase OS=Homo sapiens OX=9606 GN=AHCY PE=1 SV=4 |
| sp\|P00367\|DHE3_HUMAN | 38.45497935 | 61359.19214 | CAVVDVPFGGAK;DDGSWEVIEGYR;ELEDFKLQHGSILGFPK;FTMELAK;HGGTIPIVPTAEFQDR;IIAEGANGPTTPEADKIFLER;IIKPCNHVLSLSFPIR;LQHGSILGFPK;MVEGFFDR;NLNHVSYGR;NYTDNELEK;YNLGLDLR;YSTDVSVDEVK | 13 | 0.2616 | Glutamate dehydrogenase 1, mitochondrial OS=Homo sapiens OX=9606 GN=GLUD1 PE=1 SV=2 |
| sp\|P60174\|TPIS_HUMAN | 47.42924962 | 30771.69549 | DCGATWVVLGHSER;FFVGGNWK;HVFGESDELIGQK;IIYGGSVTGATCK;KFFVGGNWK;KQSLGELIGTLNAAK;QSLGELIGTLNAAK;RHVFGESDELIGQK;SNVSDAVAQSTR;TATPQQAQEVHEK;VIADNVK;VIADNVKDWSK;VVLAYEPVWAIGTGK | 13 | 0.4056 | Triosephosphate isomerase OS=Homo sapiens OX=9606 GN=TPI1 PE=1 SV=3 |
| sp\|P09651\|ROA1_HUMAN | 47.02459937 | 38723.04917 | DYFEQYGK;EDSQRPGAHLTVK;EDTEEHHLRDYFEQYGK;GFAFVTFDDHDSVDK;GFAFVTFDDHDSVDKIVIQK;GFGFVTYATVEEVDAAMNARPHK;IEVIEIMTDR;IFVGGIKEDTEEHHLR;KLFIGGLSFETTDESLR;NQGGYGGSSSSSSYGSGR;SESPKEPEQLR;SSGPYGGGGQYFAKPR;YHTVNGHNCEVR | 13 | 0.4435 | Heterogeneous nuclear ribonucleoprotein A1 OS=Homo sapiens OX=9606 GN=HNRNPA1 PE=1 SV=5 |
| sp\|P18206\|VINC_HUMAN | 36.44733012 | 123721.8103 | ALASIDSK;AQQVSQGLDVLTAK;AVAGNISDPGLQK;DYLIDGSR;EAEAASIK;ELTPQVVSAAR;IFVTTK;IPTISTQLK;LLAVAATAPPDAPNREEVFDER;STVEGIQASVK;VDQLTAQLADLAAR;VLQLTSWDEDAWASK | 12 | 0.1226 | Vinculin OS=Homo sapiens OX=9606 GN=VCL PE=1 SV=4 |
| sp\|P27816\|MAP4_HUMAN | 38.10825867 | 120929.8239 | ALPLEAEVAPVK;DVKPKPIADAK;HVPGGGNVQIQNK;QPAPTTIGGLNK;TAGPIASAQK;TDYIPLLDVDEK;TEAPLAK;TTTAAAVASTGPSSR;TTTLSGTAPAAGVVPSR;VALSSETEVALAR;VGSLDNVGHLPAGGAVK;WPTETDVSSAK | 12 | 0.1302 | Microtubule-associated protein 4 OS=Homo sapiens OX=9606 GN=MAP4 PE=1 SV=3 |
| sp\|P54577\|SYYC_HUMAN | 35.16660844 | 59106.09762 | APWELLELR;DFAAEVVHPGDLK;HVLFPLK;IDVGEAEPR;LSSVVTQHDSK;NSEPEEVIPSR;NSVEVALNK;SEFVILR;SEFVILRDEK;TVVSGLVQFVPK;VDAQFGGIDQR;YLPALGYSK | 12 | 0.2102 | Tyrosine--tRNA ligase, cytoplasmic OS=Homo sapiens OX=9606 GN=YARS1 PE=1 SV=4 |
| sp\|P09874\|PARP1_HUMAN | 43.44911481 | 113012.3927 | AQNDLIWNIKDELK;GGAAVDPDSGLEHSAHVLEK;GGSDDSSKDPIDVNYEK;KGDEVDGVDEVAK;KPPLLNNADSVQAK;LQLLEDDKENR;NREELGFRPEYSASQLK;NTHATTHNAYDLEVIDIFK;QQVPSGESAILDR;TTNFAGILSQGLR;VVDRDSEEAEIIR;VVSEDFLQDVSASTK | 12 | 0.1765 | Poly [ADP-ribose] polymerase 1 OS=Homo sapiens OX=9606 GN=PARP1 PE=1 SV=4 |
| sp\|P62701\|RS4X_HUMAN | 37.65880963 | 29579.05326 | DANGNSFATR;ERHPGSFDVVHVK;GIPHLVTHDAR;GNKPWISLPR;HPGSFDVVHVK;ITPEEAK;KIFVGTK;LRECLPLIIFLR;VNDTIQIDLETGK;YALTGDEVK;YALTGDEVKK;YPDPLIK | 12 | 0.3802 | 40S ribosomal protein S4, X isoform OS=Homo sapiens OX=9606 GN=RPS4X PE=1 SV=2 |
| sp\|P04075\|ALDOA_HUMAN | 38.61283978 | 39395.30522 | AAQEEYVKR;ALQASALK;FSHEEIAMATVTALR;GVVPLAGTNGETTTQGLDGLSER;IGEHTPSALAIMENANVLAR;KELSDIAHR;LQSIGTENTEENR;LQSIGTENTEENRR;PYQYPALTPEQK;QLLLTADDR;YTPSGQAGAAASESLFVSNHAY | 11 | 0.4066 | Fructose-bisphosphate aldolase A OS=Homo sapiens OX=9606 GN=ALDOA PE=1 SV=2 |
| sp\|P08758\|ANXA5_HUMAN | 34.1977387 | 35914.40033 | ADAETLRK;FITIFGTR;GAGTDDHTLIR;GTVTDFPGFDER;LIVALMKPSR;LYDAYELK;MLVVLLQANR;NFATSLYSMIK;SEIDLFNIR;SEIDLFNIRK;WGTDEEKFITIFGTR | 11 | 0.2969 | Annexin A5 OS=Homo sapiens OX=9606 GN=ANXA5 PE=1 SV=2 |
| sp\|P23396\|RS3_HUMAN | 31.47488472 | 26671.42974 | AELNEFLTR;DEILPTTPISEQK;ELAEDGYSGVEVR;ELTAVVQK;FVADGIFK;GCEVVVSGK;GGKPEPPAMPQPVPTA;GLCAIAQAESLR;KFVADGIFK;QGVLGIK;TQNVLGEK | 11 | 0.428 | 40S ribosomal protein S3 OS=Homo sapiens OX=9606 GN=RPS3 PE=1 SV=2 |
| sp\|Q92945\|FUBP2_HUMAN | 38.06718021 | 73070.02259 | AINQQTGAFVEISR;DAFADAVQR;GGGGPGGGGPGGGSAGGPSQPPGGGGPGIR;HSVGVVIGR;IGGDAATTVNNSTPDFGFGGQK;IGQQPQQPGAPPQQDYTK;IINDLLQSLR;MILIQDGSQNTNVDKPLR;QLEDGDQPESK;SVSLTGAPESVQK;VQISPDSGGLPER | 11 | 0.2461 | Far upstream element-binding protein 2 OS=Homo sapiens OX=9606 GN=KHSRP PE=1 SV=4 |
| sp\|P27797\|CALR_HUMAN | 38.32738239 | 48111.82369 | AKIDDPTDSKPEDWDKPEHIPDPDAK;EQFLDGDGWTSR;FYALSASFEPFSNK;FYGDEEKDK;GQTLVVQFTVK;HEQNIDCGGGYVK;IDDPTDSKPEDWDKPEHIPDPDAK;IDNSQVESGSLEDDWDFLPPKK;IKDPDASKPEDWDER;KVHVIFNYK;VHVIFNYK | 11 | 0.3141 | Calreticulin OS=Homo sapiens OX=9606 GN=CALR PE=1 SV=1 |
| sp\|P26640\|SYVC_HUMAN | 29.25656889 | 140387.3714 | ALSPLEEWLR;AVLVALK;DVVEPLLRPQWYVR;GALINVPPPFLGLPR;GFVPSPTSQPGGHESLVDR;KAVLVALK;KVDEAIALFQK;LHEEGIIYR;NVIHPFLSR;QLWWGHR;SSAQDPQAVLGALGR | 11 | 0.0926 | Valine--tRNA ligase OS=Homo sapiens OX=9606 GN=VARS1 PE=1 SV=4 |
| sp\|P11498\|PYC_HUMAN | 35.73125314 | 129551.4089 | ASPSPTDPVVPAVPIGPPPAGFR;EGPEGFAR;ENNVDAVHPGYGFLSER;FIGPSPEVVR;GANAVGYTNYPDNVVFK;GLAPVQAYLHIPDIIK;LFLQGPK;QKADEAYLIGR;VFDYSEYWEGAR;VVEIAPAAHLDPQLR;VVHSYEELEENYTR | 11 | 0.1273 | Pyruvate carboxylase, mitochondrial OS=Homo sapiens OX=9606 GN=PC PE=1 SV=2 |
| sp\|P51659\|DHB4_HUMAN | 38.4658986 | 79636.304 | ATSTATSGFAGAIGQK;AVANYDSVEEGEK;AYALAFAER;GALVVVNDLGGDFK;GSLAADKVVEEIR;IDSEGGVSANHTSR;IDVVVNNAGILR;ISDEDWDIIHR;LGLLGLANSLAIEGR;VLHGEQYLELYKPLPR;VVLVTGAGAGLGR | 11 | 0.1984 | Peroxisomal multifunctional enzyme type 2 OS=Homo sapiens OX=9606 GN=HSD17B4 PE=1 SV=3 |
| sp\|P63244\|RACK1_HUMAN | 33.23282451 | 35054.55748 | DETNYGIPQR;DVLSVAFSSDNR;FSPNSSNPIIVSCGWDK;IIVDELK;IIVDELKQEVISTSSK;IWDLEGK;LTRDETNYGIPQR;LWDLTTGTTTR;QEVISTSSK;VWQVTIGTR;YWLCAATGPSIK | 11 | 0.306 | Receptor of activated protein C kinase 1 OS=Homo sapiens OX=9606 GN=RACK1 PE=1 SV=3 |
| sp\|P34897\|GLYM_HUMAN | 29.94084406 | 55957.72944 | AHLLADMAHISGLVAAK;AMADALLER;EIPYTFEDR;HADIVTTTTHK;ISATSIFFESMPYK;SGLIFYR;TGLIDYNQLALTAR;VLELVSITANK;YSEGYPGK;YSEGYPGKR | 10 | 0.2004 | Serine hydroxymethyltransferase, mitochondrial OS=Homo sapiens OX=9606 GN=SHMT2 PE=1 SV=3 |
| sp\|Q9Y617\|SERC_HUMAN | 31.15871825 | 40396.81021 | ALELNMLSLK;ASLYNAVTIEDVQK;DDLLGFALR;FGTINIVHPK;GVGISVLEMSHR;IINNTENLVR;LPHSVLLEIQK;MNIPFR;NVGSAGVTVVIVR;QVVNFGPGPAK | 10 | 0.2865 | Phosphoserine aminotransferase OS=Homo sapiens OX=9606 GN=PSAT1 PE=1 SV=2 |
| sp\|P06744\|G6PI_HUMAN | 32.48429236 | 63107.24252 | AVLHVALR;INYTEGR;MLVDLAK;SNTPILVDGK;SPEDLER;TFTTQETITNAETAK;TLAQLNPESSLFIIASK;VDHQTGPIVWGEPGTNGQHAFYQLIHQGTK;VFEGNRPTNSIVFTK;VWYVSNIDGTHIAK | 10 | 0.233 | Glucose-6-phosphate isomerase OS=Homo sapiens OX=9606 GN=GPI PE=1 SV=4 |
| sp\|P04792\|HSPB1_HUMAN | 33.18200173 | 22768.49251 | DGVVEITGK;DGVVEITGKHEER;DWYPHSR;GPSWDPFRDWYPHSR;HEERQDEHGYISR;LATQSNEITIPVTFESR;LFDQAFGLPR;QDEHGYISR;TKDGVVEITGK;VSLDVNHFAPDELTVK | 10 | 0.4 | Heat shock protein beta-1 OS=Homo sapiens OX=9606 GN=HSPB1 PE=1 SV=2 |
| sp\|Q05682\|CALD1_HUMAN | 32.43005981 | 93175.42504 | EAEGAPQVEAGK;EAEGAPQVEAGKR;GNVFSSPTAAGTPNK;LEQYTSAIEGTK;QKEFDPTITDASLSLPSR;QQEAALELEELKK;SAKPTKPAASDLPVPAEGVR;STHQAAIVSK;TTTTNTQVEGDDEAAFLER;YEIEETETVTK | 10 | 0.1652 | Caldesmon OS=Homo sapiens OX=9606 GN=CALD1 PE=1 SV=3 |
| sp\|Q15008\|PSMD6_HUMAN | 31.58747279 | 45502.27266 | EGALTAFR;FLLSLPEHR;GAEILEVLHSLPAVR;IDKVNEIVETNRPDSK;IHAYSQLLESYR;KDWLFAPHYR;KGDLLLNR;LDEELEDAEK;RLDEELEDAEK;VNEIVETNRPDSK | 10 | 0.2288 | 26S proteasome non-ATPase regulatory subunit 6 OS=Homo sapiens OX=9606 GN=PSMD6 PE=1 SV=1 |
| sp\|P30041\|PRDX6_HUMAN | 33.8065981 | 25019.18976 | ELAILLGMLDPAEKDEK;FHDFLGDSWGILFSHPR;LAPEFAK;LIALSIDSVEDHLAWSK;LPFPIIDDR;LPFPIIDDRNR;LSILYPATTGR;VVFVFGPDK;VVFVFGPDKK;VVISLQLTAEKR | 10 | 0.4554 | Peroxiredoxin-6 OS=Homo sapiens OX=9606 GN=PRDX6 PE=1 SV=3 |
| sp\|Q13263\|TIF1B_HUMAN | 35.04534964 | 88493.41747 | FQWDLNAWTK;IVAERPGTNSTGPAPMAPPR;KLIYFQLHR;LDLDLTADSQPPVFK;LIYFQLHR;LSPPYSSPQEFAQDVGR;LTEDKADVQSIIGLQR;MAILQIMK;SGEGEVSGLMR;VLVNDAQK | 10 | 0.1365 | Transcription intermediary factor 1-beta OS=Homo sapiens OX=9606 GN=TRIM28 PE=1 SV=5 |
| sp\|P31948\|STIP1_HUMAN | 29.41197098 | 62599.40476 | AAALEFLNR;DPQALSEHLKNPVIAQK;KAAALEFLNR;LAYINPDLALEEK;LDPHNHVLYSNR;LMDVGLIAIR;TVDLKPDWGK;TYEEGLKHEANNPQLK;YKDAIHFYNK | 9 | 0.1805 | Stress-induced-phosphoprotein 1 OS=Homo sapiens OX=9606 GN=STIP1 PE=1 SV=1 |
| sp\|Q15084\|PDIA6_HUMAN | 29.5497005 | 48091.26107 | AATALKDVVK;DVIELTDDSFDK;GESPVDYDGGR;GSFSEQGINEFLR;GSTAPVGGGAFPTIVER;GSTAPVGGGAFPTIVEREPWDGR;LAAVDATVNQVLASR;NLEPEWAAAASEVK;NSYLEVLLK | 9 | 0.2432 | Protein disulfide-isomerase A6 OS=Homo sapiens OX=9606 GN=PDIA6 PE=1 SV=1 |
| sp\|P51812\|KS6A3_HUMAN | 29.68605306 | 83682.94442 | ADPSQFELLK;DILVEVNHPFIVK;EASAVLFTITK;FSLSGGYWNSVSDTAK;HPWIVHWDQLPQYQLNR;HSFFSTIDWNK;LGAGPDGVEEIK;LHYAFQTEGK;TVEYLHAQGVVHR | 9 | 0.1527 | Ribosomal protein S6 kinase alpha-3 OS=Homo sapiens OX=9606 GN=RPS6KA3 PE=1 SV=1 |
| sp\|P27824\|CALX_HUMAN | 29.84058929 | 67525.84889 | AEEDEILNR;APVPTGEVYFADSFDR;GTLSGWILSK;IVDDWANDGWGLK;KIPNPDFFEDLEPFR;KPEDWDERPK;RPDADLK;TPELNLDQFHDK;TYFTDKK | 9 | 0.1672 | Calnexin OS=Homo sapiens OX=9606 GN=CANX PE=1 SV=2 |
| sp\|P31939\|PUR9_HUMAN | 24.54179019 | 64575.34086 | AFTHTAQYDEAISDYFR;ALFEEVPELLTEAEKK;ANYWWLR;EALGIPAAASFK;LDFNLIR;NGQVIGIGAGQQSR;TGLVEFAR;TLFGLHLSQK;TLHPAVHAGILAR | 9 | 0.1757 | Bifunctional purine biosynthesis protein ATIC OS=Homo sapiens OX=9606 GN=ATIC PE=1 SV=3 |
| sp\|P15880\|RS2_HUMAN | 27.39266271 | 31304.59455 | AFVAIGDYNGHVGLGVK;ATFDAISK;CGSVLVR;GCTATLGNFAK;GTGIVSAPVPK;IGKPHTVPCK;LSIVPVRR;SPYQEFTDHLVK;TYSYLTPDLWK | 9 | 0.3242 | 40S ribosomal protein S2 OS=Homo sapiens OX=9606 GN=RPS2 PE=1 SV=2 |
| sp\|O43143\|DHX15_HUMAN | 29.86058174 | 90875.16434 | ALVTGYFMQVAHLER;EVDDLGPEVGDIK;FAHIDGDHLTLLNVYHAFK;IRVESLLVTAISK;LQLPVWEYK;LQLPVWEYKDR;SNLGSVVLQLK;TLATDILMGVLK;YGVIILDEAHER | 9 | 0.1333 | Pre-mRNA-splicing factor ATP-dependent RNA helicase DHX15 OS=Homo sapiens OX=9606 GN=DHX15 PE=1 SV=2 |
| sp\|O75874\|IDHC_HUMAN | 28.52180465 | 46629.5137 | ATDFVVPGPGK;DATNDQVTK;FKDIFQEIYDK;GWPLYLSTK;HAYGDQYR;LVSGWVKPIIIGR;SDYLNTFEFMDK;TVEAEAAHGTVTR;VEITYTPSDGTQK | 9 | 0.2391 | Isocitrate dehydrogenase [NADP] cytoplasmic OS=Homo sapiens OX=9606 GN=IDH1 PE=1 SV=2 |
| sp\|P49588\|SYAC_HUMAN | 31.73240085 | 106743.2225 | AVFDETYPDPVR;FDFTAK;GGYVLHIGTIYGDLK;GLEVTDDSPK;IVAVTGAEAQK;NSSHAGAFVIVTEEAIAK;QIWQNLGLDDTK;VDDSSEDKTEFTVK;VGDQVWLFIDEPR | 9 | 0.1147 | Alanine--tRNA ligase, cytoplasmic OS=Homo sapiens OX=9606 GN=AARS1 PE=1 SV=2 |
| sp\|O14980\|XPO1_HUMAN | 25.72102303 | 123306.0943 | AVGHPFVIQLGR;DFLVQIK;EPEVLSTMAIIVNK;LISTLIYK;LNMILVQILK;NVDILKDPETVK;QLLDFSQK;SAFPHLQDAQVK;YVVGLIIK | 9 | 0.085 | Exportin-1 OS=Homo sapiens OX=9606 GN=XPO1 PE=1 SV=1 |
| sp\|Q14566\|MCM6_HUMAN | 31.16027987 | 92831.23576 | DEEQTAESIK;ELRDEEQTAESIK;ESEDFIVEQYK;IQETQAELPR;ISNLIVLHLR;KVEEEEDESALK;SELVNWYLK;VSGVDGYETEGIR;VYSLDDIRR | 9 | 0.106 | DNA replication licensing factor MCM6 OS=Homo sapiens OX=9606 GN=MCM6 PE=1 SV=1 |
| sp\|P00338\|LDHA_HUMAN | 28.73634088 | 36665.36034 | DQLIYNLLK;DQLIYNLLKEEQTPQNK;FIIPNVVK;IVSGKDYNVTANSK;QVVESAYEVIK;SADTLWGIQK;TLHPDLGTDKDK;VHPVSTMIK;VIGSGCNLDSAR | 9 | 0.2801 | L-lactate dehydrogenase A chain OS=Homo sapiens OX=9606 GN=LDHA PE=1 SV=2 |
| sp\|P61247\|RS3A_HUMAN | 26.2864577 | 29925.73881 | DWYDVK;EVQTNDLKEVVNK;KDWYDVK;LFCVGFTK;LIPDSIGKDIEK;LITEDVQGK;LMELHGEGSSSGK;TTDGYLLR;VVDPFSK | 9 | 0.2917 | 40S ribosomal protein S3a OS=Homo sapiens OX=9606 GN=RPS3A PE=1 SV=2 |
| sp\|P22626\|ROA2_HUMAN | 31.010596 | 37406.72611 | EESGKPGAHVTVK;GFGFVTFDDHDPVDK;GFGFVTFDDHDPVDKIVLQK;GGGGNFGPGPGSNFR;IDTIEIITDR;NYYEQWGK;QEMQEVQSSR;YHTINGHNAEVR;YHTINGHNAEVRK | 9 | 0.2521 | Heterogeneous nuclear ribonucleoproteins A2/B1 OS=Homo sapiens OX=9606 GN=HNRNPA2B1 PE=1 SV=2 |
| sp\|P47897\|SYQ_HUMAN | 27.90781568 | 87742.87197 | FDDTNPEKEEAK;FSEGEATLR;HRPQLLVER;ILQLVATGAVR;LAWGQPVGLR;LFTLTALR;LNLHYAVVSK;QHLEITGGQVR;TDFKEEPEPGFK | 9 | 0.1187 | Glutamine--tRNA ligase OS=Homo sapiens OX=9606 GN=QARS1 PE=1 SV=1 |
| sp\|Q92614\|MY18A_HUMAN | 30.99837169 | 232971.0277 | IISNLFLGR;ISELTSELTDER;LVEINGHNVESK;NTGESASQLLDAETAER;QGPEESGLGDGTGPK;QNPATQNAPR;SEELNLPEGK;TFVQELER;YGASLLHTYAGPSLLVLGPR | 9 | 0.055 | Unconventional myosin-XVIIIa OS=Homo sapiens OX=9606 GN=MYO18A PE=1 SV=3 |
| sp\|P09211\|GSTP1_HUMAN | 30.68529896 | 23341.02235 | AFLASPEYVNLPINGNGK;AFLASPEYVNLPINGNGKQ;ALPGQLKPFETLLSQNQGGK;ASCLYGQLPK;EEVVTVETWQEGSLK;FQDGDLTLYQSNTILR;PPYTVVYFPVR;YISLIYTNYEAGKDDYVK | 8 | 0.519 | Glutathione S-transferase P OS=Homo sapiens OX=9606 GN=GSTP1 PE=1 SV=2 |
| sp\|P62269\|RS18_HUMAN | 25.52527491 | 17707.85986 | AGELTEDEVER;FQHILR;IAFAITAIK;IPDWFLNR;KADIDLTK;KIAFAITAIK;RAGELTEDEVER;YSQVLANGLDNK | 8 | 0.3684 | 40S ribosomal protein S18 OS=Homo sapiens OX=9606 GN=RPS18 PE=1 SV=3 |
| sp\|P15559\|NQO1_HUMAN | 25.70201775 | 30848.00765 | ALIVLAHSER;EAAAAALK;EGHLSPDIVAEQK;FGLSVGHHLGK;IQILEGWK;IQILEGWKK;LKDPANFQYPAESVLAYK;RALIVLAHSER | 8 | 0.2555 | NAD(P)H dehydrogenase [quinone] 1 OS=Homo sapiens OX=9606 GN=NQO1 PE=1 SV=1 |
| sp\|O60664\|PLIN3_HUMAN | 28.22985528 | 47045.94516 | ALTMFR;DTVATQLSEAVDATR;LEPQIASASEYAHR;LGQMVLSGVDTVLGK;LHQMWLSWNQK;QEQSYFVR;QLQGPEKEPPKPEQVESR;SEEWADNHLPLTDAELAR | 8 | 0.2419 | Perilipin-3 OS=Homo sapiens OX=9606 GN=PLIN3 PE=1 SV=3 |
| sp\|P28074\|PSB5_HUMAN | 26.27899183 | 28462.2092 | ATAGAYIASQTVK;DAYSGGAVNLYHVR;GPGLYYVDSEGNR;HGVIVAADSR;ISVAAASK;LLANMVYQYK;RAIYQATYR;RGPGLYYVDSEGNR | 8 | 0.2966 | Proteasome subunit beta type-5 OS=Homo sapiens OX=9606 GN=PSMB5 PE=1 SV=3 |
| sp\|P50552\|VASP_HUMAN | 29.66227813 | 39805.08806 | DESANQEEPEAR;QQPGPSEHIER;QVWGLNFGSK;TPKDESANQEEPEAR;VKEEIIEAFVQELR;VPAQSESVR;WLPAGTGPQAFSR;YNQATPNFHQWR | 8 | 0.2211 | Vasodilator-stimulated phosphoprotein OS=Homo sapiens OX=9606 GN=VASP PE=1 SV=3 |
| sp\|P53396\|ACLY_HUMAN | 22.88618842 | 120761.8788 | DGVYVLDLAAK;DYQGPLKEHEVTIFVR;EAGVFVPR;EAYPEEAYIADLDAK;FICTTSAIQNR;GGPNYQEGLR;LYRPGSVAYVSR;SGGMSNELNNIISR | 8 | 0.0881 | ATP-citrate synthase OS=Homo sapiens OX=9606 GN=ACLY PE=1 SV=3 |
| sp\|P50395\|GDIB_HUMAN | 24.35333618 | 50630.87681 | DLGTESQIFISR;DWNVDLIPK;EIRPALELLEPIEQK;LYSESLAR;MLLYTEVTR;SPYLYPLYGLGELPQGFAR;TFEGIDPK;VICILSHPIK | 8 | 0.2022 | Rab GDP dissociation inhibitor beta OS=Homo sapiens OX=9606 GN=GDI2 PE=1 SV=2 |
| sp\|Q99460\|PSMD1_HUMAN | 20.04691463 | 105768.6928 | DNLEWLAR;FTATASLGVIHK;LLHVAVSDVNDDVR;LLHVAVSDVNDDVRR;QAIGIALETR;QFAALVASK;TPEASPEPK;VSTAVLSITAK | 8 | 0.0776 | 26S proteasome non-ATPase regulatory subunit 1 OS=Homo sapiens OX=9606 GN=PSMD1 PE=1 SV=2 |
| sp\|P07737\|PROF1_HUMAN | 27.96614299 | 15044.55581 | DSLLQDGEFSMDLR;EGVHGGLINK;EGVHGGLINKK;SSFYVNGLTLGGQK;STGGAPTFNVTVTK;TDKTLVLLMGK;TFVNITPAEVGVLVGKDR;TLVLLMGK | 8 | 0.5857 | Profilin-1 OS=Homo sapiens OX=9606 GN=PFN1 PE=1 SV=2 |
| sp\|P26373\|RL13_HUMAN | 24.8793412 | 24246.5256 | EAAEQDVEK;GFSLEELR;KGDSSAEELK;LATQLTGPVMPVR;STESLQANVQR;VATWFNQPAR;VITEEEK;VITEEEKNFK | 8 | 0.3365 | 60S ribosomal protein L13 OS=Homo sapiens OX=9606 GN=RPL13 PE=1 SV=4 |
| sp\|P18124\|RL7_HUMAN | 25.43802943 | 29207.1972 | EANNFLWPFK;FKEANNFLWPFK;IALTDNALIAR;KAGNFYVPAEPK;QIFNGTFVK;SVNELIYKR;TTHFVEGGDAGNR;TTHFVEGGDAGNREDQINR | 8 | 0.2903 | 60S ribosomal protein L7 OS=Homo sapiens OX=9606 GN=RPL7 PE=1 SV=1 |
| sp\|P78417\|GSTO1_HUMAN | 23.78149669 | 27548.03764 | EDPTVSALLTSEK;GSAPPGPVPEGSIR;HEVININLK;LEEVLTNK;LLPDDPYEK;MILELFSK;NKPEWFFK;VPSLVGSFIR | 8 | 0.3278 | Glutathione S-transferase omega-1 OS=Homo sapiens OX=9606 GN=GSTO1 PE=1 SV=2 |
| sp\|Q16851\|UGPA_HUMAN | 22.61440126 | 56904.71899 | EFPTVPLVK;ESLLPVAK;FVQDLSK;GGTLTQYEGK;GTVIIIANHGDR;ILTTASSHEFEHTK;IQRPPEDSIQPYEK;LVEIAQVPK | 8 | 0.1634 | UTP--glucose-1-phosphate uridylyltransferase OS=Homo sapiens OX=9606 GN=UGP2 PE=1 SV=5 |
| sp\|P41250\|GARS_HUMAN | 25.25043568 | 83112.609 | ELALQPK;GEFTIETEGK;NNIIQTWR;TFFSFPAVVAPFK;TVNVVQFEPSK;VDDSSGSIGR;VPLVAEKPLK;YPLFEGQETGKK | 8 | 0.1096 | Glycine--tRNA ligase OS=Homo sapiens OX=9606 GN=GARS1 PE=1 SV=3 |
| sp\|P48735\|IDHP_HUMAN | 30.09159462 | 50876.86267 | FKDIFQEIFDK;GKLDGNQDLIR;GRPTSTNPIASIFAWTR;IIWQFIK;LILPHVDIQLK;LNEHFLNTTDFLDTIK;LVPGWTKPITIGR;TIEAEAAHGTVTR | 8 | 0.219 | Isocitrate dehydrogenase [NADP], mitochondrial OS=Homo sapiens OX=9606 GN=IDH2 PE=1 SV=2 |
| sp\|O15027\|SC16A_HUMAN | 26.47820935 | 251737.5101 | LVLIGSNHSLPFLK;NGLWGHALLLASK;NPSSAAPVQSR;QALQSTPLGSSSK;QSGPGAPNLDR;SVFWASSPYR;YGPLPGPAVPR;YRPYDGAASAYAQNYR | 8 | 0.042 | Protein transport protein Sec16A OS=Homo sapiens OX=9606 GN=SEC16A PE=1 SV=4 |
| sp\|P29966\|MARCS_HUMAN | 31.51957609 | 31535.86388 | AAEEPSKVEEK;AEDGATPSPSNETPK;EAGEGGEAEAPAAEGGK;EELQANGSAPAADKEEPAAAGSGAASPSAAEK;GEAAAERPGEAAVASSPSK;GEPAAAAAPEAGASPVEK;VNGDASPAAAESGAK | 7 | 0.3825 | Myristoylated alanine-rich C-kinase substrate OS=Homo sapiens OX=9606 GN=MARCKS PE=1 SV=4 |
| sp\|Q9NR30\|DDX21_HUMAN | 23.39427423 | 87290.37907 | AAVIGDVIR;APQVLVLAPTR;EGAFSNFPISEETIK;GVTFLFPIQAK;QDAQSLHGDIPQK;TAITVEHLAIK;TFSFAIPLIEK | 7 | 0.1034 | Nucleolar RNA helicase 2 OS=Homo sapiens OX=9606 GN=DDX21 PE=1 SV=5 |
| sp\|Q06830\|PRDX1_HUMAN | 22.50322504 | 22096.27844 | ADEGISFR;ATAVMPDGQFK;HGEVCPAGWKPGSDTIKPDVQK;IGHPAPNFK;LVQAFQFTDK;QGGLGPMNIPLVSDPK;TIAQDYGVLK | 7 | 0.5477 | Peroxiredoxin-1 OS=Homo sapiens OX=9606 GN=PRDX1 PE=1 SV=1 |
| sp\|P08195\|4F2_HUMAN | 22.88032197 | 67951.72611 | ADLLLSTQPGREEGSPLELER;EDFDSLLQSAK;EEGSPLELER;GQSEDPGSLLSLFR;LKLEPHEGLLLR;VAEDEAEAAAAAK;WWHTGALYR | 7 | 0.127 | 4F2 cell-surface antigen heavy chain OS=Homo sapiens OX=9606 GN=SLC3A2 PE=1 SV=3 |
| sp\|Q86VP6\|CAND1_HUMAN | 25.09804721 | 136288.6576 | ADVFHAYLSLLK;ALTLIAGSPLK;AVAALLTIPEAEK;ITSEALLVTQQLVK;LGTLSALDILIK;RQYLLLHSLK;VALVTFNSAAHNKPSLIR | 7 | 0.0732 | Cullin-associated NEDD8-dissociated protein 1 OS=Homo sapiens OX=9606 GN=CAND1 PE=1 SV=2 |
| sp\|P67936\|TPM4_HUMAN | 22.69909289 | 28504.49049 | AEGDVAALNR;AEGDVAALNRR;EKAEGDVAALNR;IQALQQQADEAEDR;KIQALQQQADEAEDR;KLVILEGELER;TIDDLEEK | 7 | 0.3105 | Tropomyosin alpha-4 chain OS=Homo sapiens OX=9606 GN=TPM4 PE=1 SV=3 |
| sp\|P02786\|TFR1_HUMAN | 24.09829409 | 84817.94215 | AFTYINLDK;HVFWGSGSHTLPALLENLK;LAVDEEENADNNTK;LLNENSYVPR;LTVSNVLK;VEYHFLSPYVSPK;VSASPLLYTLIEK | 7 | 0.1132 | Transferrin receptor protein 1 OS=Homo sapiens OX=9606 GN=TFRC PE=1 SV=2 |
| sp\|O14818\|PSA7_HUMAN | 25.04506196 | 27869.61416 | AITVFSPDGHLFQVEYAQEAVK;AITVFSPDGHLFQVEYAQEAVKK;ALLEVVQSGGK;DIVVLGVEK;GRDIVVLGVEK;LTVEDPVTVEYITR;LYQTDPSGTYHAWK | 7 | 0.2944 | Proteasome subunit alpha type-7 OS=Homo sapiens OX=9606 GN=PSMA7 PE=1 SV=1 |
| sp\|Q04637\|IF4G1_HUMAN | 20.5987991 | 175382.2626 | ALPSEELNR;EAVGDLLDAFK;EFLPEGQDIGAFVAEQK;GVIDLIFEK;ITKPGSIDSNNQLFAPGGR;SVTAFFK;VPTTEKPTVTVNFR | 7 | 0.0538 | Eukaryotic translation initiation factor 4 gamma 1 OS=Homo sapiens OX=9606 GN=EIF4G1 PE=1 SV=4 |
| sp\|Q99873\|ANM1_HUMAN | 23.92053585 | 42433.93942 | ANKLDHVVTIIK;ATLYVTAIEDR;DKWLAPDGLIFPDR;DVAIKEPLVDVVDPK;EVDIYTVK;GKVEEVELPVEK;TGFSTSPESPYTHWK | 7 | 0.2345 | Protein arginine N-methyltransferase 1 OS=Homo sapiens OX=9606 GN=PRMT1 PE=1 SV=3 |
| sp\|P15311\|EZRI_HUMAN | 21.56211861 | 69369.73669 | AQEEAERLEADR;EVWYFGLHYVDNK;GFPTWLK;IALLEEAR;IQVWHAEHR;QLLTLSSELSQAR;SQEQLAAELAEYTAK | 7 | 0.1911 | Ezrin OS=Homo sapiens OX=9606 GN=EZR PE=1 SV=4 |
| sp\|P25786\|PSA1_HUMAN | 22.0005375 | 29536.86021 | AQPAQPADEPAEK;ETLPAEQDLTTK;LVSLIGSK;NQYDNDVTVWSPQGR;NVSIGIVGK;THAVLVALK;THAVLVALKR | 7 | 0.2548 | Proteasome subunit alpha type-1 OS=Homo sapiens OX=9606 GN=PSMA1 PE=1 SV=1 |
| sp\|O15144\|ARPC2_HUMAN | 22.84756469 | 34311.48465 | ASHTAPQVLFSHR;DNTINLIHTFR;DYLHYHIK;ELQAHGADELLK;IIEETLALK;YFQFQEEGK;YFQFQEEGKEGENR | 7 | 0.2233 | Actin-related protein 2/3 complex subunit 2 OS=Homo sapiens OX=9606 GN=ARPC2 PE=1 SV=1 |
| sp\|P51149\|RAB7A_HUMAN | 25.02622614 | 23474.84091 | ATIGADFLTK;DPENFPFVVLGNK;EAINVEQAFQTIAR;FQSLGVAFYR;LVTMQIWDTAGQER;NNIPYFETSAK;TLDSWRDEFLIQASPR | 7 | 0.4251 | Ras-related protein Rab-7a OS=Homo sapiens OX=9606 GN=RAB7A PE=1 SV=1 |
| sp\|O94979\|SC31A_HUMAN | 19.18363086 | 132931.0062 | ATVWDLR;NPAVLSAASFDGR;NVWSFLK;RLEFLYDK;RPVGASFSFGGK;TTFEDLIQR;YLELLGYR | 7 | 0.0525 | Protein transport protein Sec31A OS=Homo sapiens OX=9606 GN=SEC31A PE=1 SV=3 |
| sp\|P55786\|PSA_HUMAN | 22.25342522 | 103210.7272 | DAESIHQYLLQR;IDFVGELNDK;LGLQNDLFSLAR;LNLGTVGFYR;SPVYLTVLK;VLGATLLPDLIQK;YAAVTQFEATDAR | 7 | 0.086 | Puromycin-sensitive aminopeptidase OS=Homo sapiens OX=9606 GN=NPEPPS PE=1 SV=2 |
| sp\|P38117\|ETFB_HUMAN | 22.89613437 | 27826.15229 | EIDGGLETLR;EKVDLVLLGK;KIEVIKPGDLGVDLTSK;LGPLQVAR;LPAVVTADLR;VDLVLLGK;VETTEDLVAK | 7 | 0.2549 | Electron transfer flavoprotein subunit beta OS=Homo sapiens OX=9606 GN=ETFB PE=1 SV=3 |
| sp\|P63104\|1433Z_HUMAN | 22.97320357 | 27727.72799 | EKIETELR;FLIPNASQAESK;GIVDQSQQAYQEAFEISK;SVTEQGAELSNEER;VVSSIEQK;YLAEVAAGDDK;YLAEVAAGDDKK | 7 | 0.2939 | 14-3-3 protein zeta/delta OS=Homo sapiens OX=9606 GN=YWHAZ PE=1 SV=1 |
| sp\|P62241\|RS8_HUMAN | 20.14723295 | 24190.16227 | ELEFYLR;KYELGRPAANTK;LTPEEEEILNK;LTPEEEEILNKK;NCIVLIDSTPYR;QWYESHYALPLGR;YELGRPAANTK | 7 | 0.2692 | 40S ribosomal protein S8 OS=Homo sapiens OX=9606 GN=RPS8 PE=1 SV=2 |
| sp\|P46781\|RS9_HUMAN | 20.47340595 | 22577.55535 | ELLTLDEKDPR;IGVLDEGK;KQVVNIPSFIVR;LDYILGLK;LFEGNALLR;MKLDYILGLK;QVVNIPSFIVR | 7 | 0.2577 | 40S ribosomal protein S9 OS=Homo sapiens OX=9606 GN=RPS9 PE=1 SV=3 |
| sp\|P30086\|PEBP1_HUMAN | 25.97739737 | 21043.66567 | EWHHFLVVNMK;GNDISSGTVLSDYVGSGPPK;LYTLVLTDPDAPSR;LYTLVLTDPDAPSRK;VLTPTQVK;WSGPLSLQEVDEQPQHPLHVTYAGAAVDELGK;YVWLVYEQDRPLK | 7 | 0.5294 | Phosphatidylethanolamine-binding protein 1 OS=Homo sapiens OX=9606 GN=PEBP1 PE=1 SV=3 |
| sp\|Q02543\|RL18A_HUMAN | 21.41276043 | 20748.89948 | FWYFVSQLK;FWYFVSQLKK;IFAPNHVVAK;RPNTFF;SRFWYFVSQLK;SSGEIVYCGQVFEK;VKNFGIWLR | 7 | 0.2898 | 60S ribosomal protein L18a OS=Homo sapiens OX=9606 GN=RPL18A PE=1 SV=2 |
| sp\|Q9Y295\|DRG1_HUMAN | 22.76740474 | 40516.82262 | GGGGGGPGEGFDVAK;GQLPDYTSPVVLPYSR;IGFVGFPSVGK;IIENELEGFGIR;IQLLDLPGIIEGAK;LNSKPPNIGFK;WNFDDLLEK | 7 | 0.2398 | Developmentally-regulated GTP-binding protein 1 OS=Homo sapiens OX=9606 GN=DRG1 PE=1 SV=1 |
| sp\|Q13561\|DCTN2_HUMAN | 20.69605115 | 44203.85528 | LLGPDAAINLTDPDGALAK;LTPVLLAK;QQLVASHLEK;RLLLQLEATK;VHQLYETIQR;WSPIASTLPELVQR;YADLPGIAR | 7 | 0.1995 | Dynactin subunit 2 OS=Homo sapiens OX=9606 GN=DCTN2 PE=1 SV=4 |
| sp\|O14745\|NHRF1_HUMAN | 21.45243277 | 38844.60938 | AALNAVR;KGPSGYGFNLHSDK;LLVVDPETDEQLQK;LVEVNGENVEKETHQQVVSR;QHGDVVSAIR;SVDPDSPAEASGLR | 6 | 0.2207 | Na(+)/H(+) exchange regulatory cofactor NHE-RF1 OS=Homo sapiens OX=9606 GN=SLC9A3R1 PE=1 SV=4 |
| sp\|Q9Y6G9\|DC1L1_HUMAN | 16.49376723 | 56543.99918 | AEDNFEDIITKPPVR;IGILHENFQTLK;IPAVVVEK;LQSLLAK;NVLLLGEDGAGK;QPPTAAGRPVDASPR | 6 | 0.1319 | Cytoplasmic dynein 1 light intermediate chain 1 OS=Homo sapiens OX=9606 GN=DYNC1LI1 PE=1 SV=3 |
| sp\|P05388\|RLA0_HUMAN | 20.73158218 | 34251.79701 | AFLADPSAFVAAAPVAAATTAAPAAAAAPAK;AGAIAPCEVTVPAQNTGLGPEK;AVVLMGK;GHLENNPALEK;GNVGFVFTK;IIQLLDDYPK | 6 | 0.2839 | 60S acidic ribosomal protein P0 OS=Homo sapiens OX=9606 GN=RPLP0 PE=1 SV=1 |
| sp\|P27487\|DPP4_HUMAN | 19.26239895 | 88222.48321 | AGAVNPTVK;HSYTASYDIYDLNK;HSYTASYDIYDLNKR;IISNEEGYR;LAYVWNNDIYVK;WEYYDSVYTER | 6 | 0.0731 | Dipeptidyl peptidase 4 OS=Homo sapiens OX=9606 GN=DPP4 PE=1 SV=2 |
| sp\|O43175\|SERA_HUMAN | 20.65668496 | 56614.40332 | AGTGVDNVDLEAATR;ALVDHENVISCPHLGASTK;GTIQVITQGTSLK;ILQDGGLQVVEK;QHVTEAFQFHF;TLGILGLGR | 6 | 0.1482 | D-3-phosphoglycerate dehydrogenase OS=Homo sapiens OX=9606 GN=PHGDH PE=1 SV=4 |
| sp\|P62424\|RL7A_HUMAN | 20.65668496 | 29977.02345 | AGVNTVTTLVENK;AGVNTVTTLVENKK;KVVNPLFEK;LKVPPAINQFTQALDR;NFGIGQDIQPK;QTATQLLK | 6 | 0.218 | 60S ribosomal protein L7a OS=Homo sapiens OX=9606 GN=RPL7A PE=1 SV=2 |
| sp\|Q7Z6Z7\|HUWE1_HUMAN | 17.96557923 | 481588.6479 | AIQDPAFSDGIR;LGSSGLGSASSIQAAVR;LLVGNDDVHIIAR;LPGGVQNFPQFSALR;LQHLAESWGGK;SLLSILQR | 6 | 0.0174 | E3 ubiquitin-protein ligase HUWE1 OS=Homo sapiens OX=9606 GN=HUWE1 PE=1 SV=3 |
| sp\|P30153\|2AAA_HUMAN | 19.92302574 | 65266.91209 | AISHEHSPSDLEAHFVPLVK;ELVSDANQHVK;IGPILDNSTLQSEVKPILEK;KLSTIALALGVER;LAGGDWFTSR;LTQDQDVDVK | 6 | 0.1426 | Serine/threonine-protein phosphatase 2A 65 kDa regulatory subunit A alpha isoform OS=Homo sapiens OX=9606 GN=PPP2R1A PE=1 SV=4 |
| sp\|P33991\|MCM4_HUMAN | 19.62654293 | 96497.62991 | ALADDDFLTVTGK;ATPAQTPR;GILLQLFGGTR;LHGLDEEAEQK;THIDVIHYR;TSVLAAANPIESQWNPK | 6 | 0.08 | DNA replication licensing factor MCM4 OS=Homo sapiens OX=9606 GN=MCM4 PE=1 SV=5 |
| sp\|O75390\|CISY_HUMAN | 17.76711976 | 51679.51269 | ALGFPLERPK;DILADLIPK;DYIWNTLNSGR;IVPNVLLEQGK;LRDYIWNTLNSGR;VVPGYGHAVLR | 6 | 0.1159 | Citrate synthase, mitochondrial OS=Homo sapiens OX=9606 GN=CS PE=1 SV=2 |
| sp\|P60660\|MYL6_HUMAN | 20.79434725 | 16919.13288 | ALGQNPTNAEVLK;DQGTYEDYVEGLR;EAFQLFDR;HVLVTLGEK;NKDQGTYEDYVEGLR;VLDFEHFLPMLQTVAK | 6 | 0.404 | Myosin light polypeptide 6 OS=Homo sapiens OX=9606 GN=MYL6 PE=1 SV=2 |
| sp\|P25205\|MCM3_HUMAN | 20.429658 | 90924.02531 | ALKDFVASIDATYAK;IIKPVLTQESATYIAEEYSR;LIVNVNDLRR;LTESINR;SKDIFDQLAK;VALLDVFR | 6 | 0.0866 | DNA replication licensing factor MCM3 OS=Homo sapiens OX=9606 GN=MCM3 PE=1 SV=3 |
| sp\|P04843\|RPN1_HUMAN | 20.80601245 | 68526.80688 | ALTSEIALLQSR;APDELHYTYLDTFGRPVIVAYK;FPLFGGWK;SEDLLDYGPFR;VACITEQVLTLVNKR;VTAEVVLAHLGGGSTSR | 6 | 0.14 | Dolichyl-diphosphooligosaccharide--protein glycosyltransferase subunit 1 OS=Homo sapiens OX=9606 GN=RPN1 PE=1 SV=1 |
| sp\|Q16531\|DDB1_HUMAN | 17.21767527 | 126887.321 | ALYYLQIHPQELR;IAVMELFRPK;LPSFELLHK;LYDGLFK;LYEWTTEK;YLAIAPPIIK | 6 | 0.05 | DNA damage-binding protein 1 OS=Homo sapiens OX=9606 GN=DDB1 PE=1 SV=1 |
| sp\|Q14974\|IMB1_HUMAN | 18.45061197 | 97108.01319 | ANFDKESER;DTAAWTVGR;LLETTDRPDGHQNNLR;LQQVLQMESHIQSTSDR;TTLVIMER;TVSPDRLELEAAQK | 6 | 0.0833 | Importin subunit beta-1 OS=Homo sapiens OX=9606 GN=KPNB1 PE=1 SV=2 |
| sp\|Q00341\|VIGLN_HUMAN | 17.8902482 | 141368.1383 | ANSFTVSSVAAPSWLHR;ASVITQVFHVPLEER;ELLELASR;HEVLLISAEQDKR;IEGDPQGVQQAK;LHNSLIGTK | 6 | 0.0584 | Vigilin OS=Homo sapiens OX=9606 GN=HDLBP PE=1 SV=2 |
| sp\|Q9Y265\|RUVB1_HUMAN | 21.69807765 | 50196.31316 | AQTEGINISEEALNHLGEIGTK;AVLLAGPPGTGK;LDPSIFESLQK;QAASGLVGQENAR;TALALAIAQELGSK;TISHVIIGLK | 6 | 0.1798 | RuvB-like 1 OS=Homo sapiens OX=9606 GN=RUVBL1 PE=1 SV=1 |
| sp\|O14950\|ML12B_HUMAN | 20.64029083 | 19766.51563 | ATSNVFAMFDQSQIQEFK;EAFNMIDQNR;ELLTTMGDR;FTDEEVDELYR;KGNFNYIEFTR;LNGTDPEDVIR | 6 | 0.407 | Myosin regulatory light chain 12B OS=Homo sapiens OX=9606 GN=MYL12B PE=1 SV=2 |
| sp\|Q9Y230\|RUVB2_HUMAN | 18.00458997 | 51124.55341 | AVLIAGQPGTGK;GLGLDDALEPR;GTSYQSPHGIPIDLLDR;IGLETSLR;LLIVSTTPYSEK;VYSLFLDESR | 6 | 0.1512 | RuvB-like 2 OS=Homo sapiens OX=9606 GN=RUVBL2 PE=1 SV=3 |
| sp\|Q13200\|PSMD2_HUMAN | 21.47460078 | 100135.7878 | AVPLALALISVSNPR;FGGSGSQVDSAR;LNILDTLSK;SETELKDTYAR;VGQAVDVVGQAGKPK;WLPLGLGLNHLGK | 6 | 0.0826 | 26S proteasome non-ATPase regulatory subunit 2 OS=Homo sapiens OX=9606 GN=PSMD2 PE=1 SV=3 |
| sp\|P05455\|LA_HUMAN | 21.33653939 | 46808.15992 | DANNGNLQLR;EVTWEVLEGEVEK;FVETPGQK;GSIFVVFDSIESAK;SPSKPLPEVTDEYKNDVK;YKETDLLILFK | 6 | 0.1814 | Lupus La protein OS=Homo sapiens OX=9606 GN=SSB PE=1 SV=2 |
| sp\|Q13838\|DX39B_HUMAN | 19.92474667 | 48959.94062 | DFLLKPELLR;GSYVSIHSSGFR;ILVATNLFGR;LTLHGLQQYYVK;RILVATNLFGR;VAVFFGGLSIK | 6 | 0.1308 | Spliceosome RNA helicase DDX39B OS=Homo sapiens OX=9606 GN=DDX39B PE=1 SV=1 |
| sp\|P00387\|NB5R3_HUMAN | 17.97366665 | 34212.72196 | DILLRPELEELR;EIISHDTR;GPSGLLVYQGK;LIDREIISHDTR;LWYTLDR;STPAITLESPDIKYPLR | 6 | 0.196 | NADH-cytochrome b5 reductase 3 OS=Homo sapiens OX=9606 GN=CYB5R3 PE=1 SV=3 |
| sp\|P06493\|CDK1_HUMAN | 18.76666584 | 34073.88292 | DLKPQNLLIDDKGTIK;IGEGTYGVVYK;KPLFHGDSEIDQLFR;NLDENGLDLLSK;SPEVLLGSAR;VYTHEVVTLWYR | 6 | 0.2559 | Cyclin-dependent kinase 1 OS=Homo sapiens OX=9606 GN=CDK1 PE=1 SV=3 |
| sp\|O75534\|CSDE1_HUMAN | 15.48624704 | 88828.87052 | EAFGFIER;IKVDFVIPK;INFVIDNNK;LTIAFQAK;NITLDDASAPR;VTLLEGDHVR | 6 | 0.0689 | Cold shock domain-containing protein E1 OS=Homo sapiens OX=9606 GN=CSDE1 PE=1 SV=2 |
| sp\|P62750\|RL23A_HUMAN | 17.11948981 | 17684.1338 | EAPAPPKAEAK;LYDIDVAK;NKLDHYAIIK;TSPTFR;VNTLIRPDGEK;VNTLIRPDGEKK | 6 | 0.3013 | 60S ribosomal protein L23a OS=Homo sapiens OX=9606 GN=RPL23A PE=1 SV=1 |
| sp\|O43776\|SYNC_HUMAN | 18.76403782 | 62902.51588 | EGIDPTPYYWYTDQR;FLTWILNR;IFDSEEILAGYK;IGALEGYR;NLMFLVLR;WNVISK | 6 | 0.104 | Asparagine--tRNA ligase, cytoplasmic OS=Homo sapiens OX=9606 GN=NARS1 PE=1 SV=1 |
| sp\|O75821\|EIF3G_HUMAN | 18.09289058 | 35588.91969 | ELAEQLGLSTGEK;GFAFISFHR;LPGELEPVQATQNK;TVTEYKIDEDGK;VTNLSEDTR;VTNLSEDTRETDLQELFRPFGSISR | 6 | 0.2281 | Eukaryotic translation initiation factor 3 subunit G OS=Homo sapiens OX=9606 GN=EIF3G PE=1 SV=2 |
| sp\|Q9Y266\|NUDC_HUMAN | 20.16268831 | 38219.12684 | ELTDEEAER;GQPAIIDGELYNEVK;LITQTFSHHNQLAQK;LKPNLGNGADLPNYR;LSDLDSETR;LVSSDPEINTK | 6 | 0.2236 | Nuclear migration protein nudC OS=Homo sapiens OX=9606 GN=NUDC PE=1 SV=1 |
| sp\|Q14847\|LASP1_HUMAN | 21.43345911 | 29698.21951 | EPAAPVSIQR;IVYPTEK;LKQQSELQSQVR;QQSELQSQVR;QSFTMVADTPENLR;RPLEQQQPHHIPTSAPVYQQPQQQPVAQSYGGYKEPAAPVSIQR | 6 | 0.295 | LIM and SH3 domain protein 1 OS=Homo sapiens OX=9606 GN=LASP1 PE=1 SV=2 |
| sp\|P35998\|PRS7_HUMAN | 19.87602957 | 48603.11772 | FDDGAGGDNEVQR;IATEKDFLEAVNK;KIEFSLPDLEGR;QTLQSEQPLQVAR;QVEDDIQQLLK;TYGQSTYSR | 6 | 0.164 | 26S proteasome regulatory subunit 7 OS=Homo sapiens OX=9606 GN=PSMC2 PE=1 SV=3 |
| sp\|Q15942\|ZYX_HUMAN | 20.26616498 | 61238.16586 | FSPGAPGGSGSQPNQK;FSPVTPK;GPPASSPAPAPK;LGHPEALSAGTGSPQPPSFTYAQQR;QHPVPPPAQNQNQVR;VNPFRPGDSEPPPAPGAQR | 6 | 0.1643 | Zyxin OS=Homo sapiens OX=9606 GN=ZYX PE=1 SV=1 |
| sp\|Q9NTK5\|OLA1_HUMAN | 21.14677453 | 44715.29773 | FYHDWNDKEIEVLNK;GGDGIKPPPIIGR;IGIVGLPNVGK;LQELSAEER;NYIVEDGDIIFFK;YEDFKEEGSENAVK | 6 | 0.1894 | Obg-like ATPase 1 OS=Homo sapiens OX=9606 GN=OLA1 PE=1 SV=2 |
| sp\|Q9UHX1\|PUF60_HUMAN | 19.24519868 | 59837.62313 | GYGFIEYEK;LGLPPLTPEQQEALQK;QAFAPFGPIK;SVFEAFGK;VVAEVYDQER;VYVGSIYYELGEDTIR | 6 | 0.1234 | Poly(U)-binding-splicing factor PUF60 OS=Homo sapiens OX=9606 GN=PUF60 PE=1 SV=1 |
| sp\|Q12905\|ILF2_HUMAN | 20.99863774 | 43035.18626 | ILITTVPPNLR;ILPTLEAVAALGNK;KLDPELHLDIK;NQDLAPNSAEQASILSLVTK;VLQSALAAIR;WFEENASQSTVK | 6 | 0.2 | Interleukin enhancer-binding factor 2 OS=Homo sapiens OX=9606 GN=ILF2 PE=1 SV=2 |
| sp\|P07954\|FUMH_HUMAN | 18.12493532 | 54602.16757 | AAAEVNQDYGLDPK;IEYDTFGELK;IPVHPNDHVNK;IYELAAGGTAVGTGLNTR;VAALTGLPFVTAPNK | 5 | 0.1333 | Fumarate hydratase, mitochondrial OS=Homo sapiens OX=9606 GN=FH PE=1 SV=3 |
| sp\|P35637\|FUS_HUMAN | 17.79710906 | 53393.79057 | AAIDWFDGK;AAIDWFDGKEFSGNPIK;APKPDGPGGGPGGSHMGGNYGDDR;GEATVSFDDPPSAK;LKGEATVSFDDPPSAK | 5 | 0.1084 | RNA-binding protein FUS OS=Homo sapiens OX=9606 GN=FUS PE=1 SV=1 |
| sp\|P46783\|RS10_HUMAN | 18.29865428 | 18885.86619 | AEAGAGSATEFQFR;DYLHLPPEIVPATLR;HFYWYLTNEGIQYLR;HPELADK;KAEAGAGSATEFQFR | 5 | 0.3152 | 40S ribosomal protein S10 OS=Homo sapiens OX=9606 GN=RPS10 PE=1 SV=1 |
| sp\|P40926\|MDHM_HUMAN | 17.02772776 | 35480.73194 | AGAGSATLSMAYAGAR;ANTFVAELK;IFGVTTLDIVR;VDFPQDQLTALTGR;VNVPVIGGHAGK | 5 | 0.1834 | Malate dehydrogenase, mitochondrial OS=Homo sapiens OX=9606 GN=MDH2 PE=1 SV=3 |
| sp\|Q15393\|SF3B3_HUMAN | 15.39648398 | 135491.7384 | AGNGQWASVIR;LGAVFNQVAFPLQYTPR;LTISSPLEAHK;LVYILNR;TVLDPVTGDLSDTR | 5 | 0.0493 | Splicing factor 3B subunit 3 OS=Homo sapiens OX=9606 GN=SF3B3 PE=1 SV=4 |
| sp\|Q92616\|GCN1_HUMAN | 14.68591904 | 292572.4635 | AIITALGVER;ALVAVLLSR;HHIETGGGQLPAK;KLDAGNQLALIEELHK;LLTWVIGTGSPR | 5 | 0.0225 | eIF-2-alpha kinase activator GCN1 OS=Homo sapiens OX=9606 GN=GCN1 PE=1 SV=6 |
| sp\|P60900\|PSA6_HUMAN | 17.71353912 | 27381.80857 | AINQGGLTSVAVR;HITIFSPEGR;LLDSSTVTHLFK;QTESTSFLEK;YEAANWK | 5 | 0.2114 | Proteasome subunit alpha type-6 OS=Homo sapiens OX=9606 GN=PSMA6 PE=1 SV=1 |
| sp\|Q15102\|PA1B3_HUMAN | 14.91669668 | 25718.23189 | AIVQLVNER;ALHSLLLR;IVVVWVGTNNHGHTAEQVTGGIK;LENGELEHIRPK;VVVLGLLPR | 5 | 0.2641 | Platelet-activating factor acetylhydrolase IB subunit gamma OS=Homo sapiens OX=9606 GN=PAFAH1B3 PE=1 SV=1 |
| sp\|P39019\|RS19_HUMAN | 15.54630216 | 16050.53475 | ALAAFLK;ELAPYDENWFYTR;IAGQVAAANK;LKVPEWVDTVK;RVLQALEGLK | 5 | 0.3517 | 40S ribosomal protein S19 OS=Homo sapiens OX=9606 GN=RPS19 PE=1 SV=2 |
| sp\|Q8NBS9\|TXND5_HUMAN | 15.01209521 | 47598.66009 | ALAPTWEQLALGLEHSETVK;EFPGLAGVK;GYPTLLLFR;GYPTLLWFR;TLAPTWEELSKK | 5 | 0.1366 | Thioredoxin domain-containing protein 5 OS=Homo sapiens OX=9606 GN=TXNDC5 PE=1 SV=2 |
| sp\|P38159\|RBMX_HUMAN | 16.64397794 | 42306.33305 | ALEAVFGK;GFAFVTFESPADAK;IVEVLLMK;LFIGGLNTETNEK;VEQATKPSFESGR | 5 | 0.1432 | RNA-binding motif protein, X chromosome OS=Homo sapiens OX=9606 GN=RBMX PE=1 SV=3 |
| sp\|O95831\|AIFM1_HUMAN | 19.99163286 | 66858.9054 | ALGTEVIQLFPEK;APSHVPFLLIGGGTAAFAAAR;ILPEYLSNWTMEK;LNDGSQITYEK;TGGLEIDSDFGGFR | 5 | 0.1175 | Apoptosis-inducing factor 1, mitochondrial OS=Homo sapiens OX=9606 GN=AIFM1 PE=1 SV=1 |
| sp\|Q01518\|CAP1_HUMAN | 15.82114008 | 51868.69175 | ALLVTASQCQQPAENK;GYADSPSK;KEPAVLELEGK;LEAVSHTSDMHR;VPTISINK | 5 | 0.1158 | Adenylyl cyclase-associated protein 1 OS=Homo sapiens OX=9606 GN=CAP1 PE=1 SV=5 |
| sp\|P18669\|PGAM1_HUMAN | 19.166328 | 28785.83459 | ALPFWNEEIVPQIK;FSGWYDADLSPAGHEEAK;FSGWYDADLSPAGHEEAKR;HGESAWNLENR;VLIAAHGNSLR | 5 | 0.2165 | Phosphoglycerate mutase 1 OS=Homo sapiens OX=9606 GN=PGAM1 PE=1 SV=2 |
| sp\|Q9UNM6\|PSD13_HUMAN | 15.99426694 | 42918.14634 | ALSVGLVK;DLPVSEQQER;LYENFISEFEHR;TAWGQQPDLAANEAQLLR;VLDLQQIK | 5 | 0.1489 | 26S proteasome non-ATPase regulatory subunit 13 OS=Homo sapiens OX=9606 GN=PSMD13 PE=1 SV=2 |
| sp\|P62491\|RB11A_HUMAN | 14.63366663 | 24378.41247 | AQIWDTAGQER;DDEYDYLFK;GAVGALLVYDIAK;HLTYENVER;VVLIGDSGVGK | 5 | 0.2454 | Ras-related protein Rab-11A OS=Homo sapiens OX=9606 GN=RAB11A PE=1 SV=3 |
| sp\|P14314\|GLU2B_HUMAN | 14.73697753 | 59387.82018 | AQQEQELAADAFK;ESLQQMAEVTR;ETMVTSTTEPSR;ILIEDWK;LWEEQLAAAK | 5 | 0.1004 | Glucosidase 2 subunit beta OS=Homo sapiens OX=9606 GN=PRKCSH PE=1 SV=2 |
| sp\|P22234\|PUR6_HUMAN | 15.29295213 | 47049.13597 | ASILNTWISLK;IATGSFLK;IEFGVDVTTK;NFEWVAER;TKEVYELLDSPGK | 5 | 0.1176 | Multifunctional protein ADE2 OS=Homo sapiens OX=9606 GN=PAICS PE=1 SV=3 |
| sp\|P14324\|FPPS_HUMAN | 18.01319689 | 48244.62818 | ATPEQYQILK;GLTVVVAFR;KQDADSLQR;LKEVLEYNAIGGK;QDFVQHFSQIVR | 5 | 0.1265 | Farnesyl pyrophosphate synthase OS=Homo sapiens OX=9606 GN=FDPS PE=1 SV=4 |
| sp\|P23528\|COF1_HUMAN | 17.31375698 | 18490.65742 | AVLFCLSEDKK;KEDLVFIFWAPESAPLK;LGGSAVISLEGKPL;NIILEEGK;YALYDATYETK | 5 | 0.3675 | Cofilin-1 OS=Homo sapiens OX=9606 GN=CFL1 PE=1 SV=3 |
| sp\|Q13011\|ECH1_HUMAN | 13.81917482 | 35793.38262 | AVVISGAGK;HVLHVQLNRPNK;HVLHVQLNRPNKR;YCAQDAFFQVK;YQETFNVIER | 5 | 0.1311 | Delta(3,5)-Delta(2,4)-dienoyl-CoA isomerase, mitochondrial OS=Homo sapiens OX=9606 GN=ECH1 PE=1 SV=2 |
| sp\|P62280\|RS11_HUMAN | 12.42379671 | 18418.99282 | CPFTGNVSIR;DYLHYIR;EAIEGTYIDKK;NIGLGFK;VLLGETGKEK | 5 | 0.2848 | 40S ribosomal protein S11 OS=Homo sapiens OX=9606 GN=RPS11 PE=1 SV=3 |
| sp\|P06748\|NPM_HUMAN | 17.68248178 | 32554.84112 | DELHIVEAEAMNYEGSPIK;GPSSVEDIKAK;MTDQEAIQDLWQWR;VDNDENEHQLSLR;VTLATLK | 5 | 0.2177 | Nucleophosmin OS=Homo sapiens OX=9606 GN=NPM1 PE=1 SV=2 |
| sp\|O00151\|PDLI1_HUMAN | 17.22120492 | 36049.04184 | DFEQPLAISR;QSTSFLVLQEILESEEKGDPNKPSGFR;TAASGVEANSRPLDHAQPPSSLVIDKESEVYK;VTPPEGYEVVTVFPK;VWSPLVTEEGKR | 5 | 0.2918 | PDZ and LIM domain protein 1 OS=Homo sapiens OX=9606 GN=PDLIM1 PE=1 SV=4 |
| sp\|P24752\|THIL_HUMAN | 13.67145616 | 45170.64265 | DGLTDVYNK;FGNEVIPVTVTVK;IVGHLTHALK;LGSIAIQGAIEK;NEQDAYAINSYTR | 5 | 0.1335 | Acetyl-CoA acetyltransferase, mitochondrial OS=Homo sapiens OX=9606 GN=ACAT1 PE=1 SV=1 |
| sp\|P22087\|FBRL_HUMAN | 19.27806643 | 33763.41765 | DHAVVVGVYRPPPK;IVALNAHTFLR;LAAAILGGVDQIHIKPGAK;TNIIPVIEDAR;VSISEGDDKIEYR | 5 | 0.2118 | rRNA 2'-O-methyltransferase fibrillarin OS=Homo sapiens OX=9606 GN=FBL PE=1 SV=2 |
| sp\|Q99829\|CPNE1_HUMAN | 15.82821073 | 59021.54273 | DIVQFVPYR;GTITVSAQELKDNR;LYGPTNFAPIINHVAR;QALPQVR;SDPFLEFFR | 5 | 0.1024 | Copine-1 OS=Homo sapiens OX=9606 GN=CPNE1 PE=1 SV=1 |
| sp\|Q02790\|FKBP4_HUMAN | 17.35754437 | 51772.07045 | DKFSFDLGK;GEHSIVYLKPSYAFGSVGK;RGEAHLAVNDFELAR;SNTAGSQSQVETEA;VFVHYTGWLLDGTK | 5 | 0.1547 | Peptidyl-prolyl cis-trans isomerase FKBP4 OS=Homo sapiens OX=9606 GN=FKBP4 PE=1 SV=3 |
| sp\|P98082\|DAB2_HUMAN | 17.24598396 | 82396.99881 | DLFQVIYNVK;INEPPKPAPR;KGEQTSSGTLSAFASYFNSK;STDNAFENPFFK;TDEYLLAR | 5 | 0.0779 | Disabled homolog 2 OS=Homo sapiens OX=9606 GN=DAB2 PE=1 SV=3 |
| sp\|Q99729\|ROAA_HUMAN | 16.47914406 | 36202.40732 | DLKDYFTK;EVYQQQQYGSGGR;GFGFILFK;GFVFITFK;MFVGGLSWDTSK | 5 | 0.1476 | Heterogeneous nuclear ribonucleoprotein A/B OS=Homo sapiens OX=9606 GN=HNRNPAB PE=1 SV=2 |
| sp\|P23284\|PPIB_HUMAN | 16.10240954 | 23727.53502 | DTNGSQFFITTVK;IEVEKPFAIAK;TAWLDGK;TVDNFVALATGEK;VIFGLFGK | 5 | 0.2407 | Peptidyl-prolyl cis-trans isomerase B OS=Homo sapiens OX=9606 GN=PPIB PE=1 SV=2 |
| sp\|P40925\|MDHC_HUMAN | 16.09181431 | 36403.01945 | DVIATDKEDVAFK;ELTEEKESAFEFLSSA;FVEGLPINDFSR;LGVTANDVK;VIVVGNPANTNCLTASK | 5 | 0.2006 | Malate dehydrogenase, cytoplasmic OS=Homo sapiens OX=9606 GN=MDH1 PE=1 SV=4 |
| sp\|Q13151\|ROA0_HUMAN | 19.38980486 | 30821.78445 | EDIYSGGGGGGSR;GDVAEGDLIEHFSQFGTVEK;GFGFVYFQNHDAADK;RGFGFVYFQNHDAADK;SGGGGGGGGSSWGGR | 5 | 0.2098 | Heterogeneous nuclear ribonucleoprotein A0 OS=Homo sapiens OX=9606 GN=HNRNPA0 PE=1 SV=1 |
| sp\|P51991\|ROA3_HUMAN | 20.31945912 | 39570.58264 | EDSVKPGAHLTVK;GFAFVTFDDHDTVDK;GFAFVTFDDHDTVDKIVVQK;IFVGGIKEDTEEYNLR;SSGSPYGGGYGSGGGSGGYGSR | 5 | 0.1905 | Heterogeneous nuclear ribonucleoprotein A3 OS=Homo sapiens OX=9606 GN=HNRNPA3 PE=1 SV=2 |
| sp\|Q12904\|AIMP1_HUMAN | 15.79650507 | 34331.11143 | EIEELKQELIQAEIQNGVK;GAEADQIIEYLK;IEILAPPNGSVPGDR;KHPDADSLYVEEVDVGEIAPR;KQQSIAGSADSKPIDVSR | 5 | 0.2724 | Aminoacyl tRNA synthase complex-interacting multifunctional protein 1 OS=Homo sapiens OX=9606 GN=AIMP1 PE=1 SV=2 |
| sp\|P52292\|IMA1_HUMAN | 16.34673729 | 57825.94619 | EKQPPIDNIIR;FVSFLGR;GINSSNVENQLQATQAAR;LLGASELPIVTPALR;TGVVPQLVK | 5 | 0.1134 | Importin subunit alpha-1 OS=Homo sapiens OX=9606 GN=KPNA2 PE=1 SV=1 |
| sp\|Q96AG4\|LRC59_HUMAN | 18.10504595 | 34908.8759 | ELAALPK;LVNLQHLDLLNNK;LVTLPVSFAQLK;RHEILQWVLQTDSQQ;WLDLKDNPLDPVLAK | 5 | 0.202 | Leucine-rich repeat-containing protein 59 OS=Homo sapiens OX=9606 GN=LRRC59 PE=1 SV=1 |
| sp\|O15067\|PUR4_HUMAN | 18.1027673 | 144642.8874 | ELSDPAGAIIYTSR;FGEPVLAGFAR;GHLLYVALSPGQHR;HWFFK;LSFAHPPSAEVEAIALATLHDR | 5 | 0.0493 | Phosphoribosylformylglycinamidine synthase OS=Homo sapiens OX=9606 GN=PFAS PE=1 SV=4 |
| sp\|Q9HDC9\|APMAP_HUMAN | 15.80559971 | 46450.84937 | EPPLLLGVLHPNTK;GLFEVNPWK;LENGEIETIAR;RPLRPQVVTDDDGQAPEAK;VLLDQLR | 5 | 0.1442 | Adipocyte plasma membrane-associated protein OS=Homo sapiens OX=9606 GN=APMAP PE=1 SV=2 |
| sp\|P05141\|ADT2_HUMAN | 12.6611322 | 32831.15033 | EQGVLSFWR;GAWSNVLR;LLLQVQHASK;QIFLGGVDKR;YFPTQALNFAFK | 5 | 0.1644 | ADP/ATP translocase 2 OS=Homo sapiens OX=9606 GN=SLC25A5 PE=1 SV=7 |
| sp\|P18621\|RL17_HUMAN | 17.91062088 | 21383.33238 | EQIVPKPEEEVAQK;GLDVDSLVIEHIQVNK;QWGWTQGR;SAEFLLHMLK;YSLDPENPTK | 5 | 0.3152 | 60S ribosomal protein L17 OS=Homo sapiens OX=9606 GN=RPL17 PE=1 SV=3 |
| sp\|P49736\|MCM2_HUMAN | 15.70843861 | 101832.1564 | ESLVVNYEDLAAR;GLALALFGGEPK;IFASIAPSIYGHEDIKR;ISHLPLVEELR;QLHLNQLIR | 5 | 0.0686 | DNA replication licensing factor MCM2 OS=Homo sapiens OX=9606 GN=MCM2 PE=1 SV=4 |
| sp\|P62195\|PRS8_HUMAN | 15.52025999 | 45597.05226 | EVIELPVKHPELFEALGIAQPK;FVVDVDK;IDILDSALLRPGR;IEELQLIVNDK;LEGGSGGDSEVQR | 5 | 0.1626 | 26S proteasome regulatory subunit 8 OS=Homo sapiens OX=9606 GN=PSMC5 PE=1 SV=1 |
| sp\|P62937\|PPIA_HUMAN | 17.53723384 | 18000.88496 | FEDENFILK;SIYGEKFEDENFILK;TEWLDGK;VSFELFADK;VSFELFADKVPK | 5 | 0.2424 | Peptidyl-prolyl cis-trans isomerase A OS=Homo sapiens OX=9606 GN=PPIA PE=1 SV=2 |
| sp\|P61313\|RL15_HUMAN | 16.14823375 | 24131.08353 | FFEVILIDPFHK;GATYGKPVHHGVNQLK;NTLQLHR;SLQSVAEER;VLNSYWVGEDSTYK | 5 | 0.2843 | 60S ribosomal protein L15 OS=Homo sapiens OX=9606 GN=RPL15 PE=1 SV=2 |
| sp\|P26639\|SYTC_HUMAN | 15.18237093 | 83381.86442 | FLGDIEVWDQAEK;LADFGVLHR;TVYSVFGFSFK;VNTPTTTVYR;VTLPDGK | 5 | 0.0692 | Threonine--tRNA ligase 1, cytoplasmic OS=Homo sapiens OX=9606 GN=TARS1 PE=1 SV=3 |
| sp\|P62826\|RAN_HUMAN | 18.69036495 | 24407.61733 | FNVWDTAGQEK;KYVATLGVEVHPLVFHTNR;LVLVGDGGTGK;SNYNFEKPFLWLAR;YVATLGVEVHPLVFHTNR | 5 | 0.2546 | GTP-binding nuclear protein Ran OS=Homo sapiens OX=9606 GN=RAN PE=1 SV=3 |
| sp\|P46777\|RL5_HUMAN | 12.93075759 | 34340.69362 | FPGYDSESK;GAVDGGLSIPHSTK;QFSQYIK;RFPGYDSESK;VGLTNYAAAYCTGLLLAR | 5 | 0.165 | 60S ribosomal protein L5 OS=Homo sapiens OX=9606 GN=RPL5 PE=1 SV=3 |
| sp\|P05556\|ITB1_HUMAN | 16.32186481 | 88357.00959 | GEVFNELVGK;IGFGSFVEK;LKPEDITQIQPQQLVLR;NVLSLTNKGEVFNELVGK;SGEPQTFTLK | 5 | 0.0677 | Integrin beta-1 OS=Homo sapiens OX=9606 GN=ITGB1 PE=1 SV=2 |
| sp\|O00299\|CLIC1_HUMAN | 15.70250037 | 26905.74764 | GFTIPEAFR;GVTFNVTTVDTK;GVTFNVTTVDTKR;IGNCPFSQR;LAALNPESNTAGLDIFAK | 5 | 0.2033 | Chloride intracellular channel protein 1 OS=Homo sapiens OX=9606 GN=CLIC1 PE=1 SV=4 |
| sp\|P09661\|RU2A_HUMAN | 16.01941634 | 28398.13292 | GGPSPGDVEAIK;GLLQSGQIPGR;LTAELIEQAAQYTNAVR;NAIANASTLAEVER;SLTYLSILR | 5 | 0.2471 | U2 small nuclear ribonucleoprotein A' OS=Homo sapiens OX=9606 GN=SNRPA1 PE=1 SV=2 |
| sp\|P09525\|ANXA4_HUMAN | 17.87011239 | 35860.12033 | GLGTDDNTLIR;ISQTYQQQYGR;NHLLHVFDEYKR;QDAQDLYEAGEK;VLVSLSAGGR | 5 | 0.1755 | Annexin A4 OS=Homo sapiens OX=9606 GN=ANXA4 PE=1 SV=4 |
| sp\|Q96AE4\|FUBP1_HUMAN | 15.36863389 | 67518.47027 | GTPQQIDYAR;IGGNEGIDVPIPR;IQFKPDDGTTPER;IQIAPDSGGLPER;TGLIIGK | 5 | 0.0994 | Far upstream element-binding protein 1 OS=Homo sapiens OX=9606 GN=FUBP1 PE=1 SV=3 |
| sp\|P62258\|1433E_HUMAN | 19.05458957 | 29155.41555 | HLIPAANTGESK;IISSIEQKEENK;KEAAENSLVAYK;YLAEFATGNDR;YLAEFATGNDRK | 5 | 0.1843 | 14-3-3 protein epsilon OS=Homo sapiens OX=9606 GN=YWHAE PE=1 SV=1 |
| sp\|P17174\|AATC_HUMAN | 16.8401764 | 46218.52806 | IANDNSLNHEYLPILGLAEFR;IVASTLSNPELFEEWTGNVK;NFGLYNER;VGGVQSLGGTGALR;VNLGVGAYR | 5 | 0.1743 | Aspartate aminotransferase, cytoplasmic OS=Homo sapiens OX=9606 GN=GOT1 PE=1 SV=3 |
| sp\|Q99832\|TCPH_HUMAN | 16.59544723 | 59328.92306 | IKEIAVTVK;LLDVVHPAAK;LPIGDVATQYFADR;QVKPYVEEGLHPQIIIR;SLHDAIMIVR | 5 | 0.1105 | T-complex protein 1 subunit eta OS=Homo sapiens OX=9606 GN=CCT7 PE=1 SV=2 |
| sp\|Q15019\|SEPT2_HUMAN | 16.14753547 | 41461.24964 | ILDEIEEHNIK;QQPTQFINPETPGYVGFANLPNQVHR;TIISYIDEQFER;VNIVPVIAK;YLHDESGLNR | 5 | 0.1884 | Septin-2 OS=Homo sapiens OX=9606 GN=SEPTIN2 PE=1 SV=1 |
| sp\|P37837\|TALDO_HUMAN | 14.21577734 | 37516.46095 | ILDWHVANTDK;LLGELLQDNAK;LSSTWEGIQAGK;LVPVLSAK;VSTEVDAR | 5 | 0.1484 | Transaldolase OS=Homo sapiens OX=9606 GN=TALDO1 PE=1 SV=2 |
| sp\|P02545\|LMNA_HUMAN | 17.15478829 | 74094.7071 | ITESEEVVSR;LKDLEALLNSK;NSNLVGAAHEELQQSR;SVGGSGGGSFGDNLVTR;TLEGELHDLR | 5 | 0.0964 | Prelamin-A/C OS=Homo sapiens OX=9606 GN=LMNA PE=1 SV=1 |
| sp\|Q08257\|QOR_HUMAN | 17.72243398 | 35184.54279 | IVLQNGAHEVFNHR;QGAAIGIPYFTAYR;VAEAHENIIHGSGATGK;VFTSSTISGGYAEYALAADHTVYK;VIVVGSR | 5 | 0.231 | Quinone oxidoreductase OS=Homo sapiens OX=9606 GN=CRYZ PE=1 SV=1 |
| sp\|P62851\|RS25_HUMAN | 13.03443725 | 13733.69831 | AALQELLSK;DKLNNLVLFDK;LITPAVVSER;LNNLVLFDK | 4 | 0.24 | 40S ribosomal protein S25 OS=Homo sapiens OX=9606 GN=RPS25 PE=1 SV=1 |
| sp\|P11766\|ADHX_HUMAN | 15.48883628 | 39698.37861 | AAVAWEAGKPLSIEEIEVAPPK;IIGVDINK;IKVDEFVTHNLSFDEINK;VDEFVTHNLSFDEINK | 4 | 0.1283 | Alcohol dehydrogenase class-3 OS=Homo sapiens OX=9606 GN=ADH5 PE=1 SV=4 |
| sp\|P51858\|HDGF_HUMAN | 13.89345285 | 26771.86615 | AGDLLEDSPK;GFSEGLWEIENNPTVK;KGFSEGLWEIENNPTVK;YQVFFFGTHETAFLGPK | 4 | 0.1833 | Hepatoma-derived growth factor OS=Homo sapiens OX=9606 GN=HDGF PE=1 SV=1 |
| sp\|P62873\|GBB1_HUMAN | 12.88141875 | 37353.0126 | AGVLAGHDNR;LIIWDSYTTNK;LLVSASQDGK;VHAIPLR | 4 | 0.1118 | Guanine nucleotide-binding protein G(I)/G(S)/G(T) subunit beta-1 OS=Homo sapiens OX=9606 GN=GNB1 PE=1 SV=3 |
| sp\|P50995\|ANX11_HUMAN | 13.75980102 | 54355.06916 | AHLVAVFNEYQR;DAQELYAAGENR;QQILLSFK;TPVLFDIYEIK | 4 | 0.0851 | Annexin A11 OS=Homo sapiens OX=9606 GN=ANXA11 PE=1 SV=1 |
| sp\|P62136\|PP1A_HUMAN | 14.07582994 | 37487.80356 | AHQVVEDGYEFFAK;IKYPENFFLLR;LLEVQGSRPGK;LNLDSIIGR | 4 | 0.1364 | Serine/threonine-protein phosphatase PP1-alpha catalytic subunit OS=Homo sapiens OX=9606 GN=PPP1CA PE=1 SV=1 |
| sp\|P22102\|PUR2_HUMAN | 12.62858331 | 107698.9975 | AIAFLQQPR;ENLISALEEAK;HGIPTAQWK;IYSHSLLPVLR | 4 | 0.0396 | Trifunctional purine biosynthetic protein adenosine-3 OS=Homo sapiens OX=9606 GN=GART PE=1 SV=1 |
| sp\|P28066\|PSA5_HUMAN | 12.00500238 | 26394.20454 | AIGSASEGAQSSLQEVYHK;EELEEVIKDI;GVNTFSPEGR;LFQVEYAIEAIK | 4 | 0.2116 | Proteasome subunit alpha type-5 OS=Homo sapiens OX=9606 GN=PSMA5 PE=1 SV=3 |
| sp\|P62081\|RS7_HUMAN | 12.86620344 | 22113.25751 | AIIIFVPVPQLK;AQQNNVEHKVETFSGVYK;HVVFIAQR;KAIIIFVPVPQLK | 4 | 0.201 | 40S ribosomal protein S7 OS=Homo sapiens OX=9606 GN=RPS7 PE=1 SV=1 |
| sp\|Q7Z739\|YTHD3_HUMAN | 12.66020771 | 63821.76294 | AITDGQAGFGNDTLSK;DFDWNLK;IGGDLTAAVTK;SYSEDDIHR | 4 | 0.0735 | YTH domain-containing family protein 3 OS=Homo sapiens OX=9606 GN=YTHDF3 PE=1 SV=1 |
| sp\|P83731\|RL24_HUMAN | 12.90375177 | 17767.89649 | AITGASLADIMAK;GQSEEIQK;VELCSFSGYK;VFQFLNAK | 4 | 0.2484 | 60S ribosomal protein L24 OS=Homo sapiens OX=9606 GN=RPL24 PE=1 SV=1 |
| sp\|Q13247\|SRSF6_HUMAN | 12.81547357 | 39563.39288 | ALDKLDGTEINGR;LIEDKPR;QAGEVTYADAHK;VIVEHAR | 4 | 0.1134 | Serine/arginine-rich splicing factor 6 OS=Homo sapiens OX=9606 GN=SRSF6 PE=1 SV=2 |
| sp\|O00429\|DNM1L_HUMAN | 10.37591791 | 81826.11108 | ALQGASQIIAEIR;GVSPEPIHLK;SATLLQLITK;TLAVITK | 4 | 0.0543 | Dynamin-1-like protein OS=Homo sapiens OX=9606 GN=DNM1L PE=1 SV=2 |
| sp\|P62333\|PRS10_HUMAN | 12.43963091 | 44145.09825 | ALQSVGQIVGEVLK;EVIELPLTNPELFQR;FSEGTSADR;LLEHKEIDGR | 4 | 0.1234 | 26S proteasome regulatory subunit 10B OS=Homo sapiens OX=9606 GN=PSMC6 PE=1 SV=1 |
| sp\|Q99497\|PARK7_HUMAN | 9.880153518 | 19878.48781 | ALVILAK;EILKEQENR;VTTHPLAK;VTVAGLAGK | 4 | 0.1746 | Parkinson disease protein 7 OS=Homo sapiens OX=9606 GN=PARK7 PE=1 SV=2 |
| sp\|Q9NYU2\|UGGG1_HUMAN | 10.5714387 | 177078.0084 | ANPGAWILR;FWFLK;IEYQFFEDR;INYVFR | 4 | 0.0186 | UDP-glucose:glycoprotein glucosyltransferase 1 OS=Homo sapiens OX=9606 GN=UGGT1 PE=1 SV=3 |
| sp\|Q92841\|DDX17_HUMAN | 15.19646268 | 80222.0164 | ELAQQVQQVADDYGK;LIDFLESGK;SSQSSSQQFSGIGR;TLAYLLPAIVHINHQPYLER | 4 | 0.1358 | Probable ATP-dependent RNA helicase DDX17 OS=Homo sapiens OX=9606 GN=DDX17 PE=1 SV=2 |
| sp\|Q9NSD9\|SYFB_HUMAN | 14.16319365 | 66073.5457 | ASEGPAFFPGR;IRPFAVAAVLR;NPGFEIIHGLLDR;TTLLPGLLK | 4 | 0.0747 | Phenylalanine--tRNA ligase beta subunit OS=Homo sapiens OX=9606 GN=FARSB PE=1 SV=3 |
| sp\|Q9Y5L0\|TNPO3_HUMAN | 12.33094622 | 104136.0724 | ASFWLGELQR;ETTVGAVTVTHK;GTALVLAR;SVHAWEISDQLLQIR | 4 | 0.0488 | Transportin-3 OS=Homo sapiens OX=9606 GN=TNPO3 PE=1 SV=3 |
| sp\|P29692\|EF1D_HUMAN | 13.56828453 | 31102.78262 | ATAPQTQHVSPMR;IWFDKFK;QENGASVILR;SLAGSSGPGASSGTSGDHGELVVR | 4 | 0.2171 | Elongation factor 1-delta OS=Homo sapiens OX=9606 GN=EEF1D PE=1 SV=5 |
| sp\|P09493\|TPM1_HUMAN | 13.96860468 | 32688.68102 | ATDAEADVASLNR;KATDAEADVASLNR;KLVIIESDLER;MEIQEIQLK | 4 | 0.1972 | Tropomyosin alpha-1 chain OS=Homo sapiens OX=9606 GN=TPM1 PE=1 SV=2 |
| sp\|P25705\|ATPA_HUMAN | 12.4537898 | 59713.59237 | AVDSLVPIGR;EAYPGDVFYLHSR;GIRPAINVGLSVSR;QMSLLLR | 4 | 0.0796 | ATP synthase subunit alpha, mitochondrial OS=Homo sapiens OX=9606 GN=ATP5F1A PE=1 SV=1 |
| sp\|P25788\|PSA3_HUMAN | 13.75591514 | 28415.08093 | AVENSSTAIGIR;HVGMAVAGLLADAR;IIYIVHDEVKDK;SNFGYNIPLK | 4 | 0.1882 | Proteasome subunit alpha type-3 OS=Homo sapiens OX=9606 GN=PSMA3 PE=1 SV=2 |
| sp\|P54727\|RD23B_HUMAN | 16.96979365 | 43144.61868 | AVEYLLMGIPGDR;ILNDDTALKEYK;QEKPAEKPAETPVATSPTATDSTSGDSSR;QIIQQNPSLLPALLQQIGR | 4 | 0.198 | UV excision repair protein RAD23 homolog B OS=Homo sapiens OX=9606 GN=RAD23B PE=1 SV=1 |
| sp\|O43747\|AP1G1_HUMAN | 12.32312422 | 91293.1273 | AVEYNALFK;IEFTFER;VLAINILGR;YVALTSLLK | 4 | 0.0414 | AP-1 complex subunit gamma-1 OS=Homo sapiens OX=9606 GN=AP1G1 PE=1 SV=5 |
| sp\|P27348\|1433T_HUMAN | 14.91293213 | 27746.76636 | AVTEQGAELSNEER;QTIDNSQGAYQEAFDISK;QTIDNSQGAYQEAFDISKK;YLIANATNPESK | 4 | 0.1837 | 14-3-3 protein theta OS=Homo sapiens OX=9606 GN=YWHAQ PE=1 SV=1 |
| sp\|Q8WXF1\|PSPC1_HUMAN | 11.64473888 | 58706.19366 | AVVVVDDR;GFVEFAAKPPAR;LFVGNLPTDITEEDFKR;YGEPSEVFINR | 4 | 0.0918 | Paraspeckle component 1 OS=Homo sapiens OX=9606 GN=PSPC1 PE=1 SV=1 |
| sp\|Q15392\|DHC24_HUMAN | 11.08211064 | 60061.99371 | AWVVFK;EGLEYIPLR;LNSIGNYYKPWFFK;LTQGETLR | 4 | 0.0717 | Delta(24)-sterol reductase OS=Homo sapiens OX=9606 GN=DHCR24 PE=1 SV=2 |
| sp\|Q14008\|CKAP5_HUMAN | 11.24571726 | 225351.7697 | DAAFEALGTALK;NLGIPIITVLGDSK;QELLGWLAEK;VNDFLAEIFK | 4 | 0.0226 | Cytoskeleton-associated protein 5 OS=Homo sapiens OX=9606 GN=CKAP5 PE=1 SV=3 |
| sp\|Q15717\|ELAV1_HUMAN | 13.63569006 | 36069.14952 | DANLYISGLPR;NVALLSQLYHSPAR;SLFSSIGEVESAK;VLVDQTTGLSR | 4 | 0.1503 | ELAV-like protein 1 OS=Homo sapiens OX=9606 GN=ELAVL1 PE=1 SV=2 |
| sp\|P62191\|PRS4_HUMAN | 11.34257084 | 49153.69272 | DDLSGADIK;GVILYGPPGTGK;IETLDPALIRPGR;VAEEHAPSIVFIDEIDAIGTK | 4 | 0.125 | 26S proteasome regulatory subunit 4 OS=Homo sapiens OX=9606 GN=PSMC1 PE=1 SV=1 |
| sp\|P27635\|RL10_HUMAN | 10.19862333 | 24587.86717 | DGFHIR;EHVIEALR;LQNKEHVIEALR;VHIGQVIMSIR | 4 | 0.1355 | 60S ribosomal protein L10 OS=Homo sapiens OX=9606 GN=RPL10 PE=1 SV=4 |
| sp\|P30520\|PURA2_HUMAN | 14.94898881 | 50065.77332 | DGVYFLYEALHGPPK;DGVYFLYEALHGPPKK;TLPGWNTDISNAR;VGIGAFPTEQDNEIGELLQTR | 4 | 0.1096 | Adenylosuccinate synthetase isozyme 2 OS=Homo sapiens OX=9606 GN=ADSS2 PE=1 SV=3 |
| sp\|P30048\|PRDX3_HUMAN | 13.94710583 | 27675.17514 | DLSLDDFKGK;DYGVLLEGSGLALR;GLFIIDPNGVIK;GTAVVNGEFK | 4 | 0.1797 | Thioredoxin-dependent peroxide reductase, mitochondrial OS=Homo sapiens OX=9606 GN=PRDX3 PE=1 SV=3 |
| sp\|O94776\|MTA2_HUMAN | 11.94062595 | 74975.73612 | DLVAQAPLKPK;ELFLSR;QFESLPATHIR;QIDQFLVVAR | 4 | 0.0569 | Metastasis-associated protein MTA2 OS=Homo sapiens OX=9606 GN=MTA2 PE=1 SV=1 |
| sp\|P35606\|COPB2_HUMAN | 11.5676064 | 102422.4826 | DNNQFASASLDR;EAFVVEEWVK;IWHSSTYR;QLAELAISK | 4 | 0.043 | Coatomer subunit beta' OS=Homo sapiens OX=9606 GN=COPB2 PE=1 SV=2 |
| sp\|O00232\|PSD12_HUMAN | 13.71170915 | 52870.57238 | DPNNLLNDWSQK;GSLESPATDVFGSTEEGEKR;ILVAVVK;LAGIINFQRPK | 4 | 0.1096 | 26S proteasome non-ATPase regulatory subunit 12 OS=Homo sapiens OX=9606 GN=PSMD12 PE=1 SV=3 |
| sp\|P31153\|METK2_HUMAN | 12.49298875 | 43633.33372 | DSFPWEVPK;ELLEIVK;FVIGGPQGDAGLTGR;NGTLPWLRPDSK | 4 | 0.1089 | S-adenosylmethionine synthase isoform type-2 OS=Homo sapiens OX=9606 GN=MAT2A PE=1 SV=1 |
| sp\|P08243\|ASNS_HUMAN | 11.8492028 | 64328.6267 | DTYGVRPLFK;EAFSDGITSVK;LFPGFEIETVK;TTAAHGLELR | 4 | 0.0749 | Asparagine synthetase [glutamine-hydrolyzing] OS=Homo sapiens OX=9606 GN=ASNS PE=1 SV=4 |
| sp\|P52907\|CAZA1_HUMAN | 14.77526985 | 32902.32743 | DVQDSLTVSNEAQTAK;EASDPQPEEADGGLK;FITHAPPGEFNEVFNDVR;FTITPPTAQVVGVLK | 4 | 0.2832 | F-actin-capping protein subunit alpha-1 OS=Homo sapiens OX=9606 GN=CAPZA1 PE=1 SV=3 |
| sp\|P46778\|RL21_HUMAN | 11.40752214 | 18553.06058 | EAHFVR;EKGTWVQLK;TNGKEPELLEPIPYEFMA;VYNVTQHAVGIVVNK | 4 | 0.3 | 60S ribosomal protein L21 OS=Homo sapiens OX=9606 GN=RPL21 PE=1 SV=2 |
| sp\|O00203\|AP3B1_HUMAN | 11.2893752 | 121244.4957 | EASADLSPYVR;LLTQYILNLGK;LVYVYLVR;QFAAATIQTIGR | 4 | 0.0384 | AP-3 complex subunit beta-1 OS=Homo sapiens OX=9606 GN=AP3B1 PE=1 SV=3 |
| sp\|P30740\|ILEU_HUMAN | 14.38667051 | 42714.70175 | EATTNAPFR;FAYGYIEDLK;LGVQDLFNSSK;LVLVNAIYFK | 4 | 0.1055 | Leukocyte elastase inhibitor OS=Homo sapiens OX=9606 GN=SERPINB1 PE=1 SV=1 |
| sp\|P63241\|IF5A1_HUMAN | 12.41249548 | 16821.40426 | EDLRLPEGDLGK;NGFVVLK;VHLVGIDIFTGK;VHLVGIDIFTGKK | 4 | 0.2078 | Eukaryotic translation initiation factor 5A-1 OS=Homo sapiens OX=9606 GN=EIF5A PE=1 SV=2 |
| sp\|Q9H223\|EHD4_HUMAN | 12.055146 | 61136.52186 | EGADEEEWVVAK;FGNAFLNR;IILLFDAHK;LFEAEAQDLFR | 4 | 0.0739 | EH domain-containing protein 4 OS=Homo sapiens OX=9606 GN=EHD4 PE=1 SV=1 |
| sp\|P62249\|RS16_HUMAN | 13.6872306 | 16435.04063 | EIKDILIQYDR;GPLQSVQVFGR;LLEPVLLLGK;YVDEASK | 4 | 0.2671 | 40S ribosomal protein S16 OS=Homo sapiens OX=9606 GN=RPS16 PE=1 SV=2 |
| sp\|Q06210\|GFPT1_HUMAN | 12.25548484 | 78756.32023 | EILETLIK;ESQDTSFTTLVER;GSPLLIGVR;GYDVDFPR | 4 | 0.0544 | Glutamine--fructose-6-phosphate aminotransferase [isomerizing] 1 OS=Homo sapiens OX=9606 GN=GFPT1 PE=1 SV=3 |
| sp\|O43390\|HNRPR_HUMAN | 11.55674096 | 70899.24697 | ENILEEFSK;LKDYAFVHFEDR;STAYEDYYYHPPPR;TLIEAGLPQK | 4 | 0.09 | Heterogeneous nuclear ribonucleoprotein R OS=Homo sapiens OX=9606 GN=HNRNPR PE=1 SV=1 |
| sp\|P28370\|SMCA1_HUMAN | 11.96448001 | 122527.0828 | ESELTDEDITTILER;FDWFIK;IYLGLSK;TLQTIALLGYLK | 4 | 0.038 | Probable global transcription activator SNF2L1 OS=Homo sapiens OX=9606 GN=SMARCA1 PE=1 SV=2 |
| sp\|P30040\|ERP29_HUMAN | 13.07083562 | 28975.15499 | ESYPVFYLFR;FDTQYPYGEK;GALPLDTVTFYK;SLNILTAFQK | 4 | 0.1609 | Endoplasmic reticulum resident protein 29 OS=Homo sapiens OX=9606 GN=ERP29 PE=1 SV=4 |
| sp\|Q8N163\|CCAR2_HUMAN | 11.53285223 | 102837.773 | FAEFQYLQPGPPR;ILLTLGIR;VLLLSSPGLEELYR;VQTLSNQPLLK | 4 | 0.0498 | Cell cycle and apoptosis regulator protein 2 OS=Homo sapiens OX=9606 GN=CCAR2 PE=1 SV=2 |
| sp\|P07339\|CATD_HUMAN | 13.18257405 | 44523.62509 | FDGILGMAYPR;QPGITFIAAK;VSTLPAITLK;YYTVFDRDNNR | 4 | 0.1019 | Cathepsin D OS=Homo sapiens OX=9606 GN=CTSD PE=1 SV=1 |
| sp\|P62244\|RS15A_HUMAN | 13.57350565 | 14829.96717 | FDVQLK;FDVQLKDLEK;HGYIGEFEIIDDHR;IVVNLTGR | 4 | 0.2462 | 40S ribosomal protein S15a OS=Homo sapiens OX=9606 GN=RPS15A PE=1 SV=2 |
| sp\|P00505\|AATM_HUMAN | 15.81666253 | 47487.31566 | FVTVQTISGTGALR;IAAAILNTPDLRK;ISVAGVTSSNVGYLAHAIHQVTK;QWLQEVK | 4 | 0.1326 | Aspartate aminotransferase, mitochondrial OS=Homo sapiens OX=9606 GN=GOT2 PE=1 SV=3 |
| sp\|P61019\|RAB2A_HUMAN | 12.53211296 | 23530.77033 | GAAGALLVYDITR;IQEGVFDINNEANGIK;RDTFNHLTTWLEDAR;YIIIGDTGVGK | 4 | 0.2594 | Ras-related protein Rab-2A OS=Homo sapiens OX=9606 GN=RAB2A PE=1 SV=1 |
| sp\|P09429\|HMGB1_HUMAN | 13.73387716 | 24878.16079 | GEHPGLSIGDVAK;HPDASVNFSEFSK;IKGEHPGLSIGDVAK;KHPDASVNFSEFSK | 4 | 0.1349 | High mobility group protein B1 OS=Homo sapiens OX=9606 GN=HMGB1 PE=1 SV=3 |
| sp\|P62277\|RS13_HUMAN | 15.7049241 | 17211.66624 | GLAPDLPEDLYHLIKK;GLTPSQIGVILR;KGLTPSQIGVILR;LTSDDVKEQIYK | 4 | 0.2715 | 40S ribosomal protein S13 OS=Homo sapiens OX=9606 GN=RPS13 PE=1 SV=2 |
| sp\|E9PAV3\|NACAM_HUMAN | 12.79787 | 205294.8886 | IEDLSQQAQLAAAEK;NILFVITKPDVYK;QVTGVTR;SPASDTYIVFGEAK | 4 | 0.0236 | Nascent polypeptide-associated complex subunit alpha, muscle-specific form OS=Homo sapiens OX=9606 GN=NACA PE=1 SV=1 |
| sp\|P49591\|SYSC_HUMAN | 10.36735921 | 58740.0463 | IEQFVYSSPHDNK;KLDLEAWFPGSGAFR;LLIDEAILK;VLDLDLFR | 4 | 0.0875 | Serine--tRNA ligase, cytoplasmic OS=Homo sapiens OX=9606 GN=SARS1 PE=1 SV=3 |
| sp\|Q9Y678\|COPG1_HUMAN | 12.15530788 | 97655.46497 | ILHLLGQEGPK;NTHTLLLAGVFR;TLEEAVGNIVK;VVLEHEEVR | 4 | 0.0492 | Coatomer subunit gamma-1 OS=Homo sapiens OX=9606 GN=COPG1 PE=1 SV=1 |
| sp\|Q15075\|EEA1_HUMAN | 13.72822254 | 162367.1786 | IQAGEGETAVLNQLQEK;ISVLQNNYEK;LSASETSLHR;QDFETLSQETK | 4 | 0.034 | Early endosome antigen 1 OS=Homo sapiens OX=9606 GN=EEA1 PE=1 SV=2 |
| sp\|Q9Y3F4\|STRAP_HUMAN | 14.33570516 | 38413.97349 | IYDLNKPEAEPK;LWQTVVGK;SIAFHSAVSLDPIK;YDYNSGEELESYK | 4 | 0.1343 | Serine-threonine kinase receptor-associated protein OS=Homo sapiens OX=9606 GN=STRAP PE=1 SV=1 |
| sp\|P24534\|EF1B_HUMAN | 14.74299308 | 24748.26175 | LAQYESK;LVPVGYGIK;SIQADGLVWGSSK;SPAGLQVLNDYLADK | 4 | 0.2267 | Elongation factor 1-beta OS=Homo sapiens OX=9606 GN=EEF1B2 PE=1 SV=3 |
| sp\|Q8NC51\|PAIRB_HUMAN | 13.8309424 | 44938.45883 | KPNEGADGQWK;RFEKPLEEK;RPDQQLQGEGK;SAAQAAAQTNSNAAGK | 4 | 0.1152 | Plasminogen activator inhibitor 1 RNA-binding protein OS=Homo sapiens OX=9606 GN=SERBP1 PE=1 SV=2 |
| sp\|P07910\|HNRPC_HUMAN | 13.5104003 | 33649.53837 | KSDVEAIFSK;LKGDDLQAIK;QKVDSLLENLEK;VFIGNLNTLVVK | 4 | 0.1438 | Heterogeneous nuclear ribonucleoproteins C1/C2 OS=Homo sapiens OX=9606 GN=HNRNPC PE=1 SV=4 |
| sp\|P51572\|BAP31_HUMAN | 12.47177666 | 27974.0111 | KYDDVTEK;LDVGNAEVKLEEENR;LLEEHAK;LVTLISQQATLLASNEAFKK | 4 | 0.2033 | B-cell receptor-associated protein 31 OS=Homo sapiens OX=9606 GN=BCAP31 PE=1 SV=3 |
| sp\|P42704\|LPPRC_HUMAN | 11.11346836 | 157805.1136 | LDDLFLK;MVFINNIALAQIK;SSLLLGFR;VYLQNEYK | 4 | 0.0258 | Leucine-rich PPR motif-containing protein, mitochondrial OS=Homo sapiens OX=9606 GN=LRPPRC PE=1 SV=3 |
| sp\|P48681\|NEST_HUMAN | 12.34998503 | 177332.023 | LELQQLQAER;SLEEQDQETLR;SLETEILESLK;SLGAWNLENLR | 4 | 0.0265 | Nestin OS=Homo sapiens OX=9606 GN=NES PE=1 SV=2 |
| sp\|O00303\|EIF3F_HUMAN | 12.33094622 | 37540.15078 | LHPVILASIVDSYER;VIGTLLGTVDK;VSADNTVGR;YAYYDTER | 4 | 0.1204 | Eukaryotic translation initiation factor 3 subunit F OS=Homo sapiens OX=9606 GN=EIF3F PE=1 SV=1 |
| sp\|P78330\|SERB_HUMAN | 11.05261793 | 24991.90311 | LIAEQPPHLTPGIR;LNIPATNVFANR;NVQVFLISGGFR;SIVEHVASK | 4 | 0.2089 | Phosphoserine phosphatase OS=Homo sapiens OX=9606 GN=PSPH PE=1 SV=2 |
| sp\|P39656\|OST48_HUMAN | 11.87484189 | 50768.96922 | LPDVYGVFQFK;SSLNPILFR;TLVLLDNLNVR;WVFKEEGVLR | 4 | 0.0899 | Dolichyl-diphosphooligosaccharide--protein glycosyltransferase 48 kDa subunit OS=Homo sapiens OX=9606 GN=DDOST PE=1 SV=4 |
| sp\|P25789\|PSA4_HUMAN | 9.934004791 | 29465.1796 | LSAEKVEIATLTR;QAYTQFGGK;RPFGVSLLYIGWDK;TTIFSPEGR | 4 | 0.1724 | Proteasome subunit alpha type-4 OS=Homo sapiens OX=9606 GN=PSMA4 PE=1 SV=1 |
| sp\|P61353\|RL27_HUMAN | 13.35122104 | 15787.7473 | NIDDGTSDRPYSHALVAGIDR;VVLVLAGR;VYNYNHLMPTR;YSVDIPLDK | 4 | 0.3603 | 60S ribosomal protein L27 OS=Homo sapiens OX=9606 GN=RPL27 PE=1 SV=2 |
| sp\|P05091\|ALDH2_HUMAN | 9.103869449 | 56345.62455 | AAFQLGSPWR;TEQGPQVDETQFK;VAEQTPLTALYVANLIK | 3 | 0.0774 | Aldehyde dehydrogenase, mitochondrial OS=Homo sapiens OX=9606 GN=ALDH2 PE=1 SV=2 |
| sp\|P12004\|PCNA_HUMAN | 11.12747829 | 28750.29271 | AEDNADTLALVFEAPNQEK;FSASGELGNGNIK;YLNFFTK | 3 | 0.1494 | Proliferating cell nuclear antigen OS=Homo sapiens OX=9606 GN=PCNA PE=1 SV=1 |
| sp\|Q96QK1\|VPS35_HUMAN | 8.24471572 | 91648.93058 | AELAELPLR;IREDLPNLESSEETEQINK;NIIIALIDR | 3 | 0.0465 | Vacuolar protein sorting-associated protein 35 OS=Homo sapiens OX=9606 GN=VPS35 PE=1 SV=2 |
| sp\|P16402\|H13_HUMAN | 9.368742866 | 22336.31858 | ALAAAGYDVEK;SGVSLAALK;SGVSLAALKK | 3 | 0.095 | Histone H1.3 OS=Homo sapiens OX=9606 GN=H1-3 PE=1 SV=2 |
| sp\|O15143\|ARC1B_HUMAN | 9.310542246 | 40923.41421 | ASSEGGTAAGAGLDSLHK;NAYVWTLK;TWKPTLVILR | 3 | 0.0968 | Actin-related protein 2/3 complex subunit 1B OS=Homo sapiens OX=9606 GN=ARPC1B PE=1 SV=3 |
| sp\|Q07955\|SRSF1_HUMAN | 7.700207047 | 27727.81453 | DGTGVVEFVR;DIEDVFYK;IYVGNLPPDIR | 3 | 0.1169 | Serine/arginine-rich splicing factor 1 OS=Homo sapiens OX=9606 GN=SRSF1 PE=1 SV=2 |
| sp\|P22392\|NDKB_HUMAN | 8.071428295 | 17286.94074 | DRPFFPGLVK;NIIHGSDSVK;SAEKEISLWFKPEELVDYK | 3 | 0.5066 | Nucleoside diphosphate kinase B OS=Homo sapiens OX=9606 GN=NME2 PE=1 SV=1 |
| sp\|Q16576\|RBBP7_HUMAN | 11.42560438 | 47790.17873 | DYALHWLVLGTHTSDEQNHLVVAR;IGEEQSAEDAEDGPPELLFIHGGHTAK;LHTFESHKDEIFQVHWSPHNETILASSGTDR | 3 | 0.2588 | Histone-binding protein RBBP7 OS=Homo sapiens OX=9606 GN=RBBP7 PE=1 SV=1 |
| sp\|P27708\|PYR1_HUMAN | 8.839353153 | 242829.4515 | EATAGNPGGQTVR;LALGIPLPELR;LSLDDLLQR | 3 | 0.0148 | CAD protein OS=Homo sapiens OX=9606 GN=CAD PE=1 SV=3 |
| sp\|Q08J23\|NSUN2_HUMAN | 9.070709062 | 86415.80101 | EILFYDR;ILLTQENPFFR;LAQEGIYTLYPFINSR | 3 | 0.0443 | RNA cytosine C(5)-methyltransferase NSUN2 OS=Homo sapiens OX=9606 GN=NSUN2 PE=1 SV=2 |
| sp\|P30050\|RL12_HUMAN | 10.16073483 | 17807.54009 | EILGTAQSVGCNVDGR;IGPLGLSPK;QAQIEVVPSASALIIK | 3 | 0.2485 | 60S ribosomal protein L12 OS=Homo sapiens OX=9606 GN=RPL12 PE=1 SV=1 |
| sp\|P50914\|RL14_HUMAN | 10.16073483 | 17807.54009 | EILGTAQSVGCNVDGR;IGPLGLSPK;QAQIEVVPSASALIIK | 3 | 0.2485 | 60S ribosomal protein L14 OS=Homo sapiens OX=9606 GN=RPL14 PE=1 SV=4 |
| sp\|P62263\|RS14_HUMAN | 9.652273408 | 16262.53252 | ELGITALHIK;IEDVTPIPSDSTR;TPGPGAQSALR | 3 | 0.2252 | 40S ribosomal protein S14 OS=Homo sapiens OX=9606 GN=RPS14 PE=1 SV=3 |
| sp\|P49915\|GUAA_HUMAN | 9.100970291 | 76666.95561 | ELGLPEELVSR;EPPTDVTPTFLTTGVLSTLR;VVYIFGPPVK | 3 | 0.0592 | GMP synthase [glutamine-hydrolyzing] OS=Homo sapiens OX=9606 GN=GMPS PE=1 SV=1 |
| sp\|P78344\|IF4G2_HUMAN | 8.596207526 | 102296.8113 | ENPLLPEEEEQR;GVILLIVDK;LDLIHESILHK | 3 | 0.0353 | Eukaryotic translation initiation factor 4 gamma 2 OS=Homo sapiens OX=9606 GN=EIF4G2 PE=1 SV=1 |
| sp\|O95433\|AHSA1_HUMAN | 9.902681853 | 38250.24876 | ETFLTSPEELYR;VFTTQELVQAFTHAPATLEADR;YYFEGIK | 3 | 0.1213 | Activator of 90 kDa heat shock protein ATPase homolog 1 OS=Homo sapiens OX=9606 GN=AHSA1 PE=1 SV=1 |
| sp\|Q92499\|DDX1_HUMAN | 9.404799539 | 82379.75402 | FGFGFGGTGK;GHVDILAPTVQELAALEK;VPVDEFDGK | 3 | 0.05 | ATP-dependent RNA helicase DDX1 OS=Homo sapiens OX=9606 GN=DDX1 PE=1 SV=2 |
| sp\|P06576\|ATPB_HUMAN | 10.98603969 | 56524.60469 | FTQAGSEVSALLGR;IGLFGGAGVGK;LVLEVAQHLGESTVR | 3 | 0.0756 | ATP synthase subunit beta, mitochondrial OS=Homo sapiens OX=9606 GN=ATP5F1B PE=1 SV=3 |
| sp\|P55735\|SEC13_HUMAN | 9.393343891 | 35518.11382 | GQGSVSASVTEGQQNEQ;LEAHSDWVR;NGGQILIADLR | 3 | 0.1149 | Protein SEC13 homolog OS=Homo sapiens OX=9606 GN=SEC13 PE=1 SV=3 |
| sp\|Q9BUJ2\|HNRL1_HUMAN | 10.32553257 | 95679.28973 | HLPSTEPDPHVVR;NYILDQTNVYGSAQR;QGAPTSFLPPEASQLKPDR | 3 | 0.0549 | Heterogeneous nuclear ribonucleoprotein U-like protein 1 OS=Homo sapiens OX=9606 GN=HNRNPUL1 PE=1 SV=2 |
| sp\|Q92688\|AN32B_HUMAN | 9.065487949 | 28770.30799 | IFGGLDMLAEK;KLELSENR;LPNLTHLNLSGNK | 3 | 0.2032 | Acidic leucine-rich nuclear phosphoprotein 32 family member B OS=Homo sapiens OX=9606 GN=ANP32B PE=1 SV=1 |
| sp\|P54709\|AT1B3_HUMAN | 9.413023438 | 31492.09376 | IIGLKPEGVPR;LFIYNPTTGEFLGR;SDPTSYAGYIEDLKK | 3 | 0.1434 | Sodium/potassium-transporting ATPase subunit beta-3 OS=Homo sapiens OX=9606 GN=ATP1B3 PE=1 SV=1 |
| sp\|P62906\|RL10A_HUMAN | 10.81352818 | 24815.53467 | ILGPGLNK;KYDAFLASESLIK;YDAFLASESLIK | 3 | 0.0968 | 60S ribosomal protein L10a OS=Homo sapiens OX=9606 GN=RPL10A PE=1 SV=2 |
| sp\|Q07666\|KHDR1_HUMAN | 8.869542324 | 48197.18199 | ILGPQGNTIK;KDDEENYLDLFSHK;SGSMDPSGAHPSVR | 3 | 0.0858 | KH domain-containing, RNA-binding, signal transduction-associated protein 1 OS=Homo sapiens OX=9606 GN=KHDRBS1 PE=1 SV=1 |
| sp\|Q9HB71\|CYBP_HUMAN | 10.16073483 | 26193.6443 | ISNYGWDQSDK;KAELLDNEKPAAVVAPITTGYTVK;SFDLLVK | 3 | 0.1842 | Calcyclin-binding protein OS=Homo sapiens OX=9606 GN=CACYBP PE=1 SV=2 |
| sp\|P41091\|IF2G_HUMAN | 9.909667626 | 51077.2171 | LIGWGQIR;LTPLSHEVISR;VGQEIEVRPGIVSK | 3 | 0.0699 | Eukaryotic translation initiation factor 2 subunit 3 OS=Homo sapiens OX=9606 GN=EIF2S3 PE=1 SV=3 |
| sp\|P42766\|RL35_HUMAN | 10.3842117 | 14542.55999 | QLDDLKVELSQLR;VLTVINQTQK;YKPLDLRPK | 3 | 0.2602 | 60S ribosomal protein L35 OS=Homo sapiens OX=9606 GN=RPL35 PE=1 SV=2 |
| sp\|Q13177\|PAK2_HUMAN | 8.729401867 | 58005.88205 | IISIFSGTEK;LLQTSNITK;LSPIFR | 3 | 0.0477 | Serine/threonine-protein kinase PAK 2 OS=Homo sapiens OX=9606 GN=PAK2 PE=1 SV=3 |
| sp\|P09936\|UCHL1_HUMAN | 9.093019185 | 24808.45738 | LGFEDGSVLK;QFLSETEK;QIEELKGQEVSPK | 3 | 0.139 | Ubiquitin carboxyl-terminal hydrolase isozyme L1 OS=Homo sapiens OX=9606 GN=UCHL1 PE=1 SV=2 |
| sp\|Q6DD88\|ATLA3_HUMAN | 10.87430126 | 60503.42233 | ALASILLQDHIR;DQHSFELDEK;IYQGEDLPHPK | 3 | 0.061 | Atlastin-3 OS=Homo sapiens OX=9606 GN=ATL3 PE=1 SV=1 |
| sp\|Q5T4S7\|UBR4_HUMAN | 8.457071087 | 573476.4424 | FVPLILAR;LLLIWQHK;TPVFIFER | 3 | 0.0046 | E3 ubiquitin-protein ligase UBR4 OS=Homo sapiens OX=9606 GN=UBR4 PE=1 SV=1 |
| sp\|P12955\|PEPD_HUMAN | 9.220611596 | 54512.90616 | AFTPFSGPK;AVYEAVLR;VPLALFALNR | 3 | 0.0548 | Xaa-Pro dipeptidase OS=Homo sapiens OX=9606 GN=PEPD PE=1 SV=3 |
| sp\|Q16658\|FSCN1_HUMAN | 9.39577853 | 54496.05128 | FLIVAHDDGR;LINRPIIVFR;YLTAEAFGFK | 3 | 0.0609 | Fascin OS=Homo sapiens OX=9606 GN=FSCN1 PE=1 SV=3 |
| sp\|Q92538\|GBF1_HUMAN | 9.27891784 | 206314.6081 | AASIYSSWAEEQR;ENYVWNVLLHR;LYLEAFR | 3 | 0.0167 | Golgi-specific brefeldin A-resistance guanine nucleotide exchange factor 1 OS=Homo sapiens OX=9606 GN=GBF1 PE=1 SV=2 |
| sp\|Q9UBT2\|SAE2_HUMAN | 9.779054378 | 71179.2449 | ESVLQFYPK;QFILVMNALDNR;QFLFQK | 3 | 0.0422 | SUMO-activating enzyme subunit 2 OS=Homo sapiens OX=9606 GN=UBA2 PE=1 SV=2 |
| sp\|P22061\|PIMT_HUMAN | 10.16073483 | 24620.57082 | KDDPTLLSSGR;SGGASHSELIHNLR;VQLVVGDGR | 3 | 0.1498 | Protein-L-isoaspartate(D-aspartate) O-methyltransferase OS=Homo sapiens OX=9606 GN=PCMT1 PE=1 SV=4 |
| sp\|P07099\|HYEP_HUMAN | 7.548871579 | 52914.96301 | DVELLYPVK;FLSVLER;FSTWTNTEFR | 3 | 0.0571 | Epoxide hydrolase 1 OS=Homo sapiens OX=9606 GN=EPHX1 PE=1 SV=1 |
| sp\|Q13148\|TADBP_HUMAN | 12.0906401 | 44711.30571 | FGGNPGGFGNQGGFGNSR;FTEYETQVK;TSDLIVLGLPWK | 3 | 0.0942 | TAR DNA-binding protein 43 OS=Homo sapiens OX=9606 GN=TARDBP PE=1 SV=1 |
| sp\|Q9Y5B9\|SP16H_HUMAN | 9.257004435 | 119838.4176 | AASITSEVFNK;APGEQTVPALNLQNAFR;QDSLVINLNR | 3 | 0.0363 | FACT complex subunit SPT16 OS=Homo sapiens OX=9606 GN=SUPT16H PE=1 SV=1 |
| sp\|Q14444\|CAPR1_HUMAN | 10.00785473 | 78318.02343 | LNQDQLDAVSK;QILGVIDK;YQEVTNNLEFAK | 3 | 0.0437 | Caprin-1 OS=Homo sapiens OX=9606 GN=CAPRIN1 PE=1 SV=2 |
| sp\|Q92522\|H1X_HUMAN | 9.883873928 | 22473.53213 | ALVQNDTLLQVK;GAPAAATAPAPTAHK;RGAPAAATAPAPTAHK | 3 | 0.1315 | Histone H1.10 OS=Homo sapiens OX=9606 GN=H1-10 PE=1 SV=1 |
| sp\|Q15181\|IPYR_HUMAN | 8.937994876 | 32639.15482 | DKDFAIDIIK;DPLNPIK;LKPGYLEATVDWFR | 3 | 0.1073 | Inorganic pyrophosphatase OS=Homo sapiens OX=9606 GN=PPA1 PE=1 SV=2 |
| sp\|A5YKK6\|CNOT1_HUMAN | 8.277592278 | 266767.8926 | DAIAALGLLQK;QLAVYEEFAR;SPVTFLSDLR | 3 | 0.013 | CCR4-NOT transcription complex subunit 1 OS=Homo sapiens OX=9606 GN=CNOT1 PE=1 SV=2 |
| sp\|O15372\|EIF3H_HUMAN | 9.026774585 | 39905.05414 | GEPPLPEEDLSK;LFKPPQPPAR;SAVADKHELLSLASSNHLGK | 3 | 0.1193 | Eukaryotic translation initiation factor 3 subunit H OS=Homo sapiens OX=9606 GN=EIF3H PE=1 SV=1 |
| sp\|P00390\|GSHR_HUMAN | 9.044692924 | 56220.94976 | ALLTPVAIAAGR;LGIQTDDKGHIIVDEFQNTNVK;YGIENVK | 3 | 0.0785 | Glutathione reductase, mitochondrial OS=Homo sapiens OX=9606 GN=GSR PE=1 SV=2 |
| sp\|Q8N8S7\|ENAH_HUMAN | 10.29839712 | 66469.89355 | ERLEQEQLER;VHIYHHTGNNTFR;YNQATQTFHQWR | 3 | 0.0592 | Protein enabled homolog OS=Homo sapiens OX=9606 GN=ENAH PE=1 SV=2 |
| sp\|Q15758\|AAAT_HUMAN | 10.54647501 | 56562.25033 | GPAGDATVASEK;NIFPSNLVSAAFR;SYSTTYEER | 3 | 0.0628 | Neutral amino acid transporter B(0) OS=Homo sapiens OX=9606 GN=SLC1A5 PE=1 SV=2 |
| sp\|P35659\|DEK_HUMAN | 7.440565664 | 42647.91981 | IHFFLSK;LLYNRPGTVSSLK;SGVNSELVK | 3 | 0.0773 | Protein DEK OS=Homo sapiens OX=9606 GN=DEK PE=1 SV=1 |
| sp\|Q9BZZ5\|API5_HUMAN | 10.09866077 | 58967.91055 | ELPQFATGENLPR;LAAQFIPK;STVTLSWKPVQK | 3 | 0.063 | Apoptosis inhibitor 5 OS=Homo sapiens OX=9606 GN=API5 PE=1 SV=3 |
| sp\|O43592\|XPOT_HUMAN | 10.02267344 | 109893.298 | ALAYFEQLK;LAQVSPELLLASVR;TAYLFSR | 3 | 0.0312 | Exportin-T OS=Homo sapiens OX=9606 GN=XPOT PE=1 SV=2 |
| sp\|P30043\|BLVRB_HUMAN | 11.42560438 | 22105.42603 | LPSEGPRPAHVVVGDVLQAADVDK;LQAVTDDHIR;TVAGQDAVIVLLGTR | 3 | 0.2379 | Flavin reductase (NADPH) OS=Homo sapiens OX=9606 GN=BLVRB PE=1 SV=3 |
| sp\|Q15637\|SF01_HUMAN | 9.540534978 | 68285.90143 | FQRPGDPQSAQDK;ILRPWQSSETR;QGIETPEDQNDLR | 3 | 0.0579 | Splicing factor 1 OS=Homo sapiens OX=9606 GN=SF1 PE=1 SV=4 |
| sp\|P32969\|RL9_HUMAN | 8.041799252 | 21849.79683 | FLDGIYVSEK;KFLDGIYVSEK;RDFNHINVELSLLGK | 3 | 0.1354 | 60S ribosomal protein L9 OS=Homo sapiens OX=9606 GN=RPL9 PE=1 SV=1 |
| sp\|Q92973\|TNPO1_HUMAN | 9.720949364 | 102288.78 | ATVGILITTIASK;ESQSPDTTIQR;SLSGLILK | 3 | 0.0356 | Transportin-1 OS=Homo sapiens OX=9606 GN=TNPO1 PE=1 SV=2 |
| sp\|P61289\|PSME3_HUMAN | 11.20212752 | 29487.5586 | MWVQLLIPR;SNQQLVDIIEK;TVESEAASYLDQISR | 3 | 0.1378 | Proteasome activator complex subunit 3 OS=Homo sapiens OX=9606 GN=PSME3 PE=1 SV=1 |
| sp\|Q99714\|HCD2_HUMAN | 10.17428616 | 26906.11196 | DLAPIGIR;GQTHTLEDFQR;LVGQGASAVLLDLPNSGGEAQAK | 3 | 0.1609 | 3-hydroxyacyl-CoA dehydrogenase type-2 OS=Homo sapiens OX=9606 GN=HSD17B10 PE=1 SV=3 |
| sp\|Q13428\|TCOF_HUMAN | 8.195559292 | 152015.3201 | ASEAQPPVAR;NPQNSTVLAR;SPAGPAATPAQAQAASTPR | 3 | 0.0262 | Treacle protein OS=Homo sapiens OX=9606 GN=TCOF1 PE=1 SV=3 |
| sp\|Q9Y4L1\|HYOU1_HUMAN | 9.980939161 | 111266.2079 | AEAGPEGVAPAPEGEK;EVEEEPGIHSLK;TPVIVTLK | 3 | 0.036 | Hypoxia up-regulated protein 1 OS=Homo sapiens OX=9606 GN=HYOU1 PE=1 SV=1 |
| sp\|P50570\|DYN2_HUMAN | 9.404799539 | 98003.16435 | GISPVPINLR;NLVDSYVAIINK;RPLILQLIFSK | 3 | 0.0379 | Dynamin-2 OS=Homo sapiens OX=9606 GN=DNM2 PE=1 SV=2 |
| sp\|P40121\|CAPG_HUMAN | 9.21119121 | 38474.47244 | EGNPEEDLTADK;QAALQVAEGFISR;YQEGGVESAFHK | 3 | 0.1063 | Macrophage-capping protein OS=Homo sapiens OX=9606 GN=CAPG PE=1 SV=2 |
| sp\|P35613\|BASI_HUMAN | 12.46699707 | 42174.05979 | GGVVLKEDALPGQK;RKPEDVLDDDDAGSAPLK;SESVPPVTDWAWYK | 3 | 0.1195 | Basigin OS=Homo sapiens OX=9606 GN=BSG PE=1 SV=2 |
| sp\|Q9H0U4\|RAB1B_HUMAN | 9.17722638 | 22157.22849 | GAHGIIVVYDVTDQESYANVK;MGPGAASGGERPNLK;TITSSYYR | 3 | 0.2189 | Ras-related protein Rab-1B OS=Homo sapiens OX=9606 GN=RAB1B PE=1 SV=1 |
| sp\|O43242\|PSMD3_HUMAN | 8.30472773 | 60939.48697 | EPQLAFHQR;ISLADIAQK;VYEFLDKLDVVR | 3 | 0.0562 | 26S proteasome non-ATPase regulatory subunit 3 OS=Homo sapiens OX=9606 GN=PSMD3 PE=1 SV=2 |
| sp\|P51148\|RAB5C_HUMAN | 8.708574798 | 23467.78528 | GVDLQENNPASR;NEPQNATGAPGR;QASPNIVIALAGNK | 3 | 0.2778 | Ras-related protein Rab-5C OS=Homo sapiens OX=9606 GN=RAB5C PE=1 SV=2 |
| sp\|P55157\|MTP_HUMAN | 9.09945278 | 99288.77152 | ETVMIITGTLVR;NFLAFIQHLR;SNLNIFQYIGK | 3 | 0.0369 | Microsomal triglyceride transfer protein large subunit OS=Homo sapiens OX=9606 GN=MTTP PE=1 SV=1 |
| sp\|O95373\|IPO7_HUMAN | 9.896116298 | 119439.8686 | AFAVGVQQVLLK;EYNEFAEVFLK;NPVWYQALTHGLNEEQR | 3 | 0.0385 | Importin-7 OS=Homo sapiens OX=9606 GN=IPO7 PE=1 SV=1 |
| sp\|P62834\|RAP1A_HUMAN | 6.943575025 | 20973.70768 | LVVLGSGGVGK;YDPTIEDSYR;YDPTIEDSYRK | 3 | 0.1196 | Ras-related protein Rap-1A OS=Homo sapiens OX=9606 GN=RAP1A PE=1 SV=1 |
| sp\|O60841\|IF2P_HUMAN | 11.04294826 | 138741.6585 | ILPQYIFNSR;LKEGDTIIVPGVEGPIVTQIR;LLQAQGVEVPSK | 3 | 0.0352 | Eukaryotic translation initiation factor 5B OS=Homo sapiens OX=9606 GN=EIF5B PE=1 SV=4 |
| sp\|P18085\|ARF4_HUMAN | 7.579785049 | 20497.69278 | IQEVADELQK;IRPLWK;LGLQSLR | 3 | 0.2722 | ADP-ribosylation factor 4 OS=Homo sapiens OX=9606 GN=ARF4 PE=1 SV=3 |
| sp\|Q9Y285\|SYFA_HUMAN | 6.913053564 | 57527.50875 | LDAEPRPPPTQEAA;LGITQLR;LLAEVTLK | 3 | 0.0571 | Phenylalanine--tRNA ligase alpha subunit OS=Homo sapiens OX=9606 GN=FARSA PE=1 SV=3 |
| sp\|Q15459\|SF3A1_HUMAN | 9.505260337 | 88830.50908 | IGEEEIQKPEEK;LQYEGIFIK;LTAQFVAR | 3 | 0.0366 | Splicing factor 3A subunit 1 OS=Homo sapiens OX=9606 GN=SF3A1 PE=1 SV=1 |
| sp\|P11387\|TOP1_HUMAN | 10.27247327 | 90668.90645 | AVALYFIDK;LEVQATDREENK;TYNASITLQQQLK | 3 | 0.0444 | DNA topoisomerase 1 OS=Homo sapiens OX=9606 GN=TOP1 PE=1 SV=2 |
| sp\|Q9Y383\|LC7L2_HUMAN | 8.353274036 | 46485.59076 | LAETQEEISAEVAAK;LHLGFIEIR;VHELNEEIGK | 3 | 0.0867 | Putative RNA-binding protein Luc7-like 2 OS=Homo sapiens OX=9606 GN=LUC7L2 PE=1 SV=2 |
| sp\|Q13492\|PICAL_HUMAN | 8.225277198 | 70709.9808 | NTLFNLSNFLDK;TTNSSWVVVFK;VAEQVGIDR | 3 | 0.0491 | Phosphatidylinositol-binding clathrin assembly protein OS=Homo sapiens OX=9606 GN=PICALM PE=1 SV=2 |
| sp\|Q99733\|NP1L4_HUMAN | 7.469464924 | 42796.89007 | LDNVPHTPSSYIETLPK;LTDQVMQNPR;VLAALQER | 3 | 0.12 | Nucleosome assembly protein 1-like 4 OS=Homo sapiens OX=9606 GN=NAP1L4 PE=1 SV=1 |
| sp\|P04083\|ANXA1_HUMAN | 10.3842117 | 38689.97848 | GTDVNVFNTILTTR;GVDEATIIDILTKR;TPAQFDADELR | 3 | 0.1127 | Annexin A1 OS=Homo sapiens OX=9606 GN=ANXA1 PE=1 SV=2 |
| sp\|Q16698\|DECR_HUMAN | 9.832908578 | 36044.80056 | ATAEQISSQTGNK;EQWDTIEELIR;FNVIQPGPIK | 3 | 0.1015 | 2,4-dienoyl-CoA reductase, mitochondrial OS=Homo sapiens OX=9606 GN=DECR1 PE=1 SV=1 |
| sp\|O00410\|IPO5_HUMAN | 11.20212752 | 123549.8574 | ATAAFILANEHNVALFK;ITFLLQAIR;VSDILHSIFSSYK | 3 | 0.0356 | Importin-5 OS=Homo sapiens OX=9606 GN=IPO5 PE=1 SV=4 |
| sp\|P17301\|ITA2_HUMAN | 8.042265528 | 129213.8522 | FIFSLK;TQVGLIQYANNPR;VYLFTIK | 3 | 0.022 | Integrin alpha-2 OS=Homo sapiens OX=9606 GN=ITGA2 PE=1 SV=1 |
| sp\|P15170\|ERF3A_HUMAN | 10.16073483 | 55720.29589 | HFTILDAPGHK;HLIVLINK;KEHVNVVFIGHVDAGK | 3 | 0.0701 | Eukaryotic peptide chain release factor GTP-binding subunit ERF3A OS=Homo sapiens OX=9606 GN=GSPT1 PE=1 SV=1 |
| sp\|O15031\|PLXB2_HUMAN | 7.726994885 | 204997.176 | FWVNILK;IQPETGPLGGGIR;VLYAVFSR | 3 | 0.0152 | Plexin-B2 OS=Homo sapiens OX=9606 GN=PLXNB2 PE=1 SV=3 |
| sp\|P17980\|PRS6A_HUMAN | 11.20212752 | 49172.45554 | EKFENLGIQPPK;LAGPQLVQMFIGDGAK;VDILDPALLR | 3 | 0.0866 | 26S proteasome regulatory subunit 6A OS=Homo sapiens OX=9606 GN=PSMC3 PE=1 SV=3 |
| sp\|P56192\|SYMC_HUMAN | 10.16073483 | 101051.9042 | ADKNEVAAEVAK;GLESLPPLRPQQNPVLPVAGER;TLPGSDWTPNAQFITR | 3 | 0.0556 | Methionine--tRNA ligase, cytoplasmic OS=Homo sapiens OX=9606 GN=MARS1 PE=1 SV=2 |
| sp\|P38919\|IF4A3_HUMAN | 7.414481923 | 46841.18141 | EQIYDVYR;LDYGQHVVAGTPGR;VLISTDVWAR | 3 | 0.1363 | Eukaryotic initiation factor 4A-III OS=Homo sapiens OX=9606 GN=EIF4A3 PE=1 SV=4 |
| sp\|Q14157\|UBP2L_HUMAN | 8.988154931 | 114465.2693 | DGSLASNPYSGDLTK;GGSTTGSQFLEQFK;IDLAVLLGK | 3 | 0.035 | Ubiquitin-associated protein 2-like OS=Homo sapiens OX=9606 GN=UBAP2L PE=1 SV=2 |
| sp\|P12268\|IMDH2_HUMAN | 9.832908578 | 55769.64593 | FGVPVIADGGIQNVGHIAK;NLIDAGVDALR;REDLVVAPAGITLK | 3 | 0.0856 | Inosine-5'-monophosphate dehydrogenase 2 OS=Homo sapiens OX=9606 GN=IMPDH2 PE=1 SV=2 |
| sp\|P0DP23\|CALM1_HUMAN | 10.23198049 | 16826.83404 | DTDSEEEIR;DTDSEEEIREAFR;VFDKDGNGYISAAELR | 3 | 0.1946 | Calmodulin-1 OS=Homo sapiens OX=9606 GN=CALM1 PE=1 SV=1 |
| sp\|P62495\|ERF1_HUMAN | 11.31386595 | 49000.14894 | GFGGIGGILR;LSVLGAITSVQQR;YFDEISQDTGK | 3 | 0.0778 | Eukaryotic peptide chain release factor subunit 1 OS=Homo sapiens OX=9606 GN=ETF1 PE=1 SV=3 |
| sp\|Q14839\|CHD4_HUMAN | 6.842425631 | 217867.0701 | IGVMSLIR;VGGNIEVLGFNAR;YAILNEPFK | 3 | 0.0157 | Chromodomain-helicase-DNA-binding protein 4 OS=Homo sapiens OX=9606 GN=CHD4 PE=1 SV=2 |
| sp\|P47756\|CAPZB_HUMAN | 10.21861906 | 31330.76878 | KLEVEANNAFDQYR;LEVEANNAFDQYR;STLNEIYFGK | 3 | 0.0866 | F-actin-capping protein subunit beta OS=Homo sapiens OX=9606 GN=CAPZB PE=1 SV=4 |
| sp\|Q8N1G4\|LRC47_HUMAN | 8.581369577 | 63433.73419 | APGPGLAQGLPQLHSLVLR;ELLLTGPGLEER;TAATLATHELR | 3 | 0.072 | Leucine-rich repeat-containing protein 47 OS=Homo sapiens OX=9606 GN=LRRC47 PE=1 SV=1 |
| sp\|P00491\|PNPH_HUMAN | 9.39577853 | 32097.15553 | FEVGDIMLIR;LVFGFLNGR;VFGFSLITNK | 3 | 0.1003 | Purine nucleoside phosphorylase OS=Homo sapiens OX=9606 GN=PNP PE=1 SV=2 |
| sp\|Q09666\|AHNK_HUMAN | 9.896116298 | 628699.3707 | ADIDVSGPK;ADVDVSGPK;AEGPEVDVNLPK | 3 | 0.0051 | Neuroblast differentiation-associated protein AHNAK OS=Homo sapiens OX=9606 GN=AHNAK PE=1 SV=2 |
| sp\|Q07021\|C1QBP_HUMAN | 12.35525864 | 31342.60783 | AFVDFLSDEIKEER;EVSFQSTGESEWK;VEEQEPELTSTPNFVVEVIK | 3 | 0.1667 | Complement component 1 Q subcomponent-binding protein, mitochondrial OS=Homo sapiens OX=9606 GN=C1QBP PE=1 SV=1 |
| sp\|P23588\|IF4B_HUMAN | 9.048376567 | 69110.28439 | AASIFGGAKPVDTAAR;DSDKTDTDWR;VAPAQPSEEGPGR | 3 | 0.0638 | Eukaryotic translation initiation factor 4B OS=Homo sapiens OX=9606 GN=EIF4B PE=1 SV=2 |
| sp\|Q15691\|MARE1_HUMAN | 9.573033355 | 29980.1893 | KPLTSSSAAPQRPISTQR;LEHEYIQNFK;QGQETAVAPSLVAPALNKPK | 3 | 0.1791 | Microtubule-associated protein RP/EB family member 1 OS=Homo sapiens OX=9606 GN=MAPRE1 PE=1 SV=3 |
| sp\|P14866\|HNRPL_HUMAN | 9.549429122 | 64092.36274 | IEYAKPTR;NPNGPYPYTLK;TDNAGDQHGGGGGGGGGAGAAGGGGGGENYDDPHK | 3 | 0.0917 | Heterogeneous nuclear ribonucleoprotein L OS=Homo sapiens OX=9606 GN=HNRNPL PE=1 SV=2 |
| sp\|O75695\|XRP2_HUMAN | 7.917720055 | 39615.45643 | APDFLPLLNK;GFFLVQTK;QYSWDQR | 3 | 0.0714 | Protein XRP2 OS=Homo sapiens OX=9606 GN=RP2 PE=1 SV=4 |
| sp\|Q13310\|PABP4_HUMAN | 8.959417896 | 70738.08962 | EFSPFGSITSAK;FSPAGPVLSIR;IVGSKPLYVALAQR | 3 | 0.1165 | Polyadenylate-binding protein 4 OS=Homo sapiens OX=9606 GN=PABPC4 PE=1 SV=1 |
| sp\|P11586\|C1TC_HUMAN | 9.216407383 | 101495.3038 | GALALAQAVQR;GVPTGFILPIR;IFHELTQTDK | 3 | 0.0342 | C-1-tetrahydrofolate synthase, cytoplasmic OS=Homo sapiens OX=9606 GN=MTHFD1 PE=1 SV=3 |
| sp\|P62266\|RS23_HUMAN | 10.60968273 | 15797.71686 | GHAVGDIPGVR;KGHAVGDIPGVR;VANVSLLALYK | 3 | 0.1608 | 40S ribosomal protein S23 OS=Homo sapiens OX=9606 GN=RPS23 PE=1 SV=3 |
| sp\|P48163\|MAOX_HUMAN | 7.691677628 | 64109.06754 | IWLVDSK;KIWLVDSK;TATVYPEPQNK | 3 | 0.0332 | NADP-dependent malic enzyme OS=Homo sapiens OX=9606 GN=ME1 PE=1 SV=1 |
| sp\|Q13867\|BLMH_HUMAN | 9.161987777 | 52528.01898 | AQHVFQHAVPQEGKPITNQK;IGPITPLEFYR;VAALIQK | 3 | 0.0835 | Bleomycin hydrolase OS=Homo sapiens OX=9606 GN=BLMH PE=1 SV=1 |
| sp\|P05198\|IF2A_HUMAN | 10.72616447 | 36089.37476 | INLIAPPR;TAWVFDDKYK;VVTDTDETELAR | 3 | 0.0952 | Eukaryotic translation initiation factor 2 subunit 1 OS=Homo sapiens OX=9606 GN=EIF2S1 PE=1 SV=3 |
| sp\|Q7L2H7\|EIF3M_HUMAN | 11.31386595 | 42475.8044 | ALKDPNAFLFDHLLTLKPVK;QQWQQLYDTLNAWK;WISDWNLTTEK | 3 | 0.1203 | Eukaryotic translation initiation factor 3 subunit M OS=Homo sapiens OX=9606 GN=EIF3M PE=1 SV=1 |
| sp\|Q3ZCQ8\|TIM50_HUMAN | 12.35525864 | 39621.53934 | IPDEFDNDPILVQQLR;QNLFLGSLTSR;TVLEHYALEDDPLAAFK | 3 | 0.1246 | Mitochondrial import inner membrane translocase subunit TIM50 OS=Homo sapiens OX=9606 GN=TIMM50 PE=1 SV=2 |
