# Supplementary material for: Protein arginine methyltransferase 3 promotes glycolysis and hepatocellular carcinoma growth by enhancing arginine methylation of lactate dehydrogenase A
Source: Clin Transl Med. 2022 Jan 28;12(1):e686. doi: 10.1002/ctm2.686 (PMC8797063; doi:10.1002/ctm2.686)
Supplement: Supplementary file 25 — Table S4 [file CTM2-12-e686-s012.docx]

| **Supplementary Table S4. Differentially metabolites between control and PRMT3-overexpressing Huh7 cells in positive ion mode** | | | | | | | | | | | | |
| --- | --- | --- | --- | --- | --- | --- | --- | --- | --- | --- | --- | --- |
| **MS2 name** | **MS2 score** | **MS1 name** | **MS1 ppm** | **rt** | **mz** | **Mean PRMT3** | **Mean control** | **VIP** | **P-VALUE** | **Q-VALUE** | **FOLD CHANGE** | **LOG_FOLDCHANGE** |
| Adenosine 2'-phosphate | 0.999999538 |  |  | 484.9255 | 348.0699987 | 0.643064688 | 0.438123632 | 1.722076996 | 0.018130659 | 0.181493446 | 1.467769918 | 0.553625835 |
| 1-Pyrroline | 0.999747154 |  |  | 326.469 | 70.06575725 | 0.978176734 | 1.385448732 | 1.443417297 | 0.035584993 | 0.21508809 | 0.706036039 | -0.502186269 |
| Cytosine | 0.999579154 |  |  | 217.924 | 112.0508172 | 4.098354963 | 2.976564407 | 1.775846228 | 0.004547518 | 0.108000888 | 1.376874276 | 0.461396831 |
| 2-Furancarboxaldehyde | 0.998233538 |  |  | 450.92 | 97.02885374 | 0.956788834 | 0.600353216 | 1.902575804 | 0.003878619 | 0.102081714 | 1.593709851 | 0.672388998 |
| 1H-Pyrrole-2-carboxaldehyde | 0.997479 |  |  | 455.7455 | 96.04486548 | 0.338982133 | 0.231789564 | 2.131918426 | 0.000705902 | 0.059273643 | 1.462456405 | 0.548393619 |
| Cytarabine | 0.996565462 |  |  | 255.8205 | 244.0927249 | 0.284727579 | 0.150536596 | 1.638681458 | 0.033523352 | 0.212572352 | 1.891417677 | 0.919467985 |
| L-Acetylcarnitine | 0.994892077 |  |  | 322.662 | 204.1231048 | 12.35232367 | 19.10615435 | 1.853500835 | 0.010143735 | 0.150733637 | 0.6465102 | -0.629254963 |
| Methyl 2-aminobenzoate | 0.992643538 |  |  | 271.7815 | 152.0708902 | 0.162520571 | 0.138652085 | 1.838041389 | 0.009198341 | 0.145000029 | 1.172146602 | 0.229153021 |
| Uridine 5'-monophosphate | 0.991382 |  |  | 451.4195 | 325.0425971 | 0.824079813 | 0.491865892 | 1.667894225 | 0.012211049 | 0.161235617 | 1.675415649 | 0.744519054 |
| D-Arabinose 5-phosphate | 0.987224692 |  |  | 455.0245 | 213.0161499 | 1.478021627 | 0.829949997 | 2.115759061 | 0.000839623 | 0.060528905 | 1.780856236 | 0.832571056 |
| Proline betaine | 0.984053462 |  |  | 364.24 | 158.1177454 | 0.052535518 | 0.075219044 | 1.548392111 | 0.047548729 | 0.231863303 | 0.698433741 | -0.517804838 |
| sec-Butylamine | 0.979778769 |  |  | 262.354 | 74.09713795 | 0.121171575 | 0.084066008 | 1.651879373 | 0.045067377 | 0.228403799 | 1.441386102 | 0.52745684 |
| Leukoaminochrome | 0.979514385 |  |  | 258.925 | 152.0707636 | 0.138689789 | 0.115089145 | 1.70830155 | 0.036898221 | 0.21657139 | 1.205064036 | 0.269109812 |
| Glucose 1-phosphate | 0.975008231 |  |  | 468.497 | 283.0192559 | 0.309058351 | 0.168787513 | 1.847316641 | 0.002857412 | 0.090267073 | 1.831049857 | 0.872671074 |
| S-Adenosylmethionine | 0.967177538 |  |  | 587.797 | 399.1446482 | 0.121489399 | 0.071346693 | 1.480665978 | 0.038845659 | 0.218685988 | 1.702803502 | 0.767911962 |
| Glucose 6-phosphate | 0.965567462 |  |  | 476.889 | 261.0373225 | 0.235800849 | 0.149332295 | 1.941726211 | 0.008875082 | 0.142876097 | 1.579034521 | 0.659042712 |
| Styrene | 0.965139923 |  |  | 34.5043 | 105.0702125 | 1.868159093 | 1.43059437 | 1.896635465 | 0.003424233 | 0.097272224 | 1.305862188 | 0.385002652 |
| L-Hexanoylcarnitine | 0.957540615 |  |  | 245.6155 | 260.1858142 | 0.120626081 | 0.205841048 | 2.185664569 | 7.61441E-07 | 0.001469743 | 0.586015675 | -0.77098884 |
| Mannose 6-phosphate | 0.957375692 |  |  | 485.4345 | 283.0192764 | 0.566373136 | 0.328427921 | 2.047139684 | 0.001784429 | 0.075160338 | 1.72449752 | 0.786176054 |
| NAD | 0.954189154 |  |  | 447.61 | 664.1148576 | 0.297286221 | 0.211475668 | 1.52713537 | 0.032657775 | 0.211440964 | 1.405770337 | 0.491360918 |
| 1-Butylamine | 0.953064385 |  |  | 76.5139 | 74.09705521 | 0.051185595 | 0.042148819 | 1.447699023 | 0.048943175 | 0.233694957 | 1.21440163 | 0.280245632 |
| LysoPE(20:4(5Z,8Z,11Z,14Z)/0:0) | 0.951616692 |  |  | 211.994 | 502.293023 | 0.13096188 | 0.069872639 | 1.915132101 | 0.004894211 | 0.110635943 | 1.874294171 | 0.906347402 |
| 4-Hydroxy-2-butenoic acid gamma-lactone | 0.950346846 |  |  | 322.7155 | 85.02902169 | 0.294231654 | 0.45637463 | 1.856568438 | 0.007303148 | 0.131113496 | 0.644715185 | -0.633266131 |
| N-Acetylglutamic acid | 0.941088231 |  |  | 396.935 | 190.0714297 | 0.258015897 | 0.326820511 | 1.372625425 | 0.048375344 | 0.232958374 | 0.789472778 | -0.341038573 |
| Citicoline | 0.940872154 |  |  | 454.702 | 489.1155447 | 0.15686156 | 0.112084365 | 1.63250024 | 0.040487523 | 0.221248255 | 1.399495461 | 0.484906808 |
| Phenylalanyl-Arginine | 0.932322692 |  |  | 330.968 | 322.1876176 | 0.033989531 | 0.054196036 | 1.530841934 | 0.028113204 | 0.204622569 | 0.627158993 | -0.673096863 |
| Asymmetric dimethylarginine | 0.928437462 |  |  | 514.1315 | 203.1504016 | 1.678277627 | 1.17756084 | 1.74314949 | 0.005362229 | 0.113815063 | 1.425215217 | 0.511179792 |
| Cytidine monophosphate | 0.920254462 |  |  | 468.8165 | 324.0583992 | 0.364432897 | 0.207144832 | 1.678803566 | 0.009568404 | 0.147325533 | 1.759314448 | 0.815013363 |
| Mulberrofuran M | 0.917389 |  |  | 453.606 | 591.1228731 | 0.014834327 | 0.012242451 | 1.537002853 | 0.03925812 | 0.219143608 | 1.211712193 | 0.27704707 |
| Triethanolamine | 0.901219615 |  |  | 150.173 | 150.1124048 | 0.103125614 | 0.047682889 | 1.710627895 | 0.019608915 | 0.185935799 | 2.162738381 | 1.112859158 |
| alpha-Methylstyrene | 0.872939769 |  |  | 35.3834 | 119.0857287 | 70.01124351 | 53.64738414 | 1.622555327 | 0.021664275 | 0.19141832 | 1.305026231 | 0.384078805 |
| Indole | 0.853787385 |  |  | 34.50285 | 118.0653865 | 2.971430955 | 2.438892901 | 1.831259676 | 0.005663195 | 0.115662114 | 1.218352374 | 0.284931452 |
| UDP-N-acetyl-alpha-D-galactosamine | 0.849887154 |  |  | 440.257 | 608.0875939 | 0.20538495 | 0.156070287 | 1.531920271 | 0.028045547 | 0.204508243 | 1.315977273 | 0.396134574 |
| 2-Amino-3,8-dimethyl-3H-imidazo[4,5-f]quinoxaline | 0.848323615 |  |  | 386.156 | 214.1076437 | 0.044335938 | 0.106918717 | 1.942483005 | 0.005196015 | 0.112731454 | 0.414669564 | -1.269965935 |
| Acrylamide | 0.847549692 |  |  | 452.805 | 72.04507982 | 0.167785751 | 0.12012864 | 2.039164005 | 0.001185749 | 0.066704608 | 1.396717309 | 0.482040053 |
| 8-Deoxy-11,13-dihydroxygrosheimin | 0.837356846 |  |  | 35.38 | 281.1384502 | 9.382056545 | 7.244402818 | 1.7284738 | 0.014045304 | 0.168770518 | 1.295076596 | 0.373037428 |
| N-a-Acetyl-L-arginine | 0.826232308 |  |  | 386.786 | 217.1298003 | 0.037622146 | 0.055458968 | 1.557624732 | 0.048984931 | 0.233748629 | 0.678378026 | -0.559838655 |
| 6-Phosphonoglucono-D-lactone | 0.826004077 |  |  | 474.372 | 259.0214531 | 0.132855601 | 0.078473537 | 2.097234967 | 0.001680855 | 0.074015255 | 1.692998757 | 0.759580914 |
| Theogallin | 0.809219154 |  |  | 429.763 | 345.0837978 | 0.392610655 | 0.334518047 | 1.693048262 | 0.019927604 | 0.186837848 | 1.173660611 | 0.231015283 |
| Deoxyadenosine monophosphate | 0.806536692 |  |  | 431.645 | 332.0759768 | 0.129764482 | 0.08502511 | 2.062220601 | 0.001102491 | 0.065057163 | 1.526190105 | 0.609934678 |
| Perilloside C | 0.800549 |  |  | 449.871 | 317.193434 | 0.020700062 | 0.008329759 | 1.622607706 | 0.025702825 | 0.200270377 | 2.485073449 | 1.313288493 |
| Butyrylcarnitine | 0.789936385 |  |  | 277.208 | 232.1546721 | 4.04860265 | 6.09826897 | 1.919433069 | 0.001774248 | 0.075052208 | 0.663893749 | -0.590975727 |
| Quercetin | 0.775544231 |  |  | 476.9135 | 303.0485989 | 0.068519311 | 0.049027994 | 1.952136689 | 0.000932081 | 0.061207658 | 1.397554858 | 0.482904914 |
| (E)-Monocrotophos | 0.773668615 |  |  | 459.697 | 224.0669933 | 0.164558604 | 0.132397163 | 1.901583239 | 0.00249538 | 0.085663003 | 1.242916388 | 0.313729249 |
| Ethylbenzene | 0.769878 |  |  | 35.376 | 107.0859896 | 6.023386599 | 4.700834506 | 1.855896825 | 0.015029555 | 0.172280509 | 1.281344108 | 0.357657967 |
| PC(20:1(11Z)/20:4(5Z,8Z,11Z,14Z)) | 0.74567 |  |  | 155.5755 | 836.6164215 | 1.180805531 | 0.994823873 | 1.544493965 | 0.04054648 | 0.221347477 | 1.186949332 | 0.247258351 |
| N-Acetylputrescine | 0.725079077 |  |  | 354.434 | 131.118194 | 0.064415964 | 0.034532846 | 1.59039751 | 0.030075743 | 0.207764231 | 1.865353449 | 0.899449019 |
| 3-Dehydroxycarnitine | 0.714608231 |  |  | 393.6915 | 146.1175066 | 8.356344202 | 12.23872182 | 1.740720513 | 0.016347613 | 0.176503974 | 0.68277916 | -0.550509071 |
| 2-Methylbutyroylcarnitine | 0.701742154 |  |  | 261.435 | 246.1696555 | 11.87880887 | 15.80253023 | 1.504790295 | 0.048009054 | 0.232476508 | 0.751702968 | -0.411765396 |
| PC(20:2(11Z,14Z)/14:0) | 0.661079308 |  |  | 139.3725 | 758.5713858 | 4.564136072 | 3.488519717 | 2.040908498 | 0.001486667 | 0.071561766 | 1.308330307 | 0.387726816 |
| PC(20:2(11Z,14Z)/15:0) | 0.659299308 |  |  | 161.062 | 772.5854001 | 3.139524465 | 2.789321732 | 1.621929003 | 0.023490023 | 0.195697147 | 1.125551215 | 0.170631704 |
| 2,5-Dichloro-carboxymethylenebut-2-en-4-olide | 0.648262692 |  |  | 382.7085 | 208.9389032 | 0.046729578 | 0.034589461 | 1.80020588 | 0.011637039 | 0.158561823 | 1.350977347 | 0.434003484 |
| PC(22:1(13Z)/15:0) | 0.645714769 |  |  | 160.129 | 802.6276086 | 0.051146957 | 0.115869344 | 1.321241935 | 0.037474829 | 0.217195888 | 0.441419234 | -1.179778602 |
| PC(20:4(8Z,11Z,14Z,17Z)/20:3(8Z,11Z,14Z)) | 0.645366231 |  |  | 154.7635 | 832.5844222 | 2.039080629 | 1.530635164 | 1.579495793 | 0.02380655 | 0.196389741 | 1.332179397 | 0.413788375 |
| Aspartyl-Aspartate | 0.644675231 |  |  | 469.904 | 249.0719918 | 0.196390022 | 0.153920226 | 1.611381984 | 0.025641599 | 0.20015182 | 1.275920832 | 0.351538815 |
| Inosine 2'-phosphate | 0.638391846 |  |  | 457.1425 | 349.0549301 | 0.107245617 | 0.068173683 | 1.814545468 | 0.008551143 | 0.140655204 | 1.573123413 | 0.653631856 |
| PC(22:5(7Z,10Z,13Z,16Z,19Z)/16:0) | 0.636993231 |  |  | 156.551 | 808.5855063 | 5.220207918 | 3.885281394 | 1.956418861 | 0.010700402 | 0.153812162 | 1.343585544 | 0.426088179 |
| Dihydrowyerol | 0.634924 |  |  | 34.49675 | 263.1273914 | 0.913610139 | 0.659345774 | 1.610244905 | 0.018171696 | 0.181599734 | 1.385631296 | 0.47054342 |
| PC(22:6(4Z,7Z,10Z,13Z,16Z,19Z)/20:1(11Z)) | 0.634424769 |  |  | 153.861 | 860.6153966 | 0.256780388 | 0.19824702 | 2.07540421 | 0.00041179 | 0.054230129 | 1.295254716 | 0.373235837 |
| Tyrosyl-Lysine | 0.615814231 |  |  | 443.194 | 310.1758856 | 0.234066009 | 0.120450603 | 1.985431881 | 0.002049732 | 0.078768744 | 1.94325311 | 0.958473825 |
| PC(22:6(4Z,7Z,10Z,13Z,16Z,19Z)/18:0) | 0.610458231 |  |  | 155.5775 | 834.6002661 | 2.232775866 | 1.790745896 | 1.72397724 | 0.028507741 | 0.205280925 | 1.246841258 | 0.3182778 |
| Arginyl-Serine | 0.606044923 |  |  | 454.9205 | 262.1507179 | 0.018334008 | 0.028523719 | 1.599414465 | 0.024739565 | 0.198354478 | 0.6427636 | -0.637639864 |
| 3-beta-Hydroxy-4-beta-methyl-5-alpha-cholest-7-ene-4-alpha-carbaldehyde | 0.603208846 |  |  | 30.9114 | 429.3724336 | 0.194495445 | 0.152277373 | 1.469038925 | 0.045228641 | 0.228636878 | 1.277244549 | 0.353034779 |
| Cohibin C | 0.602270077 |  |  | 167.186 | 577.5187612 | 0.06812472 | 0.129200061 | 1.43959906 | 0.044341932 | 0.227340394 | 0.527280863 | -0.923356457 |
| 8-Oxo-dGMP | 0.596735077 |  |  | 473.515 | 364.0654215 | 0.08586948 | 0.073398991 | 1.585815651 | 0.034283339 | 0.213527962 | 1.169900012 | 0.226385231 |
| PC(22:4(7Z,10Z,13Z,16Z)/22:5(4Z,7Z,10Z,13Z,16Z)) | 0.578189846 |  |  | 150.3 | 884.6149064 | 0.021245826 | 0.015308233 | 1.976957653 | 0.001506382 | 0.071831517 | 1.387869263 | 0.472871673 |
| PC(20:5(5Z,8Z,11Z,14Z,17Z)/15:0) | 0.566754231 |  |  | 159.283 | 766.5356943 | 1.927172399 | 2.353392253 | 1.366074626 | 0.048926154 | 0.233673059 | 0.818891282 | -0.288256166 |
| Arginyl-Gamma-glutamate | 0.565981846 |  |  | 458.368 | 303.1775392 | 0.018525149 | 0.03107454 | 1.854802148 | 0.006174305 | 0.120843927 | 0.596152002 | -0.74624787 |
| Thiolutin | 0.565653769 |  |  | 424.268 | 229.0108917 | 0.29773179 | 0.174024872 | 2.118803072 | 0.000252424 | 0.04803655 | 1.710857688 | 0.774719759 |
| Sorbitol-6-phosphate | 0.565046154 |  |  | 501.077 | 263.0538986 | 0.138608077 | 0.079113648 | 1.453760871 | 0.038246154 | 0.218007178 | 1.752012206 | 0.809012826 |
| Glycerylphosphorylethanolamine | 0.547887 |  |  | 409.0445 | 216.0633206 | 0.073740361 | 0.101833443 | 1.668102885 | 0.008232233 | 0.138372364 | 0.72412715 | -0.465685052 |
| 3,5,6-Trihydroxy-5-(hydroxymethyl)-2-methoxy-2-cyclohexen-1-one | 0.545785231 |  |  | 476.847 | 205.0720499 | 0.563444309 | 0.28797826 | 1.764688991 | 0.012340408 | 0.161815501 | 1.956551547 | 0.96831312 |
| LysoPI(18:0/0:0) | 0.528119692 |  |  | 264.1965 | 601.3340805 | 0.062421505 | 0.04596674 | 1.640837687 | 0.039142781 | 0.219016421 | 1.357971122 | 0.441452801 |
| PS(20:5(5Z,8Z,11Z,14Z,17Z)/18:0) | 0.527140923 |  |  | 212.007 | 810.5272933 | 0.327102248 | 0.217989216 | 1.726875928 | 0.018645519 | 0.183080021 | 1.500543254 | 0.585484906 |
| hesperetin 3'-O-sulfate | 0.525648538 |  |  | 408.6275 | 383.0409868 | 0.086569582 | 0.069324964 | 1.979797106 | 0.001689186 | 0.074111201 | 1.248750478 | 0.32048523 |
| 5-Aminoimidazole ribonucleotide | 0.480874769 |  |  | 403.178 | 296.0656605 | 0.204356955 | 0.271930998 | 1.448039458 | 0.045446829 | 0.228950346 | 0.751502978 | -0.412149274 |
| Isoniazid alpha-ketoglutaric acid | 0.466786615 |  |  | 255.884 | 266.0744442 | 0.11753638 | 0.070932566 | 1.978430391 | 0.001106193 | 0.065133826 | 1.657015752 | 0.728587317 |
| PE(18:2(9Z,12Z)/14:0) | 0.421398385 |  |  | 39.766 | 688.4890961 | 0.042435716 | 0.066180663 | 1.709478312 | 0.022994758 | 0.194585509 | 0.641210208 | -0.641130702 |
| PE(P-18:1(9Z)/20:4(5Z,8Z,11Z,14Z)) | 0.399139462 |  |  | 154.6955 | 750.5432004 | 0.073745247 | 0.115000011 | 1.847061996 | 0.001164559 | 0.066300087 | 0.64126296 | -0.641012018 |
| PS(20:1(11Z)/15:0) | 0.332255846 |  |  | 215.359 | 776.5448739 | 0.03670209 | 0.052809782 | 1.722845184 | 0.008954287 | 0.143404804 | 0.694986581 | -0.524942972 |
| PE(P-18:1(11Z)/18:4(6Z,9Z,12Z,15Z)) | 0.320216769 |  |  | 156.596 | 722.5096133 | 0.036168812 | 0.05254621 | 1.741273673 | 0.004911327 | 0.110759471 | 0.688323906 | -0.538840477 |
|  |  |  |  | 371.551 | 214.1077077 | 0.060638199 | 0.107573298 | 1.878050524 | 0.005519744 | 0.114799474 | 0.563691922 | -0.827021202 |
|  |  |  |  | 479.9535 | 229.0834932 | 0.285611225 | 0.204008372 | 1.81800653 | 0.036221394 | 0.215817793 | 1.399997569 | 0.485424322 |
|  |  |  |  | 279.108 | 158.1175109 | 0.080035794 | 0.117970156 | 1.720018223 | 0.048802072 | 0.233513088 | 0.678441032 | -0.559704668 |
|  |  |  |  | 294.7805 | 89.07158848 | 0.113799369 | 0.161899201 | 1.872203912 | 0.004498305 | 0.10760507 | 0.702902598 | -0.508603309 |
|  |  |  |  | 155.6645 | 484.2823274 | 0.001178617 | 0.00414624 | 1.47043781 | 0.019518685 | 0.185676674 | 0.284261668 | -1.814708528 |
|  |  |  |  | 25.3947 | 457.2775662 | 0.010726503 | 0.014979078 | 1.345507685 | 0.045593601 | 0.229160002 | 0.716099026 | -0.481768989 |
|  |  |  |  | 276.525 | 312.1160672 | 0.033364933 | 0.05544092 | 1.671854052 | 0.006398302 | 0.123026444 | 0.601810585 | -0.732618613 |
|  |  |  |  | 30.9118 | 391.284725 | 1.741891715 | 1.492282865 | 1.525401496 | 0.038671568 | 0.218490492 | 1.167266445 | 0.223133915 |
|  |  |  |  | 258.63 | 496.2664434 | 0.018376018 | 0.010930175 | 1.901498198 | 0.008042581 | 0.136966789 | 1.681218951 | 0.749507624 |
|  |  |  |  | 35.37955 | 415.2119217 | 111.5780777 | 88.38261095 | 1.670119853 | 0.039512743 | 0.219578285 | 1.262443783 | 0.336219146 |
|  |  |  |  | 292.928 | 276.1804728 | 0.008037202 | 0.014100999 | 1.800145324 | 0.008008422 | 0.136709683 | 0.569973985 | -0.811032021 |
|  |  |  |  | 255.059 | 103.0618646 | 0.422365616 | 0.208498478 | 1.898484531 | 0.001816461 | 0.075494584 | 2.025749159 | 1.018455541 |
|  |  |  |  | 427.8765 | 285.1192576 | 0.129719455 | 0.092903698 | 1.91487519 | 0.005392549 | 0.114007688 | 1.396278706 | 0.481586941 |
|  |  |  |  | 450.295 | 327.0796934 | 0.061139883 | 0.045311745 | 1.880094195 | 0.002046858 | 0.078729002 | 1.349316467 | 0.432228756 |
|  |  |  |  | 439.3205 | 391.1829957 | 0.011520089 | 0.008550711 | 1.640676996 | 0.025562112 | 0.199997266 | 1.347266798 | 0.430035575 |
|  |  |  |  | 317.2515 | 518.2734717 | 0.036806119 | 0.01624961 | 1.869536659 | 0.006719503 | 0.126025448 | 2.265046351 | 1.179540573 |
|  |  |  |  | 458.5405 | 232.1403788 | 0.050414412 | 0.080879896 | 1.828574505 | 0.00541966 | 0.114178638 | 0.623324394 | -0.68194492 |
|  |  |  |  | 325.652 | 211.071486 | 0.052759989 | 0.084262632 | 1.674883727 | 0.026437493 | 0.201660558 | 0.626137438 | -0.67544873 |
|  |  |  |  | 35.3811 | 295.1174795 | 1.547340049 | 1.168857936 | 1.62733924 | 0.022470733 | 0.19337058 | 1.323805059 | 0.40469069 |
|  |  |  |  | 46.9334 | 506.2763118 | 0.033198658 | 0.02157667 | 1.666061668 | 0.007697666 | 0.134313191 | 1.538636756 | 0.621652678 |
|  |  |  |  | 447.312 | 292.1139482 | 0.245795092 | 0.186534464 | 1.846901693 | 0.008552386 | 0.140663916 | 1.317692651 | 0.398013904 |
|  |  |  |  | 323.157 | 346.054493 | 0.0299006 | 0.019974342 | 1.487685952 | 0.035776079 | 0.215309396 | 1.496950419 | 0.582026439 |
|  |  |  |  | 484.5585 | 225.0162265 | 1.249677061 | 0.649762781 | 2.08352886 | 0.001730932 | 0.074581606 | 1.923282001 | 0.943570313 |
|  |  |  |  | 503.83 | 334.0535337 | 0.059721028 | 0.033554918 | 1.328296947 | 0.047212099 | 0.231409398 | 1.779799562 | 0.831714776 |
|  |  |  |  | 428.1115 | 284.0857007 | 0.025488929 | 0.016919362 | 1.694411825 | 0.029959548 | 0.207587159 | 1.506494639 | 0.591195539 |
|  |  |  |  | 279.093 | 172.0967564 | 0.050409029 | 0.102765732 | 2.061718646 | 0.000526983 | 0.056764125 | 0.49052372 | -1.027605192 |
|  |  |  |  | 253.95 | 645.3011116 | 0.012641292 | 0.007566335 | 1.66244045 | 0.033784996 | 0.212905223 | 1.670728652 | 0.74047744 |
|  |  |  |  | 259.351 | 524.2985726 | 0.020405156 | 0.015549401 | 1.860653829 | 0.008998999 | 0.143700854 | 1.312279263 | 0.392074769 |
|  |  |  |  | 473.453 | 219.0055376 | 0.227107027 | 0.120720747 | 1.641207538 | 0.039888624 | 0.220228893 | 1.881259287 | 0.911698704 |
|  |  |  |  | 41.7388 | 129.102492 | 0.040907607 | 0.029264014 | 1.750268228 | 0.009157479 | 0.144736439 | 1.397880949 | 0.483241498 |
|  |  |  |  | 153.088 | 846.5977143 | 0.038082943 | 0.028116946 | 1.843106545 | 0.006752469 | 0.126324916 | 1.354448043 | 0.437705052 |
|  |  |  |  | 212.644 | 438.2977842 | 0.056598677 | 0.037112487 | 1.711177816 | 0.016317477 | 0.176412898 | 1.525057523 | 0.60886366 |
|  |  |  |  | 211.877 | 550.3879329 | 0.196351139 | 0.149698284 | 1.683193739 | 0.034287606 | 0.213533232 | 1.311645891 | 0.391378284 |
|  |  |  |  | 210.697 | 361.2732984 | 0.085258209 | 0.060988519 | 1.530689945 | 0.03416728 | 0.213384232 | 1.39793867 | 0.483301069 |
|  |  |  |  | 470.609 | 230.0423079 | 0.190275721 | 0.102991223 | 2.149542049 | 0.003445281 | 0.097511798 | 1.847494538 | 0.885570099 |
|  |  |  |  | 213.034 | 536.371535 | 0.054508957 | 0.041060559 | 1.708364236 | 0.010027816 | 0.150066237 | 1.327525927 | 0.408740038 |
|  |  |  |  | 476.5585 | 243.0265397 | 0.655497586 | 0.371943903 | 1.887732393 | 0.015593417 | 0.174149416 | 1.762356047 | 0.81750542 |
|  |  |  |  | 154.761 | 607.5081139 | 0.006579144 | 0.012609952 | 1.629520564 | 0.013194039 | 0.165449565 | 0.521742241 | -0.938590854 |
|  |  |  |  | 167.1195 | 716.5211135 | 1.385544142 | 1.87367912 | 1.386474591 | 0.043447019 | 0.225994004 | 0.739477815 | -0.435421229 |
|  |  |  |  | 444.026 | 392.0337367 | 0.015401258 | 0.010502876 | 1.490104127 | 0.030658184 | 0.208635982 | 1.466384746 | 0.552263684 |
|  |  |  |  | 325.14 | 227.0430238 | 0.002743088 | 0.015765576 | 1.603976055 | 0.036701749 | 0.216354958 | 0.173992263 | -2.522904943 |
|  |  |  |  | 429.099 | 231.0438049 | 0.010641951 | 0.006243633 | 1.57922195 | 0.005183824 | 0.112650084 | 1.704448418 | 0.76930494 |
|  |  |  |  | 252.1275 | 570.2833388 | 0.014065493 | 0.008281907 | 1.797612868 | 0.034257697 | 0.213496273 | 1.69833992 | 0.764125241 |
|  |  |  |  | 25.3948 | 363.1718777 | 0.050636028 | 0.027568025 | 1.440222383 | 0.035250816 | 0.214696415 | 1.836766641 | 0.877168345 |
|  |  |  |  | 337.4185 | 248.1492488 | 0.199143544 | 0.427184059 | 1.097076336 | 0.042175601 | 0.224012353 | 0.466177378 | -1.101049098 |
|  |  |  |  | 472.0955 | 298.0295776 | 0.095066177 | 0.045777413 | 2.04717327 | 0.007501389 | 0.132744102 | 2.076704856 | 1.054296193 |
|  |  |  |  | 353.259 | 330.0368364 | 0.006466211 | 0.004935409 | 1.485206661 | 0.014594178 | 0.170768591 | 1.310167188 | 0.389750924 |
|  |  |  |  | 416.602 | 178.0087667 | 0.073294355 | 0.045116841 | 1.662664531 | 0.022819071 | 0.19418272 | 1.624545357 | 0.700036024 |
|  |  |  |  | 277.0555 | 254.1365193 | 0.016137077 | 0.021271665 | 1.556093756 | 0.017582189 | 0.180037867 | 0.758618408 | -0.398553716 |
|  |  |  |  | 511.2935 | 394.0877305 | 0.242174272 | 0.16726236 | 1.894915127 | 0.007430571 | 0.132166986 | 1.447870713 | 0.533932784 |
|  |  |  |  | 264.207 | 565.3136862 | 0.013883294 | 0.009522897 | 1.585178536 | 0.042177971 | 0.224016124 | 1.457885587 | 0.543877503 |
|  |  |  |  | 253.293 | 548.2975633 | 0.014724581 | 0.010062413 | 1.729102669 | 0.023618169 | 0.19597919 | 1.463325003 | 0.549250226 |
|  |  |  |  | 88.8641 | 476.3063316 | 0.038140783 | 0.01370871 | 1.111737851 | 0.017467022 | 0.179723707 | 2.782229994 | 1.476241686 |
|  |  |  |  | 389.638 | 241.1548781 | 0.038413074 | 0.052143257 | 1.819090607 | 0.005477783 | 0.114541125 | 0.736683437 | -0.440883288 |
|  |  |  |  | 6.04342 | 139.9149425 | 0.131137967 | 0.114942226 | 1.733888538 | 0.01952911 | 0.185706698 | 1.140903311 | 0.190176532 |
|  |  | 3-(Phosphoacetylamido)-L-alanine | 15.00449542 | 6.05213 | 243.1347095 | 0.010266643 | 0.008392984 | 1.360332536 | 0.045315377 | 0.228761748 | 1.223241046 | 0.290708723 |
|  |  |  |  | 18.6038 | 166.0974575 | 0.038717312 | 0.031970483 | 1.742115194 | 0.01187989 | 0.159713605 | 1.211033087 | 0.276238282 |
|  |  |  |  | 21.6915 | 265.0035243 | 0.009872434 | 0.008032435 | 1.650498182 | 0.035701152 | 0.215222847 | 1.229071173 | 0.297568462 |
|  |  |  |  | 22.59445 | 330.2640259 | 0.022626052 | 0.018174259 | 2.016785504 | 0.002025105 | 0.078425809 | 1.244950489 | 0.316088368 |
|  |  | Phenolic phosphate | -2.487495668 | 24.4876 | 175.0979435 | 0.031429372 | 0.020013242 | 1.460314895 | 0.04853311 | 0.233164286 | 1.57042883 | 0.651158564 |
|  |  | 3-(Phosphoacetylamido)-L-alanine | 16.38368084 | 24.6746 | 243.1350435 | 0.015068921 | 0.01005555 | 1.406048255 | 0.037256582 | 0.216961367 | 1.498567567 | 0.583584133 |
|  |  |  |  | 25.3947 | 420.2376696 | 0.018861739 | 0.01027704 | 1.464103209 | 0.031005367 | 0.209143385 | 1.835327959 | 0.876037884 |
|  |  |  |  | 25.39475 | 492.3237281 | 0.069204501 | 0.038951577 | 1.394727199 | 0.04443946 | 0.227484793 | 1.776680346 | 0.82918414 |
|  |  |  |  | 25.398 | 476.3008182 | 0.087929729 | 0.049363623 | 1.414780536 | 0.041952893 | 0.223656591 | 1.781265719 | 0.832902746 |
|  |  |  |  | 25.3982 | 475.2978762 | 0.279968343 | 0.158499069 | 1.424575626 | 0.03962225 | 0.219768706 | 1.76637216 | 0.820789339 |
|  |  |  |  | 26.3464 | 609.135561 | 0.013077168 | 0.0097051 | 1.819254113 | 0.014862402 | 0.171707382 | 1.347453106 | 0.430235065 |
|  |  |  |  | 26.3665 | 610.1411918 | 0.005260961 | 0.004046855 | 1.753453607 | 0.013368474 | 0.166153438 | 1.300012133 | 0.378525088 |
|  |  |  |  | 29.9834 | 647.4595485 | 0.00580818 | 0.003042856 | 1.259197404 | 0.030427451 | 0.208293762 | 1.908792175 | 0.932660034 |
|  |  |  |  | 30.02335 | 664.4565639 | 0.496521581 | 0.402978979 | 1.405740997 | 0.049411851 | 0.234293576 | 1.232127746 | 0.301151841 |
|  |  |  |  | 30.0298 | 633.6360568 | 0.001560425 | 0.008993985 | 1.647764782 | 0.006656572 | 0.12544954 | 0.173496541 | -2.527021198 |
|  |  |  |  | 30.03335 | 663.4534224 | 1.13770974 | 0.914701757 | 1.474266223 | 0.037131397 | 0.216825834 | 1.243804039 | 0.314759207 |
|  |  |  |  | 30.0433 | 632.6343474 | 0.008031321 | 0.020000262 | 1.382867791 | 0.038082073 | 0.217836846 | 0.401560819 | -1.316309584 |
|  |  |  |  | 30.90005 | 531.2731105 | 1.030158389 | 0.780190571 | 1.420118343 | 0.04907091 | 0.233858936 | 1.320393283 | 0.400967704 |
|  |  | Permethrin | -17.82344116 | 30.9061 | 392.2880025 | 0.452128782 | 0.381087847 | 1.596517683 | 0.028041764 | 0.204501838 | 1.186416165 | 0.24661016 |
|  |  | 2-Bromo-1H-indole-3-carboxaldehyde | -3.177267123 | 30.9159 | 225.0605647 | 0.002574149 | 0.005908287 | 1.787086132 | 0.039856353 | 0.220173367 | 0.435684515 | -1.198644255 |
|  |  |  |  | 30.93625 | 430.3798427 | 0.145924392 | 0.120933187 | 1.553087189 | 0.041438593 | 0.222824853 | 1.206652996 | 0.271010852 |
|  |  |  |  | 30.9562 | 224.0625269 | 0.002842099 | 0.005870619 | 1.604836677 | 0.019207211 | 0.184769194 | 0.484122595 | -1.046555666 |
|  |  |  |  | 32.7351 | 376.2597788 | 1.166026403 | 0.769018344 | 1.638429919 | 0.023816165 | 0.196410566 | 1.516253041 | 0.600510539 |
|  |  |  |  | 34.5034 | 106.0656253 | 0.557269878 | 0.441978974 | 1.851408449 | 0.006513101 | 0.12411551 | 1.26085156 | 0.334398437 |
|  |  |  |  | 34.5049 | 77.03920718 | 0.088579157 | 0.067721252 | 1.520128043 | 0.029596723 | 0.207027285 | 1.307996454 | 0.38735863 |
|  |  |  |  | 35.3746 | 144.0808314 | 0.349890682 | 0.237931987 | 1.465619536 | 0.011006213 | 0.15541931 | 1.470549151 | 0.556355005 |
|  |  | 3-Fluoro-D-alanine | -15.85122974 | 35.3773 | 108.0891792 | 0.502696183 | 0.392988097 | 1.813628219 | 0.00552359 | 0.114823013 | 1.279163891 | 0.35520112 |
|  |  |  |  | 35.3779 | 103.0545797 | 0.966748091 | 0.717815519 | 1.593345275 | 0.026978332 | 0.202647151 | 1.346791851 | 0.429526897 |
|  |  | Bis(4'-chlorophenyl)acetate | 2.397698304 | 35.3781 | 282.1419507 | 1.473699144 | 1.1612963 | 1.55275068 | 0.03775722 | 0.217496052 | 1.269012175 | 0.343705911 |
|  |  |  |  | 35.3791 | 136.0838654 | 1.408569988 | 1.068599242 | 1.767705051 | 0.029473356 | 0.206834479 | 1.318146161 | 0.39851035 |
|  |  |  |  | 35.37925 | 135.0803905 | 14.71498006 | 11.24851607 | 1.676471734 | 0.032106355 | 0.210695046 | 1.308170782 | 0.387550898 |
|  |  |  |  | 35.37955 | 176.1071416 | 0.551839722 | 0.447936693 | 1.543723005 | 0.037166388 | 0.216863792 | 1.231959184 | 0.300954459 |
|  |  |  |  | 35.3806 | 91.05467781 | 8.087758251 | 6.310790039 | 1.771759056 | 0.022613324 | 0.193705226 | 1.281576189 | 0.357919249 |
|  |  |  |  | 35.3808 | 434.2448449 | 1.10285168 | 0.887399756 | 1.604869112 | 0.026675569 | 0.202098593 | 1.242790155 | 0.313582718 |
|  |  |  |  | 37.139 | 769.5923001 | 0.203735449 | 0.296565041 | 1.530116937 | 0.016359346 | 0.17653937 | 0.686984036 | -0.541651521 |
|  |  | 3,4,5-trihydroxy-6-{3,4,8,9,10-pentahydroxy-6-oxo-2-[5,6,12,13,14-pentahydroxy-4-(hydroxymethyl)-9-oxo-3,8-dioxatricyclo[8.4.0.0,]tetradeca-1(14),10,12-trien-11-yl]-6H-benzo[c]chromene-1-carbonyloxy}oxane-2-carboxylic acid | 20.32828411 | 38.0089 | 809.5867133 | 1.188341399 | 0.92163443 | 1.603502618 | 0.0441554 | 0.227062954 | 1.289384772 | 0.36668285 |
|  |  | Cyanidin 3-O-(2-O-beta-D-glucuronosyl)-beta-D-glucoside | 7.038913362 | 38.0097 | 626.5206795 | 0.019358787 | 0.029415087 | 1.696890215 | 0.012386211 | 0.162018904 | 0.658124393 | -0.603567799 |
|  |  |  |  | 38.0099 | 759.5708825 | 11.30324732 | 9.1502264 | 1.78714591 | 0.010541487 | 0.152954019 | 1.235297011 | 0.304857961 |
|  |  |  |  | 38.0111 | 772.5861191 | 1.567965073 | 1.350283823 | 1.689049184 | 0.015092221 | 0.17249307 | 1.161211477 | 0.215630737 |
|  |  |  |  | 38.0135 | 758.5695532 | 22.87885339 | 19.16456812 | 1.606967359 | 0.027950305 | 0.204346583 | 1.193810017 | 0.255573264 |
|  |  | Dihydrostreptomycin 3'alpha,6-bisphosphate | -5.450725561 | 38.8802 | 744.5529237 | 1.618410604 | 1.36970126 | 1.659845821 | 0.030851399 | 0.208919468 | 1.181579261 | 0.24071641 |
|  |  | Capensinidin | 1.350435765 | 38.8842 | 346.3311429 | 0.732332453 | 0.422012713 | 2.074829214 | 0.001553459 | 0.072455631 | 1.73533268 | 0.795212268 |
|  |  |  |  | 38.886 | 749.5865323 | 0.089782024 | 0.248066333 | 1.798582491 | 0.005604815 | 0.115314823 | 0.361927486 | -1.466227418 |
|  |  |  |  | 39.7324 | 144.9820679 | 9.720621899 | 7.195509378 | 1.772057894 | 0.01213941 | 0.160910988 | 1.350928946 | 0.433951796 |
|  |  | N(omega)-(ADP-D-ribosyl)-L-arginine | 17.01239839 | 39.77135 | 716.520849 | 0.306109825 | 0.428709394 | 1.3714886 | 0.04161253 | 0.223107753 | 0.714026399 | -0.48595068 |
|  |  | {[3-(6,7-dimethoxy-2H-1,3-benzodioxol-5-yl)prop-2-en-1-yl]oxy}sulfonic acid | -11.69811603 | 40.65965 | 319.3035531 | 0.962019963 | 0.508351002 | 1.375270209 | 0.017184524 | 0.178940069 | 1.892432509 | 0.920241849 |
|  |  |  |  | 40.6621 | 362.3262695 | 1.246914523 | 0.804382099 | 1.822616252 | 0.013344131 | 0.166055961 | 1.550152003 | 0.632409689 |
|  |  | Lettowianthine | -5.742924488 | 40.66395 | 318.3003544 | 5.249883066 | 3.277425846 | 1.892076905 | 0.008282202 | 0.138736633 | 1.601831228 | 0.67972215 |
|  |  |  |  | 40.6812 | 274.2734221 | 11.43409852 | 7.219742517 | 1.781297253 | 0.016482224 | 0.176907858 | 1.583726635 | 0.663323335 |
|  |  | Phloretin | 4.190710537 | 40.6817 | 275.277126 | 2.010489758 | 1.267200585 | 1.749016554 | 0.018838345 | 0.183667736 | 1.586559998 | 0.66590208 |
|  |  |  |  | 46.035 | 290.2692601 | 0.520592895 | 0.368879501 | 1.595757463 | 0.046039315 | 0.229790794 | 1.411281715 | 0.497006002 |
|  |  |  |  | 46.95675 | 432.2381752 | 0.801473369 | 0.570644057 | 1.536627892 | 0.035370206 | 0.214837033 | 1.404506642 | 0.490063447 |
|  |  | ({3-[(7-methoxy-2-oxo-2H-chromen-8-yl)methyl]-2-methyloxiran-2-yl}methoxy)sulfonic acid | -5.125453824 | 54.0698 | 357.3554501 | 0.142673004 | 0.092290903 | 1.53604585 | 0.036742454 | 0.216399953 | 1.545905391 | 0.628452029 |
|  |  |  |  | 54.0784 | 356.3515211 | 0.597265059 | 0.389403978 | 1.486812756 | 0.048401473 | 0.232992545 | 1.533792906 | 0.617103702 |
|  |  | Deoxyviolaceinic acid | -4.091295607 | 54.90315 | 358.3679145 | 0.071357776 | 0.042597197 | 1.672765102 | 0.042228434 | 0.224096363 | 1.67517538 | 0.744312144 |
|  |  | (6-carboxy-3,4,5-trihydroxyoxan-2-yl)[4-(2H-chromen-2-ylidene)cyclohexa-2,5-dien-1-ylidene]oxidanium | -7.667844372 | 55.80485 | 400.3782143 | 0.2193135 | 0.14406394 | 1.722417252 | 0.013256147 | 0.165701618 | 1.522334452 | 0.606285349 |
|  |  |  |  | 56.6775 | 795.6098801 | 0.074701027 | 0.035780528 | 1.65532627 | 0.003861941 | 0.101917709 | 2.087756429 | 1.061953408 |
|  |  | Violaceinic acid | -16.41110091 | 57.59115 | 374.3626493 | 0.603089214 | 0.389355878 | 1.677030959 | 0.012133717 | 0.160885081 | 1.54894082 | 0.631282025 |
|  |  | Hippeastrine | -23.87871342 | 57.6696 | 316.3203471 | 0.34245297 | 0.213980887 | 1.654229304 | 0.016022548 | 0.17550861 | 1.600390462 | 0.678423936 |
|  |  |  |  | 58.3836 | 807.5739079 | 1.476502032 | 0.900368219 | 1.653042335 | 0.04722046 | 0.231420729 | 1.639886884 | 0.713596304 |
|  |  |  |  | 58.4253 | 796.6179564 | 0.472738578 | 0.371613144 | 1.588307968 | 0.046219651 | 0.230043526 | 1.272125556 | 0.347241068 |
|  |  |  |  | 58.67 | 834.6000304 | 0.928595587 | 0.39987962 | 1.846990127 | 0.016624148 | 0.177328557 | 2.322187827 | 1.215484667 |
|  |  | 3-hydroxy-2-[4-hydroxy-3-(sulfooxy)phenyl]-5-sulfino-3,4-dihydro-2H-1-benzopyran-7-olate | 4.559671827 | 59.27385 | 418.3891797 | 0.141402653 | 0.095179226 | 1.796962442 | 0.008032222 | 0.136888949 | 1.485646186 | 0.571090572 |
|  |  |  |  | 59.42695 | 74.06078499 | 0.133206124 | 0.11667849 | 1.747873577 | 0.003685874 | 0.100130708 | 1.141651079 | 0.191121789 |
|  |  | Aucubin | -5.985137415 | 59.81825 | 347.3350038 | 0.551008418 | 0.402400139 | 1.569590329 | 0.037401251 | 0.217117074 | 1.369304742 | 0.453443557 |
|  |  |  |  | 60.9779 | 785.5860658 | 8.116210389 | 5.230178849 | 1.502021299 | 0.046415178 | 0.23031595 | 1.551803604 | 0.633945982 |
|  |  |  |  | 61.1004 | 390.3576598 | 0.609636495 | 0.443887741 | 1.636902495 | 0.031255929 | 0.209504067 | 1.373402414 | 0.457754405 |
|  |  |  |  | 61.1258 | 391.361669 | 0.125751907 | 0.090846618 | 1.548531451 | 0.033480368 | 0.21251727 | 1.384222207 | 0.469075555 |
|  |  | 4-Nitrophenol | 15.21441184 | 61.169 | 140.1181931 | 0.228288422 | 0.191674422 | 2.007633556 | 0.003530534 | 0.09846451 | 1.191021835 | 0.252199863 |
|  |  |  |  | 64.6068 | 130.0862274 | 3.098099399 | 2.728784152 | 1.548644547 | 0.036581314 | 0.216221355 | 1.135340586 | 0.183125151 |
|  |  | Letrozole | 1.994107777 | 66.5223 | 286.3105455 | 0.030851507 | 0.01505992 | 1.902927565 | 0.004173017 | 0.10483752 | 2.048583664 | 1.034626814 |
|  |  |  |  | 76.189 | 388.2544986 | 0.031040226 | 0.015003047 | 1.355978629 | 0.030520005 | 0.208431523 | 2.068928158 | 1.04888355 |
|  |  | Crotonoyl-CoA | 2.005236799 | 79.0363 | 836.6170522 | 0.113395493 | 0.066887486 | 1.633857104 | 0.018412344 | 0.18235807 | 1.695317011 | 0.761555071 |
|  |  |  |  | 81.7692 | 376.3420188 | 0.085958965 | 0.065383751 | 1.867470802 | 0.015011251 | 0.172218187 | 1.314683903 | 0.394715966 |
|  |  |  |  | 81.87875 | 432.2795082 | 0.040167483 | 0.014098864 | 1.58940441 | 0.004936874 | 0.110942765 | 2.848987119 | 1.510449099 |
|  |  |  |  | 85.4841 | 362.3262386 | 0.103880265 | 0.065756108 | 2.128050068 | 0.000113704 | 0.035320058 | 1.579781235 | 0.65972479 |
|  |  |  |  | 85.9177 | 101.0713361 | 0.564430288 | 0.511432474 | 1.65547468 | 0.042891795 | 0.225138794 | 1.10362622 | 0.142251638 |
|  |  |  |  | 86.1678 | 806.5706749 | 0.864326778 | 0.325634431 | 1.690092373 | 0.013663192 | 0.167314783 | 2.654285592 | 1.408323608 |
|  |  |  |  | 87.96 | 734.5649952 | 11.8827669 | 5.569537647 | 1.555975964 | 0.041207629 | 0.222446636 | 2.133528428 | 1.093241334 |
|  |  | Prolyl-Gamma-glutamate | -13.36058395 | 91.5369 | 244.2637265 | 0.107587645 | 0.067766076 | 1.652251453 | 0.009104901 | 0.144395226 | 1.587632802 | 0.666877275 |
|  |  | Phosfolan | -10.30017719 | 95.7252 | 256.299347 | 0.319640434 | 0.262596512 | 1.701880543 | 0.039274694 | 0.219161836 | 1.217230313 | 0.283602167 |
|  |  |  |  | 96.8622 | 267.1448329 | 0.002925516 | 0.015152628 | 1.900937602 | 0.015300583 | 0.173190978 | 0.193069883 | -2.372804958 |
|  |  |  |  | 100.448 | 216.2323541 | 0.013882489 | 0.006447721 | 1.793012062 | 0.001159686 | 0.066205678 | 2.153084701 | 1.106405075 |
|  |  |  |  | 104.8165 | 564.3591845 | 0.036742259 | 0.010379018 | 1.237123024 | 0.042037161 | 0.223791513 | 3.540051663 | 1.823770415 |
|  |  | Urothion | 9.910502964 | 113.561 | 326.3775012 | 0.035890945 | 0.018579502 | 1.470110801 | 0.020902902 | 0.189476852 | 1.931749581 | 0.949908085 |
|  |  |  |  | 113.6275 | 392.3361766 | 0.032432984 | 0.016943857 | 1.858467947 | 0.014001247 | 0.168605463 | 1.914144035 | 0.936699394 |
|  |  |  |  | 123.042 | 328.3205643 | 0.028819602 | 0.015181542 | 1.590496514 | 0.049485734 | 0.234387185 | 1.898331669 | 0.924732076 |
|  |  | Tazobactam | -17.43957526 | 123.716 | 301.2931397 | 0.038719102 | 0.018081843 | 1.893514109 | 0.00149403 | 0.07166311 | 2.141324959 | 1.09850375 |
|  |  |  |  | 125.9045 | 180.0769027 | 0.008296358 | 0.00379374 | 1.611712163 | 0.005677802 | 0.115748215 | 2.186854904 | 1.128857502 |
|  |  | Flutriafol | 23.33494185 | 129.9005 | 302.3050072 | 0.726141755 | 0.35353991 | 1.626811296 | 0.012442426 | 0.162267187 | 2.053917352 | 1.03837813 |
|  |  | Luteolin 7-O-[beta-D-glucuronosyl-(1->2)-beta-D-glucuronide]-4'-O-beta-D-glucuronide | 24.76094665 | 130.0135 | 815.6361471 | 0.014410746 | 0.023100867 | 1.47634019 | 0.049213662 | 0.234041456 | 0.623818409 | -0.680801966 |
|  |  | N-Hydroxy-MeIQx | 10.92073428 | 131.1955 | 230.24768 | 0.172541935 | 0.104451001 | 1.492405429 | 0.025631008 | 0.200131268 | 1.651893546 | 0.724120717 |
|  |  |  |  | 139.394 | 759.5709122 | 2.307439502 | 1.769935346 | 1.641101609 | 0.025322339 | 0.199526648 | 1.303685758 | 0.382596162 |
|  |  |  |  | 143.877 | 202.089701 | 0.004642 | 0.002073974 | 1.793197875 | 0.015466089 | 0.173735836 | 2.238214536 | 1.162348327 |
|  |  |  |  | 148.345 | 704.523426 | 0.563948286 | 0.365284771 | 2.064641176 | 0.000936387 | 0.061236336 | 1.543859286 | 0.626541265 |
|  |  |  |  | 150.2655 | 885.6186304 | 0.009435385 | 0.007104074 | 1.701888802 | 0.024236965 | 0.197310029 | 1.32816535 | 0.409434766 |
|  |  |  |  | 150.715 | 222.0549461 | 1.046236143 | 0.841747202 | 1.594796104 | 0.020921105 | 0.189524442 | 1.242933912 | 0.313749589 |
|  |  |  |  | 151.0495 | 887.6355446 | 0.009559142 | 0.004809592 | 2.034941228 | 0.000526425 | 0.056754084 | 1.987516105 | 0.990966551 |
|  |  |  |  | 151.068 | 385.273676 | 0.034838064 | 0.061104867 | 1.616232243 | 0.039041615 | 0.218904366 | 0.570135662 | -0.81062285 |
|  |  |  |  | 151.24 | 881.5863239 | 0.01041325 | 0.006036753 | 1.385854423 | 0.018014656 | 0.181191054 | 1.724975421 | 0.786575805 |
|  |  |  |  | 152.104 | 889.6506737 | 0.014441614 | 0.011576328 | 1.491470124 | 0.040115649 | 0.220617775 | 1.2475125 | 0.319054271 |
|  |  |  |  | 152.11 | 857.5871407 | 0.07889841 | 0.063677294 | 1.976015301 | 0.002883434 | 0.090569871 | 1.239035219 | 0.309217196 |
|  |  |  |  | 152.122 | 856.5838315 | 0.15821187 | 0.121367274 | 2.128783052 | 0.000188595 | 0.0436745 | 1.303579339 | 0.382478392 |
|  |  |  |  | 152.262 | 859.6049687 | 0.105428934 | 0.076374092 | 2.220270363 | 7.88575E-07 | 0.001469743 | 1.38042799 | 0.465115631 |
|  |  |  |  | 152.925 | 858.5993347 | 0.195985829 | 0.148359797 | 2.182529305 | 1.81715E-05 | 0.01913732 | 1.321017103 | 0.401649145 |
|  |  |  |  | 153.791 | 847.6034189 | 0.019686531 | 0.014303252 | 1.577501707 | 0.029288239 | 0.206542804 | 1.37636746 | 0.460865689 |
|  |  |  |  | 153.826 | 861.619217 | 0.133573766 | 0.10471436 | 2.028797189 | 0.000756944 | 0.059798705 | 1.275601224 | 0.351177387 |
|  |  |  |  | 153.954 | 752.5569431 | 0.038629618 | 0.070022689 | 1.599231836 | 0.011930392 | 0.159949278 | 0.55167288 | -0.858115034 |
|  |  |  |  | 154.677 | 820.6199216 | 0.07512881 | 0.189738095 | 1.65517631 | 0.01112753 | 0.156041287 | 0.395960599 | -1.336571216 |
|  |  |  |  | 154.7525 | 823.6036375 | 0.136250093 | 0.10721769 | 1.736235533 | 0.016135616 | 0.175858097 | 1.270779964 | 0.345714249 |
|  |  |  |  | 154.761 | 751.5468365 | 0.01543462 | 0.040154728 | 1.781982095 | 0.004456043 | 0.107260561 | 0.384378652 | -1.379399881 |
|  |  |  |  | 154.7635 | 833.5873736 | 1.025098923 | 0.769562558 | 1.551899789 | 0.028959604 | 0.206017891 | 1.332054051 | 0.413652624 |
|  |  |  |  | 154.797 | 390.2768629 | 0.002111354 | 0.005387373 | 1.226098613 | 0.023096869 | 0.194817553 | 0.391907984 | -1.351413129 |
|  |  |  |  | 154.797 | 748.5270892 | 0.265181053 | 0.34640477 | 1.582142518 | 0.024150327 | 0.197126737 | 0.765523678 | -0.385481093 |
|  |  |  |  | 154.797 | 749.5288469 | 0.122981358 | 0.167777131 | 1.516860189 | 0.026672964 | 0.202093833 | 0.733004296 | -0.448106441 |
|  |  |  |  | 155.627 | 835.6042863 | 1.111126296 | 0.921304605 | 1.535235204 | 0.034923843 | 0.214307348 | 1.206035756 | 0.27027268 |
|  |  |  |  | 156.467 | 725.5322915 | 0.025439247 | 0.053491056 | 1.895737755 | 0.000304673 | 0.050597213 | 0.475579459 | -1.072241691 |
|  |  |  |  | 156.495 | 724.5265074 | 0.086123644 | 0.146852089 | 2.093575719 | 0.000145199 | 0.039441225 | 0.586465228 | -0.769882521 |
|  |  | 3,4,5-trihydroxy-6-{3,4,8,9,10-pentahydroxy-6-oxo-2-[5,6,12,13,14-pentahydroxy-4-(hydroxymethyl)-9-oxo-3,8-dioxatricyclo[8.4.0.0,]tetradeca-1(14),10,12-trien-11-yl]-6H-benzo[c]chromene-1-carbonyloxy}oxane-2-carboxylic acid | 19.95333763 | 156.57 | 809.5864101 | 2.56871335 | 1.901012002 | 1.98940059 | 0.010004748 | 0.149932293 | 1.351234683 | 0.434278264 |
|  |  |  |  | 156.598 | 145.9855615 | 0.657489895 | 0.569522309 | 1.529352371 | 0.026599936 | 0.201960082 | 1.154458543 | 0.207216367 |
|  |  | Malonylapiin | -17.94683979 | 158.242 | 651.5339015 | 0.027222877 | 0.049331654 | 1.36382341 | 0.025673748 | 0.200214125 | 0.551833873 | -0.857694079 |
|  |  |  |  | 158.291 | 783.5740197 | 4.090417544 | 3.301802762 | 1.594545701 | 0.039740564 | 0.219973627 | 1.238843698 | 0.308994178 |
|  |  |  |  | 158.342 | 782.5682648 | 9.014139412 | 7.193281941 | 1.745275478 | 0.015985451 | 0.175393178 | 1.25313306 | 0.325539611 |
|  |  |  |  | 159.171 | 793.5553071 | 0.151503638 | 0.300740739 | 1.719104181 | 0.007838144 | 0.135409533 | 0.503768256 | -0.989167878 |
|  |  | Quercetin 3-O-beta-D-glucosyl-(1->2)-beta-D-glucoside | 13.87381982 | 159.214 | 627.5328688 | 0.151759069 | 0.247592662 | 1.387898919 | 0.041320879 | 0.222632457 | 0.612938476 | -0.706185824 |
|  |  |  |  | 159.243 | 792.5527885 | 0.509320979 | 0.733664042 | 1.550531557 | 0.020484758 | 0.188367185 | 0.69421554 | -0.526544435 |
|  |  | 3,4,5-trihydroxy-6-(3-{5,6,7-trihydroxy-4-oxo-8-[3,4,5-trihydroxy-6-(hydroxymethyl)oxan-2-yl]-4H-chromen-2-yl}phenoxy)oxane-2-carboxylic acid | 13.68476645 | 159.2585 | 625.5198228 | 0.186443956 | 0.234915369 | 1.342143805 | 0.049421557 | 0.234305884 | 0.793664359 | -0.333399074 |
|  |  | Theogallinin | -18.33247528 | 160.0145 | 801.6192991 | 0.176083886 | 0.241193092 | 1.691484669 | 0.012633768 | 0.163101258 | 0.730053603 | -0.4539257 |
|  |  |  |  | 160.957 | 789.6194 | 1.564784193 | 2.477109157 | 1.457133307 | 0.037545261 | 0.217271098 | 0.631697714 | -0.662693743 |
|  |  |  |  | 161.077 | 773.588208 | 1.503895827 | 1.310771956 | 1.45716933 | 0.046931115 | 0.231026924 | 1.14733598 | 0.198287925 |
|  |  |  |  | 161.8665 | 600.5068031 | 0.020963651 | 0.045921533 | 1.691714228 | 0.039041487 | 0.218904224 | 0.456510259 | -1.131280814 |
|  |  |  |  | 162.727 | 758.569464 | 38.77027734 | 33.26290967 | 1.59050152 | 0.031841965 | 0.210330162 | 1.165570833 | 0.221036681 |
|  |  |  |  | 162.727 | 759.570625 | 18.71670873 | 15.97383028 | 1.631472735 | 0.02636547 | 0.201526864 | 1.171710754 | 0.228616473 |
|  |  | Amaroswerin | -24.02968006 | 165.346 | 603.5328978 | 0.054605137 | 0.126843384 | 1.827010405 | 0.00065642 | 0.058697481 | 0.430492587 | -1.2159397 |
|  |  | Luteoskyrin | 11.98192884 | 167.122 | 575.5026601 | 0.213277784 | 0.32769208 | 1.566207318 | 0.017601105 | 0.180089179 | 0.650848149 | -0.61960711 |
|  |  |  |  | 167.1265 | 576.5074936 | 0.07620577 | 0.118002431 | 1.581965338 | 0.023335683 | 0.195354437 | 0.645798312 | -0.630844426 |
|  |  | [2,6-dihydroxy-4-({[3-hydroxy-6-(hydroxymethyl)-2,5-bis(3,4,5-trihydroxybenzoyloxy)oxan-4-yl]oxy}carbonyl)phenyl]oxidanesulfonic acid | -15.64110509 | 167.154 | 717.5260693 | 0.515283852 | 0.83462332 | 1.50383495 | 0.021303938 | 0.190511848 | 0.617384921 | -0.695757848 |
|  |  | Pradimicinone I | 4.733676182 | 168.9115 | 550.4921777 | 0.052896582 | 0.079362808 | 1.396619054 | 0.033888651 | 0.213035959 | 0.666516009 | -0.585288567 |
|  |  |  |  | 178.6475 | 814.6865566 | 0.058510061 | 0.126542824 | 1.652992603 | 0.013610937 | 0.167111373 | 0.462373599 | -1.112869072 |
|  |  |  |  | 180.845 | 444.0559977 | 0.134088948 | 0.077331483 | 1.673002402 | 0.03454149 | 0.21384488 | 1.7339503 | 0.794062547 |
|  |  |  |  | 182.5965 | 168.0326408 | 0.003270945 | 0.012990446 | 1.629178987 | 0.014411359 | 0.17011481 | 0.25179616 | -1.989671813 |
|  |  |  |  | 183.153 | 152.0107222 | 0.017276523 | 0.008678396 | 1.721109777 | 0.016236903 | 0.176168194 | 1.990750793 | 0.993312632 |
|  |  |  |  | 183.226 | 712.067534 | 0.071125146 | 0.059217072 | 1.749112825 | 0.010013646 | 0.149983999 | 1.201091916 | 0.264346561 |
|  |  |  |  | 184.142 | 289.0411996 | 0.018973359 | 0.013140442 | 1.687712702 | 0.024371361 | 0.197592447 | 1.443890426 | 0.529961263 |
|  |  |  |  | 184.968 | 234.0409506 | 0.00752269 | 0.003815278 | 1.748044958 | 0.02093621 | 0.189563888 | 1.971727583 | 0.979460241 |
|  |  |  |  | 185.806 | 239.1063673 | 0.002437088 | 0.005403832 | 1.586566116 | 0.014857945 | 0.171691978 | 0.450992491 | -1.148824682 |
|  |  |  |  | 185.9015 | 202.0035649 | 0.008949972 | 0.006452546 | 1.509794971 | 0.036746755 | 0.216404703 | 1.387045132 | 0.472014731 |
|  |  |  |  | 186.796 | 115.957737 | 0.021299009 | 0.014770534 | 1.735488256 | 0.015001664 | 0.172185501 | 1.441993111 | 0.528064272 |
|  |  |  |  | 188.474 | 151.0616382 | 0.049878854 | 0.028936809 | 1.823648699 | 0.003236429 | 0.095053973 | 1.723716442 | 0.785522465 |
|  |  |  |  | 189.4165 | 112.8960871 | 0.252248347 | 0.205566009 | 1.70413248 | 0.021582207 | 0.191213778 | 1.227091716 | 0.295243084 |
|  |  |  |  | 191.22 | 108.0560067 | 0.611548296 | 0.870584244 | 1.433862397 | 0.032689725 | 0.211483572 | 0.702457344 | -0.509517473 |
|  |  |  |  | 191.269 | 164.0819053 | 0.032356046 | 0.053292932 | 1.616208118 | 0.004907291 | 0.110730397 | 0.60713579 | -0.719908873 |
|  |  |  |  | 192.01 | 454.3880947 | 0.003452656 | 0.009881087 | 1.834796459 | 0.000791834 | 0.060123255 | 0.349420661 | -1.51696318 |
|  |  |  |  | 194.2595 | 523.3586052 | 0.301713085 | 0.181100554 | 1.647851354 | 0.028185074 | 0.204743553 | 1.665997576 | 0.736386302 |
|  |  |  |  | 194.661 | 522.3545103 | 0.995181236 | 0.618077682 | 1.575924698 | 0.03481194 | 0.214172843 | 1.610123233 | 0.687171111 |
|  |  |  |  | 194.845 | 180.0444249 | 0.089225978 | 0.133412217 | 1.727613977 | 0.014851217 | 0.171668712 | 0.668799152 | -0.580355077 |
|  |  |  |  | 195.522 | 334.29453 | 0.046877999 | 0.023999796 | 1.543681442 | 0.035208729 | 0.214646662 | 1.953266525 | 0.96588882 |
|  |  |  |  | 195.552 | 447.3295557 | 0.000273346 | 0.001911632 | 1.657335552 | 0.018711101 | 0.183280846 | 0.142990965 | -2.806004106 |
|  |  |  |  | 195.72 | 503.3414479 | 0.024176463 | 0.01717798 | 1.585848417 | 0.029520654 | 0.206908548 | 1.407410156 | 0.493042828 |
|  |  |  |  | 196.6945 | 501.3255083 | 0.040613151 | 0.029124284 | 1.742999197 | 0.008312934 | 0.138959434 | 1.39447723 | 0.479724377 |
|  |  | 3-Iodo-4-hydroxyphenylpyruvate | -5.336523006 | 197.423 | 307.0596433 | 0.044094949 | 0.035141063 | 1.5212844 | 0.038144175 | 0.217901455 | 1.254798388 | 0.32745558 |
|  |  | 6-({3-[(3-{[3,4-dihydroxy-4-(hydroxymethyl)oxolan-2-yl]oxy}-4,5-dihydroxy-6-(hydroxymethyl)oxan-2-yl)oxy]-5-hydroxy-2-(4-hydroxyphenyl)-4-oxo-4H-chromen-7-yl}oxy)-3,4,5-trihydroxyoxane-2-carboxylic acid | -6.037871551 | 199.1685 | 757.6217082 | 0.118711014 | 0.147905504 | 1.667064502 | 0.01634227 | 0.176487845 | 0.802613907 | -0.317221941 |
|  |  | Delphinidin 3-O-(6-caffeoyl-beta-D-glucoside) | 5.91472037 | 199.2175 | 628.5373882 | 0.460656136 | 0.565347111 | 1.594400141 | 0.0231622 | 0.194965232 | 0.814820005 | -0.295446693 |
|  |  | (S)-2,3-Dihydro-3,5-dihydroxy-2-oxo-3-indoleacetic acid 5-glucoside | -8.616258329 | 199.335 | 386.3267566 | 0.016048332 | 0.032830063 | 1.885616961 | 0.016955735 | 0.178291489 | 0.48883037 | -1.032594175 |
|  |  | [2,6-dihydroxy-4-(3,5,7-trihydroxy-3,4-dihydro-2H-1-benzopyran-2-yl)phenyl]oxidanesulfonic acid | -18.54314334 | 200.022 | 387.3301128 | 0.000739604 | 0.005771527 | 1.850086169 | 0.002519241 | 0.085990874 | 0.128146986 | -2.964128544 |
|  |  |  |  | 202.831 | 390.7700031 | 0.019376893 | 0.012865516 | 1.819345076 | 0.019061289 | 0.184336989 | 1.50611089 | 0.590827995 |
|  |  |  |  | 202.944 | 723.5461635 | 0.042262259 | 0.031907292 | 1.548176828 | 0.027731411 | 0.20397182 | 1.324532944 | 0.405483727 |
|  |  |  |  | 202.953 | 701.5583543 | 3.836428943 | 2.929179731 | 1.696285809 | 0.019927726 | 0.186838189 | 1.309728079 | 0.389267316 |
|  |  |  |  | 205.461 | 863.6287226 | 0.015550619 | 0.019179432 | 1.469210471 | 0.048733984 | 0.233425055 | 0.810796663 | -0.302587945 |
|  |  |  |  | 208.2955 | 233.9148358 | 0.016160911 | 0.021171977 | 1.480768743 | 0.02944447 | 0.206789154 | 0.763316085 | -0.389647503 |
|  |  |  |  | 208.392 | 583.3234749 | 0.007988211 | 0.014921372 | 1.486694227 | 0.048185016 | 0.232708657 | 0.53535366 | -0.901435828 |
|  |  |  |  | 210.199 | 825.5470153 | 0.001514905 | 0.004448623 | 1.447495906 | 0.014955253 | 0.172026856 | 0.340533353 | -1.554131987 |
|  |  |  |  | 210.735 | 824.5426423 | 0.011383995 | 0.017873689 | 1.456226899 | 0.024692219 | 0.198257439 | 0.636913579 | -0.650830465 |
|  |  |  |  | 211.014 | 362.2767691 | 0.016057126 | 0.01165166 | 1.578207966 | 0.033138302 | 0.212074867 | 1.378097704 | 0.462678175 |
|  |  |  |  | 211.091 | 880.5892932 | 0.02322173 | 0.042735246 | 1.383336832 | 0.027244054 | 0.203120934 | 0.543385891 | -0.879950987 |
|  |  |  |  | 211.582 | 881.5929444 | 0.006039724 | 0.010548331 | 1.717936418 | 0.007535011 | 0.133016035 | 0.57257626 | -0.804460239 |
|  |  |  |  | 211.624 | 569.3434955 | 0.022792904 | 0.012146743 | 1.596003143 | 0.016826251 | 0.177918745 | 1.876462103 | 0.908015154 |
|  |  | Kolaflavanone | -7.668627211 | 211.733 | 589.5178635 | 0.026328022 | 0.045799198 | 1.634749272 | 0.013318816 | 0.165954332 | 0.574857707 | -0.798723201 |
|  |  |  |  | 211.962 | 835.5328751 | 0.11740706 | 0.086384368 | 1.8049154 | 0.004960641 | 0.111112127 | 1.359123912 | 0.442676994 |
|  |  |  |  | 212.327 | 606.5531973 | 0.00473616 | 0.010042816 | 1.075405467 | 0.031791997 | 0.210260663 | 0.471596786 | -1.084374208 |
|  |  |  |  | 212.728 | 129.1469947 | 0.389494082 | 0.311571205 | 2.052131721 | 0.000835631 | 0.060496597 | 1.250096531 | 0.322039503 |
|  |  |  |  | 212.823 | 128.143574 | 4.625205941 | 3.796722582 | 2.018217345 | 0.001738537 | 0.074665501 | 1.218210138 | 0.284763016 |
|  |  |  |  | 213.468 | 439.3019753 | 0.010333834 | 0.006622299 | 1.604899268 | 0.033101009 | 0.212026196 | 1.560460074 | 0.641971444 |
|  |  |  |  | 214.5185 | 788.5437167 | 0.461699426 | 0.596126125 | 1.586980189 | 0.025394721 | 0.199669419 | 0.774499569 | -0.36866366 |
|  |  |  |  | 214.843 | 790.5569539 | 0.170795368 | 0.227523046 | 1.801363459 | 0.006436387 | 0.123389915 | 0.750672828 | -0.413743832 |
|  |  | 3,4,5-trihydroxy-6-({6,13,14-trihydroxy-3,10-dioxo-12-[5,6,7-trihydroxy-1-oxo-3-(1,2,3,4-tetrahydroxybutyl)-1H-isochromen-8-yl]-2,9-dioxatetracyclo[6.6.2.0,.0,]hexadeca-1(15),4,6,8(16),11,13-hexaen-7-yl}oxy)oxane-2-carboxylic acid | 6.597094599 | 215.3265 | 791.5604919 | 0.067278647 | 0.090109367 | 2.030738594 | 0.000856179 | 0.060660039 | 0.74663322 | -0.421528396 |
|  |  |  |  | 215.3525 | 777.5453773 | 0.008830325 | 0.015914667 | 1.435731609 | 0.029965443 | 0.207596168 | 0.554854505 | -0.849818579 |
|  |  |  |  | 216.727 | 764.5345148 | 0.041935779 | 0.086137013 | 1.44126454 | 0.008684078 | 0.141578181 | 0.486849704 | -1.038451631 |
|  |  |  |  | 217.425 | 442.3528475 | 0.001653339 | 0.005055173 | 1.847452134 | 0.005309788 | 0.113478292 | 0.327058913 | -1.612377564 |
|  |  |  |  | 217.794 | 519.3261934 | 0.028705027 | 0.02027559 | 1.458246268 | 0.045796631 | 0.22944843 | 1.415743091 | 0.50155949 |
|  |  |  |  | 219.2965 | 247.1439026 | 0.053825188 | 0.039417558 | 1.587659804 | 0.015298199 | 0.173183069 | 1.365512993 | 0.449443042 |
|  |  | 4,5-Dichloro-3H-1,3-dithiol-2-one | -24.16551414 | 219.4955 | 188.070756 | 0.0008087 | 0.002705052 | 1.256691193 | 0.030603761 | 0.208555628 | 0.298959263 | -1.741979181 |
|  |  |  |  | 223.813 | 123.0554962 | 0.71450745 | 1.153668624 | 1.673324191 | 0.02256548 | 0.193593284 | 0.619335081 | -0.691207929 |
|  |  |  |  | 224.4205 | 163.0503332 | 0.080168511 | 0.09707006 | 1.537310288 | 0.037480642 | 0.217202105 | 0.82588299 | -0.275990697 |
|  |  |  |  | 224.521 | 281.0992434 | 0.096421933 | 0.071679292 | 1.596824816 | 0.020215705 | 0.187636073 | 1.345185351 | 0.427804973 |
|  |  | L-2-Aminoadipate adenylate | -15.74551852 | 225.606 | 491.3613556 | 0.02594787 | 0.017213846 | 1.752246617 | 0.016136659 | 0.175861303 | 1.507383622 | 0.592046622 |
|  |  |  |  | 229.1065 | 224.1282514 | 0.06741359 | 0.059136495 | 1.54310187 | 0.038729854 | 0.218556101 | 1.139965932 | 0.18899071 |
|  |  |  |  | 230.419 | 182.0320312 | 0.373125104 | 0.277383515 | 1.770131375 | 0.025376986 | 0.199634494 | 1.34515962 | 0.427777377 |
|  |  | N-phosphocreatinate(2-) | 19.04096932 | 231.748 | 210.110258 | 0.014179004 | 0.025056001 | 1.306839707 | 0.023655646 | 0.196061249 | 0.565892558 | -0.821399931 |
|  |  |  |  | 235.523 | 274.2013192 | 0.038470119 | 0.089931156 | 1.707360292 | 0.005684358 | 0.115786754 | 0.427772988 | -1.22508271 |
|  |  |  |  | 241.069 | 193.0446983 | 0.043761126 | 0.029166265 | 1.688109894 | 0.01505717 | 0.172374329 | 1.500402121 | 0.585349208 |
|  |  |  |  | 241.323 | 192.052075 | 0.06502775 | 0.04492397 | 1.703081861 | 0.011011019 | 0.155444112 | 1.447506744 | 0.53357007 |
|  |  |  |  | 241.836 | 191.0487147 | 1.056227497 | 0.745142456 | 1.636862294 | 0.017192482 | 0.178962403 | 1.417483983 | 0.503332434 |
|  |  |  |  | 244.501 | 332.2433455 | 0.002692236 | 0.003894287 | 1.54681833 | 0.025594162 | 0.200059671 | 0.691329748 | -0.532554088 |
|  |  |  |  | 245.305 | 220.0392158 | 0.028311335 | 0.041423153 | 1.58584332 | 0.017952241 | 0.181027156 | 0.683466451 | -0.549057571 |
|  |  |  |  | 245.615 | 261.1890468 | 0.014626852 | 0.026160378 | 2.119461886 | 4.83751E-05 | 0.029017061 | 0.559122363 | -0.838764046 |
|  |  | Nitrofen | 2.023121615 | 248.6295 | 285.1027514 | 0.105135999 | 0.058640327 | 1.516522919 | 0.016251552 | 0.176212815 | 1.792895846 | 0.842291681 |
|  |  |  |  | 248.814 | 284.0985547 | 1.042050458 | 0.648646166 | 1.513964412 | 0.016207921 | 0.176079749 | 1.606500606 | 0.683921525 |
|  |  | Methyl viologen | 13.69517395 | 252.167 | 258.1697984 | 0.007987343 | 0.013222802 | 1.881697348 | 0.002840339 | 0.090066516 | 0.604058291 | -0.72724032 |
|  |  |  |  | 253.0625 | 377.3043314 | 0.009562473 | 0.00560741 | 1.759375714 | 0.012485866 | 0.162458032 | 1.705328135 | 0.770049366 |
|  |  |  |  | 253.964 | 640.3457893 | 0.009260795 | 0.005412443 | 1.663815685 | 0.036598657 | 0.216240639 | 1.711019289 | 0.774856024 |
|  |  |  |  | 254.4795 | 335.0735715 | 0.010575392 | 0.00647335 | 1.57733736 | 0.040435125 | 0.221159905 | 1.63368155 | 0.70812679 |
|  |  |  |  | 255.813 | 302.1457045 | 0.005946801 | 0.003150427 | 1.568733747 | 0.026991323 | 0.202670478 | 1.887617342 | 0.916566331 |
|  |  |  |  | 255.833 | 245.095854 | 0.02255466 | 0.012275071 | 1.533132536 | 0.042853604 | 0.225079395 | 1.837436192 | 0.877694151 |
|  |  |  |  | 255.86 | 355.1355478 | 0.099250172 | 0.046585485 | 1.756132844 | 0.017857426 | 0.180776563 | 2.130495623 | 1.091189088 |
|  |  |  |  | 257.033 | 112.0508083 | 6.341836564 | 4.871955897 | 1.631326883 | 0.048587854 | 0.233235508 | 1.301702375 | 0.380399625 |
|  |  | Pyridoxamine phosphate | -9.49307772 | 261.87 | 249.1779207 | 0.003394724 | 0.006285325 | 1.367860501 | 0.03088112 | 0.208962829 | 0.540103166 | -0.888693089 |
|  |  |  |  | 261.899 | 122.0813894 | 0.800198976 | 0.663892204 | 1.717387996 | 0.025853861 | 0.200561036 | 1.205314613 | 0.269409771 |
|  |  | Nitrofen | 2.195547438 | 262.346 | 285.1028003 | 0.098101229 | 0.056514497 | 1.640634509 | 0.017603517 | 0.180095716 | 1.735859553 | 0.795650225 |
|  |  | Lorazepam glucuronide | -14.9128896 | 263.275 | 498.2818607 | 0.025005154 | 0.019026236 | 1.832351668 | 0.008280234 | 0.138722336 | 1.314245972 | 0.394235314 |
|  |  |  |  | 264.1805 | 602.3371002 | 0.018041517 | 0.013649427 | 1.597468611 | 0.038028223 | 0.217780683 | 1.321778329 | 0.402480248 |
|  |  |  |  | 264.202 | 583.3235749 | 0.152619738 | 0.11671008 | 1.593040028 | 0.046349755 | 0.230224982 | 1.307682581 | 0.387012392 |
|  |  |  |  | 264.2215 | 584.326976 | 0.04583624 | 0.033344184 | 1.632981525 | 0.036569259 | 0.216207943 | 1.374639755 | 0.459053588 |
|  |  | O-Phospho-L-serine | -19.06925709 | 269.8905 | 186.0762474 | 0.071279902 | 0.053678813 | 1.996563175 | 0.004600371 | 0.108419697 | 1.327896379 | 0.409142572 |
|  |  |  |  | 270.484 | 168.0656651 | 0.020863359 | 0.01631605 | 1.693258578 | 0.012857625 | 0.164055967 | 1.278701643 | 0.354679682 |
|  |  |  |  | 271.6265 | 364.0167805 | 0.003741711 | 0.002391016 | 2.03077989 | 0.000827472 | 0.060429697 | 1.564904544 | 0.646074658 |
|  |  |  |  | 271.632 | 145.9857065 | 0.045699158 | 0.034216614 | 2.062296514 | 0.002934447 | 0.091153569 | 1.335583874 | 0.41747058 |
|  |  |  |  | 271.6565 | 320.0651778 | 0.022265148 | 0.013654804 | 1.806078098 | 0.00358964 | 0.099108836 | 1.630572547 | 0.705378631 |
|  |  |  |  | 271.6845 | 297.9907013 | 0.065265046 | 0.046102734 | 1.71238015 | 0.011303082 | 0.156926174 | 1.415643721 | 0.501458224 |
|  |  |  |  | 272.719 | 279.0384777 | 0.029665373 | 0.023416595 | 1.544446576 | 0.037181149 | 0.216879788 | 1.266852512 | 0.341248574 |
|  |  | Polixetonium chloride | 14.66902885 | 275.435 | 326.2080469 | 0.014679055 | 0.008043045 | 1.979140374 | 0.002833345 | 0.089983916 | 1.825061825 | 0.867945337 |
|  |  | sn-Glycerol 3-phosphate | 0.440553077 | 277.018 | 173.0810524 | 0.041729365 | 0.060442448 | 1.847825653 | 0.004820311 | 0.110095768 | 0.690398322 | -0.534499136 |
|  |  | CNQX | -8.977219358 | 277.19 | 233.1576925 | 0.487034436 | 0.746452161 | 1.928548106 | 0.001830257 | 0.075635822 | 0.652465706 | -0.616026021 |
|  |  |  |  | 277.933 | 90.09188867 | 0.141249249 | 0.120594622 | 1.652795059 | 0.021146687 | 0.190109354 | 1.171273206 | 0.228077632 |
|  |  |  |  | 279.061 | 114.0554169 | 0.075732229 | 0.12027947 | 1.853759213 | 0.003130474 | 0.09373482 | 0.629635538 | -0.667411124 |
|  |  | N-Nitrosomethylvinylamine | 6.688308169 | 281.809 | 87.10035241 | 0.252608015 | 0.352951768 | 1.485952779 | 0.038359375 | 0.218136359 | 0.715701232 | -0.482570633 |
|  |  |  |  | 291.981 | 178.0865198 | 0.022251571 | 0.049775551 | 2.017783814 | 0.012106523 | 0.160761115 | 0.447038178 | -1.161530048 |
|  |  |  |  | 292.9265 | 172.0966993 | 0.04005294 | 0.090315815 | 1.826931999 | 0.025436535 | 0.199751618 | 0.443476486 | -1.173070482 |
|  |  |  |  | 293.771 | 284.0985603 | 0.11282479 | 0.169462215 | 1.592956096 | 0.029528464 | 0.20692076 | 0.665781393 | -0.586879544 |
|  |  |  |  | 297.5405 | 339.0176444 | 0.208333107 | 0.139504718 | 1.683460345 | 0.026015075 | 0.20086847 | 1.493376778 | 0.578578202 |
|  |  |  |  | 297.8215 | 297.0073389 | 0.046957682 | 0.029513561 | 1.607043585 | 0.047210088 | 0.231406673 | 1.591054403 | 0.669983167 |
|  |  |  |  | 301.2885 | 698.3789562 | 0.005674593 | 0.002648909 | 1.981788372 | 0.001618345 | 0.073272394 | 2.14223799 | 1.099118764 |
|  |  | Glucosyl passiflorate | 5.211277922 | 301.395 | 697.8760082 | 0.007654504 | 0.004890716 | 1.644640485 | 0.023213946 | 0.195081769 | 1.565109249 | 0.646263365 |
|  |  |  |  | 304.274 | 320.0279393 | 0.008110658 | 0.003357208 | 1.904611299 | 0.001054391 | 0.064029731 | 2.41589409 | 1.27255721 |
|  |  |  |  | 306.133 | 116.0709883 | 0.58779517 | 0.452735964 | 1.819468517 | 0.013348789 | 0.166074629 | 1.298317819 | 0.376643588 |
|  |  |  |  | 307.7385 | 103.0618669 | 0.217265209 | 0.181905888 | 1.828673793 | 0.020788586 | 0.189176632 | 1.194382499 | 0.256264931 |
|  |  |  |  | 307.9625 | 176.0375885 | 0.086337459 | 0.119304174 | 1.436981639 | 0.042165554 | 0.223996359 | 0.723675091 | -0.46658598 |
|  |  |  |  | 310.695 | 539.0121035 | 0.004380602 | 0.003138875 | 1.412827868 | 0.037030349 | 0.216715889 | 1.395596265 | 0.480881641 |
|  |  |  |  | 316.341 | 300.1552543 | 0.006165902 | 0.004004035 | 1.799967958 | 0.020920558 | 0.189523014 | 1.539922136 | 0.622857405 |
|  |  | Neburon | -3.639956617 | 318.801 | 276.180475 | 0.005963428 | 0.012397113 | 1.712436966 | 0.006345729 | 0.122521126 | 0.481033582 | -1.055790479 |
|  |  | Oxaloacetate | -18.69749132 | 319.686 | 133.0764072 | 0.013082253 | 0.005067521 | 1.312071595 | 0.013992193 | 0.168571454 | 2.581588369 | 1.368258983 |
|  |  |  |  | 320.042 | 100.0398401 | 0.052877872 | 0.03348341 | 1.925072643 | 0.002745312 | 0.088921946 | 1.579225994 | 0.659217642 |
|  |  |  |  | 321.507 | 241.1548872 | 0.075552672 | 0.098325978 | 1.737368645 | 0.002897565 | 0.090732861 | 0.768389731 | -0.380089856 |
|  |  |  |  | 322.427 | 334.0549957 | 0.018204985 | 0.013024214 | 1.347330087 | 0.047164482 | 0.231344814 | 1.397780016 | 0.483137326 |
|  |  |  |  | 322.5825 | 206.1268907 | 0.093218972 | 0.131114633 | 1.713573649 | 0.017291249 | 0.179238321 | 0.71097306 | -0.492133201 |
|  |  |  |  | 322.681 | 205.1263954 | 0.94412192 | 1.929513275 | 1.055847559 | 0.012373746 | 0.161963645 | 0.489305739 | -1.03119189 |
|  |  |  |  | 325.935 | 155.5573465 | 0.086536643 | 0.130819134 | 1.426740392 | 0.033755627 | 0.212868065 | 0.661498362 | -0.59619051 |
|  |  |  |  | 325.946 | 192.5760423 | 0.684040613 | 1.057190756 | 1.35602881 | 0.047622593 | 0.231962278 | 0.647036128 | -0.628081826 |
|  |  | Alendronic acid | 15.94853543 | 325.9635 | 250.1072493 | 0.022599352 | 0.04653083 | 1.589691997 | 0.020232462 | 0.187682008 | 0.485685563 | -1.041905492 |
|  |  |  |  | 325.981 | 369.1690076 | 0.069434636 | 0.097790849 | 1.493772803 | 0.026175433 | 0.201171436 | 0.710032037 | -0.494043974 |
|  |  |  |  | 326.035 | 294.1293892 | 0.082375037 | 0.115649929 | 1.48698673 | 0.027080297 | 0.202829791 | 0.712279183 | -0.489485269 |
|  |  |  |  | 326.0695 | 368.1664594 | 0.280904586 | 0.394807489 | 1.453128815 | 0.026906343 | 0.202517569 | 0.711497615 | -0.491069175 |
|  |  |  |  | 329.264 | 205.0237389 | 0.047238685 | 0.035139813 | 1.410160649 | 0.040795908 | 0.221765052 | 1.344306693 | 0.426862316 |
|  |  |  |  | 329.479 | 207.0602942 | 0.013317628 | 0.005094602 | 1.928087301 | 0.000584808 | 0.05771741 | 2.614066443 | 1.386295811 |
|  |  |  |  | 330.099 | 186.1124991 | 0.016421791 | 0.024801589 | 1.130333804 | 0.043347107 | 0.22584125 | 0.662126552 | -0.594821111 |
|  |  | 4-Nitrophenol | 14.99533603 | 331.4755 | 140.1181626 | 0.030226951 | 0.046577637 | 1.323601734 | 0.046637684 | 0.23062396 | 0.648958451 | -0.623801982 |
|  |  | N-Methyl-N'-nitro-N-nitrosoguanidine | -22.42524117 | 332.816 | 148.096778 | 0.023921044 | 0.034583738 | 1.432262064 | 0.022982966 | 0.194558615 | 0.691684733 | -0.531813482 |
|  |  |  |  | 333.622 | 159.0513535 | 0.014915583 | 0.008896994 | 1.987652526 | 0.002388873 | 0.08415261 | 1.676474496 | 0.745430536 |
|  |  | Aminoethoxyacetic acid | -2.343810291 | 335.043 | 119.1181998 | 0.825560183 | 0.328219298 | 1.502883153 | 0.032055843 | 0.210625702 | 2.515270094 | 1.330713328 |
|  |  |  |  | 335.533 | 254.1611089 | 0.00503933 | 0.00318976 | 1.577914925 | 0.018972988 | 0.184073217 | 1.579846096 | 0.659784022 |
|  |  |  |  | 337.719 | 276.0646578 | 0.202145766 | 0.174396959 | 1.980335381 | 0.002105391 | 0.07971847 | 1.159112907 | 0.213021103 |
|  |  |  |  | 341.5415 | 356.8555069 | 0.011258925 | 0.01719324 | 1.809224714 | 0.002522983 | 0.086041954 | 0.654846034 | -0.610772352 |
|  |  |  |  | 343.837 | 276.8320278 | 0.029643555 | 0.020027469 | 1.338893245 | 0.046006588 | 0.229744776 | 1.480144849 | 0.565738367 |
|  |  |  |  | 344.34 | 162.8995706 | 0.05442845 | 0.069763844 | 1.491247478 | 0.038405503 | 0.218188974 | 0.780181356 | -0.358118573 |
|  |  |  |  | 349.5975 | 175.0536213 | 0.135988569 | 0.210342064 | 1.351801452 | 0.03802622 | 0.217778591 | 0.646511527 | -0.629252003 |
|  |  | N-Formylmaleamic acid | -18.12145357 | 350.024 | 144.1021835 | 0.042403989 | 0.069058425 | 1.262447429 | 0.045741374 | 0.229370113 | 0.614030645 | -0.703617437 |
|  |  |  |  | 352.953 | 309.0587912 | 0.004747988 | 0.004078562 | 1.615434154 | 0.012448185 | 0.162292537 | 1.164132765 | 0.219255601 |
|  |  |  |  | 352.96 | 405.9480674 | 0.003341854 | 0.002014572 | 2.045178676 | 9.26314E-05 | 0.034073311 | 1.658840251 | 0.730174959 |
|  |  |  |  | 357.7265 | 146.0562631 | 0.060236017 | 0.052758186 | 1.8645055 | 0.005604435 | 0.115312546 | 1.141737842 | 0.191231427 |
|  |  |  |  | 358.2115 | 113.0462328 | 0.220535019 | 0.176485212 | 1.546985977 | 0.047638794 | 0.231983956 | 1.249594893 | 0.321460462 |
|  |  |  |  | 360.011 | 176.0375901 | 0.230500512 | 0.333181764 | 1.559975986 | 0.016276365 | 0.176288258 | 0.69181611 | -0.531539485 |
|  |  |  |  | 361.307 | 262.1043862 | 0.007630566 | 0.005640238 | 1.626039306 | 0.017405658 | 0.179555072 | 1.352880137 | 0.436034024 |
|  |  |  |  | 361.367 | 332.1020172 | 0.013088522 | 0.010246934 | 1.555470146 | 0.025635342 | 0.20013968 | 1.277311074 | 0.353109919 |
|  |  |  |  | 362.232 | 229.0499651 | 0.004135072 | 0.005968652 | 1.497921887 | 0.047853378 | 0.232270088 | 0.692798181 | -0.529492953 |
|  |  | Thioxanthine monophosphate | -4.169955486 | 365.07 | 263.1461835 | 0.227859022 | 0.389564799 | 1.75538669 | 0.034978963 | 0.214373345 | 0.584906599 | -0.773721828 |
|  |  | 2,4-Dichlorobenzoate | -21.10083866 | 365.1165 | 192.0147461 | 0.00769707 | 0.012905813 | 1.866584802 | 0.003237221 | 0.095063634 | 0.596403368 | -0.74563969 |
|  |  |  |  | 365.12 | 154.0589067 | 0.314882406 | 0.377231082 | 1.467131291 | 0.04887566 | 0.233608032 | 0.834720203 | -0.260635406 |
|  |  |  |  | 365.1215 | 264.1499262 | 0.017018089 | 0.030779746 | 1.70921928 | 0.022609722 | 0.193696812 | 0.552898937 | -0.854912297 |
|  |  |  |  | 365.1215 | 285.1283821 | 0.012452287 | 0.021252992 | 1.809735849 | 0.003822384 | 0.10152517 | 0.585907454 | -0.771255292 |
|  |  |  |  | 368.758 | 139.0179629 | 0.07160239 | 0.059276978 | 1.452645201 | 0.043267438 | 0.22571909 | 1.207929147 | 0.272535833 |
|  |  |  |  | 370.592 | 170.1172547 | 0.02862771 | 0.035033883 | 1.666448611 | 0.011347624 | 0.157147899 | 0.817143501 | -0.291338638 |
|  |  |  |  | 373.437 | 146.0923115 | 0.135527567 | 0.381360474 | 1.892786698 | 0.000182351 | 0.043143922 | 0.355379165 | -1.492568992 |
|  |  |  |  | 374.195 | 99.05578416 | 0.147985818 | 0.231901911 | 1.759354936 | 0.004839033 | 0.110233675 | 0.63813971 | -0.648055783 |
|  |  |  |  | 377.7925 | 884.0221636 | 0.134953867 | 0.065792902 | 1.594906461 | 0.033766525 | 0.212881859 | 2.051191909 | 1.036462477 |
|  |  |  |  | 377.8235 | 937.0046909 | 0.169065059 | 0.0968822 | 1.714991998 | 0.036252299 | 0.2158527 | 1.745057998 | 0.803274986 |
|  |  |  |  | 377.838 | 720.0138982 | 0.108286368 | 0.044656923 | 1.840179339 | 0.001908131 | 0.076721538 | 2.424850617 | 1.277895873 |
|  |  |  |  | 377.851 | 907.9819925 | 0.197267545 | 0.140820953 | 1.633128257 | 0.032093054 | 0.210676803 | 1.400839448 | 0.486291616 |
|  |  |  |  | 377.8585 | 744.4770382 | 0.124564375 | 0.081750475 | 1.714167178 | 0.025951423 | 0.20074743 | 1.523714394 | 0.607592508 |
|  |  | Nodularin | 14.39684718 | 377.8655 | 825.9818535 | 0.257473621 | 0.195340143 | 1.687269922 | 0.048483821 | 0.23310006 | 1.318078385 | 0.398436169 |
|  |  |  |  | 377.868 | 854.9994266 | 0.22596188 | 0.128515671 | 1.812836507 | 0.033208958 | 0.212166844 | 1.75824379 | 0.814135122 |
|  |  |  |  | 377.868 | 948.9834606 | 0.59058691 | 0.402620424 | 1.880219061 | 0.024579456 | 0.198025203 | 1.466857801 | 0.552729021 |
|  |  |  |  | 377.868 | 989.9873164 | 0.119149739 | 0.075923913 | 1.819437596 | 0.031082195 | 0.209254464 | 1.569330855 | 0.650149542 |
|  |  |  |  | 377.8695 | 731.9960247 | 0.38780104 | 0.240675229 | 1.69224677 | 0.044103018 | 0.226984743 | 1.611304337 | 0.68822901 |
|  |  | Linoleoyl-CoA | 13.25292249 | 377.8695 | 1030.985227 | 0.179555411 | 0.126175051 | 1.763480893 | 0.008313252 | 0.138961732 | 1.423065891 | 0.509002464 |
|  |  |  |  | 377.87 | 826.4792237 | 0.096570833 | 0.079481683 | 2.025960552 | 0.001375098 | 0.069933724 | 1.215007397 | 0.280965097 |
|  |  |  |  | 377.874 | 772.996587 | 0.314792751 | 0.199011566 | 1.736857864 | 0.035764294 | 0.215295802 | 1.581781183 | 0.661550037 |
|  |  |  |  | 377.874 | 949.9878437 | 0.131212372 | 0.088056856 | 1.919669947 | 0.004361717 | 0.106475886 | 1.490086939 | 0.575396507 |
|  |  |  |  | 377.8765 | 926.0240678 | 0.23023421 | 0.124473102 | 1.783593189 | 0.027079163 | 0.202827766 | 1.849670383 | 0.887268201 |
|  |  |  |  | 377.879 | 568.4925507 | 0.278080127 | 0.197597888 | 1.786404916 | 0.011220047 | 0.15650983 | 1.407303135 | 0.49293312 |
|  |  |  |  | 377.879 | 599.0167535 | 0.191622999 | 0.103426408 | 1.653878167 | 0.017577753 | 0.180025822 | 1.852747303 | 0.889666125 |
|  |  | Bayogenin 3-O-cellobioside | 15.94145241 | 377.879 | 814.0005367 | 0.260415535 | 0.161128186 | 1.805189208 | 0.024004031 | 0.196815011 | 1.616200994 | 0.692606626 |
|  |  |  |  | 377.879 | 1007.028135 | 0.527802985 | 0.299477314 | 1.676352459 | 0.042047325 | 0.223807762 | 1.762413914 | 0.81755279 |
|  |  |  |  | 377.88 | 579.9703402 | 0.415424915 | 0.3430765 | 1.643888695 | 0.039053192 | 0.218917212 | 1.210881287 | 0.276057433 |
|  |  | Patellamide A | 21.60094904 | 377.88 | 743.9748251 | 0.347131223 | 0.249028698 | 2.144305027 | 0.0001107 | 0.0351666 | 1.39394064 | 0.479169127 |
|  |  |  |  | 377.88 | 808.9378204 | 0.38274473 | 0.318909818 | 1.746326095 | 0.019524996 | 0.185694852 | 1.20016603 | 0.263234 |
|  |  |  |  | 377.88 | 1008.029953 | 0.129227011 | 0.075157216 | 1.737682847 | 0.016726901 | 0.177629913 | 1.719422548 | 0.781924131 |
|  |  |  |  | 377.881 | 562.9307759 | 0.528151516 | 0.458547242 | 1.553917661 | 0.042556492 | 0.224614739 | 1.151793028 | 0.203881495 |
|  |  | DG(18:4(6Z,9Z,12Z,15Z)/22:6(4Z,7Z,10Z,13Z,16Z,19Z)/0:0) | 2.760218019 | 377.881 | 661.974401 | 0.326944421 | 0.259071026 | 1.892195877 | 0.016776946 | 0.177775717 | 1.261987595 | 0.335697729 |
|  |  |  |  | 377.881 | 866.9805383 | 0.976520691 | 0.66308138 | 1.929427473 | 0.018513846 | 0.182673872 | 1.47270112 | 0.55846467 |
|  |  |  |  | 377.882 | 925.0256834 | 0.924876762 | 0.530723367 | 1.729443459 | 0.043513989 | 0.226096114 | 1.742672019 | 0.801301071 |
|  |  | Pristimycin IA | 19.85627766 | 377.883 | 867.9823912 | 0.192275841 | 0.133556246 | 1.822054027 | 0.032393753 | 0.211086327 | 1.439661912 | 0.525730051 |
|  |  |  |  | 377.884 | 526.9891514 | 0.818437657 | 0.560834587 | 1.878370467 | 0.021435184 | 0.190844542 | 1.45932094 | 0.545297202 |
|  |  |  |  | 377.884 | 726.9351523 | 0.489218881 | 0.411091858 | 1.794090078 | 0.010855622 | 0.154635042 | 1.190047605 | 0.251019286 |
|  |  |  |  | 377.885 | 620.9716681 | 2.59436031 | 1.883925052 | 1.869105192 | 0.026910193 | 0.202524512 | 1.37710378 | 0.461637286 |
|  |  |  |  | 377.885 | 703.9769725 | 0.318382784 | 0.223193038 | 1.771604971 | 0.038249618 | 0.218010761 | 1.426490661 | 0.512470301 |
|  |  | PE-NMe2(18:4(6Z,9Z,12Z,15Z)/18:4(6Z,9Z,12Z,15Z)) | 5.22187771 | 377.885 | 761.0172453 | 2.493616948 | 1.463123753 | 1.72969564 | 0.038213874 | 0.217973764 | 1.704310344 | 0.769188065 |
|  |  |  |  | 377.885 | 843.0191598 | 1.730391431 | 1.009132064 | 1.757586299 | 0.03637727 | 0.215993365 | 1.714732386 | 0.777983436 |
|  |  | Ginsenoside Rf | -2.269512225 | 377.8895 | 802.0181587 | 0.137933922 | 0.072779631 | 1.554523064 | 0.034603745 | 0.213920739 | 1.895227009 | 0.922370663 |
|  |  |  |  | 377.8915 | 271.0025146 | 1.394411267 | 0.98628299 | 1.726846197 | 0.044616592 | 0.227745905 | 1.413804437 | 0.499582575 |
|  |  |  |  | 377.8915 | 351.0006403 | 21.10824339 | 14.42590698 | 1.75792801 | 0.0379525 | 0.217701486 | 1.463217766 | 0.549144497 |
|  |  |  |  | 377.8915 | 374.9623311 | 4.641051197 | 3.649559011 | 1.864701275 | 0.023988337 | 0.196781403 | 1.271674518 | 0.346729464 |
|  |  |  |  | 377.8915 | 434.0072434 | 1.980985983 | 1.32806381 | 1.699383567 | 0.045258053 | 0.22867926 | 1.491634639 | 0.576894205 |
|  |  |  |  | 377.8915 | 456.9651305 | 3.083899481 | 2.342850886 | 1.891969224 | 0.024056214 | 0.196926524 | 1.316302074 | 0.396490607 |
|  |  |  |  | 377.8915 | 496.9583968 | 0.482031333 | 0.418933044 | 1.595962606 | 0.040826533 | 0.221816079 | 1.150616644 | 0.202407245 |
|  |  |  |  | 377.8915 | 517.0118955 | 0.40043379 | 0.233484921 | 1.762850418 | 0.011586565 | 0.158318522 | 1.715030627 | 0.77823434 |
|  |  |  |  | 377.8915 | 538.9684765 | 3.611559955 | 2.738002971 | 1.892513416 | 0.023578939 | 0.195893086 | 1.319048954 | 0.399498109 |
|  |  | erythro-6,8-Hexatriacontanediol | -12.89175374 | 377.8915 | 539.9719283 | 0.403790522 | 0.296779254 | 1.898660864 | 0.019259571 | 0.184923168 | 1.3605753 | 0.444216804 |
|  |  |  |  | 377.8915 | 597.0110623 | 6.234040089 | 3.851103335 | 1.745859086 | 0.036607343 | 0.216250291 | 1.618767285 | 0.694895598 |
|  |  |  |  | 377.8915 | 680.0186821 | 0.655626909 | 0.395544294 | 1.858111985 | 0.02342316 | 0.195549088 | 1.657530948 | 0.729035808 |
|  |  | PE-NMe(14:0/18:2(9Z,12Z)) | 1.418354216 | 377.8915 | 702.9752722 | 1.940214806 | 1.398849472 | 1.926874232 | 0.016313227 | 0.176400033 | 1.38700757 | 0.471975662 |
|  |  |  |  | 377.8915 | 784.9775957 | 1.479371166 | 1.047261613 | 1.852796464 | 0.028480857 | 0.20523651 | 1.412608986 | 0.498362179 |
|  |  |  |  | 377.896 | 293.9626474 | 0.641753356 | 0.537245639 | 1.720218932 | 0.040129705 | 0.220641751 | 1.194525017 | 0.256437068 |
|  |  | Galactosylceramide (d18:1/12:0) | -13.97123917 | 377.896 | 644.93328 | 0.568271091 | 0.464294057 | 1.684396506 | 0.029251361 | 0.206484356 | 1.223946513 | 0.291540513 |
|  |  | 4-Bromo-3,5-cyclohexadiene-1,2-dione | 3.585433962 | 377.9005 | 187.998747 | 3.817359869 | 2.89292494 | 1.821870679 | 0.029043148 | 0.206152205 | 1.319550264 | 0.400046307 |
|  |  |  |  | 377.9005 | 268.997993 | 115.923031 | 82.63518468 | 1.75315986 | 0.036415149 | 0.216035846 | 1.40282897 | 0.48833913 |
|  |  |  |  | 377.9005 | 270.0014444 | 7.748758642 | 5.514843661 | 1.77010568 | 0.036873633 | 0.216544406 | 1.405073129 | 0.49064522 |
|  |  |  |  | 377.9005 | 292.9600488 | 11.59799094 | 9.304560125 | 1.875983319 | 0.021149384 | 0.190116293 | 1.246484603 | 0.317865063 |
|  |  |  |  | 377.9005 | 352.005369 | 1.899018086 | 1.27410228 | 1.811806685 | 0.029323403 | 0.206598429 | 1.490475385 | 0.575772549 |
|  |  |  |  | 377.9005 | 390.9938702 | 0.290975946 | 0.2240422 | 1.907953206 | 0.023477303 | 0.195669029 | 1.298755083 | 0.377129395 |
|  |  |  |  | 377.9005 | 433.0039329 | 18.69856313 | 12.27976155 | 1.741328361 | 0.03821476 | 0.217974681 | 1.522713861 | 0.606644865 |
|  |  |  |  | 377.9005 | 515.0076681 | 13.42414601 | 8.54609519 | 1.751766579 | 0.03620335 | 0.21579739 | 1.570792943 | 0.651493022 |
|  |  |  |  | 377.9005 | 516.0102492 | 1.692501149 | 1.09165224 | 1.74090919 | 0.03576732 | 0.215299293 | 1.550403221 | 0.632643473 |
|  |  | CE(14:0) | -2.799016059 | 377.9005 | 598.0148056 | 0.980483886 | 0.620913665 | 1.664472452 | 0.04845085 | 0.233057044 | 1.579098581 | 0.659101239 |
|  |  |  |  | 377.9005 | 621.9741206 | 0.363899233 | 0.264358138 | 1.916832215 | 0.019726399 | 0.186270708 | 1.376538798 | 0.461045273 |
|  |  |  |  | 377.9005 | 679.0154519 | 3.66145647 | 2.181254298 | 1.749404746 | 0.034539782 | 0.213842796 | 1.678601378 | 0.74725967 |
|  |  |  |  | 377.9105 | 186.9953749 | 85.25434071 | 65.01225131 | 1.798692748 | 0.031966576 | 0.210502732 | 1.311358075 | 0.391061677 |
|  |  | Dimethyl diselenide | 12.44335893 | 377.9105 | 188.9996158 | 0.680599732 | 0.513331643 | 1.726369715 | 0.036877248 | 0.216548376 | 1.325847999 | 0.406915388 |
|  |  |  |  | 377.9105 | 228.022285 | 1.091509805 | 0.805002302 | 1.797266049 | 0.034430466 | 0.21370905 | 1.355908925 | 0.439260277 |
|  |  |  |  | 377.9195 | 104.9927482 | 110.6550212 | 88.28381892 | 1.813719351 | 0.030175068 | 0.207914751 | 1.253400935 | 0.325847974 |
|  |  |  |  | 377.9195 | 106.9968407 | 0.501143368 | 0.402149911 | 1.682374591 | 0.045131279 | 0.228496301 | 1.246160586 | 0.317489993 |
|  |  |  |  | 377.9195 | 146.0188616 | 18.42906793 | 14.61254669 | 1.801493276 | 0.032839243 | 0.211682087 | 1.261181115 | 0.334775472 |
|  |  |  |  | 377.9195 | 226.9879827 | 10.30982398 | 8.626341635 | 1.821874441 | 0.012305974 | 0.161661926 | 1.195156002 | 0.257198944 |
|  |  |  |  | 377.9435 | 105.9960719 | 2.605388795 | 2.03974618 | 1.860016176 | 0.025767143 | 0.200394467 | 1.277310295 | 0.353109039 |
|  |  |  |  | 377.9435 | 254.9830013 | 0.199871505 | 0.14281725 | 1.781061586 | 0.034089941 | 0.21328802 | 1.399491343 | 0.484902562 |
|  |  |  |  | 377.964 | 144.9848039 | 0.658926604 | 0.568533333 | 1.764876994 | 0.011986525 | 0.160209703 | 1.158993793 | 0.21287284 |
|  |  | 1-Hexadecanoyl-2-(9Z-octadecenoyl)-sn-glycero-3-phosphoserine | 10.69277198 | 377.964 | 763.0213246 | 0.107860124 | 0.065655082 | 1.503073534 | 0.037418747 | 0.217135838 | 1.642829768 | 0.716182994 |
|  |  |  |  | 378.2845 | 128.9537843 | 42.27880392 | 35.36128556 | 1.793446939 | 0.015163822 | 0.172734422 | 1.195624063 | 0.257763838 |
|  |  |  |  | 378.705 | 262.9815931 | 0.196394147 | 0.130432378 | 1.561248852 | 0.022555129 | 0.193569021 | 1.505716218 | 0.590449892 |
|  |  | Cohibin C | 1.54298192 | 379.637 | 577.9415668 | 0.005363689 | 0.014886223 | 1.92330608 | 0.000282607 | 0.049599831 | 0.36031229 | -1.472680234 |
|  |  |  |  | 380.488 | 107.9671226 | 0.032711514 | 0.024299906 | 1.36103178 | 0.044605418 | 0.227729478 | 1.346158082 | 0.428847838 |
|  |  | 9,10-12,13-diepoxy-octadecanoate | -15.0548341 | 381.459 | 626.882854 | 0.020052351 | 0.012727138 | 1.949723378 | 0.001776271 | 0.075073775 | 1.575558548 | 0.655863366 |
|  |  |  |  | 382.185 | 422.8849626 | 0.077464249 | 0.056226663 | 1.631731349 | 0.02545398 | 0.199785851 | 1.37771379 | 0.462276209 |
|  |  |  |  | 382.2 | 385.9404538 | 0.007764554 | 0.011607998 | 1.502146743 | 0.016249806 | 0.176207497 | 0.668896963 | -0.5801441 |
|  |  |  |  | 382.247 | 239.9831614 | 0.008217345 | 0.012282906 | 1.57724721 | 0.02579136 | 0.200441067 | 0.669006569 | -0.579907719 |
|  |  |  |  | 382.247 | 462.8770597 | 0.032232702 | 0.024432229 | 1.651459982 | 0.025602448 | 0.200075785 | 1.319269787 | 0.399739622 |
|  |  |  |  | 382.263 | 91.05882777 | 0.034246046 | 0.051850482 | 1.516076906 | 0.025920985 | 0.200689391 | 0.660476907 | -0.598419975 |
|  |  |  |  | 382.2735 | 356.9134685 | 0.122305799 | 0.097629762 | 1.562821644 | 0.038701907 | 0.218524663 | 1.25275117 | 0.325099885 |
|  |  |  |  | 382.2875 | 345.9474077 | 0.01372069 | 0.018527379 | 1.708237569 | 0.008425103 | 0.13976476 | 0.740562924 | -0.433305772 |
|  |  |  |  | 382.3395 | 274.9105736 | 0.433293591 | 0.269031244 | 2.158114147 | 2.48474E-05 | 0.02242259 | 1.610569776 | 0.687571165 |
|  |  |  |  | 382.425 | 634.8142949 | 0.011186079 | 0.006018316 | 1.833677063 | 0.00679186 | 0.126680786 | 1.858672626 | 0.894272686 |
|  |  | Terodiline hydrochloride | -9.380359457 | 382.793 | 318.9002946 | 0.023590142 | 0.016475747 | 1.903868948 | 0.002420546 | 0.084609959 | 1.431810149 | 0.517840211 |
|  |  |  |  | 382.793 | 340.8825348 | 0.141588569 | 0.108273956 | 1.694949302 | 0.023394699 | 0.195485875 | 1.307688157 | 0.387018544 |
|  |  |  |  | 383.027 | 446.8471544 | 0.076949732 | 0.053972542 | 1.786288736 | 0.016932261 | 0.178224222 | 1.425719975 | 0.511690651 |
|  |  |  |  | 383.103 | 486.8384922 | 0.036530918 | 0.025110549 | 1.51763866 | 0.048937878 | 0.233688143 | 1.454803651 | 0.540824451 |
|  |  | Oxamate | -20.24193509 | 383.1605 | 90.05557405 | 1.15288145 | 1.813952163 | 1.645402163 | 0.013345371 | 0.16606093 | 0.635563315 | -0.65389224 |
|  |  |  |  | 383.192 | 380.8744 | 0.088599118 | 0.06038756 | 1.730492325 | 0.017707771 | 0.180377007 | 1.467174985 | 0.553040947 |
|  |  |  |  | 383.285 | 592.8038306 | 0.016343268 | 0.010918602 | 1.759692638 | 0.02553949 | 0.199953148 | 1.496827971 | 0.581908424 |
|  |  |  |  | 383.314 | 658.7751643 | 0.019704235 | 0.011888347 | 1.577140329 | 0.04748091 | 0.231772232 | 1.65744105 | 0.728957559 |
|  |  | Rifamycin | -13.83327286 | 383.314 | 698.7662242 | 0.00984162 | 0.005907317 | 1.60580455 | 0.049474413 | 0.234372855 | 1.666005156 | 0.736392866 |
|  |  |  |  | 384.844 | 304.0081199 | 0.00693649 | 0.003683091 | 1.545905184 | 0.04143583 | 0.222820347 | 1.883333642 | 0.913288603 |
|  |  |  |  | 385.021 | 162.1127318 | 0.421391349 | 0.662771715 | 1.783219343 | 0.021620423 | 0.191309165 | 0.635801648 | -0.653351339 |
|  |  |  |  | 385.043 | 286.9426355 | 0.004219405 | 0.001800163 | 1.790229857 | 0.016460518 | 0.176843054 | 2.343901953 | 1.228912222 |
|  |  |  |  | 385.0635 | 132.0769621 | 0.096286796 | 0.159243066 | 1.849547943 | 0.00217152 | 0.080811195 | 0.604652992 | -0.725820673 |
|  |  |  |  | 385.7555 | 219.1343052 | 0.009284674 | 0.015099136 | 1.499940958 | 0.016080325 | 0.175687637 | 0.614914258 | -0.701542837 |
|  |  |  |  | 386.137 | 129.0662084 | 0.134980662 | 0.185724084 | 1.676546005 | 0.020463111 | 0.188308865 | 0.726780605 | -0.460408174 |
|  |  |  |  | 388.916 | 358.9787186 | 0.004108754 | 0.002437435 | 1.791119536 | 0.009670385 | 0.147947496 | 1.685687692 | 0.753337272 |
|  |  |  |  | 389.249 | 400.9896512 | 0.003491953 | 0.001501461 | 1.447491123 | 0.044915044 | 0.228182532 | 2.32570403 | 1.217667511 |
|  |  | S-Methyl-3-phospho-1-thio-D-glycerate | -9.064631732 | 390.696 | 217.1547173 | 0.010418885 | 0.00800856 | 1.627882238 | 0.020630589 | 0.18875781 | 1.300968643 | 0.37958619 |
|  |  |  |  | 392.3295 | 150.0138082 | 0.119940849 | 0.085348099 | 1.556121322 | 0.036150495 | 0.21573753 | 1.405313645 | 0.490892154 |
|  |  |  |  | 394.1145 | 333.0864513 | 0.038186011 | 0.033863383 | 1.570944214 | 0.03163003 | 0.210034199 | 1.127649029 | 0.173318111 |
|  |  |  |  | 396.428 | 224.9726649 | 0.025274476 | 0.018171249 | 1.710577852 | 0.021407958 | 0.190775768 | 1.390904674 | 0.476023548 |
|  |  |  |  | 396.71 | 172.0604063 | 0.048767469 | 0.061696653 | 1.376454368 | 0.046116168 | 0.229898674 | 0.790439469 | -0.339273108 |
|  |  |  |  | 396.71 | 290.9438652 | 0.007439927 | 0.003766426 | 1.801758465 | 0.011296343 | 0.156892533 | 1.975327928 | 0.982092178 |
|  |  |  |  | 399.084 | 164.9300027 | 0.00510153 | 0.00301325 | 1.75569642 | 0.009421961 | 0.146418329 | 1.69303265 | 0.759609796 |
|  |  |  |  | 401.287 | 171.0879332 | 0.507011735 | 0.61858866 | 1.374911507 | 0.043124083 | 0.225498475 | 0.819626623 | -0.286961248 |
|  |  |  |  | 401.3115 | 172.0912762 | 0.027570883 | 0.035069952 | 1.43134184 | 0.042885345 | 0.225128767 | 0.786168272 | -0.347089954 |
|  |  |  |  | 402.2435 | 128.9537732 | 3.167345703 | 1.929065333 | 1.74117609 | 0.019772684 | 0.18640189 | 1.641906912 | 0.715372335 |
|  |  |  |  | 402.526 | 236.992597 | 0.002865229 | 0.004044247 | 1.59196172 | 0.018953657 | 0.184015248 | 0.708470228 | -0.497220867 |
|  |  | 3-hydroxy-2-isobutyrate | 21.54118881 | 402.619 | 104.1074975 | 0.696118515 | 0.584687924 | 1.695028404 | 0.031887464 | 0.210393295 | 1.19058131 | 0.251666152 |
|  |  |  |  | 402.892 | 297.0692268 | 0.016958845 | 0.022778258 | 1.478655401 | 0.037227694 | 0.216930157 | 0.744518962 | -0.425619502 |
|  |  |  |  | 403.1595 | 242.0016343 | 0.010433626 | 0.014956641 | 1.862306473 | 0.004078838 | 0.103983582 | 0.697591509 | -0.519545616 |
|  |  |  |  | 403.1675 | 220.0191484 | 0.027537517 | 0.048204274 | 2.115770169 | 0.000323179 | 0.051354528 | 0.571267114 | -0.807762613 |
|  |  | Aerophobin 1 | -9.720588583 | 403.199 | 477.1225484 | 0.001947205 | 0.004691733 | 1.945571259 | 0.043022715 | 0.225341851 | 0.415028857 | -1.268716446 |
|  |  |  |  | 403.22 | 417.0489374 | 0.000404244 | 0.00266769 | 1.781963023 | 0.000436534 | 0.054865244 | 0.151533446 | -2.722291835 |
|  |  |  |  | 403.317 | 198.037538 | 0.005053202 | 0.010555048 | 2.078782882 | 5.98934E-05 | 0.030860417 | 0.478747379 | -1.062663507 |
|  |  |  |  | 405.2935 | 215.1027209 | 0.032149467 | 0.024540883 | 1.610179843 | 0.016332863 | 0.176459429 | 1.310037077 | 0.389607644 |
|  |  |  |  | 408.6045 | 170.0424689 | 0.933346469 | 0.779747643 | 1.899842078 | 0.002870558 | 0.090420474 | 1.196985303 | 0.259405438 |
|  |  |  |  | 408.6275 | 596.0404419 | 0.026369895 | 0.016251433 | 1.595372208 | 0.00916516 | 0.144786094 | 1.622619684 | 0.698324895 |
|  |  |  |  | 408.678 | 107.0658002 | 0.081134349 | 0.068683402 | 1.974446678 | 0.001805902 | 0.075385383 | 1.181280286 | 0.240351318 |
|  |  |  |  | 408.678 | 192.0243342 | 0.546701057 | 0.474468227 | 1.944963105 | 0.013807634 | 0.167871556 | 1.152239553 | 0.204440687 |
|  |  |  |  | 408.795 | 260.0270958 | 0.008702697 | 0.01459281 | 1.514043165 | 0.025500499 | 0.199876967 | 0.596368837 | -0.745723222 |
|  |  |  |  | 408.869 | 175.0536313 | 0.2494682 | 0.433105177 | 1.732189522 | 0.005646823 | 0.115565237 | 0.575999119 | -0.79586149 |
|  |  |  |  | 408.869 | 238.0451599 | 0.032276499 | 0.044084485 | 1.420672741 | 0.035940665 | 0.215498484 | 0.732150989 | -0.449786894 |
|  |  |  |  | 410.8885 | 190.0645294 | 0.191565457 | 0.268939562 | 1.626684166 | 0.014217998 | 0.169410679 | 0.71229928 | -0.489444563 |
|  |  |  |  | 412.6245 | 193.0643054 | 0.007001363 | 0.01199395 | 1.418643059 | 0.040452827 | 0.22118977 | 0.583741268 | -0.77659903 |
|  |  | Nitrogen mustard | 0.629671343 | 412.9135 | 157.0608749 | 0.072295344 | 0.053758551 | 1.712406346 | 0.025895993 | 0.200641659 | 1.34481571 | 0.427408484 |
|  |  |  |  | 413.2575 | 103.0329491 | 0.001376187 | 0.013391572 | 1.596702866 | 0.046054709 | 0.229812423 | 0.102765177 | -3.282576625 |
|  |  |  |  | 414.534 | 122.0813751 | 1.005079655 | 0.905487399 | 1.595021325 | 0.039659006 | 0.219832458 | 1.109987457 | 0.150543375 |
|  |  |  |  | 415.498 | 376.9933449 | 0.012916365 | 0.009370429 | 1.604836243 | 0.0294698 | 0.206828904 | 1.37841762 | 0.463013048 |
|  |  |  |  | 416.046 | 70.02947374 | 0.000489794 | 7.0591E-05 | 1.946642849 | 0.018167492 | 0.181588862 | 6.938470269 | 2.794617626 |
|  |  |  |  | 417.352 | 203.0664038 | 0.004950101 | 0.004067971 | 1.598434647 | 0.02647461 | 0.201729242 | 1.216847604 | 0.283148499 |
|  |  |  |  | 417.5625 | 702.9100697 | 0.009737877 | 0.006604871 | 1.680723292 | 0.040865895 | 0.221881585 | 1.474347801 | 0.560076898 |
|  |  | Riccionidin A | 7.371440018 | 418.323 | 286.2377791 | 0.002209378 | 0.001518553 | 1.471180903 | 0.029989419 | 0.207632782 | 1.45492272 | 0.540942524 |
|  |  |  |  | 419.802 | 163.0793601 | 0.017279052 | 0.026883481 | 1.95182414 | 0.003974949 | 0.103011885 | 0.642738625 | -0.637695921 |
|  |  |  |  | 421.1415 | 223.0004756 | 0.018433732 | 0.009798649 | 1.745208951 | 0.017376226 | 0.179473879 | 1.881252457 | 0.911693466 |
|  |  |  |  | 421.315 | 209.032199 | 0.055558305 | 0.029905455 | 2.052111421 | 0.000927651 | 0.061177908 | 1.857798343 | 0.893593911 |
|  |  |  |  | 421.415 | 241.0111877 | 0.180296334 | 0.111525744 | 2.1046342 | 0.000669721 | 0.05885959 | 1.616634221 | 0.692993292 |
|  |  |  |  | 422.134 | 176.0378145 | 0.056532543 | 0.076334178 | 1.326275312 | 0.048289348 | 0.232845721 | 0.740592809 | -0.433247553 |
|  |  |  |  | 423.768 | 274.0685023 | 0.004366391 | 0.008587138 | 1.745787622 | 0.004855089 | 0.110351365 | 0.50848035 | -0.975736072 |
|  |  |  |  | 423.9305 | 323.064023 | 0.011215511 | 0.007267001 | 1.990536843 | 0.002153498 | 0.080517133 | 1.543347977 | 0.626063382 |
|  |  |  |  | 424.05 | 230.0141542 | 0.010342212 | 0.005698664 | 2.038900746 | 0.000519463 | 0.056627186 | 1.814848523 | 0.859849138 |
|  |  |  |  | 424.1365 | 246.0378074 | 0.356621783 | 0.196103925 | 2.030833893 | 0.000575096 | 0.057568621 | 1.818534655 | 0.862776419 |
|  |  |  |  | 424.163 | 272.9745023 | 0.037870563 | 0.024923169 | 1.969331318 | 0.002347373 | 0.083542461 | 1.519492278 | 0.603589343 |
|  |  |  |  | 424.1795 | 250.9930666 | 0.062570416 | 0.033843736 | 1.971653283 | 0.003264185 | 0.095391237 | 1.848803426 | 0.886591839 |
|  |  |  |  | 424.269 | 500.9786188 | 0.00303235 | 0.001191673 | 2.054954985 | 0.000904542 | 0.061018477 | 2.544615429 | 1.347447636 |
|  |  |  |  | 424.481 | 532.1146915 | 0.00269096 | 0.001420173 | 1.192065448 | 0.016859023 | 0.178013479 | 1.894810985 | 0.922053941 |
|  |  |  |  | 424.5425 | 270.0371587 | 0.022940139 | 0.013807068 | 1.805811136 | 0.008326208 | 0.139055374 | 1.66147798 | 0.732467172 |
|  |  |  |  | 424.612 | 559.0969244 | 0.006389093 | 0.004449199 | 1.488595242 | 0.028648298 | 0.205512089 | 1.436009789 | 0.522065584 |
|  |  |  |  | 425.491 | 261.175466 | 0.024383593 | 0.01464541 | 1.622143572 | 0.013254991 | 0.165696941 | 1.664930771 | 0.735462191 |
|  |  | Bromazepam | -18.95023122 | 426.0375 | 317.1542854 | 0.002768017 | 0.001733841 | 1.424133733 | 0.048422219 | 0.233019655 | 1.596464744 | 0.674880693 |
|  |  |  |  | 427.156 | 146.0448975 | 0.044422545 | 0.026459825 | 1.97180258 | 0.004021276 | 0.103449135 | 1.678867667 | 0.747488518 |
|  |  |  |  | 427.531 | 244.0220087 | 0.008033503 | 0.003314659 | 1.725555974 | 0.002356617 | 0.083679451 | 2.423628846 | 1.277168782 |
|  |  |  |  | 428.7745 | 259.0214521 | 0.199094785 | 0.130538208 | 2.01497711 | 0.003571532 | 0.098912822 | 1.525183985 | 0.608983287 |
|  |  |  |  | 428.951 | 529.2624016 | 0.002084415 | 0.00071571 | 1.64207788 | 0.000402271 | 0.053969311 | 2.912373672 | 1.542195472 |
|  |  |  |  | 429.0105 | 164.0819558 | 0.263705878 | 0.196312862 | 1.571839481 | 0.033489751 | 0.212529303 | 1.343293941 | 0.425775032 |
|  |  |  |  | 429.025 | 131.0452636 | 0.024069864 | 0.015869212 | 1.63194196 | 0.008526489 | 0.140482218 | 1.516764959 | 0.60099754 |
|  |  |  |  | 429.4785 | 85.04025659 | 0.054214019 | 0.040727199 | 1.488635335 | 0.027302089 | 0.203223474 | 1.331150188 | 0.412673354 |
|  |  |  |  | 429.493 | 179.0286957 | 0.030038608 | 0.022738259 | 1.434504411 | 0.036898239 | 0.21657141 | 1.321060187 | 0.401696197 |
|  |  |  |  | 429.493 | 347.0793786 | 0.010953145 | 0.008111377 | 1.563163334 | 0.01485178 | 0.171670658 | 1.350343432 | 0.433326374 |
|  |  | 2,6-Dichloro-para-phenylenediamine | -12.67353915 | 429.578 | 178.036233 | 0.033229275 | 0.026029208 | 1.591510786 | 0.032402811 | 0.211098569 | 1.276614912 | 0.352323405 |
|  |  |  |  | 429.697 | 307.0793384 | 0.072122071 | 0.05646031 | 1.547968372 | 0.033343988 | 0.212341753 | 1.277394174 | 0.353203775 |
|  |  | 2-Deoxy-D-ribose 1-phosphate | -17.19124724 | 431.1385 | 215.1139958 | 0.002856044 | 0.00457589 | 1.67683294 | 0.017985927 | 0.181115716 | 0.624150618 | -0.680033877 |
|  |  |  |  | 431.66 | 410.0529783 | 0.007944485 | 0.004639322 | 1.767634947 | 0.010051985 | 0.150206166 | 1.712423657 | 0.776039671 |
|  |  |  |  | 431.674 | 297.0599626 | 0.022334127 | 0.018003232 | 1.590762428 | 0.031607375 | 0.210002376 | 1.240562107 | 0.310993963 |
|  |  |  |  | 431.7005 | 276.0479118 | 0.046358557 | 0.029515707 | 1.647629616 | 0.033579064 | 0.212643577 | 1.57064026 | 0.651352783 |
|  |  |  |  | 432.306 | 186.0987643 | 0.050274404 | 0.066068446 | 1.453364938 | 0.026873857 | 0.202458922 | 0.760944237 | -0.394137359 |
|  |  |  |  | 433.097 | 344.0518454 | 0.02039357 | 0.016522914 | 1.551700243 | 0.043724405 | 0.2264155 | 1.234259847 | 0.303646155 |
|  |  |  |  | 433.9245 | 316.0577638 | 0.006631513 | 0.003730333 | 1.698302816 | 0.036116896 | 0.215699403 | 1.777727076 | 0.830033852 |
|  |  | Ferricyanide | 12.76649166 | 434.88 | 212.9593825 | 0.030164734 | 0.018073939 | 1.958883223 | 0.009836755 | 0.148945333 | 1.668962906 | 0.73895189 |
|  |  |  |  | 435.091 | 160.042882 | 0.021664425 | 0.047924379 | 1.860542239 | 0.013772223 | 0.167735799 | 0.452054367 | -1.145431804 |
|  |  |  |  | 437.256 | 277.0489385 | 0.018396253 | 0.015116316 | 1.639168471 | 0.020777651 | 0.189147791 | 1.216979924 | 0.283305368 |
|  |  |  |  | 438.4285 | 243.0376234 | 0.023461224 | 0.019301715 | 1.520144278 | 0.043264244 | 0.225714186 | 1.215499447 | 0.281549237 |
|  |  |  |  | 439.07 | 272.0879988 | 0.225826846 | 0.198742561 | 1.675084058 | 0.024642901 | 0.198156064 | 1.136278233 | 0.184316142 |
|  |  |  |  | 439.402 | 293.0975859 | 0.242266613 | 0.15871843 | 1.329283187 | 0.037447128 | 0.217166245 | 1.526392444 | 0.610125935 |
|  |  |  |  | 439.5365 | 256.0219247 | 0.030864828 | 0.020809789 | 1.895321838 | 0.008632603 | 0.141222732 | 1.483187911 | 0.56870139 |
|  |  |  |  | 439.9015 | 809.0120604 | 0.003871258 | 0.001909382 | 1.128374707 | 0.035548723 | 0.215045867 | 2.027492347 | 1.019696469 |
|  |  |  |  | 440.215 | 241.0111186 | 0.03453921 | 0.024452003 | 1.820869332 | 0.0169681 | 0.178326866 | 1.412530906 | 0.498282433 |
|  |  |  |  | 440.38 | 955.1498972 | 0.021467798 | 0.01338965 | 1.414971091 | 0.048907104 | 0.233648538 | 1.603312859 | 0.68105597 |
|  |  |  |  | 440.569 | 108.0448583 | 0.027912515 | 0.020936825 | 1.939980552 | 0.005960414 | 0.118685715 | 1.333177973 | 0.414869386 |
|  |  |  |  | 441.3425 | 119.0354948 | 0.052272077 | 0.037785957 | 1.622473929 | 0.023851687 | 0.1964874 | 1.383373117 | 0.468190326 |
|  |  |  |  | 441.6075 | 348.0695136 | 15.22100454 | 11.19872804 | 1.548083384 | 0.028995325 | 0.206075394 | 1.359172621 | 0.442728697 |
|  |  |  |  | 441.6075 | 349.0745276 | 1.693097676 | 1.213674363 | 1.618643613 | 0.019831908 | 0.186569115 | 1.395018076 | 0.480283816 |
|  |  | Captafol | 20.2174154 | 441.69 | 350.0753337 | 0.26648864 | 0.197212163 | 1.480150809 | 0.041834915 | 0.223467058 | 1.351278929 | 0.434325505 |
|  |  |  |  | 442.5465 | 266.0883744 | 0.10690472 | 0.097717804 | 1.783264637 | 0.006499885 | 0.123991132 | 1.094014764 | 0.129632208 |
|  |  |  |  | 442.5845 | 370.0528528 | 0.23903324 | 0.170461365 | 1.550601897 | 0.030293519 | 0.208093247 | 1.402272239 | 0.487766463 |
|  |  |  |  | 443.0385 | 426.0481742 | 0.408821782 | 0.339490266 | 1.588323614 | 0.027642676 | 0.203818605 | 1.204222395 | 0.268101853 |
|  |  |  |  | 443.0765 | 450.0847754 | 0.064687051 | 0.051958173 | 1.771319028 | 0.01365816 | 0.167295241 | 1.244983167 | 0.316126237 |
|  |  |  |  | 443.414 | 468.0943852 | 0.160921526 | 0.136740455 | 1.591337665 | 0.031144946 | 0.209344871 | 1.176839186 | 0.234917191 |
|  |  | Tricalcium phosphate | -14.53934757 | 443.495 | 311.1794668 | 0.031590568 | 0.0170319 | 2.195079856 | 0.001304631 | 0.068807071 | 1.854788174 | 0.891254434 |
|  |  |  |  | 443.5115 | 469.0976553 | 0.028706366 | 0.023743942 | 1.573651624 | 0.024535636 | 0.197934529 | 1.20899748 | 0.273811237 |
|  |  |  |  | 444.032 | 282.1185908 | 0.009206832 | 0.007411067 | 1.834943872 | 0.001953308 | 0.077394977 | 1.242308614 | 0.313023612 |
|  |  |  |  | 444.361 | 319.1250566 | 0.009479866 | 0.007262963 | 1.597046915 | 0.022955814 | 0.194496611 | 1.305233913 | 0.384308378 |
|  |  |  |  | 445.505 | 381.1270712 | 0.019156525 | 0.012538422 | 1.462846684 | 0.035999029 | 0.215565201 | 1.52782589 | 0.611480144 |
|  |  |  |  | 445.683 | 175.0536385 | 0.060771218 | 0.100845685 | 1.784551113 | 0.005404795 | 0.114085054 | 0.602615941 | -0.730689258 |
|  |  | SWEP | -3.590919162 | 446.223 | 221.0591864 | 0.068413549 | 0.094521881 | 1.440100704 | 0.027927785 | 0.204308236 | 0.723785309 | -0.466366271 |
|  |  |  |  | 446.476 | 122.0813937 | 1.168726513 | 0.996298413 | 1.735347671 | 0.015869145 | 0.175028766 | 1.173068729 | 0.230287542 |
|  |  |  |  | 447.048 | 208.000625 | 0.007023223 | 0.004631276 | 1.612894829 | 0.033473857 | 0.212508916 | 1.516476993 | 0.60072361 |
|  |  |  |  | 449.6295 | 148.0605239 | 5.683744946 | 2.493229546 | 1.725962499 | 0.030176422 | 0.207916797 | 2.279671744 | 1.188826102 |
|  |  |  |  | 450.552 | 347.0249657 | 0.053902545 | 0.030546321 | 1.843002771 | 0.003773839 | 0.101036461 | 1.764616623 | 0.819354781 |
|  |  |  |  | 451.482 | 326.0471914 | 0.083734023 | 0.044662053 | 1.754397424 | 0.004521704 | 0.107793985 | 1.874835954 | 0.906764367 |
|  |  |  |  | 451.938 | 212.0430493 | 0.041145785 | 0.019879301 | 2.112786676 | 0.004896163 | 0.110650056 | 2.069780256 | 1.049477608 |
|  |  |  |  | 452.027 | 227.0660865 | 0.039109194 | 0.024791718 | 1.398722385 | 0.036991906 | 0.216673933 | 1.577510453 | 0.657649565 |
|  |  |  |  | 452.369 | 204.0870701 | 0.18592766 | 0.161246427 | 1.6491148 | 0.032028793 | 0.210588496 | 1.153065301 | 0.205474219 |
|  |  |  |  | 452.796 | 376.085688 | 0.013564727 | 0.020622524 | 1.676773464 | 0.013776165 | 0.167750935 | 0.657762685 | -0.60436093 |
|  |  | Isopentenyl diphosphate | -22.34055515 | 453.773 | 247.0938788 | 27.03052694 | 21.83051985 | 1.942642529 | 0.00223359 | 0.081803253 | 1.238198958 | 0.308243151 |
|  |  | Isocil | 2.316683791 | 453.773 | 248.096949 | 2.852715102 | 2.253934954 | 1.980544947 | 0.001411099 | 0.07047882 | 1.265659906 | 0.339889792 |
|  |  |  |  | 454.091 | 249.1001176 | 0.146264721 | 0.120675491 | 1.580868158 | 0.042063136 | 0.223833026 | 1.212049929 | 0.27744913 |
|  |  |  |  | 454.219 | 327.0605213 | 0.087313245 | 0.059195715 | 1.958732725 | 0.00093353 | 0.061217336 | 1.474992662 | 0.560707777 |
|  |  |  |  | 454.4565 | 490.1192015 | 0.023332282 | 0.015783673 | 1.832321376 | 0.013972075 | 0.168495778 | 1.478254284 | 0.563894459 |
|  |  |  |  | 454.649 | 253.0082447 | 0.11500241 | 0.065444905 | 2.02524785 | 0.00471872 | 0.109334631 | 1.75724007 | 0.813311302 |
|  |  |  |  | 455.0245 | 98.98461785 | 0.490646764 | 0.260059889 | 1.839642898 | 0.002528118 | 0.086111909 | 1.886668355 | 0.915840843 |
|  |  |  |  | 455.1175 | 214.0192671 | 0.079081663 | 0.03797018 | 2.155310419 | 0.000450694 | 0.055203276 | 2.082730786 | 1.058476368 |
|  |  |  |  | 455.185 | 254.0423047 | 0.082685218 | 0.038207315 | 2.132266439 | 0.000358873 | 0.052644827 | 2.164120056 | 1.113780536 |
|  |  |  |  | 455.2365 | 424.0496923 | 0.009215147 | 0.015853845 | 1.501741415 | 0.042838055 | 0.225055191 | 0.581256256 | -0.782753756 |
|  |  |  |  | 455.246 | 230.042319 | 0.37317559 | 0.211471542 | 2.096951582 | 0.000709385 | 0.059311563 | 1.764661035 | 0.81939109 |
|  |  |  |  | 455.2585 | 231.0267568 | 0.075137401 | 0.042880826 | 2.102262835 | 0.000917861 | 0.061111248 | 1.752237719 | 0.809198513 |
|  |  |  |  | 455.271 | 115.0393659 | 0.067331645 | 0.028112237 | 2.081122129 | 0.002829556 | 0.089939066 | 2.395100928 | 1.260086452 |
|  |  |  |  | 455.455 | 212.0322242 | 0.094172832 | 0.055126763 | 1.965205117 | 0.006264472 | 0.121731796 | 1.708296062 | 0.772558027 |
|  |  |  |  | 455.598 | 215.0204201 | 0.01468269 | 0.005862803 | 1.897850036 | 0.01224364 | 0.161382474 | 2.504380464 | 1.324453752 |
|  |  | 4-Hydroxycyclophosphamide | 21.04039313 | 455.6235 | 278.0985066 | 0.006864172 | 0.004782024 | 1.578714984 | 0.015289711 | 0.173154894 | 1.435411377 | 0.521464261 |
|  |  |  |  | 456.6635 | 259.0213092 | 0.075600523 | 0.046284808 | 1.924793066 | 0.009969967 | 0.149729624 | 1.633376635 | 0.707857496 |
|  |  |  |  | 457.499 | 228.0270198 | 0.576882546 | 0.361814011 | 2.065188607 | 0.001851758 | 0.075852782 | 1.594417374 | 0.673029336 |
|  |  |  |  | 457.499 | 371.0368044 | 0.012363166 | 0.008511088 | 1.461770295 | 0.048340602 | 0.232912898 | 1.452595325 | 0.538632842 |
|  |  |  |  | 457.589 | 268.018949 | 0.065767008 | 0.04125362 | 1.759086257 | 0.013769513 | 0.167725387 | 1.594211794 | 0.672843306 |
|  |  |  |  | 457.728 | 210.0163778 | 0.118646413 | 0.081831195 | 1.959408089 | 0.004655411 | 0.108849076 | 1.449892217 | 0.535945657 |
|  |  |  |  | 458.473 | 131.0452944 | 0.048390995 | 0.033773452 | 1.670107765 | 0.005414187 | 0.114144225 | 1.432811643 | 0.518848966 |
|  |  |  |  | 459.021 | 340.1006796 | 0.063855661 | 0.049296565 | 1.707524068 | 0.008727434 | 0.141875675 | 1.29533693 | 0.373327406 |
|  |  |  |  | 459.0395 | 256.0924824 | 0.099562151 | 0.077860867 | 1.602698263 | 0.028574025 | 0.205390155 | 1.278718752 | 0.354698985 |
|  |  |  |  | 459.0395 | 322.0698387 | 3.05637958 | 2.367713715 | 1.757978494 | 0.009910722 | 0.1493824 | 1.290856898 | 0.368329075 |
|  |  |  |  | 459.0395 | 324.0663427 | 0.11578944 | 0.092818866 | 1.561806388 | 0.024831008 | 0.198541116 | 1.247477424 | 0.319013707 |
|  |  |  |  | 459.058 | 155.0816009 | 0.08782998 | 0.071148866 | 1.550642849 | 0.029447241 | 0.206793505 | 1.234453684 | 0.303872708 |
|  |  |  |  | 459.0975 | 323.0746722 | 0.32543107 | 0.254652384 | 1.637763378 | 0.01706344 | 0.178598395 | 1.277942366 | 0.353822774 |
|  |  |  |  | 459.0975 | 377.0530245 | 0.010748657 | 0.014223236 | 1.51871298 | 0.043904693 | 0.226687431 | 0.755711055 | -0.404093367 |
|  |  |  |  | 459.1395 | 339.0971761 | 0.635813059 | 0.485085619 | 1.713026266 | 0.01081654 | 0.154429254 | 1.310723375 | 0.39036324 |
|  |  |  |  | 459.142 | 193.0282207 | 0.098416077 | 0.077115491 | 1.597356064 | 0.019916402 | 0.186806485 | 1.276216703 | 0.351873321 |
|  |  |  |  | 459.591 | 206.0561811 | 0.094426044 | 0.065203863 | 1.674329467 | 0.030940984 | 0.209049965 | 1.448166397 | 0.53422738 |
|  |  |  |  | 459.6115 | 258.0374258 | 0.335728015 | 0.181724346 | 2.071202388 | 0.00281954 | 0.08982013 | 1.847457548 | 0.885541213 |
|  |  |  |  | 460.719 | 358.0698901 | 0.014995826 | 0.011943236 | 1.421939502 | 0.031969615 | 0.210506927 | 1.255591562 | 0.328367239 |
|  |  |  |  | 461.5255 | 284.0534894 | 0.069070923 | 0.053327086 | 1.745463369 | 0.018656258 | 0.183112974 | 1.295231525 | 0.373210005 |
|  |  |  |  | 461.835 | 245.1132563 | 0.021057843 | 0.012754246 | 1.936854387 | 0.00083297 | 0.060474901 | 1.651045634 | 0.723379996 |
|  |  |  |  | 461.883 | 264.1268175 | 0.042361945 | 0.024142639 | 2.130353123 | 5.12872E-05 | 0.029537999 | 1.754652654 | 0.811185467 |
|  |  |  |  | 462.117 | 263.1237517 | 0.42954141 | 0.254244712 | 2.14920577 | 7.6686E-05 | 0.032775272 | 1.689480215 | 0.756579455 |
|  |  | SWEP | -3.389320186 | 462.132 | 221.0592308 | 0.03635043 | 0.0472937 | 1.356211868 | 0.036314143 | 0.215922409 | 0.768610409 | -0.37967558 |
|  |  | Diclosulam | 4.167452624 | 462.541 | 407.2285695 | 0.011552161 | 0.006782893 | 1.884239398 | 0.006257468 | 0.121663291 | 1.703131889 | 0.76819016 |
|  |  |  |  | 462.912 | 833.4582202 | 0.013965197 | 0.008653781 | 1.631540356 | 0.029336915 | 0.206619776 | 1.613768334 | 0.690433486 |
|  |  | 13-(3,4-dihydroxyphenyl)-5-(3-hydroxyphenyl)-18-[3,5,7-trihydroxy-2-(3-hydroxyphenyl)-3,4-dihydro-2H-1-benzopyran-4-yl]-4,12,14-trioxapentacyclo[11.7.1.0,.0,.0,]henicosa-2(11),3(8),9,15(20),16,18-hexaene-6,9,17,19,21-pentol | 21.56769245 | 462.915 | 833.7922375 | 0.019677305 | 0.011340815 | 1.815186798 | 0.01724839 | 0.179118873 | 1.735087388 | 0.795008327 |
|  |  |  |  | 463.061 | 834.1284782 | 0.014154836 | 0.007357647 | 1.794619552 | 0.011541955 | 0.158102343 | 1.92382639 | 0.943978613 |
|  |  |  |  | 464.047 | 426.0482092 | 0.095628267 | 0.06794091 | 1.686369879 | 0.014085 | 0.168918627 | 1.40752113 | 0.49315658 |
|  |  | 4-Carboxy-4-hydroxy-2-oxoadipate | -3.026077691 | 464.37 | 221.1402105 | 0.015661399 | 0.025156415 | 1.288792994 | 0.034816143 | 0.214177908 | 0.622560854 | -0.68371323 |
|  |  | O-Phospho-L-serine | -19.04123198 | 465.762 | 186.0762526 | 0.052995621 | 0.027068945 | 1.996839519 | 0.001732801 | 0.074602278 | 1.957801487 | 0.969234489 |
|  |  |  |  | 466.121 | 81.03411544 | 0.031081206 | 0.016426608 | 1.912578803 | 0.046138515 | 0.229929993 | 1.892125656 | 0.920007901 |
|  |  |  |  | 466.544 | 306.0759742 | 0.152905212 | 0.105481697 | 1.546465969 | 0.046168202 | 0.229971567 | 1.449589998 | 0.535644906 |
|  |  |  |  | 467.49 | 225.0269889 | 0.035132675 | 0.022525571 | 1.62316511 | 0.020996753 | 0.189721589 | 1.559679656 | 0.641249743 |
|  |  |  |  | 467.491 | 190.0348593 | 0.013399159 | 0.008224443 | 1.732497354 | 0.022540096 | 0.193533754 | 1.629187325 | 0.704152495 |
|  |  |  |  | 467.492 | 85.02905442 | 0.106420415 | 0.07010951 | 1.653262292 | 0.026284619 | 0.201376123 | 1.517916972 | 0.60209288 |
|  |  |  |  | 467.632 | 112.0508591 | 0.81702218 | 0.625439707 | 2.001151739 | 0.002929078 | 0.091092739 | 1.306316453 | 0.38550443 |
|  |  |  |  | 467.6745 | 272.1718341 | 0.00241442 | 0.008420424 | 1.506603895 | 0.000842952 | 0.060555641 | 0.286733722 | -1.802216511 |
|  |  |  |  | 467.869 | 372.0811041 | 0.066994894 | 0.08686204 | 1.555463398 | 0.03322728 | 0.212190644 | 0.771279302 | -0.374674699 |
|  |  |  |  | 468.074 | 484.1164912 | 0.015399022 | 0.011401089 | 1.426361224 | 0.043805094 | 0.2265374 | 1.350662358 | 0.433667072 |
|  |  |  |  | 468.8115 | 278.1062638 | 0.15255168 | 0.112749984 | 1.497367159 | 0.044664451 | 0.227816201 | 1.353008433 | 0.436170831 |
|  |  |  |  | 469.407 | 729.7317708 | 0.077899334 | 0.05052406 | 1.65286282 | 0.030183702 | 0.207927799 | 1.541826485 | 0.624640416 |
|  |  |  |  | 469.468 | 729.3984911 | 0.066050166 | 0.034363914 | 1.122538037 | 0.034710282 | 0.214050048 | 1.92207925 | 0.942667821 |
|  |  |  |  | 469.7755 | 310.0088025 | 0.026147961 | 0.014798771 | 1.701680567 | 0.040887684 | 0.221917808 | 1.766900894 | 0.821221121 |
|  |  |  |  | 469.803 | 240.0269734 | 0.418944786 | 0.221815736 | 2.137243133 | 0.000745881 | 0.059690248 | 1.888706337 | 0.917398404 |
|  |  |  |  | 470.0525 | 253.0082241 | 0.077100326 | 0.040612641 | 2.08945347 | 0.000800983 | 0.060204219 | 1.898431715 | 0.924808108 |
|  |  |  |  | 470.408 | 212.0322286 | 0.052318944 | 0.027859415 | 2.135734854 | 0.000392013 | 0.053677082 | 1.877962746 | 0.909168444 |
|  |  |  |  | 470.4695 | 258.0373632 | 0.475367233 | 0.260488444 | 2.131768228 | 0.00443708 | 0.10710458 | 1.824907185 | 0.86782309 |
|  |  |  |  | 470.5745 | 213.0161189 | 0.486397074 | 0.284285633 | 2.120278993 | 0.006655076 | 0.125435788 | 1.710944968 | 0.774793357 |
|  |  |  |  | 470.667 | 340.0814393 | 0.081271995 | 0.059891016 | 1.489354311 | 0.039137129 | 0.219010173 | 1.356998106 | 0.440418708 |
|  |  |  |  | 470.727 | 257.0056055 | 0.026975931 | 0.011019197 | 1.782420645 | 0.0009888 | 0.062528338 | 2.448085021 | 1.291653663 |
|  |  |  |  | 470.7735 | 96.04494631 | 0.235272698 | 0.161783888 | 2.120670519 | 0.000547599 | 0.057123361 | 1.454240599 | 0.540265978 |
|  |  |  |  | 471.0115 | 377.0606481 | 0.045347863 | 0.037972702 | 1.467138839 | 0.044396585 | 0.227421368 | 1.194222694 | 0.25607189 |
|  |  |  |  | 473.235 | 257.953747 | 0.243309405 | 0.139485909 | 2.089868766 | 0.001633846 | 0.073460478 | 1.744329639 | 0.802672703 |
|  |  |  |  | 473.25 | 241.0109982 | 0.025890539 | 0.0160926 | 1.90642645 | 0.003984259 | 0.103100279 | 1.608847415 | 0.686027506 |
|  |  |  |  | 473.3895 | 137.0460024 | 0.230850391 | 0.156622919 | 1.412094178 | 0.046229715 | 0.230057588 | 1.473924712 | 0.559662834 |
|  |  |  |  | 473.454 | 252.9986939 | 0.027832144 | 0.018271795 | 1.796473025 | 0.008722402 | 0.141841234 | 1.523229904 | 0.607133707 |
|  |  |  |  | 473.515 | 220.008563 | 0.015301715 | 0.007776445 | 1.586278738 | 0.046550571 | 0.230503625 | 1.96770059 | 0.976510713 |
|  |  |  |  | 473.553 | 237.0160455 | 0.097573787 | 0.052544235 | 1.698885659 | 0.035800569 | 0.215337621 | 1.856983691 | 0.892961145 |
|  |  |  |  | 474.078 | 313.0298045 | 0.03885777 | 0.02294127 | 1.832746491 | 0.023292947 | 0.195258954 | 1.69379338 | 0.760257896 |
|  |  |  |  | 474.481 | 276.0478992 | 0.135427466 | 0.077772901 | 2.075333345 | 0.000603921 | 0.057998344 | 1.741319466 | 0.800180907 |
|  |  |  |  | 474.481 | 319.042551 | 0.003775593 | 0.00500784 | 1.720604639 | 0.013653898 | 0.167278681 | 0.753936413 | -0.407485243 |
|  |  |  |  | 476.103 | 224.085929 | 0.045976497 | 0.034212912 | 1.65636904 | 0.010172718 | 0.150899042 | 1.343834633 | 0.426355617 |
|  |  |  |  | 476.185 | 109.0288589 | 0.268090913 | 0.140781566 | 2.013386749 | 0.006628505 | 0.125190887 | 1.904304103 | 0.929263885 |
|  |  |  |  | 476.234 | 206.0774807 | 0.058599032 | 0.039076513 | 1.844606966 | 0.005163049 | 0.112510799 | 1.499597242 | 0.584575078 |
|  |  |  |  | 476.2545 | 312.0570589 | 0.021570273 | 0.014252915 | 1.840842037 | 0.02526565 | 0.199414404 | 1.513393799 | 0.597787439 |
|  |  |  |  | 476.3405 | 321.059002 | 0.050170055 | 0.036885427 | 1.732701459 | 0.004386206 | 0.106681726 | 1.360159256 | 0.443775581 |
|  |  |  |  | 476.5725 | 244.0300507 | 0.038769127 | 0.020516535 | 1.998575615 | 0.004719648 | 0.109341687 | 1.889652791 | 0.918121175 |
|  |  |  |  | 477.202 | 352.0656469 | 0.052305228 | 0.034127791 | 1.871545109 | 0.033802142 | 0.212926893 | 1.532628583 | 0.616008117 |
|  |  |  |  | 478.7545 | 155.0217621 | 0.133746513 | 0.095152554 | 1.795268174 | 0.01804864 | 0.181279939 | 1.405600872 | 0.491186991 |
|  |  |  |  | 479.9355 | 354.0332253 | 0.00724305 | 0.004910345 | 1.905978197 | 0.005174102 | 0.112585 | 1.475059277 | 0.560772932 |
|  |  |  |  | 481.549 | 140.0567389 | 0.005214327 | 0.0351891 | 1.87891558 | 0.034980923 | 0.214375689 | 0.148180185 | -2.754575557 |
|  |  |  |  | 483.364 | 260.0529292 | 0.083468201 | 0.041426768 | 2.071529763 | 0.004046544 | 0.103684936 | 2.01483737 | 1.010663395 |
|  |  |  |  | 483.415 | 324.071048 | 0.035372991 | 0.022447094 | 2.123657906 | 0.000791228 | 0.060117827 | 1.575838272 | 0.656119479 |
|  |  |  |  | 483.485 | 316.1758417 | 0.013963287 | 0.00541058 | 2.111160388 | 9.82778E-05 | 0.034450301 | 2.580737636 | 1.367783481 |
|  |  | CE(20:3(5Z,8Z,11Z)) | -1.403013646 | 483.502 | 676.1273294 | 0.000288206 | 0.001413488 | 1.821151295 | 0.0019946 | 0.077993578 | 0.203896922 | -2.294088098 |
|  |  |  |  | 484.425 | 335.063731 | 0.013420693 | 0.007244713 | 1.948262989 | 0.001036752 | 0.063637814 | 1.852480922 | 0.889458685 |
|  |  |  |  | 484.558 | 380.0600091 | 0.028484079 | 0.041072339 | 1.484289204 | 0.031108659 | 0.209292627 | 0.693510037 | -0.528011332 |
|  |  |  |  | 484.565 | 555.1063984 | 0.021943373 | 0.017076118 | 1.516019722 | 0.029568659 | 0.206983535 | 1.285032888 | 0.361805283 |
|  |  |  |  | 484.644 | 298.9929476 | 0.036901117 | 0.020865304 | 1.999499335 | 0.008764898 | 0.142131372 | 1.768539683 | 0.822558591 |
|  |  |  |  | 485.3165 | 503.0567429 | 0.029789934 | 0.009056243 | 2.125660354 | 0.008779005 | 0.142227328 | 3.289436427 | 1.717840431 |
|  |  |  |  | 485.385 | 81.0341085 | 0.025514984 | 0.01250298 | 2.083703883 | 0.000751857 | 0.059749178 | 2.04071226 | 1.029072777 |
|  |  |  |  | 485.39 | 305.0008834 | 0.047041778 | 0.025980353 | 1.734303023 | 0.04440873 | 0.227439343 | 1.810667398 | 0.856521562 |
|  |  |  |  | 485.4525 | 127.0391087 | 1.771234662 | 0.933053299 | 2.077585562 | 0.001664798 | 0.073828357 | 1.898320991 | 0.924723961 |
|  |  |  |  | 485.4525 | 279.0672112 | 0.020187627 | 0.009476665 | 2.109132835 | 0.001205845 | 0.067079478 | 2.130245879 | 1.09101996 |
|  |  |  |  | 485.462 | 85.0290562 | 0.087109624 | 0.038749354 | 1.933033484 | 0.023318163 | 0.195315326 | 2.248027751 | 1.168659845 |
|  |  |  |  | 485.462 | 208.0088314 | 0.044789846 | 0.02142212 | 2.136335514 | 0.005459807 | 0.114429599 | 2.090822347 | 1.064070484 |
|  |  |  |  | 485.462 | 521.0666694 | 0.094866597 | 0.030405685 | 2.101828541 | 0.007045396 | 0.128921182 | 3.120028304 | 1.641559117 |
|  |  |  |  | 485.4675 | 543.0494091 | 0.037584492 | 0.01391703 | 2.136714155 | 0.004641586 | 0.108741861 | 2.700611452 | 1.433286088 |
|  |  |  |  | 485.473 | 140.0109336 | 0.60143887 | 0.305698993 | 2.06551671 | 0.003075784 | 0.093033592 | 1.967421826 | 0.976306313 |
|  |  |  |  | 485.473 | 226.0192143 | 0.077738056 | 0.037843691 | 2.141179663 | 0.000395239 | 0.053770294 | 2.054188028 | 1.038568243 |
|  |  |  |  | 485.4795 | 207.0052802 | 0.676692196 | 0.339624021 | 2.105680021 | 0.001103642 | 0.065081025 | 1.992474483 | 0.994561248 |
|  |  |  |  | 485.4795 | 278.0638625 | 0.314615763 | 0.169228286 | 2.088838179 | 0.001413005 | 0.070507134 | 1.859120428 | 0.894620226 |
|  |  |  |  | 485.531 | 168.0656999 | 0.078507847 | 0.05551524 | 1.893632027 | 0.014333213 | 0.169831827 | 1.414167482 | 0.49995299 |
|  |  |  |  | 486.347 | 625.1591053 | 0.009623525 | 0.006565061 | 1.35527793 | 0.037709275 | 0.217445348 | 1.465869886 | 0.551757052 |
|  |  |  |  | 488.071 | 240.0500039 | 0.014270813 | 0.009523566 | 1.865792884 | 0.009687904 | 0.148053546 | 1.498473733 | 0.583493795 |
|  |  |  |  | 488.098 | 178.0535431 | 0.033674197 | 0.027513456 | 1.67518799 | 0.030371398 | 0.208210011 | 1.223917414 | 0.291506213 |
|  |  |  |  | 488.182 | 230.0662386 | 7.55897E-05 | 0.001143155 | 2.249053644 | 0.008262127 | 0.138590591 | 0.066123749 | -3.918687673 |
|  |  |  |  | 490.161 | 215.0427735 | 0.01333358 | 0.001718184 | 1.81060699 | 0.008878064 | 0.142896101 | 7.76027302 | 2.95610741 |
|  |  |  |  | 493.742 | 584.1333916 | 0.156028039 | 0.110200418 | 1.614769309 | 0.034888751 | 0.214265242 | 1.415857048 | 0.501675611 |
|  |  |  |  | 497.4125 | 259.0214312 | 0.137929667 | 0.081438469 | 2.08032381 | 0.001289792 | 0.068559099 | 1.693667232 | 0.760150445 |
|  |  |  |  | 501.527 | 304.0977919 | 0.395322251 | 0.140844218 | 2.062343647 | 0.003881936 | 0.102114223 | 2.80680498 | 1.488928827 |
|  |  |  |  | 501.9925 | 196.0729262 | 0.053629317 | 0.026970952 | 1.86636039 | 0.024768542 | 0.198413732 | 1.988410257 | 0.991615451 |
|  |  |  |  | 505.64 | 307.0832192 | 0.020294701 | 0.009455823 | 1.566911116 | 0.024501441 | 0.197863602 | 2.146264913 | 1.101828159 |
|  |  |  |  | 506.628 | 582.1640208 | 0.034868319 | 0.026230453 | 1.712567415 | 0.019529934 | 0.185709069 | 1.329306795 | 0.410674107 |
|  |  |  |  | 509.329 | 412.09791 | 0.030392531 | 0.020113502 | 1.643038064 | 0.019977561 | 0.186977419 | 1.511051217 | 0.595552561 |
|  |  |  |  | 511.289 | 395.0901073 | 0.024320111 | 0.016488481 | 1.681080505 | 0.035005687 | 0.214405283 | 1.474975849 | 0.560691332 |
|  |  |  |  | 514.949 | 204.1535271 | 0.137544302 | 0.097185398 | 1.581230284 | 0.011096803 | 0.155884569 | 1.415277452 | 0.501084908 |
|  |  |  |  | 519.678 | 153.0771631 | 0.246218916 | 0.199932939 | 1.678226309 | 0.035849394 | 0.215393798 | 1.231507513 | 0.30042543 |
|  |  |  |  | 521.579 | 391.0874708 | 0.020490671 | 0.015543584 | 1.665784902 | 0.044575062 | 0.227684818 | 1.318271916 | 0.398647981 |
|  |  |  |  | 524.26 | 189.1345127 | 0.13139685 | 0.083042363 | 1.832385503 | 0.017667661 | 0.180269076 | 1.582286992 | 0.662011296 |
|  |  |  |  | 525.384 | 243.6450392 | 0.009215262 | 0.006970298 | 1.666482639 | 0.036094606 | 0.215674078 | 1.322075736 | 0.402804825 |
|  |  | Arsenic acid | -13.88065296 | 526.4995 | 142.9483063 | 0.146176915 | 0.08063741 | 1.790883908 | 0.045200981 | 0.228596985 | 1.812767977 | 0.858194281 |
|  |  | Aplysil | -15.19321406 | 527.953 | 310.2008789 | 0.305490606 | 0.163248625 | 1.55952337 | 0.046618739 | 0.230597817 | 1.871321159 | 0.904057177 |
|  |  |  |  | 528.206 | 258.6626436 | 0.040074494 | 0.02619653 | 1.60568197 | 0.01020884 | 0.15110438 | 1.529763452 | 0.613308585 |
|  |  |  |  | 540.507 | 286.6671498 | 0.012724109 | 0.008096776 | 1.503548257 | 0.037269876 | 0.216975716 | 1.57150325 | 0.652145256 |
|  |  |  |  | 541.17 | 364.2031831 | 0.005343586 | 0.002655202 | 1.615897888 | 0.00941743 | 0.146389989 | 2.012496937 | 1.008986588 |
|  |  |  |  | 541.22 | 279.6864859 | 0.010627918 | 0.014371401 | 1.26411931 | 0.045090263 | 0.22843695 | 0.739518576 | -0.435341709 |
|  |  |  |  | 560.516 | 222.6558668 | 0.006244641 | 0.003163237 | 1.656290877 | 0.045486425 | 0.229007002 | 1.97413011 | 0.981217077 |
|  |  |  |  | 563.51 | 190.9572293 | 0.016624812 | 0.014853131 | 1.659596852 | 0.028105671 | 0.204609861 | 1.11927997 | 0.162570948 |
|  |  |  |  | 572.2095 | 279.6776779 | 0.005177381 | 0.002898852 | 1.700590594 | 0.011716086 | 0.158940124 | 1.786010387 | 0.836740471 |
|  |  |  |  | 580.97 | 188.957992 | 0.025826788 | 0.021847563 | 1.706732594 | 0.013634879 | 0.167204702 | 1.182135859 | 0.24139585 |
|  |  |  |  | 582.805 | 190.9572588 | 0.020207996 | 0.016822094 | 2.065588654 | 0.000566297 | 0.057430107 | 1.201277078 | 0.264568951 |
|  |  | Phosphoagmatine | 8.212439404 | 599.102 | 211.1804026 | 0.02771075 | 0.01884361 | 1.444522854 | 0.048089678 | 0.232583029 | 1.470564793 | 0.556370351 |
|  |  |  |  | 646.046 | 156.0423281 | 0.014486187 | 0.006605226 | 1.607680565 | 0.0363931 | 0.216011127 | 2.193140177 | 1.132998027 |
|  |  | 3-(2-Carboxyethenyl)-cis,cis-mucote | 20.61091015 | 668.561 | 213.1678493 | 0.023382804 | 0.020025857 | 2.080179917 | 0.000285581 | 0.049740848 | 1.167630659 | 0.223583997 |
|  |  |  |  | 698.154 | 190.9573199 | 0.00731693 | 0.006417608 | 1.501770102 | 0.032945001 | 0.211821637 | 1.140133493 | 0.189202753 |
|  |  |  |  | 705.711 | 114.8930128 | 0.04570749 | 0.026906457 | 1.567322758 | 0.025500454 | 0.199876881 | 1.698755468 | 0.764478195 |
|  |  | Thenylchlor | 3.236665881 | 713.453 | 324.8459248 | 0.027317635 | 0.017447565 | 1.731506317 | 0.017161 | 0.178873964 | 1.56569897 | 0.646806859 |
|  |  |  |  | 714.398 | 79.99042345 | 0.042069857 | 0.026619801 | 1.550898957 | 0.04190623 | 0.223581716 | 1.580397157 | 0.660287157 |
|  |  |  |  | 25.402 | 419.2338955 | 0.067817432 | 0.038664847 | 1.409098032 | 0.039155924 | 0.219030945 | 1.753981639 | 0.810633646 |

#MS1 Primary mass spectrometry

MS2 Secondary mass spectrometry

PPM Exact quality number matching

RT Retention time

MZ Mass-to-charge ratio

VIP Variable importance values
