# Supplementary material for: Protein arginine methyltransferase 3 promotes glycolysis and hepatocellular carcinoma growth by enhancing arginine methylation of lactate dehydrogenase A
Source: Clin Transl Med. 2022 Jan 28;12(1):e686. doi: 10.1002/ctm2.686 (PMC8797063; doi:10.1002/ctm2.686)
Supplement: Supplementary file 26 — Table S5 [file CTM2-12-e686-s020.docx]

| **Supplementary Table S5. Differentially metabolites between control and PRMT3-overexpressing Huh7 cells in negative ion mode** | | | | | | | | | | | | |
| --- | --- | --- | --- | --- | --- | --- | --- | --- | --- | --- | --- | --- |
| **MS2 name** | **MS2 score** | **MS1 name** | **MS1 ppm** | **rt** | **mz** | **MEAN PRMT3** | **MEAN control** | **VIP** | **P-VALUE** | **Q-VALUE** | **FOLD CHANGE** | **LOG_FOLDCHANGE** |
|  |  |  |  | 61.3324 | 147.0114869 | 0.303687682 | 0.044526967 | 2.115191073 | 0.021198956 | 0.203877388 | 6.82030923 | 2.769837152 |
|  |  |  |  | 457.006 | 475.0457466 | 0.004171098 | 0.000863496 | 2.192736148 | 0.003090631 | 0.100263912 | 4.830476438 | 2.272165491 |
|  |  |  |  | 62.5125 | 184.0690452 | 0.194718334 | 0.045056713 | 1.962512442 | 0.00728364 | 0.133532001 | 4.321627548 | 2.111574741 |
|  |  |  |  | 62.45545 | 183.0657718 | 2.129062963 | 0.554242453 | 1.94319092 | 0.007561248 | 0.134950232 | 3.841392791 | 1.94162949 |
|  |  |  |  | 62.4677 | 139.0758276 | 0.328567066 | 0.090513324 | 1.905648689 | 0.008036541 | 0.13794762 | 3.630040874 | 1.859985793 |
|  |  | 2-[({2-[7,8,9,12,13,14,17,18,19,25-decahydroxy-24-(hydroxymethyl)-4,22,27-trioxo-3,23,26-trioxahexacyclo[13.10.3.1]nonacosa-5(10),6,8,11,13,15(28),16,18,20-nonaen-29-yl]-3,4,5-trihydroxyphenyl}(hydroxy)methylidene)amino]acetic acid | -10.45937437 | 98.969 | 842.594474 | 1.078349954 | 0.31383505 | 1.703766885 | 0.033250938 | 0.23133754 | 3.436040535 | 1.780747056 |
|  |  |  |  | 479.623 | 512.8234586 | 0.006591445 | 0.002006077 | 1.986540451 | 0.019695138 | 0.199265315 | 3.285739284 | 1.71621801 |
|  |  |  |  | 499.056 | 681.0381074 | 0.001994627 | 0.00064151 | 1.47778119 | 0.006785874 | 0.130817778 | 3.109270169 | 1.63657598 |
|  |  |  |  | 488.6915 | 459.0384827 | 0.003173331 | 0.001078202 | 1.411219267 | 0.005039335 | 0.118592816 | 2.943168349 | 1.557370067 |
| Dimethylmalonic acid | 0.580009692 |  |  | 153.636 | 131.0341037 | 0.103041798 | 0.038831262 | 1.356251944 | 0.016718297 | 0.188558596 | 2.65357837 | 1.407939157 |
| Succinic anhydride | 0.754396154 |  |  | 61.3492 | 99.00786651 | 0.333427138 | 0.126739124 | 1.902045217 | 0.041484854 | 0.24317615 | 2.630814599 | 1.395509581 |
|  |  |  |  | 485.294 | 520.0554619 | 0.026253983 | 0.010139432 | 1.935355574 | 0.00157069 | 0.076379933 | 2.58929533 | 1.372559525 |
|  |  |  |  | 130.952 | 443.3177834 | 0.017411346 | 0.006802559 | 1.863311643 | 0.01514989 | 0.181865495 | 2.559528912 | 1.355878303 |
|  |  |  |  | 60.1203 | 277.1228887 | 0.240136021 | 0.09473734 | 2.217544563 | 0.000334988 | 0.05337624 | 2.534755797 | 1.341846762 |
|  |  |  |  | 78.7222 | 245.0291261 | 0.020407333 | 0.00809746 | 1.702242969 | 0.013158244 | 0.172060037 | 2.520213973 | 1.333546228 |
|  |  |  |  | 449.264 | 280.9723123 | 0.010861211 | 0.004574478 | 1.82468681 | 0.00574992 | 0.124164144 | 2.374305838 | 1.247505783 |
|  |  |  |  | 432.043 | 228.028335 | 0.110921037 | 0.046825909 | 1.28162836 | 0.037855197 | 0.238451258 | 2.36879624 | 1.244154105 |
|  |  |  |  | 276.625 | 322.0535278 | 0.13457586 | 0.057349404 | 1.934237408 | 0.001679552 | 0.07826589 | 2.34659564 | 1.230569262 |
|  |  | Pyronaridine | -6.410637888 | 485.223 | 519.0539556 | 0.195196748 | 0.083497199 | 2.022192148 | 0.000802008 | 0.06379519 | 2.337764036 | 1.225129318 |
|  |  | 7-Methylguanosine 5'-phosphate | -23.51452123 | 44.4159 | 379.2534821 | 0.021987021 | 0.00963317 | 2.192676587 | 9.40189E-06 | 0.019974742 | 2.282428408 | 1.190569608 |
|  |  |  |  | 461.663 | 387.0103874 | 0.017230877 | 0.007625896 | 2.04596808 | 0.047084515 | 0.25405593 | 2.259521635 | 1.176017371 |
|  |  | PC(18:4(6Z,9Z,12Z,15Z)/20:5(5Z,8Z,11Z,14Z,17Z)) | 3.951020767 | 485.201 | 801.0658376 | 0.00560098 | 0.002491367 | 2.098704537 | 0.000367054 | 0.054409434 | 2.248155288 | 1.168741691 |
|  |  | Biochanin A | 0.843197519 | 44.4365 | 285.2710163 | 0.14297807 | 0.064085158 | 1.867751292 | 0.010258322 | 0.154478608 | 2.231063689 | 1.157731698 |
|  |  |  |  | 459.149 | 444.0318603 | 0.048033639 | 0.021790343 | 2.160912987 | 0.001729894 | 0.079085818 | 2.204354413 | 1.140356197 |
|  |  |  |  | 457.197 | 474.0419232 | 0.049263117 | 0.022376551 | 2.190177209 | 0.000694489 | 0.061322518 | 2.201550984 | 1.138520255 |
|  |  |  |  | 359.671 | 500.657997 | 0.018553101 | 0.008603712 | 1.464056001 | 0.035176099 | 0.234485271 | 2.156406647 | 1.108629262 |
|  |  |  |  | 453.197 | 553.0494375 | 0.070347067 | 0.032894721 | 1.723809095 | 0.004946319 | 0.117788574 | 2.138551867 | 1.096634198 |
|  |  |  |  | 59.5351 | 439.3222981 | 0.057873275 | 0.027067547 | 2.080156146 | 0.000321054 | 0.052877415 | 2.138105602 | 1.096333111 |
|  |  |  |  | 359.591 | 501.1602823 | 0.00730464 | 0.003425595 | 1.471077536 | 0.027628459 | 0.220339231 | 2.132371227 | 1.092458621 |
|  |  |  |  | 458.0945 | 489.053976 | 0.020795725 | 0.010080647 | 2.145071015 | 0.001172613 | 0.070998812 | 2.062935538 | 1.044698741 |
|  |  |  |  | 221.913 | 225.0713404 | 0.044053398 | 0.021375001 | 1.073403146 | 0.047057 | 0.254005486 | 2.060977565 | 1.043328801 |
|  |  |  |  | 394.1745 | 331.0725059 | 0.019557114 | 0.009527188 | 1.818961826 | 0.006991616 | 0.13197286 | 2.052768872 | 1.037571198 |
|  |  | GW 3965 | 3.848379893 | 499.777 | 583.0619166 | 0.003295135 | 0.001616402 | 1.093847853 | 0.03951325 | 0.240694191 | 2.038561376 | 1.027551393 |
|  |  |  |  | 485.203 | 357.9937203 | 0.023817409 | 0.011709649 | 1.705871469 | 0.000794475 | 0.063638022 | 2.033998517 | 1.024318627 |
|  |  |  |  | 620.245 | 101.0598282 | 0.354298598 | 0.17435394 | 1.774906596 | 0.009124081 | 0.146060131 | 2.032065333 | 1.022946787 |
|  |  |  |  | 457.7575 | 568.0585402 | 0.011645777 | 0.005731948 | 2.037839247 | 0.002648597 | 0.094387962 | 2.031730929 | 1.022709352 |
|  |  |  |  | 461.411 | 380.9458801 | 0.011213014 | 0.005562531 | 2.014513113 | 0.004422591 | 0.112953123 | 2.01581126 | 1.011360566 |
|  |  | 3,5-Dibromo-L-tyrosine | 12.52310877 | 461.83 | 339.9922217 | 0.010377717 | 0.00517022 | 1.67359632 | 0.026567119 | 0.21789182 | 2.007209784 | 1.005191408 |
|  |  |  |  | 485.2125 | 541.034676 | 0.027612226 | 0.013775815 | 1.586261164 | 0.001347845 | 0.073424301 | 2.004398702 | 1.003169508 |
|  |  |  |  | 451.302 | 581.0430396 | 0.010650185 | 0.005337644 | 1.667119343 | 0.002537859 | 0.092730005 | 1.995296874 | 0.996603416 |
|  |  |  |  | 456.827 | 458.0112344 | 0.003941889 | 0.001991596 | 1.651110177 | 0.028765055 | 0.222814602 | 1.979261025 | 0.984961888 |
|  |  | {2-[4-(4-chloro-1,2-diphenylbut-1-en-1-yl)phenoxy]ethyl}(methyl)amine | -2.748618738 | 454.386 | 392.9461993 | 0.004781016 | 0.002421175 | 1.864135704 | 0.009891219 | 0.151865353 | 1.974668096 | 0.981610184 |
|  |  |  |  | 459.623 | 414.0215217 | 0.053284312 | 0.027132407 | 2.181663942 | 0.000191842 | 0.046764027 | 1.963862293 | 0.973693771 |
|  |  |  |  | 199.109 | 102.0187726 | 0.317720944 | 0.162754358 | 2.020187944 | 0.005436532 | 0.121824486 | 1.95215015 | 0.965064022 |
|  |  |  |  | 214.039 | 498.2640856 | 0.012852574 | 0.006586934 | 1.762610942 | 0.025205174 | 0.214539889 | 1.951222473 | 0.964378279 |
|  |  | 2-Oxoglutaryl-CoA | -9.483254538 | 78.7015 | 896.6157832 | 0.010738467 | 0.005517124 | 1.9802415 | 0.004069263 | 0.110144646 | 1.946388429 | 0.960799649 |
|  |  |  |  | 194.448 | 183.0017553 | 0.026531623 | 0.013665696 | 1.655304199 | 0.010113561 | 0.153460186 | 1.941476132 | 0.957153972 |
|  |  |  |  | 230.7155 | 156.9115505 | 0.718963007 | 0.372617111 | 1.859649471 | 0.026958063 | 0.218809326 | 1.929495417 | 0.948223617 |
|  |  | Thiamine(1+) Diphosphate(1-) | -15.87274879 | 109.274 | 425.3065417 | 0.020966129 | 0.010914801 | 1.900945787 | 0.011035128 | 0.159690558 | 1.920889646 | 0.94177464 |
|  |  |  |  | 85.0591 | 150.0635572 | 0.127867484 | 0.066606774 | 1.399282654 | 0.03533474 | 0.234732943 | 1.91973695 | 0.940908641 |
|  |  |  |  | 458.213 | 523.0379777 | 0.01327908 | 0.006923314 | 1.886185148 | 0.003784529 | 0.107624583 | 1.918023749 | 0.939620584 |
|  |  |  |  | 277.6275 | 362.0401109 | 0.005493448 | 0.002874202 | 1.581665081 | 0.002217147 | 0.087595457 | 1.911294789 | 0.93455031 |
|  |  |  |  | 440.217 | 353.9903498 | 0.013308166 | 0.007018933 | 1.857208339 | 0.001464451 | 0.074785596 | 1.896038239 | 0.922988061 |
|  |  |  |  | 456.186 | 198.0168904 | 0.030214126 | 0.015946179 | 1.953114599 | 0.004989799 | 0.118166878 | 1.894756465 | 0.922012429 |
|  |  |  |  | 460.493 | 350.935387 | 0.009225991 | 0.004879591 | 1.85367389 | 0.007272214 | 0.133472685 | 1.890730432 | 0.918943688 |
| Fructose 1,6-bisphosphate | 0.846945308 |  |  | 461.487 | 338.9898423 | 0.233809384 | 0.124735902 | 2.07576299 | 0.002111008 | 0.085785764 | 1.874435344 | 0.906456063 |
| 8-HETE | 0.941599846 |  |  | 48.877 | 319.2288082 | 0.035921332 | 0.019232363 | 1.875145587 | 0.03280395 | 0.230566937 | 1.867754437 | 0.901304789 |
|  |  |  |  | 479.645 | 176.9354926 | 1.078651309 | 0.578680301 | 1.874221472 | 0.004118461 | 0.110555668 | 1.863984842 | 0.898390128 |
|  |  |  |  | 453.988 | 326.9799263 | 0.091236369 | 0.049294963 | 2.162410991 | 0.000408095 | 0.05553889 | 1.850825395 | 0.888168799 |
|  |  |  |  | 423.78 | 412.9857714 | 0.002565435 | 0.001388361 | 1.825654384 | 0.018732999 | 0.196054219 | 1.847815619 | 0.885820807 |
|  |  |  |  | 172.253 | 199.0011605 | 0.06753701 | 0.036606765 | 1.514251237 | 0.020217278 | 0.200920072 | 1.844932494 | 0.883568029 |
| Glucosamine 6-phosphate | 0.967138231 |  |  | 482.426 | 258.0388857 | 0.043258017 | 0.023470619 | 2.083491635 | 0.001172824 | 0.071002055 | 1.84307102 | 0.882111664 |
|  |  |  |  | 466.551 | 504.0541268 | 0.017001924 | 0.009227626 | 2.178934498 | 0.000211851 | 0.047389031 | 1.842502595 | 0.881666652 |
|  |  |  |  | 377.8355 | 862.0050948 | 0.13191419 | 0.071681265 | 1.977849063 | 0.001497596 | 0.075141988 | 1.840288263 | 0.879931768 |
|  |  |  |  | 78.719 | 897.6186705 | 0.004490481 | 0.002443681 | 1.620229926 | 0.025604065 | 0.21554758 | 1.837588955 | 0.87781409 |
|  |  |  |  | 63.9871 | 177.0915133 | 0.094283188 | 0.051476767 | 1.5342572 | 0.022983362 | 0.208791224 | 1.831567792 | 0.873079101 |
|  |  |  |  | 61.4652 | 672.4996999 | 0.001773187 | 0.000969599 | 1.069455351 | 0.041365139 | 0.243030796 | 1.828784053 | 0.870884728 |
|  |  | Potassium bromate | -8.539062135 | 453.197 | 168.0063506 | 0.018317741 | 0.010035884 | 1.779953851 | 0.006709728 | 0.130377698 | 1.825224504 | 0.868073927 |
|  |  |  |  | 693.2135 | 135.9444458 | 3.93603661 | 2.156928437 | 1.917838296 | 0.019715408 | 0.199330667 | 1.824834122 | 0.867765328 |
|  |  |  |  | 497.515 | 276.0215563 | 0.019498537 | 0.010688772 | 2.116234338 | 0.000739507 | 0.06242114 | 1.824207451 | 0.867269804 |
|  |  | 2,3,4,5-Tetrachloro-4'-biphenylol | 17.6393945 | 423.72 | 309.0001093 | 0.007137512 | 0.003950102 | 1.959001098 | 0.001834928 | 0.080700364 | 1.806918596 | 0.853531512 |
|  |  |  |  | 469.8745 | 230.0151902 | 0.051175729 | 0.028330896 | 2.111445276 | 0.001040256 | 0.068775375 | 1.806357558 | 0.853083494 |
|  |  |  |  | 466.4615 | 429.0209034 | 0.085717296 | 0.047547054 | 1.987866733 | 0.012306283 | 0.167394418 | 1.802788798 | 0.850230391 |
|  |  |  |  | 257.965 | 112.0144756 | 0.083086341 | 0.04628696 | 1.591081539 | 0.014261132 | 0.177675891 | 1.795026967 | 0.844005518 |
|  |  |  |  | 65.1128 | 399.1487403 | 0.011179438 | 0.006231811 | 1.520481505 | 0.029610008 | 0.224564587 | 1.793930855 | 0.843124284 |
|  |  |  |  | 466.441 | 459.0309626 | 0.051110617 | 0.028605301 | 1.993678566 | 0.00222957 | 0.087800726 | 1.786753302 | 0.837340455 |
|  |  | 2-[({2-[7,8,9,12,13,14,17,18,19,25-decahydroxy-24-(hydroxymethyl)-4,22,27-trioxo-3,23,26-trioxahexacyclo[13.10.3.1]nonacosa-5(10),6,8,11,13,15(28),16,18,20-nonaen-29-yl]-3,4,5-trihydroxyphenyl}(hydroxy)methylidene)amino]acetic acid | -8.198152093 | 134.271 | 842.5963771 | 0.360088914 | 0.201603432 | 1.515181539 | 0.006089689 | 0.126515893 | 1.786124917 | 0.836832982 |
|  |  |  |  | 467.707 | 534.0639342 | 0.029076426 | 0.016306026 | 2.046124225 | 0.001494085 | 0.075104835 | 1.783170615 | 0.834444748 |
|  |  |  |  | 421.0855 | 207.0176442 | 0.014538134 | 0.008191829 | 2.181971357 | 0.000621889 | 0.060158517 | 1.774711536 | 0.827584546 |
|  |  |  |  | 256.103 | 242.078813 | 0.055897992 | 0.03158943 | 1.663563418 | 0.03134931 | 0.227946034 | 1.769515684 | 0.823354549 |
|  |  |  |  | 401.978 | 305.023951 | 0.336081956 | 0.192175521 | 1.555238595 | 0.04133622 | 0.242995583 | 1.748828128 | 0.80638851 |
|  |  |  |  | 485.236 | 261.026982 | 0.086506848 | 0.049671573 | 2.025438903 | 0.001282944 | 0.072584872 | 1.741576569 | 0.800393903 |
|  |  |  |  | 40.6802 | 383.2956754 | 0.020933552 | 0.012023554 | 1.663170833 | 0.045094539 | 0.250362098 | 1.741045226 | 0.799953679 |
|  |  |  |  | 226.665 | 134.0463592 | 14.73322325 | 8.513556676 | 1.627533249 | 0.020584382 | 0.202048779 | 1.730560306 | 0.791239217 |
|  |  |  |  | 485.223 | 260.0260827 | 0.326163045 | 0.189406263 | 2.117342511 | 0.000440433 | 0.056306416 | 1.722028827 | 0.784109294 |
|  |  |  |  | 244.759 | 295.0346993 | 0.116442704 | 0.067926462 | 1.653182158 | 0.011622531 | 0.16336932 | 1.714246555 | 0.777574623 |
|  |  |  |  | 448.937 | 279.9713946 | 0.033799035 | 0.019769715 | 1.596157488 | 0.028449053 | 0.222140775 | 1.709636893 | 0.773689946 |
|  |  |  |  | 61.8418 | 187.1336745 | 0.039525519 | 0.023139053 | 1.810565907 | 0.011082312 | 0.159994061 | 1.708173541 | 0.772454552 |
|  |  |  |  | 101.0135 | 340.2862562 | 0.001834717 | 0.001078757 | 1.653821831 | 0.017119366 | 0.190142168 | 1.700768632 | 0.766186894 |
| Glucose 6-phosphate | 0.966457462 |  |  | 485.857 | 259.0229719 | 4.737822243 | 2.793160708 | 2.118273712 | 0.000311073 | 0.05249893 | 1.696222573 | 0.762325488 |
| Fexofenadine | 0.632298231 |  |  | 212.15 | 500.2793916 | 0.100890983 | 0.059761624 | 1.813467556 | 0.019635247 | 0.19907168 | 1.688223572 | 0.755505973 |
|  |  | Basellasaponin C | -23.34005731 | 377.839 | 986.013886 | 0.085145678 | 0.050659699 | 1.652417756 | 0.020192175 | 0.200841859 | 1.680737937 | 0.749094795 |
|  |  |  |  | 454.327 | 537.0176765 | 0.007715309 | 0.004592821 | 1.65028975 | 0.005861705 | 0.124958069 | 1.67986272 | 0.748343339 |
|  |  |  |  | 461.799 | 261.1098851 | 0.016544862 | 0.009895333 | 1.692491616 | 0.005805958 | 0.124564696 | 1.671986365 | 0.741563082 |
|  |  |  |  | 511.836 | 420.9956446 | 0.01140739 | 0.006833969 | 1.693364475 | 0.021846983 | 0.205727174 | 1.669218909 | 0.739173169 |
|  |  |  |  | 485.22 | 223.0014436 | 0.008295617 | 0.004973728 | 2.018302074 | 0.001273646 | 0.072459291 | 1.667887275 | 0.738021787 |
|  |  |  |  | 377.84 | 961.0489921 | 0.337476032 | 0.202734547 | 1.570473609 | 0.029401754 | 0.224140102 | 1.664620248 | 0.735193091 |
|  |  |  |  | 459.932 | 274.0339905 | 0.219274984 | 0.13182579 | 2.109196876 | 0.001615931 | 0.077183289 | 1.663369388 | 0.734108586 |
|  |  |  |  | 455.0795 | 230.0152186 | 0.145302226 | 0.087470974 | 2.060276488 | 0.009423297 | 0.148382338 | 1.661147912 | 0.73218054 |
|  |  |  |  | 51.5462 | 175.0611917 | 0.012760997 | 0.007708336 | 1.345940095 | 0.046491118 | 0.252960807 | 1.655480025 | 0.727249604 |
|  |  |  |  | 455.446 | 228.0283058 | 0.376347138 | 0.228143967 | 2.050687955 | 0.001321733 | 0.073094182 | 1.649603726 | 0.722119497 |
|  |  |  |  | 427.6615 | 242.0071346 | 0.049497439 | 0.03006222 | 1.713807054 | 0.040865736 | 0.242417171 | 1.646499803 | 0.719402339 |
| Cytidine monophosphate | 0.950190231 |  |  | 469.273 | 322.0455483 | 0.315924878 | 0.192088178 | 1.554763682 | 0.016481341 | 0.187599781 | 1.644686736 | 0.71781282 |
|  |  |  |  | 377.8435 | 850.0263025 | 0.078886771 | 0.048066807 | 1.645707209 | 0.020012805 | 0.200279099 | 1.641190177 | 0.714742424 |
| 12-Hydroxydodecanoic acid | 0.950430692 |  |  | 100.098 | 215.1653708 | 0.03093553 | 0.018950945 | 1.686168234 | 0.027355532 | 0.219722874 | 1.632400358 | 0.706994932 |
|  |  |  |  | 470.286 | 290.9684306 | 0.050644669 | 0.031144952 | 1.628234207 | 0.030539624 | 0.226407283 | 1.626095559 | 0.701412041 |
|  |  |  |  | 451.439 | 345.0106456 | 0.012150985 | 0.007522664 | 1.42530542 | 0.036554608 | 0.236581978 | 1.615250402 | 0.691757834 |
|  |  | Butefine hydrochloride | 7.880988432 | 487.565 | 354.9381659 | 0.038871287 | 0.024156844 | 2.031055601 | 0.005585162 | 0.122955727 | 1.609121045 | 0.686272856 |
|  |  |  |  | 78.7291 | 112.0224669 | 0.701697323 | 0.43703574 | 1.824541343 | 0.014543498 | 0.179040929 | 1.605583385 | 0.683097592 |
|  |  |  |  | 377.84 | 879.0453325 | 0.647918425 | 0.40355959 | 1.595122859 | 0.029228217 | 0.223783008 | 1.60550868 | 0.683030465 |
|  |  |  |  | 456.827 | 341.9911105 | 0.049272807 | 0.030806778 | 2.093432735 | 0.000455763 | 0.056638672 | 1.599414485 | 0.677543859 |
|  |  |  |  | 424.2585 | 226.9964986 | 0.986055237 | 0.616990417 | 2.116641478 | 0.00690695 | 0.13150341 | 1.598169453 | 0.676420385 |
|  |  |  |  | 377.852 | 657.9993418 | 0.164899435 | 0.10326248 | 1.393724264 | 0.021680638 | 0.205259746 | 1.596895941 | 0.675270305 |
|  |  |  |  | 416.19 | 154.01162 | 0.023906392 | 0.01498917 | 1.575519428 | 0.039417359 | 0.240568509 | 1.594910969 | 0.673475892 |
|  |  |  |  | 221.168 | 260.044572 | 0.025006674 | 0.015693195 | 1.372321515 | 0.045348503 | 0.250842516 | 1.59347248 | 0.672174103 |
|  |  |  |  | 456.827 | 384.0115151 | 0.060209005 | 0.037801233 | 2.059193285 | 0.000407299 | 0.055518738 | 1.592778853 | 0.671545972 |
|  |  |  |  | 451.344 | 464.0203749 | 0.034137927 | 0.021498108 | 1.685677227 | 0.008015583 | 0.137819662 | 1.587950293 | 0.667165753 |
|  |  |  |  | 443.5215 | 412.0055537 | 0.005692334 | 0.003588302 | 2.043747912 | 0.002416087 | 0.090807202 | 1.586358682 | 0.665719007 |
|  |  |  |  | 424.104 | 227.9996068 | 0.046563545 | 0.029393699 | 2.081645858 | 0.000848743 | 0.064723413 | 1.584133591 | 0.663694004 |
|  |  |  |  | 451.37 | 421.9996667 | 0.019109958 | 0.012065065 | 1.406274101 | 0.041073453 | 0.242673831 | 1.583908374 | 0.66348888 |
|  |  | (25S)-Spirostane-3b,5b,6a-triol 3-[4'-rhamnosylglucoside] | -11.17830886 | 342.764 | 757.9159155 | 0.014245422 | 0.008995562 | 1.60436193 | 0.017129675 | 0.190182236 | 1.583605597 | 0.663213072 |
|  |  |  |  | 479.75 | 258.002219 | 0.04597406 | 0.029047768 | 1.949110118 | 0.002594918 | 0.093594621 | 1.582705415 | 0.662392756 |
|  |  |  |  | 402.0915 | 181.0092185 | 0.235746221 | 0.148955187 | 1.846963291 | 0.04257816 | 0.245402159 | 1.582665399 | 0.662356279 |
|  |  |  |  | 469.324 | 229.0464684 | 0.386463405 | 0.244530613 | 2.234884203 | 1.36643E-05 | 0.019974742 | 1.580429547 | 0.660316724 |
|  |  |  |  | 451.492 | 463.0162401 | 0.273042542 | 0.173669616 | 1.662871539 | 0.007273742 | 0.133480624 | 1.572195233 | 0.652780381 |
|  |  |  |  | 288.629 | 283.0787422 | 0.288341835 | 0.183410196 | 1.304058843 | 0.045364386 | 0.250872444 | 1.572114535 | 0.652706328 |
|  |  |  |  | 459.95 | 493.028163 | 0.020723384 | 0.01318195 | 1.791295898 | 0.005367749 | 0.121287155 | 1.572103126 | 0.652695858 |
| D-Ribose 5-phosphate | 0.980249462 |  |  | 454.653 | 229.0120535 | 2.512267134 | 1.604712103 | 2.150869065 | 0.000505673 | 0.057791789 | 1.565556294 | 0.646675386 |
|  |  | PG(16:0/22:5(4Z,7Z,10Z,13Z,16Z)) | -16.16284511 | 377.841 | 798.044294 | 0.303037591 | 0.194225664 | 1.610559818 | 0.02761291 | 0.220304352 | 1.560234545 | 0.641762921 |
|  |  | PE-NMe(11M3/9D3) | -2.833377522 | 377.841 | 797.0410211 | 1.328846579 | 0.852882048 | 1.541870542 | 0.033315554 | 0.231447645 | 1.558066069 | 0.639756411 |
|  |  |  |  | 454.58 | 352.9682097 | 0.014090436 | 0.009059815 | 2.192397526 | 9.11866E-05 | 0.040564859 | 1.555267543 | 0.63716278 |
|  |  |  |  | 460.37 | 230.0310177 | 0.050260978 | 0.032375793 | 1.912191651 | 0.023560669 | 0.210266461 | 1.552424588 | 0.634523189 |
|  |  |  |  | 155.379 | 881.6098504 | 0.017300733 | 0.011160909 | 1.761069151 | 0.015959517 | 0.18542465 | 1.550118558 | 0.632378562 |
|  |  |  |  | 471.186 | 428.9851498 | 0.022243878 | 0.014355584 | 1.718019753 | 0.023906877 | 0.21112649 | 1.549493064 | 0.631796297 |
|  |  |  |  | 476.7905 | 282.953541 | 0.030657266 | 0.019834615 | 1.818795092 | 0.005388595 | 0.121450948 | 1.545644596 | 0.628208626 |
|  |  | Hydrocortisone cypiote | 4.115114325 | 362.144 | 487.6494792 | 0.002218084 | 0.00143579 | 1.484505902 | 0.03083362 | 0.226972921 | 1.544852051 | 0.627468679 |
|  |  |  |  | 31.2093 | 405.2658027 | 0.241815644 | 0.156805806 | 1.580537123 | 0.02134699 | 0.20430686 | 1.5421345 | 0.624928598 |
|  |  |  |  | 479.696 | 236.9575516 | 0.058096554 | 0.037729933 | 1.606518418 | 0.026105797 | 0.216784146 | 1.539800072 | 0.622743043 |
|  |  |  |  | 377.8415 | 780.0038102 | 0.165591012 | 0.108124377 | 1.703633191 | 0.013475249 | 0.173705236 | 1.531486388 | 0.614932544 |
|  |  |  |  | 46.24925 | 986.7821093 | 0.062438035 | 0.040776904 | 1.295708478 | 0.044974743 | 0.250134245 | 1.531210779 | 0.614672891 |
| 5-Thymidylic acid | 0.836363077 |  |  | 423.7175 | 321.0505008 | 0.058464811 | 0.038213029 | 1.816048975 | 0.006233065 | 0.127455633 | 1.529970601 | 0.613503931 |
|  |  |  |  | 78.96075 | 111.0189459 | 15.21684684 | 9.953905252 | 1.791612005 | 0.017891281 | 0.193058123 | 1.528731332 | 0.612334882 |
|  |  | Mitobronitol | 19.04505684 | 460.763 | 308.9782418 | 0.138882549 | 0.091042698 | 1.830031006 | 0.01304806 | 0.171477054 | 1.525466097 | 0.609250117 |
| 6-Phosphogluconic acid | 0.538376077 |  |  | 497.67 | 275.0178915 | 0.345804753 | 0.226848661 | 1.985101203 | 0.003174727 | 0.101264484 | 1.524385247 | 0.608227551 |
|  |  |  |  | 355.945 | 958.0129267 | 0.001699478 | 0.001115516 | 1.589000134 | 0.020467107 | 0.201691235 | 1.523491298 | 0.607381259 |
|  |  | Soyacerebroside I | 12.29209858 | 377.8415 | 715.0408535 | 3.824821865 | 2.510993977 | 1.547535276 | 0.032033956 | 0.22920177 | 1.523230203 | 0.60713399 |
|  |  |  |  | 487.965 | 256.9709774 | 0.172011523 | 0.112987144 | 1.864193291 | 0.018211247 | 0.194218703 | 1.522399063 | 0.606346579 |
|  |  | Hexaflurate | -24.2203118 | 469.375 | 229.0120541 | 0.903575527 | 0.593920164 | 2.061498476 | 0.002299593 | 0.088933247 | 1.5213754 | 0.605376183 |
|  |  | Phaseolic acid | 22.93269267 | 398.053 | 297.2436699 | 0.065194712 | 0.042877761 | 1.263057809 | 0.032198481 | 0.229497573 | 1.520478448 | 0.604525367 |
|  |  |  |  | 190.786 | 75.00775013 | 0.617939211 | 0.408555097 | 1.590150695 | 0.012168745 | 0.166605782 | 1.512499088 | 0.596934272 |
|  |  |  |  | 141.185 | 816.5800606 | 0.829358164 | 0.549923867 | 1.408842793 | 0.018948798 | 0.196793519 | 1.508132695 | 0.592763372 |
|  |  |  |  | 485.226 | 199.0009021 | 0.20267431 | 0.134447686 | 2.105334643 | 0.000584686 | 0.059471416 | 1.507458526 | 0.59211831 |
|  |  |  |  | 190.023 | 84.0081554 | 0.176648144 | 0.117247812 | 1.967269211 | 0.007919063 | 0.13722478 | 1.506622098 | 0.591317595 |
|  |  |  |  | 444.5125 | 218.9463803 | 0.105212498 | 0.069839725 | 1.898578321 | 0.007157825 | 0.132871395 | 1.506484997 | 0.591186306 |
|  |  |  |  | 361.067 | 904.4675957 | 0.006359684 | 0.00422328 | 1.449419762 | 0.020340317 | 0.201301491 | 1.505863836 | 0.590591324 |
|  |  |  |  | 459.868 | 214.9963159 | 0.042372821 | 0.028207814 | 2.152306843 | 0.000687128 | 0.061213826 | 1.502166077 | 0.587044324 |
|  |  |  |  | 201.534 | 375.1793166 | 0.013364015 | 0.008899286 | 1.874678286 | 0.003786199 | 0.107640102 | 1.501695106 | 0.586591927 |
|  |  |  |  | 455.495 | 319.0076398 | 0.02771138 | 0.018454126 | 1.874934406 | 0.003755063 | 0.107349138 | 1.501635953 | 0.586535098 |
|  |  |  |  | 78.8356 | 193.022469 | 0.039789828 | 0.026547347 | 1.931685344 | 0.007265107 | 0.133435722 | 1.498825037 | 0.583831982 |
|  |  |  |  | 438.1715 | 256.0228254 | 0.080570475 | 0.053914527 | 1.784580415 | 0.006590142 | 0.129672265 | 1.494411241 | 0.579577212 |
|  |  |  |  | 477.804 | 182.9697185 | 0.012541828 | 0.008403838 | 1.680611519 | 0.00762372 | 0.13534529 | 1.492392868 | 0.577627371 |
|  |  |  |  | 449.654 | 236.947637 | 0.123720714 | 0.082954726 | 1.698337757 | 0.003745229 | 0.10725657 | 1.49142454 | 0.576690985 |
|  |  |  |  | 483.967 | 356.9906189 | 0.409994018 | 0.274933373 | 1.967193723 | 0.001130833 | 0.070338508 | 1.491248636 | 0.576520819 |
|  |  |  |  | 196.309 | 365.0160304 | 0.056924854 | 0.038185922 | 1.446211363 | 0.044643761 | 0.249500545 | 1.490728805 | 0.576017825 |
|  |  |  |  | 361.0515 | 903.9685856 | 0.010577087 | 0.007100593 | 2.008491936 | 0.000251204 | 0.049768185 | 1.489606173 | 0.574930957 |
|  |  |  |  | 190.713 | 183.0173942 | 0.037620936 | 0.025277554 | 1.628868636 | 0.007504443 | 0.134652629 | 1.488313935 | 0.573678871 |
|  |  |  |  | 471.224 | 430.0169382 | 0.051862496 | 0.034932082 | 1.989428817 | 0.001501202 | 0.075180022 | 1.484666607 | 0.570138999 |
|  |  |  |  | 201.2145 | 410.1532388 | 0.017401465 | 0.011729061 | 1.700865495 | 0.011923558 | 0.165174021 | 1.483619692 | 0.569121322 |
|  |  |  |  | 377.84 | 985.012474 | 0.344395516 | 0.232351505 | 1.624947747 | 0.012926204 | 0.170825452 | 1.48221771 | 0.567757368 |
|  |  |  |  | 377.841 | 716.042883 | 0.774859539 | 0.523517048 | 1.490705726 | 0.040201275 | 0.241582108 | 1.480103737 | 0.565698294 |
|  |  |  |  | 222.063 | 305.0385263 | 0.031903641 | 0.021598359 | 1.201912089 | 0.045523554 | 0.251171597 | 1.477132686 | 0.562799424 |
|  |  |  |  | 449.011 | 278.9683128 | 0.790206778 | 0.535714589 | 2.022833393 | 0.001782531 | 0.079910563 | 1.475051818 | 0.560765636 |
|  |  |  |  | 211.9135 | 524.2794213 | 0.025640133 | 0.017395425 | 1.727712023 | 0.043254015 | 0.246771075 | 1.473958418 | 0.559695825 |
|  |  |  |  | 258.8605 | 494.252935 | 0.014934822 | 0.010153889 | 1.904122512 | 0.007998062 | 0.137712365 | 1.470847404 | 0.556647579 |
|  |  |  |  | 31.20635 | 206.1631923 | 0.428382649 | 0.291811351 | 1.839784986 | 0.004718961 | 0.115739814 | 1.468012287 | 0.553864044 |
|  |  |  |  | 31.9963 | 205.1595367 | 3.055874258 | 2.081889908 | 1.865241538 | 0.003892521 | 0.108609846 | 1.467836625 | 0.553691401 |
|  |  |  |  | 476.8175 | 96.96853484 | 23.0043419 | 15.68493145 | 1.361493922 | 0.036378834 | 0.236321442 | 1.466652372 | 0.552526961 |
|  |  |  |  | 332.695 | 157.9703356 | 0.043301993 | 0.029598076 | 1.63989148 | 0.019797311 | 0.199593804 | 1.463000289 | 0.548930054 |
|  |  |  |  | 173.133 | 199.0336456 | 0.029372957 | 0.020085603 | 2.092321443 | 0.007466365 | 0.13446279 | 1.462388583 | 0.548326713 |
|  |  |  |  | 440.904 | 369.985323 | 0.039672895 | 0.027152327 | 2.193518003 | 6.1602E-05 | 0.036200136 | 1.461123202 | 0.547077832 |
|  |  |  |  | 222.068 | 424.1548486 | 0.012239078 | 0.008398591 | 1.777507979 | 0.021699249 | 0.205312294 | 1.457277525 | 0.543275652 |
|  |  |  |  | 451.37 | 324.0327198 | 0.284103624 | 0.195072573 | 1.416949082 | 0.036665659 | 0.236745587 | 1.456399629 | 0.542406278 |
|  |  |  |  | 334.563 | 213.0881404 | 0.010375607 | 0.007141725 | 1.521616346 | 0.017098857 | 0.190062365 | 1.452815144 | 0.538851146 |
|  |  |  |  | 462.431 | 169.9936517 | 0.008790012 | 0.006056939 | 1.682848268 | 0.023561437 | 0.210268389 | 1.451230081 | 0.537276265 |
|  |  |  |  | 390.478 | 743.4068572 | 0.003844393 | 0.002652564 | 1.526325079 | 0.031878863 | 0.228920837 | 1.449312143 | 0.535368346 |
|  |  |  |  | 253.1555 | 546.284421 | 0.011778017 | 0.008128854 | 1.661044345 | 0.027555844 | 0.220176099 | 1.448914846 | 0.534972808 |
|  |  |  |  | 487.985 | 185.9887501 | 0.04305586 | 0.029770552 | 2.030835012 | 0.003816745 | 0.107922439 | 1.446256712 | 0.532323655 |
| Uridine 5'-monophosphate | 0.844193538 |  |  | 451.3525 | 323.0291518 | 2.751121448 | 1.902659761 | 1.411531129 | 0.036945112 | 0.23715393 | 1.445934531 | 0.532002231 |
|  |  | b-D-Glucopyranuronosyl-(1->3)-a-D-galactopyranuronosyl-(1->2)-a-L-rhamnopyranosyl-(1->4)-[b-D-glucopyranuronosyl-(1->3)]-a-D-galactopyranuronosyl-(1->2)-L-rhamnopyranose | 12.04110988 | 45.30625 | 1015.813696 | 0.032126648 | 0.022228289 | 1.588688705 | 0.01419431 | 0.17734804 | 1.445304562 | 0.531373537 |
|  |  |  |  | 440.249 | 272.018026 | 0.088367249 | 0.061173389 | 2.163742082 | 0.007386448 | 0.13405978 | 1.444537414 | 0.530607571 |
|  |  |  |  | 190.4795 | 198.0174295 | 0.072060169 | 0.0498848 | 1.444981194 | 0.026108401 | 0.216790478 | 1.444531583 | 0.530601747 |
|  |  |  |  | 361.0515 | 903.4656672 | 0.012194085 | 0.008444316 | 1.734609416 | 0.00559782 | 0.123050229 | 1.444058242 | 0.530128931 |
|  |  |  |  | 466.945 | 241.0118199 | 0.030629764 | 0.021212209 | 1.570617289 | 0.037309749 | 0.237679613 | 1.443968594 | 0.530039364 |
| D-Erythrose 4-phosphate | 0.997254846 |  |  | 459.123 | 199.0009181 | 1.602841129 | 1.110653683 | 2.164750824 | 0.000938128 | 0.066758981 | 1.44315114 | 0.5292224 |
|  |  |  |  | 482.431 | 275.0178503 | 0.126159367 | 0.087481172 | 2.12313675 | 0.001271055 | 0.072424035 | 1.442131653 | 0.528202875 |
|  |  |  |  | 459.141 | 200.0044773 | 0.069918241 | 0.048488809 | 2.165487143 | 0.00051212 | 0.057927367 | 1.441945933 | 0.528017071 |
|  |  |  |  | 449.563 | 354.0008266 | 0.039047152 | 0.027127596 | 2.015017483 | 0.002259775 | 0.088294291 | 1.439388608 | 0.525456145 |
|  |  |  |  | 377.8415 | 633.0378729 | 6.082573599 | 4.236196989 | 1.501665616 | 0.037799036 | 0.238372608 | 1.435857118 | 0.521912194 |
|  |  |  |  | 377.841 | 903.006419 | 0.554674531 | 0.387539422 | 1.636163714 | 0.022352347 | 0.207117035 | 1.431272535 | 0.517298409 |
|  |  |  |  | 423.9465 | 322.0535888 | 0.006393624 | 0.004475349 | 1.583713805 | 0.023613659 | 0.210399269 | 1.428631351 | 0.514633686 |
|  |  |  |  | 502.231 | 96.9685392 | 7.230456164 | 5.063258409 | 1.25415275 | 0.042348696 | 0.244930994 | 1.428024324 | 0.514020553 |
|  |  |  |  | 399.565 | 353.0606727 | 0.007446523 | 0.005222589 | 1.324536284 | 0.04209311 | 0.244402306 | 1.425829919 | 0.511801899 |
|  |  | 5-Fluorouridine monophosphate | -24.14318177 | 32.0952 | 343.1707155 | 0.194506499 | 0.136893863 | 1.830814075 | 0.002455623 | 0.091443443 | 1.420856236 | 0.506760588 |
|  |  |  |  | 377.8415 | 552.0351306 | 1.512454369 | 1.06565131 | 1.533274738 | 0.033646081 | 0.232005855 | 1.419276976 | 0.505156163 |
|  |  |  |  | 377.8415 | 634.0395743 | 1.121461546 | 0.790243235 | 1.549868808 | 0.033741612 | 0.23216565 | 1.419134635 | 0.505011466 |
|  |  |  |  | 58.8127 | 370.2794818 | 0.159586273 | 0.11285446 | 1.585185161 | 0.042860002 | 0.245976411 | 1.414089198 | 0.499873126 |
|  |  |  |  | 377.841 | 551.0337659 | 9.966827243 | 7.054573625 | 1.530247515 | 0.033484412 | 0.231733862 | 1.412817808 | 0.498575432 |
| 3-Phosphoglyceric acid | 0.945223769 |  |  | 487.908 | 184.9853541 | 1.352661115 | 0.958627249 | 2.040752342 | 0.003620386 | 0.106052367 | 1.411039709 | 0.496758588 |
|  |  |  |  | 470.2915 | 226.996645 | 0.099350572 | 0.070495277 | 2.176608286 | 0.000325696 | 0.053047263 | 1.409322387 | 0.49500167 |
|  |  |  |  | 468.2625 | 482.1032013 | 0.006752046 | 0.004793695 | 1.452605096 | 0.047397022 | 0.25462614 | 1.408526419 | 0.494186624 |
|  |  | L-Histidinol phosphate | 0.101513021 | 56.9226 | 222.158199 | 0.054861175 | 0.038973864 | 1.566382012 | 0.045693482 | 0.251489452 | 1.407640117 | 0.493278535 |
|  |  |  |  | 478.7155 | 153.0067459 | 0.020999094 | 0.014926688 | 1.916332819 | 0.020348775 | 0.201327597 | 1.406815325 | 0.492432956 |
|  |  |  |  | 447.984 | 486.0434671 | 0.01730224 | 0.012307958 | 1.456593923 | 0.034253888 | 0.233010974 | 1.405776593 | 0.491367338 |
|  |  |  |  | 219.271 | 233.0790616 | 0.169261453 | 0.12047428 | 1.439494718 | 0.049280075 | 0.25795938 | 1.404959238 | 0.490528274 |
|  |  |  |  | 57.0507 | 294.1798237 | 0.222401999 | 0.158328162 | 1.671219156 | 0.022845296 | 0.208430543 | 1.40469008 | 0.49025186 |
|  |  |  |  | 66.96655 | 152.0017745 | 0.020541355 | 0.014660792 | 1.365000692 | 0.044071322 | 0.248389883 | 1.401108147 | 0.486568317 |
|  |  |  |  | 451.234 | 481.0266968 | 0.024566608 | 0.017608984 | 1.626308111 | 0.006756665 | 0.130649787 | 1.395117825 | 0.48038697 |
|  |  | Tridodecylamine | -21.48188404 | 451.439 | 522.9834633 | 0.02956909 | 0.021209097 | 1.513665532 | 0.01900202 | 0.196974115 | 1.394170192 | 0.479406688 |
|  |  |  |  | 377.8355 | 944.0066883 | 0.075887466 | 0.054434746 | 1.496483007 | 0.037563998 | 0.238041465 | 1.394099761 | 0.479333804 |
|  |  |  |  | 160.316 | 112.0030689 | 0.141470552 | 0.101838637 | 1.86895356 | 0.00855738 | 0.141443423 | 1.389163836 | 0.474216759 |
|  |  | 4-Amino-2-methyl-5-(phosphooxymethyl)pyrimidine | 19.43293606 | 57.05195 | 220.146635 | 0.098926336 | 0.071303131 | 1.669477451 | 0.022571813 | 0.207706852 | 1.38740522 | 0.472389217 |
|  |  | Pyrophosphate | -22.63444655 | 474.584 | 174.9466395 | 0.134409787 | 0.096887422 | 1.597634055 | 0.041127156 | 0.242739853 | 1.387277978 | 0.472256898 |
|  |  |  |  | 271.066 | 284.0823293 | 0.019457289 | 0.014046461 | 1.604343571 | 0.049579542 | 0.258585854 | 1.385209365 | 0.470104046 |
|  |  |  |  | 454.517 | 243.0390135 | 0.141196667 | 0.102146841 | 1.97697811 | 0.004220643 | 0.111388008 | 1.382291082 | 0.467061449 |
| Isokobusone | 0.971259769 |  |  | 56.9967 | 221.1547909 | 0.385486201 | 0.2790253 | 1.518798174 | 0.043866373 | 0.247987643 | 1.38154569 | 0.466283275 |
|  |  |  |  | 377.8415 | 470.0337202 | 2.172103758 | 1.574411246 | 1.577632029 | 0.030007399 | 0.225362574 | 1.379629219 | 0.464280589 |
|  |  |  |  | 190.516 | 149.9954827 | 0.170810855 | 0.124273063 | 1.846788864 | 0.009158826 | 0.146333744 | 1.374480132 | 0.458886053 |
|  |  |  |  | 458.5145 | 582.1143903 | 0.005953025 | 0.004347343 | 1.590571755 | 0.023999065 | 0.211352475 | 1.36934772 | 0.453488838 |
|  |  |  |  | 270.2615 | 220.0826939 | 0.007868455 | 0.005749415 | 1.737861405 | 0.027002327 | 0.218912014 | 1.36856621 | 0.452665232 |
|  |  |  |  | 679.906 | 101.0598222 | 0.416836515 | 0.304617415 | 1.530146719 | 0.048920307 | 0.257335809 | 1.36839358 | 0.452483241 |
|  |  |  |  | 176.2505 | 149.9954803 | 0.26136531 | 0.191475243 | 1.7557074 | 0.00690256 | 0.131478849 | 1.365008375 | 0.448909802 |
|  |  |  |  | 449.094 | 139.9829721 | 0.077865425 | 0.05711484 | 1.949218951 | 0.003058757 | 0.099875697 | 1.363313383 | 0.447117231 |
|  |  |  |  | 377.8415 | 469.0288333 | 16.33170611 | 11.98037535 | 1.527539107 | 0.036261794 | 0.236146883 | 1.363204877 | 0.447002403 |
|  |  |  |  | 60.4737 | 427.3228196 | 0.196416575 | 0.144218177 | 1.579057178 | 0.036976248 | 0.23719913 | 1.361940497 | 0.445663674 |
|  |  |  |  | 377.841 | 389.0307179 | 0.689091579 | 0.506801535 | 1.684542274 | 0.015313009 | 0.182601469 | 1.359687239 | 0.443274835 |
|  |  |  |  | 377.841 | 822.0064924 | 0.14912344 | 0.110084707 | 1.503841824 | 0.029524811 | 0.224391461 | 1.354624482 | 0.437892975 |
|  |  |  |  | 329.8265 | 139.0256211 | 0.063599544 | 0.047173498 | 1.817049764 | 0.007958368 | 0.137468153 | 1.348204981 | 0.431039861 |
|  |  |  |  | 57.0268 | 236.1052073 | 0.553941732 | 0.41094698 | 1.585950279 | 0.036535574 | 0.236553859 | 1.347963993 | 0.430781959 |
|  |  |  |  | 444.008 | 257.0074118 | 0.713238236 | 0.530201387 | 2.077127671 | 0.001240416 | 0.071998795 | 1.345221369 | 0.427843602 |
|  |  |  |  | 46.0703 | 987.7826369 | 0.033297383 | 0.024833523 | 1.641218568 | 0.030975133 | 0.22724235 | 1.340823992 | 0.423119869 |
|  |  |  |  | 449.5545 | 214.0123265 | 0.223209401 | 0.166865641 | 1.819528921 | 0.011452253 | 0.162324803 | 1.337659443 | 0.419710864 |
|  |  |  |  | 488.252 | 358.0191514 | 0.012263398 | 0.009172897 | 1.593236292 | 0.026159802 | 0.216915246 | 1.336916657 | 0.418909531 |
|  |  |  |  | 60.3965 | 131.0340242 | 0.062235686 | 0.046631384 | 1.556501896 | 0.044208691 | 0.248658126 | 1.334630902 | 0.416440813 |
|  |  |  |  | 105.324 | 112.0224709 | 0.428624604 | 0.322144069 | 2.169747273 | 0.000201568 | 0.047081253 | 1.330537003 | 0.412008633 |
|  |  |  |  | 383.117 | 204.9703701 | 0.032670154 | 0.024565084 | 1.874692583 | 0.006490032 | 0.129067865 | 1.329942696 | 0.411364085 |
|  |  |  |  | 502.523 | 258.9862109 | 0.248694647 | 0.187021304 | 1.966812393 | 0.003683152 | 0.106664593 | 1.329766399 | 0.411172829 |
|  |  |  |  | 470.427 | 273.0019982 | 0.269046063 | 0.202370597 | 1.612355139 | 0.036341766 | 0.23626625 | 1.329472104 | 0.410853506 |
|  |  |  |  | 377.8415 | 387.0265123 | 30.58778871 | 23.08701437 | 1.542203615 | 0.032183093 | 0.229470003 | 1.324891483 | 0.405874198 |
| Inosinic acid | 0.802820308 |  |  | 457.7575 | 347.0405077 | 0.155051165 | 0.117323916 | 1.686803436 | 0.022927043 | 0.208644471 | 1.321564866 | 0.402247238 |
|  |  |  |  | 377.8415 | 388.0297552 | 3.355272034 | 2.53923018 | 1.506963849 | 0.038757254 | 0.239690129 | 1.321373722 | 0.402038559 |
|  |  | UDP-N-acetylmuramoyl-L-alanyl-D-glutamate | -10.76055132 | 154.6225 | 880.6060115 | 0.035656998 | 0.027033086 | 1.785686245 | 0.011162527 | 0.160506754 | 1.319013241 | 0.399459048 |
| 16-Hydroxy hexadecanoic acid | 0.987918154 |  |  | 50.7463 | 271.228608 | 0.222122953 | 0.168940235 | 1.562769275 | 0.044791619 | 0.249784394 | 1.314801967 | 0.39484552 |
|  |  |  |  | 477.752 | 350.051366 | 0.018887671 | 0.014377341 | 1.893605641 | 0.003151639 | 0.100993141 | 1.313710983 | 0.393647917 |
| Guanosine monophosphate | 0.807114846 |  |  | 473.099 | 362.051091 | 0.039181477 | 0.029831585 | 1.547084766 | 0.03006554 | 0.225478023 | 1.313422561 | 0.393331142 |
|  |  | 1,1-Dichloro-2,2-bis(4-chlorophenyl)ethylene | -14.80554782 | 221.971 | 319.0278681 | 0.033344077 | 0.025420993 | 1.584179482 | 0.029695382 | 0.224737345 | 1.31167482 | 0.391410103 |
|  |  |  |  | 652.328 | 336.8992203 | 0.003518816 | 0.0026852 | 1.391184236 | 0.035662963 | 0.23524 | 1.310448523 | 0.390060683 |
|  |  |  |  | 457.254 | 244.0233105 | 0.688762813 | 0.526916195 | 1.902193767 | 0.013994681 | 0.176357345 | 1.307158177 | 0.38643373 |
|  |  |  |  | 22.0353 | 283.1106305 | 0.029995665 | 0.023060111 | 1.653807336 | 0.023893298 | 0.211093096 | 1.300759817 | 0.379354595 |
|  |  |  |  | 213.079 | 552.3309901 | 0.09821999 | 0.076026752 | 1.666489785 | 0.044515032 | 0.249252413 | 1.291913541 | 0.369509524 |
|  |  |  |  | 460.888 | 184.0013606 | 0.074653952 | 0.057850672 | 1.868976268 | 0.012458517 | 0.168255453 | 1.290459551 | 0.367884922 |
|  |  |  |  | 441.4945 | 281.9699134 | 0.05029142 | 0.038988156 | 1.826523989 | 0.012040969 | 0.165863827 | 1.289915313 | 0.367276352 |
|  |  | Novclobiocin 105 | 21.42699349 | 474.0365 | 577.0111184 | 0.040386943 | 0.031324711 | 1.652091984 | 0.039241686 | 0.240337009 | 1.289299777 | 0.366587746 |
|  |  |  |  | 440.2755 | 324.9644125 | 0.101529778 | 0.078767372 | 2.10765702 | 0.003625863 | 0.106106353 | 1.288982691 | 0.366232891 |
|  |  |  |  | 488.6135 | 243.0258468 | 0.117521653 | 0.091317762 | 1.71295539 | 0.013535967 | 0.17401504 | 1.286952841 | 0.363959189 |
|  |  |  |  | 168.738 | 124.0396912 | 0.093419114 | 0.072615275 | 1.485478653 | 0.046216842 | 0.252458668 | 1.286493971 | 0.363444696 |
|  |  |  |  | 377.8415 | 307.0281746 | 0.682539174 | 0.531755535 | 1.605618151 | 0.02597395 | 0.216462466 | 1.283558194 | 0.360148706 |
|  |  |  |  | 449.326 | 320.9240116 | 0.015560442 | 0.012133403 | 1.736494416 | 0.019671477 | 0.19918891 | 1.282446643 | 0.358898803 |
|  |  |  |  | 377.8415 | 306.0272638 | 3.433282279 | 2.677165916 | 1.575988796 | 0.027917426 | 0.220982343 | 1.282431641 | 0.358881926 |
|  |  |  |  | 472.0605 | 324.9737571 | 0.156799443 | 0.123120195 | 1.885721333 | 0.005048414 | 0.118670303 | 1.273547712 | 0.34885301 |
|  |  |  |  | 167.824 | 768.5153312 | 1.445464642 | 1.137283139 | 1.524203377 | 0.04776475 | 0.255290768 | 1.270980456 | 0.345941846 |
|  |  |  |  | 377.8415 | 305.0240088 | 37.26882202 | 29.3336039 | 1.589391514 | 0.024676201 | 0.213168636 | 1.270516305 | 0.34541489 |
|  |  |  |  | 260.529 | 522.2843124 | 0.014548567 | 0.011473279 | 1.386190508 | 0.046369636 | 0.252738886 | 1.268039155 | 0.342599294 |
|  |  | Diclobutrazol | 15.55978261 | 37.4235 | 329.2492839 | 1.00999304 | 0.797624483 | 1.588565083 | 0.038126461 | 0.238828611 | 1.266251303 | 0.340563753 |
|  |  |  |  | 511.341 | 392.073671 | 0.102018922 | 0.080853692 | 1.446891075 | 0.043094719 | 0.246450934 | 1.261771961 | 0.335451197 |
|  |  | Phaseolic acid | 22.9373309 | 619.235 | 297.2436713 | 0.031231571 | 0.02475776 | 1.874181867 | 0.002691394 | 0.095007002 | 1.261486096 | 0.335124305 |
|  |  | PE-NMe(15:0/20:5(5Z,8Z,11Z,14Z,17Z)) | -7.241536573 | 377.842 | 739.0019323 | 1.012095409 | 0.802308203 | 1.607493849 | 0.021670248 | 0.205230383 | 1.261479573 | 0.335116846 |
|  |  |  |  | 377.842 | 225.0240479 | 0.34080366 | 0.270200983 | 1.56823979 | 0.021577715 | 0.204968004 | 1.261296893 | 0.334907907 |
|  |  |  |  | 483.661 | 399.0454343 | 0.007787643 | 0.00622011 | 1.551864981 | 0.03832972 | 0.239108632 | 1.252010505 | 0.324246668 |
|  |  |  |  | 415.038 | 356.9930139 | 0.02497014 | 0.019945928 | 1.538617708 | 0.049276533 | 0.25795327 | 1.251891584 | 0.324109628 |
|  |  |  |  | 471.3205 | 384.9950698 | 0.032720411 | 0.026161309 | 1.62665708 | 0.032574882 | 0.230165874 | 1.250717658 | 0.322756146 |
|  |  |  |  | 377.842 | 656.9992521 | 1.249875508 | 0.999901874 | 1.672193145 | 0.015678393 | 0.184215049 | 1.249998165 | 0.321925977 |
|  |  | Dichlorphemide | 17.64627023 | 530.2645 | 306.1714615 | 0.005008218 | 0.004012143 | 1.743720923 | 0.008491863 | 0.141014108 | 1.248264972 | 0.319924211 |
|  |  |  |  | 223.687 | 453.2813787 | 0.056363764 | 0.045242058 | 1.708325671 | 0.031936744 | 0.22902592 | 1.245826703 | 0.317103401 |
|  |  |  |  | 441.217 | 354.975272 | 0.175420127 | 0.140806791 | 1.83098507 | 0.011572302 | 0.163063013 | 1.245821495 | 0.31709737 |
|  |  |  |  | 471.254 | 415.0049214 | 0.170220146 | 0.136648216 | 1.617528529 | 0.049921873 | 0.259296454 | 1.24568143 | 0.316935161 |
|  |  |  |  | 167.829 | 766.5168887 | 3.927817914 | 3.161521917 | 1.561323702 | 0.035875776 | 0.235564953 | 1.242381997 | 0.31310883 |
|  |  | 4-(6-carboxy-2,3,4-trihydroxyphenoxy)-4',5,5',6,6'-pentahydroxy-[1,1'-biphenyl]-2,2'-dicarboxylic acid | -12.11370959 | 215.7725 | 507.3291431 | 0.012352754 | 0.009958895 | 1.615294137 | 0.038480807 | 0.23931528 | 1.240374035 | 0.310775232 |
|  |  |  |  | 263.525 | 496.2692775 | 0.017800373 | 0.01435269 | 1.736566344 | 0.012124497 | 0.166349871 | 1.240211676 | 0.310586377 |
|  |  |  |  | 395.8885 | 221.0045106 | 0.026971891 | 0.021747986 | 1.866296902 | 0.012130452 | 0.166384373 | 1.240201767 | 0.31057485 |
|  |  |  |  | 448.78 | 338.9346111 | 0.202772087 | 0.164549663 | 1.606328439 | 0.031003262 | 0.227295688 | 1.232285035 | 0.301335999 |
|  |  |  |  | 508.786 | 410.084054 | 0.016320203 | 0.013259684 | 1.640731771 | 0.021936461 | 0.205976541 | 1.23081387 | 0.299612606 |
|  |  |  |  | 219.4015 | 480.3106674 | 0.890418476 | 0.725534704 | 1.558711449 | 0.048181758 | 0.256036294 | 1.227258285 | 0.295438906 |
|  |  |  |  | 467.489 | 214.0123466 | 0.074559352 | 0.060755732 | 1.813471169 | 0.021934426 | 0.205970888 | 1.227198635 | 0.295368784 |
|  |  |  |  | 49.7938 | 171.1383868 | 0.512630784 | 0.418290373 | 1.986072869 | 0.003689382 | 0.106724608 | 1.225538088 | 0.293415322 |
|  |  |  |  | 471.269 | 354.9843307 | 0.097683944 | 0.079859071 | 1.723741202 | 0.021586064 | 0.204991743 | 1.223204106 | 0.290665154 |
|  |  |  |  | 453.2535 | 196.0012551 | 0.089698611 | 0.073496743 | 1.967277876 | 0.000434478 | 0.056172163 | 1.220443347 | 0.287405326 |
|  |  |  |  | 158.951 | 841.5828141 | 0.435577488 | 0.356904875 | 1.757086063 | 0.012284964 | 0.167272846 | 1.2204302 | 0.287389785 |
|  |  |  |  | 456.412 | 257.0074016 | 0.287095548 | 0.235829856 | 1.675901318 | 0.049237503 | 0.257885909 | 1.217384232 | 0.283784585 |
|  |  |  |  | 377.8415 | 224.0236858 | 1.979304067 | 1.626561759 | 1.681114574 | 0.01719017 | 0.190416737 | 1.216863766 | 0.28316766 |
|  |  |  |  | 377.8415 | 223.020179 | 30.94688486 | 25.49238155 | 1.600668221 | 0.023280834 | 0.209557921 | 1.213966016 | 0.279728035 |
| D-Glyceraldehyde 3-phosphate | 0.992016385 |  |  | 462.5065 | 168.9902109 | 0.393104813 | 0.325178136 | 1.970309241 | 0.004320644 | 0.112175854 | 1.208890664 | 0.273683769 |
|  |  |  |  | 448.78 | 138.9794719 | 3.482914416 | 2.883039643 | 1.699515056 | 0.02637875 | 0.217442851 | 1.208070248 | 0.272704348 |
|  |  |  |  | 157.18 | 866.5948316 | 0.606465175 | 0.502228194 | 1.479420167 | 0.046500106 | 0.252977195 | 1.207549041 | 0.272081781 |
|  |  | DHAP(6:0) | 16.93476753 | 58.4695 | 269.2126185 | 0.087830014 | 0.072739905 | 2.012065812 | 0.004452323 | 0.113175064 | 1.207452958 | 0.271966983 |
|  |  |  |  | 223.221 | 452.2779959 | 0.25024491 | 0.208105708 | 1.712261836 | 0.031126659 | 0.227528826 | 1.202489412 | 0.266024191 |
|  |  | Diallat | 8.389139535 | 36.4462 | 271.2286435 | 0.485446989 | 0.404406455 | 1.7955838 | 0.011779764 | 0.164318517 | 1.200393771 | 0.263507737 |
|  |  |  |  | 487.392 | 96.96855105 | 7.71371212 | 6.433310586 | 1.679245099 | 0.021322073 | 0.204234862 | 1.199026849 | 0.261863964 |
|  |  |  |  | 441.171 | 251.9586668 | 0.233232683 | 0.194628475 | 1.939189294 | 0.006250516 | 0.127567994 | 1.198348203 | 0.261047171 |
|  |  | Arsonoacetate | -8.903340086 | 455.139 | 184.9853386 | 0.12706295 | 0.106149168 | 2.009698978 | 0.001538438 | 0.075831784 | 1.19702257 | 0.259450355 |
|  |  |  |  | 157.184 | 867.5954016 | 0.316370478 | 0.264553589 | 1.447922107 | 0.039191485 | 0.240270556 | 1.195865379 | 0.258054991 |
|  |  |  |  | 377.842 | 574.9948001 | 1.083702387 | 0.907417935 | 1.552643146 | 0.028219841 | 0.221645185 | 1.194270407 | 0.256129529 |
|  |  |  |  | 377.8435 | 492.9900957 | 0.7875019 | 0.659905387 | 1.765388258 | 0.007414397 | 0.134201435 | 1.193355768 | 0.255024209 |
|  |  | Oxalyl-CoA | 16.99772464 | 158.981 | 840.5752471 | 0.912106911 | 0.767516797 | 2.073136516 | 0.001486408 | 0.075023088 | 1.188386904 | 0.249004612 |
|  |  |  |  | 454.6165 | 166.9748495 | 0.116839933 | 0.098433006 | 1.727715222 | 0.014419707 | 0.178446502 | 1.186999539 | 0.247319374 |
|  |  |  |  | 152.676 | 917.6105835 | 0.015810586 | 0.013347588 | 1.548953547 | 0.029268516 | 0.223866208 | 1.184527565 | 0.244311772 |
|  |  |  |  | 71.21305 | 199.1700273 | 0.037948798 | 0.032059 | 1.581624325 | 0.034982477 | 0.234180662 | 1.183717478 | 0.243324789 |
|  |  |  |  | 217.44 | 555.3498701 | 0.166937349 | 0.141150896 | 1.826658739 | 0.01546684 | 0.183286627 | 1.182687139 | 0.242068483 |
|  |  |  |  | 30.1976 | 697.50641 | 0.103039538 | 0.087127271 | 1.878602798 | 0.010206923 | 0.154118777 | 1.18263245 | 0.242001769 |
|  |  |  |  | 158.051 | 826.5982472 | 0.039483171 | 0.033543577 | 1.483918693 | 0.02958814 | 0.224520221 | 1.17707098 | 0.235201321 |
|  |  |  |  | 152.7255 | 916.6105358 | 0.029174578 | 0.02490153 | 1.66414193 | 0.015129425 | 0.18177246 | 1.171597837 | 0.228477435 |
|  |  |  |  | 377.843 | 427.0197137 | 1.000683868 | 0.857241515 | 1.67547246 | 0.012123174 | 0.166342202 | 1.167330153 | 0.223212652 |
|  |  |  |  | 167.8325 | 767.5203503 | 1.66490867 | 1.429836828 | 1.527505003 | 0.040960713 | 0.242534782 | 1.164404663 | 0.219592522 |
|  |  | Permanganate | 17.8159178 | 377.842 | 127.0005212 | 0.504206109 | 0.433613761 | 1.735886071 | 0.008277051 | 0.139578025 | 1.162800066 | 0.217603058 |
|  |  |  |  | 377.843 | 410.9882761 | 0.882598553 | 0.76339972 | 1.483168964 | 0.027102841 | 0.219144303 | 1.156142097 | 0.209318725 |
|  |  |  |  | 377.8425 | 141.0160292 | 274.4297596 | 237.6831726 | 1.604137276 | 0.026308072 | 0.217273215 | 1.154603233 | 0.20739717 |
|  |  |  |  | 377.8415 | 142.0195585 | 12.79471553 | 11.13343059 | 1.564067615 | 0.032789035 | 0.230540952 | 1.149215907 | 0.200649867 |
|  |  |  |  | 200.6595 | 320.0560947 | 0.077745416 | 0.067860121 | 1.576324311 | 0.03621974 | 0.236083949 | 1.145671638 | 0.196193611 |
|  |  |  |  | 377.8445 | 82.00257488 | 0.295784824 | 0.259230818 | 1.555092295 | 0.036867954 | 0.237041663 | 1.141009494 | 0.190310795 |
|  |  |  |  | 377.8505 | 345.0158028 | 1.46837277 | 1.287290511 | 1.628131433 | 0.024093101 | 0.211608906 | 1.140669304 | 0.189880595 |
|  |  | 3-phosphonato-D-glyceroyl Phosphate(4-) | 0.651595611 | 378.703 | 263.0124473 | 0.727368893 | 0.644273139 | 1.645998944 | 0.031011095 | 0.227310528 | 1.128975971 | 0.17501478 |
|  |  |  |  | 377.8425 | 143.0204846 | 2.352012291 | 2.086075978 | 1.549697769 | 0.027062595 | 0.219051443 | 1.127481605 | 0.173103896 |
|  |  |  |  | 275.634 | 131.0341964 | 0.029691392 | 0.02655825 | 1.549978639 | 0.013325356 | 0.172933176 | 1.117972466 | 0.160884657 |
|  |  |  |  | 377.9775 | 328.9852373 | 0.434042442 | 0.388349657 | 1.816701805 | 0.002355318 | 0.089805893 | 1.117658877 | 0.160479928 |
|  |  |  |  | 89.3665 | 142.059977 | 0.248545691 | 0.271602689 | 1.604328455 | 0.033753669 | 0.232185768 | 0.915107623 | -0.127986671 |
|  |  |  |  | 122.82 | 138.9892404 | 0.039043491 | 0.043887993 | 1.469849639 | 0.04107708 | 0.242678294 | 0.889616673 | -0.168744268 |
|  |  |  |  | 440.046 | 176.9163772 | 0.074976593 | 0.087060499 | 1.507488055 | 0.034105455 | 0.232768023 | 0.861201049 | -0.215578018 |
| Hydroxypyruvic acid | 0.962886769 |  |  | 445.826 | 103.0029011 | 0.074071533 | 0.086040213 | 1.572771524 | 0.029687885 | 0.224722204 | 0.860894343 | -0.216091907 |
|  |  |  |  | 38.3286 | 804.6133071 | 0.311061048 | 0.362800058 | 1.590480151 | 0.032923402 | 0.230774414 | 0.857389744 | -0.221976936 |
|  |  |  |  | 467.588 | 117.0186462 | 0.187739441 | 0.220593418 | 1.682626865 | 0.026633101 | 0.218048019 | 0.851065473 | -0.232657971 |
|  |  |  |  | 388.7005 | 109.0399258 | 0.049039977 | 0.058015077 | 1.910001427 | 0.007855656 | 0.136828898 | 0.845297108 | -0.242469581 |
|  |  |  |  | 661.8635 | 130.0867291 | 0.071772853 | 0.085016574 | 1.734882657 | 0.019099536 | 0.197303246 | 0.844221889 | -0.244305859 |
|  |  |  |  | 311.471 | 74.02378696 | 0.102558964 | 0.123073831 | 1.437330598 | 0.041036896 | 0.242628809 | 0.83331252 | -0.263070439 |
|  |  |  |  | 679.992 | 130.0867285 | 0.053347827 | 0.064512632 | 1.701148302 | 0.017229586 | 0.190568947 | 0.826936135 | -0.274152182 |
|  |  |  |  | 39.0698 | 804.5765791 | 0.314262436 | 0.380851139 | 1.74494146 | 0.013449344 | 0.173572549 | 0.825158188 | -0.277257375 |
|  |  |  |  | 364.566 | 212.0650428 | 0.015209983 | 0.018458989 | 1.828863615 | 0.011328312 | 0.161553451 | 0.823987879 | -0.279304979 |
|  |  |  |  | 38.3319 | 818.5939867 | 2.175022495 | 2.646300392 | 1.565078445 | 0.038452197 | 0.239276247 | 0.821910658 | -0.282946515 |
|  |  | Tetrafluoroethylene | 12.76762569 | 255.114 | 101.0235536 | 9.333304532 | 11.40085169 | 1.69728187 | 0.035394659 | 0.234826047 | 0.818649763 | -0.288681729 |
|  |  |  |  | 440.14 | 175.9365259 | 0.017397033 | 0.0212649 | 1.442823123 | 0.02764327 | 0.220372429 | 0.818110261 | -0.289632799 |
|  |  | 2-({2-[4-(1,2-dihydroxyethyl)-5,11,12,13-tetrahydroxy-8-oxo-3,7-dioxatricyclo[7.4.0.0trideca-1(13),9,11-trien-10-yl]-3,4,8,9,10-pentahydroxy-6-oxo-6H-benzo[c]chromen-1-yl}formamido)acetic acid | 9.342792399 | 169.592 | 690.5047184 | 0.168153054 | 0.205583474 | 1.401267603 | 0.039730731 | 0.240977469 | 0.817930791 | -0.28994932 |
|  |  |  |  | 196.532 | 218.0065825 | 0.283186425 | 0.346349889 | 1.476714611 | 0.042273098 | 0.244775046 | 0.817631054 | -0.290478103 |
|  |  |  |  | 341.84 | 238.9941416 | 0.024358153 | 0.02984944 | 1.699078074 | 0.010628699 | 0.157015443 | 0.81603382 | -0.293299149 |
|  |  |  |  | 300.316 | 212.0677741 | 0.008921496 | 0.010955179 | 1.762686055 | 0.014994611 | 0.181155679 | 0.814363354 | -0.296255453 |
|  |  |  |  | 364.779 | 215.0394952 | 0.024830553 | 0.030527926 | 1.439117234 | 0.022406252 | 0.207262663 | 0.813371753 | -0.298013205 |
|  |  |  |  | 199.838 | 885.5511351 | 1.804697147 | 2.264518176 | 1.767007807 | 0.013736484 | 0.175050441 | 0.796945313 | -0.327447365 |
|  |  |  |  | 397.666 | 170.0453434 | 0.038115565 | 0.04794269 | 1.591151449 | 0.040983653 | 0.242563124 | 0.795023489 | -0.33093061 |
|  |  |  |  | 486.108 | 136.9638894 | 0.71916546 | 0.907002214 | 1.489119726 | 0.036055349 | 0.235836856 | 0.792903753 | -0.33478234 |
|  |  |  |  | 199.769 | 886.5562367 | 0.899088574 | 1.140626036 | 1.686508699 | 0.021734184 | 0.205410757 | 0.788241321 | -0.343290714 |
|  |  |  |  | 151.2385 | 144.0448055 | 0.01658729 | 0.021109476 | 1.487395292 | 0.048823057 | 0.257166195 | 0.785774597 | -0.347812567 |
|  |  |  |  | 61.5806 | 124.970783 | 0.117537782 | 0.14962168 | 1.732744374 | 0.025791545 | 0.216013611 | 0.78556652 | -0.348194651 |
|  |  |  |  | 159.9195 | 777.5276203 | 0.038601233 | 0.049219244 | 1.769754655 | 0.013169374 | 0.172118604 | 0.784271147 | -0.35057557 |
|  |  |  |  | 158.1085 | 720.4994287 | 0.077275719 | 0.099013936 | 1.875445856 | 0.010056731 | 0.153056113 | 0.780452959 | -0.357616418 |
|  |  |  |  | 600.7435 | 222.9683138 | 0.0474992 | 0.061121652 | 1.497754281 | 0.037109369 | 0.237391723 | 0.777125582 | -0.36378034 |
|  |  | Fe(III)dicitrate | -2.131820869 | 458.969 | 435.0507513 | 0.009647872 | 0.012416993 | 1.665975386 | 0.027553793 | 0.220171483 | 0.776989433 | -0.364033117 |
|  |  |  |  | 711.323 | 314.9284818 | 0.017916575 | 0.02310893 | 1.465484503 | 0.022430346 | 0.207327593 | 0.775309606 | -0.367155555 |
|  |  |  |  | 83.9132 | 742.5406362 | 0.295015577 | 0.382343663 | 1.487623949 | 0.035635547 | 0.23519792 | 0.771597926 | -0.374078829 |
|  |  |  |  | 444.2765 | 237.8715274 | 0.014341596 | 0.018728754 | 1.651867235 | 0.040399017 | 0.241832886 | 0.765752821 | -0.385049318 |
|  |  |  |  | 277.274 | 180.0774534 | 0.028258019 | 0.03694985 | 1.726709218 | 0.020104894 | 0.200568882 | 0.76476681 | -0.386908182 |
|  |  |  |  | 187.609 | 190.0368568 | 0.05167237 | 0.067636953 | 1.648309604 | 0.044931042 | 0.250050926 | 0.763966561 | -0.388418602 |
|  |  |  |  | 159.869 | 776.522881 | 0.078145608 | 0.102293565 | 1.882169608 | 0.005360946 | 0.121233522 | 0.763934736 | -0.388478703 |
|  |  |  |  | 158.122 | 721.5039791 | 0.035046328 | 0.045949246 | 1.788607993 | 0.011487886 | 0.162544826 | 0.762718235 | -0.390777903 |
|  |  | [2,6-dihydroxy-4-({[3-hydroxy-6-(hydroxymethyl)-2,5-bis(3,4,5-trihydroxybenzoyloxy)oxan-4-yl]oxy}carbonyl)phenyl]oxidanesulfonic acid | -13.60414779 | 39.1159 | 717.5275288 | 0.098896166 | 0.129666387 | 1.500230374 | 0.041435803 | 0.243116674 | 0.762697008 | -0.390818055 |
|  |  |  |  | 115.046 | 157.1227311 | 0.189635426 | 0.249183393 | 1.591255139 | 0.04584543 | 0.251772353 | 0.761027543 | -0.393979426 |
|  |  |  |  | 38.4073 | 819.594465 | 0.779000242 | 1.02731219 | 1.499656258 | 0.046722083 | 0.253388332 | 0.758289689 | -0.399178988 |
|  |  | N(omega)-(ADP-D-ribosyl)-L-arginine | 22.92010888 | 167.008 | 716.525076 | 1.076250267 | 1.421099417 | 1.53853267 | 0.048749118 | 0.257036935 | 0.757336365 | -0.400993891 |
|  |  |  |  | 192.103 | 279.029617 | 0.216593205 | 0.286047763 | 1.681398038 | 0.02308623 | 0.20905795 | 0.757192444 | -0.40126808 |
|  |  |  |  | 206.865 | 911.5659287 | 0.091853936 | 0.121366835 | 1.596276455 | 0.031653275 | 0.228508547 | 0.756828967 | -0.401960788 |
| Acetylglycine | 0.942104154 |  |  | 311.5465 | 116.0345452 | 0.222361759 | 0.293958496 | 1.705409541 | 0.018138566 | 0.193957458 | 0.756439299 | -0.402703778 |
|  |  |  |  | 34.8106 | 153.9810706 | 0.870084854 | 1.150803159 | 1.533066189 | 0.045077844 | 0.250330392 | 0.756067489 | -0.403413076 |
|  |  |  |  | 486.154 | 292.9473821 | 0.463329405 | 0.613910534 | 1.568973476 | 0.028794901 | 0.22287769 | 0.754718122 | -0.405990179 |
|  |  |  |  | 197.14 | 137.9399254 | 0.17170688 | 0.228334377 | 1.410195329 | 0.039160169 | 0.240229033 | 0.751997497 | -0.411200234 |
| N-Acetylglutamic acid | 0.886798154 |  |  | 397.048 | 188.0563313 | 1.730365402 | 2.307792579 | 1.855032449 | 0.008351088 | 0.140077988 | 0.749792428 | -0.415436838 |
|  |  |  |  | 514.577 | 191.0194062 | 0.148811742 | 0.198613602 | 1.683134922 | 0.037332533 | 0.237712195 | 0.74925252 | -0.416476064 |
|  |  |  |  | 455.1905 | 271.0573944 | 0.030870881 | 0.041251561 | 1.629585896 | 0.038929194 | 0.23992117 | 0.748356671 | -0.418202063 |
|  |  |  |  | 153.6535 | 772.5303134 | 0.100604719 | 0.134907193 | 1.829082254 | 0.00893493 | 0.144551867 | 0.745732799 | -0.4232693 |
|  |  |  |  | 156.286 | 746.5153914 | 0.360973783 | 0.484935387 | 1.54735764 | 0.03657113 | 0.236606369 | 0.74437501 | -0.425898472 |
|  |  |  |  | 445.8405 | 85.0285458 | 0.185736731 | 0.250106515 | 1.61074117 | 0.041059907 | 0.242657156 | 0.742630521 | -0.429283485 |
|  |  | (2E)-Pentenoyl-CoA | 1.779825997 | 160.8315 | 846.6187816 | 0.559497697 | 0.756422988 | 1.693819284 | 0.009652787 | 0.150112554 | 0.739662473 | -0.435061013 |
|  |  | N(omega)-(ADP-D-ribosyl)-L-arginine | 23.27296385 | 38.3319 | 716.5253284 | 0.291069244 | 0.393655439 | 1.603988689 | 0.032482599 | 0.2300031 | 0.739401047 | -0.435571009 |
|  |  |  |  | 363.708 | 239.0774329 | 0.010046497 | 0.013604365 | 1.313134848 | 0.036067726 | 0.23585552 | 0.738475986 | -0.437377087 |
|  |  |  |  | 98.5355 | 742.5407028 | 0.233565464 | 0.317048519 | 1.790838062 | 0.007105178 | 0.132590024 | 0.736686815 | -0.440876673 |
| Itaconic acid | 0.999669308 |  |  | 445.7785 | 129.0185815 | 0.457099401 | 0.620780455 | 1.622196931 | 0.04232573 | 0.244883655 | 0.736330206 | -0.441575209 |
|  |  |  |  | 37.38455 | 762.5078164 | 0.691366557 | 0.941042051 | 1.718077763 | 0.02134216 | 0.204292914 | 0.734681894 | -0.444808374 |
|  |  |  |  | 216.688 | 761.5184967 | 0.357222436 | 0.487222613 | 1.512132126 | 0.035875939 | 0.235565201 | 0.733181149 | -0.447758402 |
|  |  |  |  | 311.486 | 377.0999926 | 0.03448956 | 0.047077963 | 1.477876152 | 0.048449547 | 0.256510536 | 0.732605205 | -0.448892144 |
|  |  |  |  | 486.11 | 293.9511698 | 0.01904514 | 0.026019905 | 1.542010424 | 0.028074362 | 0.221327606 | 0.731945018 | -0.450192815 |
|  |  |  |  | 464.6385 | 314.8582635 | 0.024268185 | 0.033195133 | 1.509060055 | 0.041161394 | 0.242781875 | 0.731076587 | -0.451905545 |
|  |  |  |  | 444.095 | 240.8713429 | 0.019409108 | 0.026598059 | 2.108305642 | 0.000109623 | 0.042353184 | 0.729718973 | -0.45458713 |
|  |  |  |  | 242.092 | 111.0192286 | 0.649511262 | 0.891114809 | 1.695541731 | 0.010297665 | 0.154752738 | 0.72887495 | -0.456256777 |
|  |  |  |  | 162.502 | 833.6128263 | 0.077135026 | 0.105998255 | 1.616227774 | 0.011962295 | 0.165402465 | 0.727700904 | -0.458582493 |
|  |  |  |  | 300.535 | 272.0625938 | 1.400398305 | 1.926791005 | 1.468986927 | 0.039139754 | 0.240201938 | 0.726803427 | -0.460362873 |
|  |  |  |  | 580.38 | 210.9318689 | 0.057864599 | 0.079634362 | 1.58221867 | 0.040347876 | 0.241768214 | 0.726628522 | -0.460710098 |
|  |  |  |  | 455.1905 | 289.068852 | 0.037141119 | 0.051487974 | 1.460240621 | 0.036273872 | 0.236164936 | 0.721355211 | -0.471218245 |
|  |  | [2,6-dihydroxy-4-({[3-hydroxy-6-(hydroxymethyl)-2,5-bis(3,4,5-trihydroxybenzoyloxy)oxan-4-yl]oxy}carbonyl)phenyl]oxidanesulfonic acid | -14.89090102 | 166.955 | 717.5266068 | 0.435586751 | 0.604478038 | 1.660267583 | 0.034412019 | 0.233268042 | 0.720599797 | -0.47272985 |
|  |  | 3,5-Dichloro-4-hydroxyphenylpyruvate | 16.07397109 | 410.014 | 250.0587798 | 0.174762624 | 0.242539951 | 1.736572695 | 0.033590332 | 0.231912286 | 0.720551907 | -0.472825731 |
|  |  |  |  | 462.052 | 237.8715297 | 0.018336202 | 0.025488542 | 1.576477769 | 0.030799345 | 0.226907389 | 0.719390002 | -0.475153986 |
|  |  |  |  | 156.281 | 748.5286822 | 0.1262964 | 0.176573552 | 1.84590944 | 0.006988794 | 0.131957344 | 0.715262272 | -0.483455749 |
|  |  |  |  | 46.5412 | 112.9871461 | 0.52233001 | 0.73117466 | 1.755001832 | 0.012604993 | 0.169072396 | 0.71437105 | -0.485254479 |
|  |  |  |  | 311.221 | 346.9901543 | 0.006557358 | 0.009179777 | 1.574143657 | 0.047911526 | 0.255554156 | 0.714326525 | -0.485344401 |
|  |  |  |  | 311.45 | 104.0344059 | 0.030124369 | 0.042221917 | 1.58632869 | 0.03758786 | 0.238075231 | 0.71347706 | -0.48706105 |
|  |  |  |  | 406.823 | 367.1061776 | 0.032269943 | 0.045263913 | 2.132850021 | 0.00561753 | 0.123196832 | 0.712928714 | -0.488170267 |
|  |  |  |  | 402.435 | 267.0725974 | 0.015343473 | 0.021573294 | 1.533620504 | 0.026705144 | 0.218217935 | 0.711225319 | -0.491621411 |
|  |  |  |  | 39.078 | 832.6070056 | 0.069841864 | 0.098272031 | 1.533783653 | 0.034298546 | 0.233083756 | 0.710699302 | -0.492688812 |
|  |  |  |  | 419.071 | 374.0511122 | 0.006600938 | 0.00929567 | 1.77902204 | 0.010285042 | 0.154664908 | 0.710108935 | -0.493887736 |
|  |  | Bretylium | -16.49756014 | 394.3985 | 244.166665 | 0.006533093 | 0.00921054 | 1.465899002 | 0.043633039 | 0.247526699 | 0.709306195 | -0.495519546 |
|  |  |  |  | 188.502 | 71.01287771 | 0.173304579 | 0.244548906 | 1.654986521 | 0.016757112 | 0.18871399 | 0.708670433 | -0.496813237 |
|  |  | Rhein | -16.47592428 | 30.34425 | 285.2229938 | 0.079523308 | 0.112238906 | 1.487779599 | 0.035603642 | 0.235148888 | 0.708518202 | -0.497123178 |
|  |  |  |  | 39.2116 | 778.5981554 | 0.057292421 | 0.081024939 | 1.480862179 | 0.043576884 | 0.247415288 | 0.707096132 | -0.500021727 |
|  |  |  |  | 467.478 | 211.9965775 | 0.062388507 | 0.088255468 | 2.014928869 | 0.000667995 | 0.060922031 | 0.706908117 | -0.500405387 |
|  |  |  |  | 455.211 | 246.0619725 | 0.029325009 | 0.041618212 | 1.742058792 | 0.025019365 | 0.214062832 | 0.704619626 | -0.505083436 |
|  |  | (2E)-Pentenoyl-CoA | 2.730753939 | 38.3178 | 846.6195858 | 0.224733613 | 0.319332676 | 1.675399491 | 0.014263237 | 0.177686188 | 0.703760152 | -0.506844266 |
|  |  | 4-Bromo-3,5-cyclohexadiene-1,2-dione | 22.13072497 | 355.455 | 188.0022148 | 0.024466425 | 0.034766177 | 1.796938029 | 0.01581906 | 0.184823697 | 0.703742184 | -0.506881101 |
|  |  |  |  | 451.572 | 373.0678797 | 0.010381718 | 0.014753674 | 1.873969022 | 0.005890142 | 0.125156808 | 0.703670044 | -0.507028998 |
|  |  |  |  | 347.407 | 135.0292579 | 0.165370417 | 0.23506331 | 1.949984842 | 0.00088172 | 0.065507482 | 0.703514373 | -0.507348197 |
|  |  |  |  | 156.28 | 749.5347173 | 0.043122024 | 0.0613114 | 1.844338398 | 0.008817756 | 0.14360135 | 0.703327993 | -0.507730456 |
|  |  |  |  | 123.922 | 846.5809714 | 0.038281794 | 0.05453435 | 1.654502317 | 0.013646188 | 0.174586432 | 0.701975803 | -0.510506794 |
|  |  |  |  | 169.5835 | 686.4775158 | 0.396244569 | 0.565036244 | 1.577469484 | 0.021560891 | 0.20492013 | 0.701272835 | -0.511952251 |
| L-Proline | 0.999776538 |  |  | 326.9725 | 114.0551448 | 18.1762179 | 25.98738315 | 1.592189791 | 0.032694805 | 0.230376368 | 0.699424709 | -0.515759331 |
|  |  |  |  | 354.539 | 204.05134 | 0.01203317 | 0.017210818 | 1.537591738 | 0.027620733 | 0.220321904 | 0.699163152 | -0.516298943 |
|  |  |  |  | 462.7735 | 308.8556892 | 0.024096647 | 0.034478708 | 1.445012474 | 0.044214075 | 0.248668616 | 0.698884859 | -0.516873303 |
|  |  |  |  | 66.48445 | 112.9871416 | 0.527806471 | 0.756856561 | 1.819954578 | 0.018818762 | 0.196349395 | 0.69736658 | -0.520010868 |
|  |  |  |  | 312.864 | 119.9753233 | 0.037711866 | 0.0542011 | 1.649584709 | 0.032086271 | 0.229296074 | 0.695776762 | -0.5233036 |
|  |  |  |  | 311.252 | 106.9799194 | 0.00982253 | 0.014133841 | 1.769242233 | 0.030749447 | 0.226811794 | 0.694965413 | -0.524986916 |
|  |  |  |  | 355.269 | 331.054937 | 0.0047781 | 0.006905815 | 1.902739132 | 0.002944479 | 0.0984413 | 0.691895137 | -0.531374694 |
|  |  |  |  | 300.4265 | 274.0671632 | 0.023888744 | 0.034561945 | 1.479156639 | 0.033421279 | 0.231627105 | 0.69118633 | -0.532853411 |
|  |  |  |  | 488.897 | 189.0040184 | 0.028468445 | 0.041228146 | 1.481497185 | 0.043780312 | 0.247818004 | 0.690509935 | -0.534265923 |
|  |  |  |  | 11.3266 | 121.0286176 | 0.099613349 | 0.144317234 | 1.469809493 | 0.048633752 | 0.256834729 | 0.690238764 | -0.534832597 |
|  |  |  |  | 444.399 | 317.0282371 | 0.007142013 | 0.010394842 | 1.760693165 | 0.017432671 | 0.19134606 | 0.687072824 | -0.541465075 |
|  |  |  |  | 272.941 | 87.01902316 | 0.14523757 | 0.212038092 | 1.704530908 | 0.019331618 | 0.19807752 | 0.684959805 | -0.545908764 |
|  |  |  |  | 38.2885 | 742.5408795 | 0.282873801 | 0.413598727 | 1.918231549 | 0.003028954 | 0.099508108 | 0.683932959 | -0.548073181 |
|  |  |  |  | 60.6812 | 688.4927009 | 0.164586847 | 0.240816631 | 1.911246033 | 0.003434489 | 0.104153587 | 0.683452994 | -0.549085978 |
|  |  |  |  | 171.3915 | 660.4616999 | 0.055526807 | 0.081294847 | 1.609358068 | 0.019661664 | 0.199157189 | 0.683029847 | -0.549979472 |
|  |  |  |  | 326.895 | 115.0585016 | 1.049940476 | 1.537679511 | 1.599477532 | 0.031431489 | 0.228098911 | 0.682808393 | -0.550447302 |
|  |  |  |  | 169.5815 | 687.4808317 | 0.151828988 | 0.222503743 | 1.595500926 | 0.018820115 | 0.196354037 | 0.682365993 | -0.551382346 |
|  |  |  |  | 453.272 | 378.9958122 | 0.005663447 | 0.008323504 | 1.585612072 | 0.044346124 | 0.248925403 | 0.680416195 | -0.555510615 |
|  |  | 2,4-Dibromophenol | 17.27692554 | 440.7585 | 252.9150287 | 0.023505735 | 0.03462305 | 1.677054013 | 0.015659619 | 0.184133292 | 0.678904245 | -0.558719988 |
|  |  |  |  | 33.0585 | 208.013859 | 0.024766106 | 0.036536152 | 1.604124074 | 0.015026175 | 0.181300701 | 0.677852057 | -0.56095766 |
|  |  |  |  | 246.705 | 99.02716166 | 0.191881011 | 0.283457365 | 1.637453622 | 0.016690461 | 0.188446871 | 0.676930763 | -0.562919813 |
|  |  |  |  | 423.785 | 272.0541566 | 0.006185419 | 0.00914061 | 1.670159342 | 0.036867517 | 0.237041027 | 0.676696518 | -0.56341913 |
|  |  |  |  | 363.0575 | 88.03945831 | 3.321537787 | 4.916451203 | 1.571320184 | 0.030754746 | 0.226821955 | 0.675596614 | -0.565765997 |
|  |  | Tetrafluoroethylene | 12.80360817 | 408.552 | 101.0235572 | 0.655134686 | 0.970145681 | 1.85946259 | 0.02350027 | 0.210114557 | 0.675295163 | -0.566409872 |
| Creatine | 0.930266 |  |  | 364.519 | 130.0612908 | 0.13514202 | 0.200381331 | 1.640370326 | 0.032774111 | 0.230514933 | 0.674424205 | -0.568271779 |
|  |  |  |  | 197.046 | 847.6472356 | 0.041448862 | 0.061509139 | 1.904870038 | 0.002804075 | 0.09658229 | 0.673865097 | -0.569468293 |
|  |  |  |  | 165.1895 | 742.5408569 | 1.019542582 | 1.514925734 | 1.735968502 | 0.013071492 | 0.171601528 | 0.672998392 | -0.571325038 |
| beta-D-Glucosamine | 0.506048231 |  |  | 409.007 | 214.0484811 | 0.110272556 | 0.163905989 | 1.867029636 | 0.004156889 | 0.110872018 | 0.672779292 | -0.571794794 |
|  |  |  |  | 157.261 | 722.5143527 | 0.133320507 | 0.198257102 | 2.043774684 | 0.000667653 | 0.0609167 | 0.672462707 | -0.572473834 |
|  |  |  |  | 231.1935 | 98.0237292 | 9.064756753 | 13.52997588 | 1.415101112 | 0.036665221 | 0.236744943 | 0.669975825 | -0.577819056 |
|  |  |  |  | 231.221 | 122.9750856 | 0.071648809 | 0.106987548 | 1.496938983 | 0.035786189 | 0.23542852 | 0.669692969 | -0.578428274 |
|  |  |  |  | 363.401 | 302.0730884 | 0.017960167 | 0.026899434 | 1.483464028 | 0.034278761 | 0.233051529 | 0.667678262 | -0.582775025 |
|  |  |  |  | 80.9166 | 689.497117 | 0.048740396 | 0.073002265 | 1.881017713 | 0.002182297 | 0.087012456 | 0.667655945 | -0.582823247 |
|  |  |  |  | 232.037 | 186.1134328 | 0.059191528 | 0.089265088 | 1.598819133 | 0.013168102 | 0.17211191 | 0.663098306 | -0.592705326 |
|  |  |  |  | 311.5115 | 84.04450988 | 0.02080916 | 0.031390545 | 1.631102131 | 0.030705618 | 0.226727636 | 0.662911722 | -0.593111332 |
|  |  | 6-[2,3-dihydroxy-5-({[3-hydroxy-6-(hydroxymethyl)-2,5-bis(3,4,5-trihydroxybenzoyloxy)oxan-4-yl]oxy}carbonyl)phenoxy]-3,4,5-trihydroxyoxane-2-carboxylic acid | -17.4556046 | 203.3195 | 813.5880923 | 0.075103754 | 0.113381749 | 1.596816792 | 0.025088818 | 0.214241727 | 0.662397209 | -0.5942315 |
|  |  |  |  | 363.345 | 277.0510226 | 0.002764292 | 0.00417631 | 1.463744702 | 0.039681978 | 0.240914178 | 0.661898203 | -0.595318741 |
|  |  |  |  | 61.4095 | 689.497241 | 0.067644768 | 0.102315185 | 1.866782281 | 0.003275349 | 0.102418446 | 0.661141035 | -0.596970035 |
|  |  |  |  | 157.236 | 723.5163326 | 0.056004247 | 0.084791644 | 2.035126425 | 0.000833813 | 0.064435329 | 0.660492522 | -0.598385868 |
|  |  |  |  | 478.892 | 350.9530021 | 0.025895933 | 0.039224738 | 1.38199443 | 0.046039176 | 0.252131282 | 0.660193919 | -0.599038245 |
|  |  |  |  | 208.578 | 172.0975101 | 0.034818725 | 0.052873818 | 1.701789144 | 0.029881263 | 0.225110973 | 0.658524889 | -0.602690127 |
|  |  |  |  | 456.518 | 188.056346 | 0.009158213 | 0.013927558 | 1.391041053 | 0.041070517 | 0.242670217 | 0.657560568 | -0.604804309 |
|  |  |  |  | 450.618 | 190.0354813 | 0.00737727 | 0.011223622 | 1.533794517 | 0.037415298 | 0.237830296 | 0.657298479 | -0.605379447 |
|  |  |  |  | 362.231 | 170.0425975 | 0.017580384 | 0.026810382 | 1.494437996 | 0.0285468 | 0.222350361 | 0.655730432 | -0.608825243 |
|  |  |  |  | 363.1635 | 275.054588 | 0.00816966 | 0.012491338 | 1.522313071 | 0.026491816 | 0.217712882 | 0.65402603 | -0.61258004 |
|  |  |  |  | 161.6785 | 847.6249734 | 0.143261684 | 0.219472536 | 1.530692125 | 0.026615854 | 0.218007242 | 0.652754493 | -0.615387612 |
|  |  |  |  | 166.065 | 730.5405898 | 0.075534335 | 0.115955479 | 1.573040935 | 0.026904968 | 0.218685833 | 0.651408074 | -0.618366492 |
|  |  |  |  | 169.595 | 689.4973476 | 0.295183623 | 0.455243193 | 1.849828054 | 0.004574355 | 0.11437166 | 0.648408649 | -0.625024761 |
|  |  | [6,7,8,11,12,13,22-heptahydroxy-3,16-dioxo-21-(3,4,5-trihydroxybenzoyloxy)-2,17,20-trioxatetracyclo[17.3.1.0tricosa-4,6,8,10,12,14-hexaen-23-yl]oxidanesulfonic acid | -5.3815875 | 166.957 | 715.5134314 | 0.690999452 | 1.067210429 | 1.812001792 | 0.006015512 | 0.126017811 | 0.647481914 | -0.627088199 |
|  |  |  |  | 211.895 | 847.5337898 | 0.031725293 | 0.049019595 | 1.848400474 | 0.004206263 | 0.111272578 | 0.647196137 | -0.627725099 |
|  |  |  |  | 191.748 | 161.0351638 | 0.05246549 | 0.081086405 | 1.833762211 | 0.014644541 | 0.179521549 | 0.6470319 | -0.628091254 |
|  |  |  |  | 166.955 | 714.5075812 | 1.610157425 | 2.501177178 | 1.752929816 | 0.009912031 | 0.152016232 | 0.643759842 | -0.635405511 |
| N-Acetylaspartylglutamic acid | 0.744536231 |  |  | 450.8975 | 303.0839703 | 0.049319247 | 0.076643785 | 1.646157861 | 0.015491125 | 0.183394008 | 0.643486585 | -0.636018022 |
|  |  |  |  | 38.2881 | 714.5075974 | 0.362837806 | 0.564112281 | 1.785743026 | 0.007322876 | 0.13373468 | 0.643201396 | -0.636657557 |
|  |  |  |  | 85.1674 | 688.492499 | 0.121699039 | 0.189492141 | 2.117230156 | 0.000194793 | 0.046863159 | 0.642237926 | -0.638820232 |
|  |  |  |  | 465.608 | 289.9499365 | 0.010363386 | 0.016168989 | 1.642345716 | 0.020151931 | 0.200716193 | 0.640942097 | -0.641734067 |
|  |  |  |  | 38.2594 | 767.5442172 | 0.448279412 | 0.699740435 | 1.619212963 | 0.019665536 | 0.199169707 | 0.640636713 | -0.642421617 |
|  |  | 3,5-Diiodo-L-tyrosine | 0.296378054 | 447.984 | 433.9890049 | 0.002915341 | 0.004552202 | 1.553870151 | 0.028364482 | 0.221958594 | 0.640424308 | -0.642900028 |
|  |  |  |  | 60.9644 | 686.4786505 | 0.101758535 | 0.15922043 | 1.894370695 | 0.002117488 | 0.085899208 | 0.639104758 | -0.645875666 |
| p-Cresol sulfate | 0.420437 |  |  | 23.92385 | 187.0066354 | 0.219986077 | 0.344772812 | 1.494575687 | 0.032449516 | 0.229944578 | 0.638060978 | -0.648233789 |
| 2-Hydroxyethanesulfonate | 0.958218538 |  |  | 153.603 | 124.9906458 | 0.128733527 | 0.201767771 | 1.69567027 | 0.011228008 | 0.160922237 | 0.638028196 | -0.648307913 |
|  |  |  |  | 193.835 | 240.0366446 | 0.095789273 | 0.150186837 | 1.530438028 | 0.034230877 | 0.232973415 | 0.637800725 | -0.648822358 |
| Threonic acid | 0.986624923 |  |  | 365.601 | 135.0291735 | 0.085496547 | 0.134109783 | 1.714448801 | 0.007329816 | 0.133770369 | 0.637511634 | -0.649476425 |
|  |  |  |  | 199.6015 | 810.5323797 | 0.27097952 | 0.425591206 | 1.319431565 | 0.021493474 | 0.204727761 | 0.636713157 | -0.651284519 |
| 2-(3,4-dihydroxy-5-methoxyphenyl)-3,5,7-trihydroxy-4H-chromen-4-one | 0.772343154 |  |  | 414.174 | 331.0437753 | 0.038562625 | 0.060686466 | 1.914731082 | 0.029198657 | 0.223721872 | 0.635440277 | -0.654171558 |
|  |  |  |  | 83.2151 | 820.6026995 | 0.171339999 | 0.269931822 | 1.235052375 | 0.049759393 | 0.258959915 | 0.634752871 | -0.655733079 |
|  |  |  |  | 215.5185 | 774.5311712 | 0.048664828 | 0.076744765 | 1.603902957 | 0.023875151 | 0.211048425 | 0.634112681 | -0.657188866 |
| Myricetin | 0.595781154 |  |  | 473.627 | 317.0282092 | 0.038958128 | 0.061454313 | 1.80821103 | 0.024961625 | 0.213913578 | 0.633936433 | -0.657589912 |
|  |  |  |  | 456.922 | 285.9977138 | 0.042569403 | 0.067163806 | 1.824567308 | 0.004105086 | 0.110444594 | 0.633814632 | -0.657867128 |
|  |  |  |  | 36.40945 | 775.5490952 | 0.389861761 | 0.615806082 | 1.140748043 | 0.04563125 | 0.251373226 | 0.633091767 | -0.65951346 |
|  |  |  |  | 403.195 | 278.0264618 | 0.0047855 | 0.007564101 | 2.189087506 | 0.000117639 | 0.042991888 | 0.632659374 | -0.660499137 |
|  |  |  |  | 38.2571 | 766.5407516 | 1.021878699 | 1.617056297 | 1.627846734 | 0.020083475 | 0.200501644 | 0.631937614 | -0.662145954 |
|  |  |  |  | 169.595 | 688.4927429 | 0.696838958 | 1.103881341 | 1.865962038 | 0.005370552 | 0.121309228 | 0.631262557 | -0.663687913 |
|  |  | 4-Bromophenylacetate | -0.732843562 | 367.749 | 216.051119 | 0.048610611 | 0.077478804 | 1.485237701 | 0.035610519 | 0.235159463 | 0.627405286 | -0.672530411 |
|  |  |  |  | 382.2895 | 88.03946957 | 0.759086429 | 1.212266509 | 1.704305935 | 0.018407063 | 0.194915708 | 0.626171245 | -0.675370837 |
|  |  |  |  | 364.566 | 283.1144923 | 0.01552087 | 0.024838597 | 1.979158733 | 0.00064867 | 0.060612913 | 0.624869049 | -0.678374213 |
|  |  |  |  | 368.781 | 276.0402668 | 0.005858601 | 0.009404576 | 1.74110223 | 0.017643538 | 0.19214052 | 0.622952205 | -0.682806617 |
|  |  |  |  | 457.141 | 236.0234398 | 0.017565978 | 0.028210741 | 1.662918692 | 0.013137862 | 0.171952639 | 0.622669854 | -0.683460661 |
|  |  |  |  | 355.92 | 299.0287388 | 0.005710015 | 0.009170717 | 1.694611643 | 0.011170563 | 0.160557888 | 0.622635655 | -0.683539901 |
|  |  |  |  | 154.545 | 774.5426431 | 0.04517503 | 0.07260335 | 1.7208981 | 0.017840405 | 0.192871056 | 0.622216879 | -0.684510564 |
| Adenine | 0.999997 |  |  | 242.1475 | 134.046362 | 6.619777678 | 10.64150538 | 1.63563407 | 0.02995695 | 0.225262131 | 0.622071544 | -0.684847582 |
|  |  | S-(PGA1)-glutathione | 7.196354841 | 341.8645 | 644.8011095 | 0.006922581 | 0.011141896 | 1.333669716 | 0.039760151 | 0.241015603 | 0.621310899 | -0.686612734 |
|  |  |  |  | 424.636 | 198.0168486 | 0.001432168 | 0.002313847 | 1.477982237 | 0.029935311 | 0.225218973 | 0.618955368 | -0.692092713 |
|  |  |  |  | 124.424 | 847.591056 | 0.015633264 | 0.025261458 | 1.623631718 | 0.014492743 | 0.17879796 | 0.61885834 | -0.692318889 |
|  |  |  |  | 354.0355 | 269.0183824 | 0.004147617 | 0.006706151 | 2.018739108 | 0.004897886 | 0.117362189 | 0.618479569 | -0.693202158 |
|  |  |  |  | 462.731 | 392.0779665 | 0.002937182 | 0.004752664 | 1.785658917 | 0.020704058 | 0.202410753 | 0.618007501 | -0.694303747 |
|  |  |  |  | 82.73665 | 722.5142515 | 0.01428944 | 0.023208927 | 1.521653512 | 0.03140769 | 0.2280547 | 0.615687232 | -0.699730445 |
|  |  |  |  | 364.779 | 331.0437582 | 0.042388109 | 0.068961976 | 1.926841422 | 0.022003313 | 0.206161922 | 0.614659133 | -0.702141526 |
|  |  | 2,3,4,7,8,9,15,21,22,23,28-undecahydroxy-14-(hydroxymethyl)-13,25,32,35-tetraoxaoctacyclo[14.13.3.3]pentatriaconta-1,3,5(30),6,8,10,19,21,23-nonaene-12,27,31,34-tetrone | -3.547119899 | 166.0375 | 729.5286924 | 0.0439986 | 0.071839092 | 1.797901249 | 0.010257595 | 0.154473537 | 0.612460414 | -0.707311496 |
|  |  | Fluo-3 | -15.88499082 | 169.186 | 770.5300525 | 0.200196012 | 0.326913357 | 1.704504302 | 0.010438282 | 0.155723372 | 0.612382479 | -0.707495089 |
|  |  |  |  | 179.354 | 166.0176018 | 0.159968565 | 0.261343936 | 1.632670962 | 0.029836878 | 0.225022068 | 0.612099777 | -0.708161251 |
|  |  | [6,7,8,11,12,13,22-heptahydroxy-3,16-dioxo-21-(3,4,5-trihydroxybenzoyloxy)-2,17,20-trioxatetracyclo[17.3.1.0tricosa-4,6,8,10,12,14-hexaen-23-yl]oxidanesulfonic acid | -5.701508428 | 38.3298 | 715.5132028 | 0.154248318 | 0.252344702 | 1.897776733 | 0.00306578 | 0.099961677 | 0.611260379 | -0.710141038 |
|  |  |  |  | 88.2349 | 767.5445612 | 0.145184022 | 0.237873171 | 1.582449266 | 0.047305867 | 0.25446033 | 0.610342149 | -0.712309871 |
|  |  |  |  | 464.678 | 555.0654982 | 0.003336806 | 0.005500553 | 1.743681107 | 0.017612854 | 0.19202569 | 0.606630967 | -0.721108949 |
|  |  |  |  | 482.424 | 284.9658463 | 0.015380911 | 0.025358081 | 1.195823477 | 0.048176551 | 0.256027038 | 0.606548691 | -0.721304632 |
|  |  |  |  | 341.84 | 271.0165711 | 0.006130362 | 0.010131169 | 1.717237961 | 0.0103741 | 0.155282106 | 0.605099158 | -0.724756517 |
|  |  | 2,2',4,4',5,5'-Hexabromodiphenyl ether | 12.51249557 | 32.1482 | 644.5989294 | 0.02923122 | 0.048707515 | 1.658736897 | 0.022587824 | 0.207749562 | 0.60013778 | -0.736634342 |
|  |  |  |  | 168.705 | 702.5077322 | 0.084961379 | 0.142800091 | 1.992858363 | 0.008506876 | 0.141112836 | 0.59496726 | -0.749117814 |
|  |  |  |  | 368.708 | 316.0442429 | 0.035868355 | 0.060346837 | 2.122297944 | 0.000558868 | 0.058951459 | 0.594370088 | -0.750566581 |
|  |  |  |  | 326.0445 | 119.9753045 | 0.02786031 | 0.047235447 | 1.709591747 | 0.042709001 | 0.245669356 | 0.589817844 | -0.761658624 |
|  |  |  |  | 289.145 | 204.0527379 | 0.018308705 | 0.031096913 | 1.45980482 | 0.042710514 | 0.24567244 | 0.588762788 | -0.764241605 |
|  |  |  |  | 211.308 | 862.5555547 | 0.089965713 | 0.152905299 | 1.138434374 | 0.042379264 | 0.244993949 | 0.58837538 | -0.765191216 |
|  |  |  |  | 276.507 | 191.0194124 | 0.004398776 | 0.007477758 | 1.809318425 | 0.011936863 | 0.165252578 | 0.588247932 | -0.76550375 |
|  |  |  |  | 30.2627 | 192.9808597 | 0.006030589 | 0.010300244 | 1.511642864 | 0.012479198 | 0.16837148 | 0.585480226 | -0.772307648 |
|  |  |  |  | 165.1895 | 743.5455694 | 0.338322791 | 0.580011439 | 1.989203266 | 0.01438586 | 0.178282887 | 0.583303653 | -0.777680986 |
|  |  |  |  | 457.023 | 393.0612275 | 0.00209235 | 0.003589758 | 1.326348369 | 0.036109545 | 0.235918508 | 0.582866647 | -0.778762244 |
|  |  |  |  | 363.009 | 286.0340831 | 0.03612632 | 0.062012075 | 2.047723486 | 0.000298554 | 0.05199697 | 0.582569118 | -0.779498869 |
|  |  |  |  | 440.44 | 239.9918209 | 0.036524947 | 0.062767785 | 1.830682409 | 0.016260337 | 0.186689439 | 0.581905943 | -0.781142115 |
| Gluconolactone | 0.709202846 |  |  | 138.0335 | 177.0401135 | 0.135023809 | 0.232597339 | 1.943864105 | 0.00408641 | 0.110288667 | 0.580504531 | -0.784620768 |
|  |  |  |  | 366.644 | 254.0073102 | 0.017050491 | 0.029376209 | 2.00378336 | 0.000762834 | 0.062953184 | 0.580418369 | -0.784834918 |
|  |  |  |  | 478.9605 | 191.0194085 | 0.153186343 | 0.264197332 | 1.83325052 | 0.016501022 | 0.187680092 | 0.579817901 | -0.786328219 |
|  |  |  |  | 326.1885 | 135.0292095 | 2.556295001 | 4.434376607 | 1.711486732 | 0.011644086 | 0.163500304 | 0.576472237 | -0.794676966 |
|  |  |  |  | 462.749 | 290.9470212 | 0.003981886 | 0.00692864 | 1.23606604 | 0.039894002 | 0.241188541 | 0.574699519 | -0.799120253 |
|  |  |  |  | 299.1505 | 428.1302313 | 0.010092721 | 0.017641515 | 1.655106391 | 0.02247958 | 0.207459968 | 0.572100579 | -0.805659291 |
|  |  |  |  | 216.7235 | 762.5233746 | 0.060438822 | 0.10573717 | 1.424178322 | 0.040263665 | 0.241661441 | 0.571594857 | -0.806935158 |
|  |  |  |  | 209.2045 | 873.5518471 | 0.295557373 | 0.519257806 | 1.630637378 | 0.017257872 | 0.1906779 | 0.569191969 | -0.813012788 |
|  |  |  |  | 326.224 | 137.033365 | 0.014166682 | 0.025069229 | 1.611556702 | 0.030000371 | 0.225348595 | 0.565102433 | -0.823415694 |
|  |  |  |  | 231.209 | 208.0285899 | 0.014780512 | 0.026188243 | 2.050633895 | 0.003393094 | 0.103712409 | 0.564394944 | -0.82522303 |
|  |  |  |  | 166.049 | 728.5252854 | 0.112039862 | 0.19893427 | 1.974001381 | 0.001370114 | 0.073698128 | 0.563200406 | -0.82827972 |
|  |  |  |  | 450.618 | 443.070709 | 0.008989571 | 0.015976646 | 1.728084468 | 0.005540466 | 0.12261974 | 0.562669495 | -0.829640344 |
|  |  |  |  | 342.74 | 212.0857465 | 0.066909376 | 0.118978116 | 1.71902095 | 0.012728689 | 0.169753662 | 0.562367083 | -0.830415944 |
|  |  |  |  | 471.208 | 232.9527598 | 0.082044957 | 0.146187055 | 1.488086257 | 0.049081725 | 0.257616346 | 0.56123271 | -0.833329 |
|  |  |  |  | 159.4605 | 792.5524473 | 0.095609055 | 0.170717964 | 1.646739832 | 0.022431577 | 0.20733091 | 0.560040971 | -0.836395721 |
|  |  |  |  | 162.495 | 698.5159573 | 0.015299319 | 0.027339544 | 1.782685752 | 0.007101532 | 0.132570426 | 0.559604023 | -0.83752176 |
|  |  |  |  | 215.5085 | 789.550895 | 0.074781102 | 0.134384697 | 2.107080798 | 0.000130855 | 0.043906516 | 0.556470367 | -0.845623231 |
|  |  | 1,4-Dichlorobenzene | -16.80595228 | 327.043 | 148.0068061 | 0.123122656 | 0.222171834 | 1.740432047 | 0.023941348 | 0.211211136 | 0.554177607 | -0.851579678 |
|  |  | 3,4,5-trihydroxy-6-({6,13,14-trihydroxy-3,10-dioxo-12-[5,6,7-trihydroxy-1-oxo-3-(1,2,3,4-tetrahydroxybutyl)-1H-isochromen-8-yl]-2,9-dioxatetracyclo[6.6.2.0,.0,]hexadeca-1(15),4,6,8(16),11,13-hexaen-7-yl}oxy)oxane-2-carboxylic acid | -17.95695066 | 158.9985 | 791.5410808 | 0.178343287 | 0.322338353 | 1.856555011 | 0.005097355 | 0.119084967 | 0.553279761 | -0.853918945 |
|  |  |  |  | 159.016 | 790.5380888 | 0.385633629 | 0.697637187 | 1.890968147 | 0.00365497 | 0.106391435 | 0.552771034 | -0.855246077 |
|  |  |  |  | 38.3893 | 728.5250359 | 0.023568925 | 0.042706363 | 1.93433445 | 0.002085579 | 0.08533672 | 0.551883224 | -0.857565062 |
|  |  |  |  | 641.283 | 135.0192439 | 0.01702244 | 0.030954215 | 1.582654031 | 0.047800569 | 0.255355144 | 0.549923164 | -0.862698037 |
|  |  |  |  | 244.759 | 172.0205692 | 0.012598132 | 0.022927955 | 1.621212275 | 0.048748532 | 0.257035909 | 0.549466001 | -0.863897879 |
|  |  |  |  | 155.4525 | 750.5446336 | 0.034177355 | 0.062444511 | 1.673739076 | 0.021802009 | 0.205601293 | 0.547323614 | -0.869533992 |
|  |  |  |  | 461.873 | 281.980007 | 0.004341901 | 0.007950221 | 1.626206288 | 0.014847054 | 0.18047268 | 0.546135877 | -0.87266816 |
|  |  |  |  | 215.119 | 788.5459825 | 0.182868454 | 0.336151557 | 1.304780989 | 0.015360706 | 0.182814823 | 0.544005971 | -0.878305609 |
| N-Acetyl-L-phenylalanine | 0.964927308 |  |  | 191.783 | 206.0822438 | 0.030480697 | 0.056376057 | 1.70620451 | 0.035699827 | 0.235296501 | 0.54066742 | -0.887186671 |
|  |  | 4-Bromocatechol | 20.97092721 | 289.5375 | 190.0179402 | 0.010738076 | 0.019874204 | 1.931958288 | 0.001699222 | 0.078589988 | 0.540302204 | -0.888161527 |
| ADP | 0.950241077 |  |  | 488.0075 | 426.0245072 | 0.049992176 | 0.092666162 | 1.467292591 | 0.041028534 | 0.242618503 | 0.539486853 | -0.890340292 |
|  |  |  |  | 457.291 | 300.9966723 | 0.012385614 | 0.02300759 | 2.173555722 | 1.78593E-05 | 0.019974742 | 0.538327289 | -0.893444535 |
|  |  |  |  | 190.7635 | 205.0286867 | 0.0903972 | 0.168073044 | 1.855142598 | 0.048056883 | 0.255813945 | 0.537844722 | -0.894738375 |
|  |  |  |  | 37.4386 | 722.5139666 | 0.034980644 | 0.06504131 | 1.868422542 | 0.005675967 | 0.123627497 | 0.537821952 | -0.894799453 |
|  |  |  |  | 158.111 | 724.5265312 | 0.035141249 | 0.065510912 | 1.342493656 | 0.047290913 | 0.254433088 | 0.53641825 | -0.898569775 |
|  |  |  |  | 163.452 | 702.5389237 | 0.023502833 | 0.043819414 | 1.532572639 | 0.047620294 | 0.255030491 | 0.536356637 | -0.898735492 |
|  |  | TG(24:1(15Z)/24:1(15Z)/o-18:0) | 22.39190369 | 44.00755 | 1042.840605 | 0.006518608 | 0.012209474 | 1.497965714 | 0.041945732 | 0.244095571 | 0.53389754 | -0.905365193 |
|  |  |  |  | 198.062 | 300.0402251 | 0.005641611 | 0.010625976 | 1.380082453 | 0.029153779 | 0.223628883 | 0.530926371 | -0.913416292 |
|  |  |  |  | 318.773 | 427.1263183 | 0.009086388 | 0.017129105 | 1.526070702 | 0.037054583 | 0.237312591 | 0.53046484 | -0.914670965 |
|  |  |  |  | 456.1025 | 206.9968238 | 0.006390795 | 0.012135253 | 1.798145237 | 0.004270875 | 0.111786982 | 0.526630552 | -0.925136873 |
| Creatinine | 0.985913846 |  |  | 366.066 | 112.0508045 | 0.017366924 | 0.033132704 | 1.449071279 | 0.028383912 | 0.222000519 | 0.524162585 | -0.931913719 |
|  |  |  |  | 368.8405 | 284.0180407 | 0.018907492 | 0.036112277 | 2.114983439 | 6.45801E-05 | 0.036762362 | 0.523575174 | -0.933531403 |
|  |  |  |  | 62.25025 | 700.5289914 | 0.011120347 | 0.021286514 | 1.503335217 | 0.008286086 | 0.139639322 | 0.522412798 | -0.936737854 |
|  |  |  |  | 471.239 | 214.9421054 | 0.028120533 | 0.053877107 | 1.517413127 | 0.034811226 | 0.233909092 | 0.521938442 | -0.938048431 |
|  |  |  |  | 189.545 | 137.0237374 | 0.021520021 | 0.041485952 | 1.878897479 | 0.003271539 | 0.102375577 | 0.518730326 | -0.94694338 |
|  |  |  |  | 61.8961 | 714.5075936 | 0.620969226 | 1.201107223 | 1.57282239 | 0.010967181 | 0.159250964 | 0.516997329 | -0.951771269 |
|  |  |  |  | 450.486 | 285.0731691 | 0.013246422 | 0.025941822 | 1.856766464 | 0.004766811 | 0.116181133 | 0.510620348 | -0.969677064 |
|  |  |  |  | 164.325 | 672.4998031 | 0.009208305 | 0.018043333 | 1.625866049 | 0.036580156 | 0.236619686 | 0.510343909 | -0.970458321 |
|  |  |  |  | 240.2295 | 160.0611114 | 0.030139983 | 0.060269332 | 2.092239862 | 0.000186491 | 0.046577411 | 0.500088231 | -0.999745443 |
|  |  |  |  | 39.1504 | 820.6027117 | 0.103249071 | 0.209203056 | 1.821605747 | 0.000984502 | 0.067711475 | 0.493535195 | -1.018775126 |
|  |  | Agrocin 84 | 8.629539043 | 168.7155 | 703.5127388 | 0.024542762 | 0.049766543 | 1.886444719 | 0.002316734 | 0.08920432 | 0.49315786 | -1.019878568 |
|  |  |  |  | 319.023 | 158.0274853 | 0.015551089 | 0.031561455 | 1.494011284 | 0.021628374 | 0.205111843 | 0.492724086 | -1.021148099 |
|  |  |  |  | 454.606 | 495.0859453 | 0.000681456 | 0.001383658 | 1.457155687 | 0.03832032 | 0.239095733 | 0.492503031 | -1.021795492 |
|  |  |  |  | 474.038 | 379.9800057 | 0.001214543 | 0.002521464 | 1.307273604 | 0.026035086 | 0.216611913 | 0.481681773 | -1.053847761 |
|  |  |  |  | 402.538 | 196.0225103 | 0.007700012 | 0.015987338 | 2.015342132 | 0.018323976 | 0.19462117 | 0.48163193 | -1.053997056 |
|  |  |  |  | 402.704 | 130.0501178 | 0.13151664 | 0.276233674 | 2.064400811 | 0.001485959 | 0.075018286 | 0.476106472 | -1.070643853 |
|  |  |  |  | 88.702 | 332.059321 | 0.003550676 | 0.007521906 | 1.79318958 | 0.013228521 | 0.172428832 | 0.472044768 | -1.083004405 |
|  |  | CoA | 17.06115301 | 38.2353 | 768.5544716 | 0.162272057 | 0.345152955 | 1.628327901 | 0.023467171 | 0.210031074 | 0.47014535 | -1.088821247 |
|  |  |  |  | 416.313 | 299.0177453 | 0.004493316 | 0.009610758 | 1.865708312 | 0.006719731 | 0.130435906 | 0.467529781 | -1.096869828 |
|  |  |  |  | 470.3935 | 454.0162866 | 0.00041657 | 0.000892693 | 1.468341613 | 0.046528229 | 0.253028448 | 0.466644027 | -1.099605664 |
|  |  |  |  | 198.445 | 843.6627715 | 0.019254351 | 0.041299041 | 1.634910035 | 0.014872938 | 0.180593095 | 0.466217877 | -1.100923769 |
|  |  |  |  | 39.0729 | 689.4972422 | 0.022736711 | 0.048881938 | 1.832887594 | 0.00667961 | 0.130201699 | 0.465135217 | -1.10427792 |
|  |  | S-Benzoate coenzyme A | -10.9565708 | 38.34015 | 872.6379264 | 0.020407449 | 0.044957974 | 1.650129806 | 0.02280752 | 0.208331316 | 0.4539228 | -1.139481139 |
| N-Acetyl-L-aspartic acid | 0.906473846 |  |  | 403.045 | 174.0403513 | 1.992075447 | 4.504392521 | 2.129322842 | 0.00126305 | 0.072314452 | 0.442251744 | -1.177060264 |
|  |  |  |  | 362.746 | 174.0225568 | 0.01151785 | 0.026081379 | 1.623105921 | 0.016491332 | 0.187640564 | 0.441611992 | -1.179148747 |
|  |  |  |  | 364.674 | 442.1685155 | 0.000530188 | 0.001222415 | 1.922910627 | 0.008069959 | 0.138150755 | 0.433721986 | -1.205157517 |
|  |  |  |  | 403.337 | 156.0295657 | 0.017367476 | 0.040155043 | 2.089932787 | 0.001076564 | 0.069424991 | 0.432510467 | -1.209193047 |
|  |  |  |  | 135.373 | 131.0706902 | 0.19127679 | 0.446165836 | 1.862308744 | 0.002107105 | 0.085717233 | 0.428712318 | -1.221918225 |
|  |  |  |  | 24.9195 | 187.0416417 | 0.13651249 | 0.320269173 | 1.951064788 | 0.010233624 | 0.154305944 | 0.426242991 | -1.230251984 |
|  |  |  |  | 344.1095 | 189.0340038 | 0.00311233 | 0.007309232 | 1.862608141 | 0.006471964 | 0.128957407 | 0.425807999 | -1.231725042 |
|  |  |  |  | 291.1475 | 158.0274761 | 0.018446587 | 0.043438917 | 1.229198564 | 0.015010516 | 0.181228802 | 0.424655786 | -1.235634187 |
|  |  |  |  | 402.908 | 176.044559 | 0.015757222 | 0.03715239 | 2.086088829 | 0.001399279 | 0.074046506 | 0.424124057 | -1.237441776 |
|  |  | (-)-Epigallocatechin 3-gallate 7-glucoside 4-glucuronide | 13.17838902 | 197.176 | 797.654175 | 0.004456701 | 0.010511754 | 1.137391942 | 0.044985317 | 0.25015439 | 0.423973078 | -1.237955437 |
|  |  |  |  | 403.232 | 259.0185202 | 0.004841186 | 0.011490254 | 1.72092905 | 0.028154213 | 0.221502213 | 0.421329761 | -1.24697827 |
|  |  |  |  | 402.9475 | 175.0436078 | 0.12246808 | 0.291401444 | 2.109108876 | 0.001860566 | 0.081076008 | 0.420272727 | -1.250602257 |
|  |  |  |  | 261.86 | 131.0537071 | 0.004936907 | 0.011875779 | 1.425332526 | 0.041468736 | 0.243156618 | 0.415712303 | -1.266342651 |
|  |  |  |  | 308.8475 | 356.1206464 | 0.003049634 | 0.00737964 | 1.651780412 | 0.019014077 | 0.197014931 | 0.413249778 | -1.274914051 |
|  |  |  |  | 364.288 | 452.1388674 | 0.000192903 | 0.000466869 | 1.132773374 | 0.042842536 | 0.245940967 | 0.413184942 | -1.275140416 |
|  |  |  |  | 308.083 | 231.0232768 | 0.002603406 | 0.006301864 | 1.601436393 | 0.020539619 | 0.20191264 | 0.413116785 | -1.275378418 |
|  |  | Tetranitromethane | 13.93651834 | 243.84 | 197.0427086 | 0.156124523 | 0.378760653 | 1.398104185 | 0.034011155 | 0.23261284 | 0.412198368 | -1.278589303 |
|  |  |  |  | 473.109 | 410.9748534 | 0.000663727 | 0.001636326 | 1.515273431 | 0.032350737 | 0.229769307 | 0.405619956 | -1.301799461 |
|  |  |  |  | 215.772 | 775.5328404 | 0.010492484 | 0.02598446 | 1.850374389 | 0.004437089 | 0.113061612 | 0.403798428 | -1.308292801 |
|  |  |  |  | 164.328 | 673.5029107 | 0.001363283 | 0.003397596 | 1.4168669 | 0.041538828 | 0.243241466 | 0.401249299 | -1.317429223 |
|  |  |  |  | 368.862 | 271.023036 | 0.002247597 | 0.005608755 | 1.839301 | 0.003702514 | 0.106850655 | 0.400730097 | -1.319297228 |
|  |  |  |  | 341.838 | 126.0552108 | 0.003791333 | 0.009535647 | 1.358916875 | 0.037437339 | 0.237861678 | 0.397595785 | -1.330625632 |
|  |  |  |  | 455.421 | 284.0180295 | 0.007193905 | 0.018384335 | 2.223939719 | 8.45164E-05 | 0.03977672 | 0.391306255 | -1.353629922 |
|  |  |  |  | 315.5595 | 173.0384555 | 0.005380892 | 0.013804567 | 1.480421364 | 0.038057386 | 0.23873292 | 0.389790704 | -1.359228409 |
|  |  | Luteolin 7-O-[beta-D-glucuronosyl-(1->2)-beta-D-glucuronide]-4'-O-beta-D-glucuronide | 18.41565553 | 200.293 | 815.6309782 | 0.002751871 | 0.007284042 | 1.700903128 | 0.028313195 | 0.22184773 | 0.37779457 | -1.404326129 |
|  |  |  |  | 277.553 | 158.0273877 | 0.018436583 | 0.048809884 | 1.652305732 | 0.008145598 | 0.138677051 | 0.377722327 | -1.404602032 |
|  |  |  |  | 364.53 | 436.1662611 | 0.000350461 | 0.000939203 | 1.606553537 | 0.004449088 | 0.113151019 | 0.373147516 | -1.422182014 |
|  |  |  |  | 467.25 | 318.0474719 | 0.001394731 | 0.003968646 | 1.549713887 | 0.006933087 | 0.131649199 | 0.351437542 | -1.50865978 |
|  |  |  |  | 415.246 | 326.0061509 | 0.000602368 | 0.001726634 | 1.816583344 | 0.015839808 | 0.184912892 | 0.348868236 | -1.519245846 |
|  |  |  |  | 456.5605 | 349.0716052 | 0.00110387 | 0.003418775 | 1.903065079 | 0.002552456 | 0.092953332 | 0.32288477 | -1.630908704 |
|  |  |  |  | 450.405 | 444.0748849 | 0.000349198 | 0.001097127 | 1.612210258 | 0.019370594 | 0.198206317 | 0.318283617 | -1.651615195 |
|  |  |  |  | 26.5895 | 417.2110767 | 0.025076389 | 0.081156609 | 1.766435352 | 0.03169652 | 0.228587921 | 0.308987633 | -1.694378997 |
|  |  |  |  | 167.8545 | 960.5932568 | 0.002963655 | 0.009618202 | 1.641705383 | 0.037149856 | 0.237450086 | 0.308129824 | -1.698389767 |
|  |  | Tienilic acid | -11.6411799 | 33.8564 | 332.1746214 | 0.022695217 | 0.075290123 | 1.871385723 | 0.021331541 | 0.204262236 | 0.301436844 | -1.730072328 |
|  |  |  |  | 463.6305 | 262.9540474 | 0.000877337 | 0.002917673 | 1.755420014 | 0.044051269 | 0.248350634 | 0.300697463 | -1.733615397 |
|  |  | 3,5-Dichloro-4-hydroxy-2-methoxy-6-methylbenzoic acid | -16.77012522 | 324.2775 | 252.0660662 | 0.000868266 | 0.002898061 | 1.672756459 | 0.034901158 | 0.234051959 | 0.299602436 | -1.738878743 |
|  |  |  |  | 213.72 | 800.5453111 | 0.007175953 | 0.024444486 | 1.460323738 | 0.007064091 | 0.13236836 | 0.293561201 | -1.76826679 |
|  |  | Notoginsenoside J | -23.28378996 | 378.8385 | 836.015234 | 0.002507653 | 0.008982893 | 1.629952256 | 0.044972282 | 0.250129556 | 0.279158683 | -1.840842666 |
|  |  |  |  | 450.5555 | 286.0770288 | 0.000291501 | 0.001130203 | 1.456000585 | 0.045973874 | 0.252010529 | 0.257919208 | -1.955008878 |
|  |  |  |  | 323.7515 | 388.1231577 | 0.000299122 | 0.001221531 | 1.733764772 | 0.03875038 | 0.239680859 | 0.244874452 | -2.02988583 |
|  |  |  |  | 197.073 | 911.7062275 | 0.001688225 | 0.007436043 | 1.541908827 | 0.031142753 | 0.227559131 | 0.227032739 | -2.139027743 |
|  |  |  |  | 210.463 | 875.5642597 | 0.012687656 | 0.059992408 | 1.547055401 | 0.047201366 | 0.254269724 | 0.21148769 | -2.241354407 |
|  |  |  |  | 475.8095 | 314.0289505 | 0.000256844 | 0.001515522 | 1.760487429 | 0.038327346 | 0.239105375 | 0.169475658 | -2.560850019 |
|  |  |  |  | 402.485 | 415.035605 | 0.000296281 | 0.001830081 | 2.016794852 | 0.001853631 | 0.080975074 | 0.161895133 | -2.62686848 |
|  |  |  |  | 450.618 | 627.1207401 | 0.000456888 | 0.002933714 | 1.789281973 | 0.000519234 | 0.058073773 | 0.155737072 | -2.682815684 |
|  |  |  |  | 125.786 | 248.0805736 | 0.017239825 | 0.161968329 | 1.571727874 | 0.025013252 | 0.214047052 | 0.10643948 | -3.231894731 |
|  |  |  |  | 450.469 | 518.1032605 | 8.27608E-05 | 0.000859689 | 2.192191186 | 0.006973232 | 0.131871615 | 0.096268397 | -3.376793922 |
|  |  |  |  | 197.428 | 967.6978601 | 8.27608E-05 | 0.002206324 | 1.860646074 | 0.040885741 | 0.242441979 | 0.037510748 | -4.736552162 |

#MS1 Primary mass spectrometry

MS2 Secondary mass spectrometry

PPM Exact quality number matching

RT Retention time

MZ Mass-to-charge ratio

VIP Variable importance values
